# Origin and diversification of the basic helix-loop-helix gene family in metazoans: insights from comparative genomics

**DOI:** 10.1186/1471-2148-7-33

**Published:** 2007-03-02

**Authors:** Elena Simionato, Valérie Ledent, Gemma Richards, Morgane Thomas-Chollier, Pierre Kerner, David Coornaert, Bernard M Degnan, Michel Vervoort

**Affiliations:** 1 Evolution et Développement des protostomiens, Centre de Génétique Moléculaire- UPR 2167 CNRS, 1, av. de la terrasse, 91198 Gif-sur-Yvette Cedex, France; 2 Belgian EMBnet Node – Laboratoire de Bioinformatique, Université Libre de Bruxelles, Institut de Biologie et de Médecine Moléculaires, Rue des Professeurs Jeener et Brachet 12, B-6041 Gosselies, Belgium; 3 School of Integrative Biology, University of Queensland, Brisbane, Qld 4072, Australia; 4 Vrije Universiteit Brussel, Laboratory for Cell Genetics, Pleinlaan 2, B-1050 Brussels, Belgium; 5 UFR de Biologie et Sciences de la Nature, Université Paris 7 – Denis Diderot, 2 place Jussieu, 75251 Paris Cedex 05, France

## Abstract

**Background:**

Molecular and genetic analyses conducted in model organisms such as *Drosophila *and vertebrates, have provided a wealth of information about how networks of transcription factors control the proper development of these species. Much less is known, however, about the evolutionary origin of these elaborated networks and their large-scale evolution. Here we report the first evolutionary analysis of a whole superfamily of transcription factors, the basic helix-loop-helix (bHLH) proteins, at the scale of the whole metazoan kingdom.

**Results:**

We identified *in silico *the putative full complement of bHLH genes in the sequenced genomes of 12 different species representative of the main metazoan lineages, including three non-bilaterian metazoans, the cnidarians *Nematostella vectensis *and *Hydra magnipapillata *and the demosponge *Amphimedon queenslandica*. We have performed extensive phylogenetic analyses of the 695 identified bHLHs, which has allowed us to allocate most of these bHLHs to defined evolutionary conserved groups of orthology.

**Conclusion:**

Three main features in the history of the bHLH gene superfamily can be inferred from these analyses: (i) an initial diversification of the bHLHs has occurred in the pre-Cambrian, prior to metazoan cladogenesis; (ii) a second expansion of the bHLH superfamily occurred early in metazoan evolution before bilaterians and cnidarians diverged; and (iii) the bHLH complement during the evolution of the bilaterians has been remarkably stable. We suggest that these features may be extended to other developmental gene families and reflect a general trend in the evolution of the developmental gene repertoires of metazoans.

## Background

The basic helix-loop-helix (bHLH) protein superfamily constitutes an ancient class of eukaryotic transcription factors that are found in fungi, plants and metazoans [1,2]. The bHLH transcription factors are named after their highly conserved domain (about 60 amino acids long) that consists of a DNA-binding basic region (b) followed by two α-helices separated by a variable loop region (HLH) [1]. Interaction between the helix regions of two different proteins leads to the formation of homodimeric or heterodimeric complexes, and the basic region of each partner recognizes and binds to a core hexanucleotide DNA sequence. Many bHLH proteins also include additional domains that are important for their activity as transcriptional regulators, such as 'leucine zipper', 'PAS' or 'orange' domains, which are mainly involved in protein-protein interactions [3-5]. In unicellular eukaryotes, such as *Saccharomyces cerevisiae*, bHLH proteins mainly regulate metabolic pathways [1,6]. In contrast, in metazoans and plants, the bHLH proteins are mainly involved in controlling developmental processes, in regulating the cell cycle, and in sensing environmental signals [1,2,7,8].

Because of the important functions that they display in various organisms, bHLH proteins have been the subject of a number of studies aimed at the identification of their full complement encoded by completely sequenced genomes. The putative full set of genes encoding bHLH proteins ('bHLH genes') has been reported for *Saccharomyces cerevisiae *(8 bHLH genes), *Drosophila melanogaster *(58), *Caenorhabditis elegans *(39), *Homo sapiens *(125), *Ciona intestinalis *(46), *Arabidopsis thaliana *(118 to 147), and *Oryza sativa *(131 to 167) [4,6,9-16]. In most of these studies, phylogenetic analyses of the amino acid sequences of the bHLHs were used to define orthologous families (that is groups of genes that derive from a common ancestor). In addition, these phylogenetic studies have enabled the definition of higher-order groups (named A, B, C, D, E and F) within the bHLH superfamily, which comprise evolutionarily related families of orthologs that share structural and biochemical properties [1,2,4,17,18]. In brief, groups A and B include bHLH proteins that bind core DNA sequences referred to as E boxes (CANNTG); respectively CACCTG or CAGCTG (group A) and CACGTG or CATGTTG (group B). Group C corresponds to the families of bHLH proteins known as 'bHLH-PAS', as they contain a 'PAS' domain in addition to the bHLH. They bind to ACGTG or GCGTG core sequences. Group D corresponds to HLH proteins that lack a basic domain and are hence unable to bind DNA. These proteins act as antagonists of group A bHLH proteins. Group E includes proteins related to the *Drosophila *Hairy and Enhancer of split bHLH (HER) proteins. These proteins bind preferentially to sequences referred to as N boxes (CACGCG or CACGAG). Most group E proteins also contain two characteristic domains in addition to the bHLH, the 'orange' domain and a WRPW peptide in their carboxyterminal part. Group F corresponds to the COE proteins, which lack a basic domain and are characterized by the presence of an additional domain, the COE domain, involved in both dimerization and DNA binding. Yeast and plant bHLHs are all included in group B [2].

**Figure 1:**
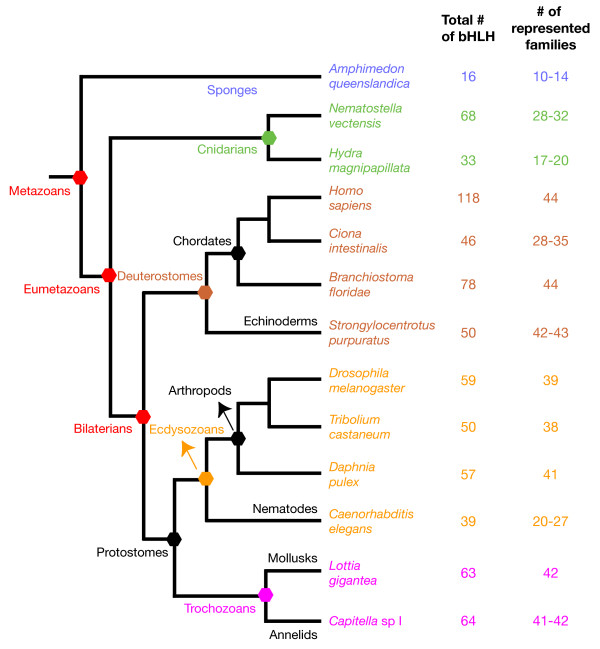
**Phylogenetic relationships between the species used in this study**. The tree is based on the current view of the phylogeny of the metazoans [73,74]. The total number of bHLHs and the number of represented bHLH metazoan families of orthologs in each genome is indicated. For the number of represented families, a range is indicated for most species, due to the uncertainty about the presence of some families in these species (see additional file 1 for details). The names of representative phylogenetic groups are indicated on the left of the nodes that define these different groups and along some of the terminal branches. The *Ciona intestinalis *data come from [12] and have not been reanalysed in this study.

Based on the analysis of the putative full-set of bHLH genes from *Drosophila melanogaster*, *Caenorhabditis elegans*, and *Homo sapiens*, we previously defined 44 orthologous families that include most of these bHLHs [4,11]. Of these, 43 include genes from *Homo sapiens*, and *Drosophila melanogaster *and/or *Caenorhabditis elegans*, indicating that these families were already present in the last common ancestor of these three species, thus of all bilaterians. This study led us to conclude that the diversification of the bHLH complement independently occurred in metazoans and plants and might have been related to the acquisition of multicellularity [11]. In the present study, we identified the putative full set of bHLHs encoded by several newly sequenced genomes of species representative of the main metazoan evolutionary lineages (Figure 1). Of special interest, we obtained data from three non-bilaterian species, two cnidarians and a demosponge, providing us with the opportunity to study the early evolutionary history of the bHLH superfamily in metazoans. Phylogenetic analysis of the sequence of these bHLHs allows us to conclude that (i) most of the bHLH bilaterian families of orthologs are present in cnidarians; (ii) only a few families are present in a demosponge species; (iii) the number of represented bHLH families of orthologs is remarkably similar in the different bilaterian lineages. We propose an evolutionary scenario in which the diversity of metazoan bHLHs has been established in two main steps, one during the early evolution of metazoans, before the divergence of demosponges from other metazoans, and the second, later, after this split but before the divergence of cnidarians and bilaterians.

## Results

### Derivation of the putative full set of bHLH genes of 12 metazoan species

We first developed a list of bHLH genes encoded by the three genomes (*Drosophila melanogaster*, *Caenorhabditis elegans*, and *Homo sapiens*) we had previously studied [4,11], using the current version of their genome assembly (Figure 1, additional file 1). While we found the same 39 bHLHs as in our previous study in *Caenorhabditis elegans*, we identified, in *Drosophila melanogaster*, one additional bHLH as compared to our published analysis, raising the total number of *Drosophila *bHLHs to 59. In *Homo sapiens*, our previous study was made on a draft assembly of the genome and a few bHLH genes that we identified cannot be found in the current assembly or correspond to pseudogenes. We found a total number of 118 bHLH genes in the current assembly of the genome of *Homo sapiens*.

We then used the sequences of all the bHLHs from *Homo sapiens *and *Drosophila melanogaster *to identify, through similarity searches using BLAST algorithm, the bHLHs encoded in the genomes of 9 additional species, the demosponge *Amphimedon queenslandica *(formerly named *Reniera *sp.), the cnidarians *Hydra magnipapillata *(a hydrozoan) and *Nematostella vectensis *(an anthozoan), the annelid *Capitella *sp. I (formerly known as *Capitella capitata*), the mollusk *Lottia gigantea*, the arthropods *Daphnia pulex *(a crustacean) and *Tribolium castaneum *(an insect), the echinoderm *Strongylocentrotus purpuratus*, and the chordate *Branchiostoma floridae *(a cephalochordate). These species were chosen because, together with the four metazoan species in which the putative full set of bHLHs has been previously determined (*Drosophila melanogaster*, *Caenorhabditis elegans*, *Ciona intestinalis*, and *Homo sapiens*; [4,11,12]), they provide a significant coverage of the main metazoan evolutionary lineages. The phylogenetic relationships between these species and the total number of bHLHs that have been found for each species are shown in Figure 1. Additional informations about the studied species and the status of the corresponding genome projects can be found in additional file 2. We identified a total number of 695 bHLH genes in the 12 aforementioned species. All the identified sequences can be found in additional file 3. In most cases, we were able to retrieve the complete bHLH domain. Given our extensive searches, we are confident that we obtained a significant coverage of the bHLH genes present in the genomes of all the studied species. We have used this very rich sampling of metazoan bHLH genes to better understand the evolution of this superfamily through phylogenetic analyses.

### Phylogenetic analyses of the bHLH genes

Carrying out evolutionary analyses of multigene families requires that orthologs, which have evolved by vertical descent from a common ancestor, are distinguished from paralogs, which arise by duplication and domain shuffling within a genome [19]. We therefore constructed phylogenetic trees to define metazoan families of orthologs. Given the large number of sequences, we independently analysed the sequences from the different species. We made 9 different multiple alignments, each comprising all the identified bHLHs from *Homo sapiens *and *Drosophila melanogaster *plus those from one of the nine other species. We also made two additional multiple alignments, one with the bHLHs from *Homo sapiens *and *Drosophila melanogaster *plus those from the two cnidarian species, *Nematostella vectensis *and *Hydra magnipapillata *and the other with the bHLHs from *Homo sapiens *and *Drosophila melanogaster *plus those from the two arthropod species, *Tribolium castaneum *and *Daphnia pulex*. These alignments allowed the study of specific relationships of bHLHs within cnidarians and arthropods. The multiple alignments were then used to construct phylogenetic trees. We used four different methods of phylogenetic reconstruction: distance (neighbour-joining; NJ), maximum parsimony (MP), maximum likelihood (ML), and Bayesian inference (BI). In general, the phylogenetic trees obtained by the different methods were congruent and displayed very similar topologies.

These phylogenetic trees were then used to define families of metazoan bHLH orthologs, using the same criterion as in our previous studies. That is, we defined bHLH families of orthologs as monophyletic groups which include sequences from different species and whose monophyly is consistent across the different phylogenetic methods and supported by bootstrap values and posterior marginal probabilities superior to 50% [4,11]. This criterion was relaxed for the Mesp, Myc, and Hairy/E(spl) families as discussed previously [4,11]. Genes that cannot be confidently assigned to any families (because for example their inclusion in a given family is weakly supported or not found in all the trees constructed by the different phylogenetic methods) are categorized as 'orphan' genes. The results of our analysis are consistent with those we previously obtained by using only the sequences from *Drosophila melanogaster*, *Caenorhabditis elegans*, and *Homo sapiens *[11]. The additional sequences included in the present study allowed us to define 2 additional metazoan families of orthologs, Delilah and MyoRa and b previously grouped into the MyoR family (additional file 1). We moreover grouped together two previously defined bilaterian families of orthologs, Hairy and E(spl), into a single one as there is no clear support for the monophyly of either of these families in our current analysis (a detailed phylogenetic study of these families will be published elsewhere). These analyses identified 45 metazoan bHLH families of orthologs (see additional file 1 for the full list and for the number of members in each species; the data that have been used to construct additional file 1 and Figure 1 can be found in additional files 4 to 15, where each file corresponds to one species and contains a table with all the bHLHs from this species, the family to which each bHLH belongs, and the statistical support for its inclusion in the corresponding family in the trees constructed using the four different phylogenetic methods).

In the case of the bilaterian species, at the exclusion of *Caenorhabditis elegans *(in which the bHLH complement is much derived; [11]), we were able to confidently allocate most of the bHLHs (more than 90%) to defined metazoan families of orthologs (additional file 1). In addition, we found, in each species, at least one member for most families (39 to 44 of the 45 families), confirming the very good coverage of the bHLH genes present in the genome of these species (Figure 1, additional file 1). This also confirms one of the main conclusions of our previous work, the fact that most bHLH families were already present in Urbilateria, the last common ancestor of all bilaterians (see Discussion) [11].

**Figure 2:**
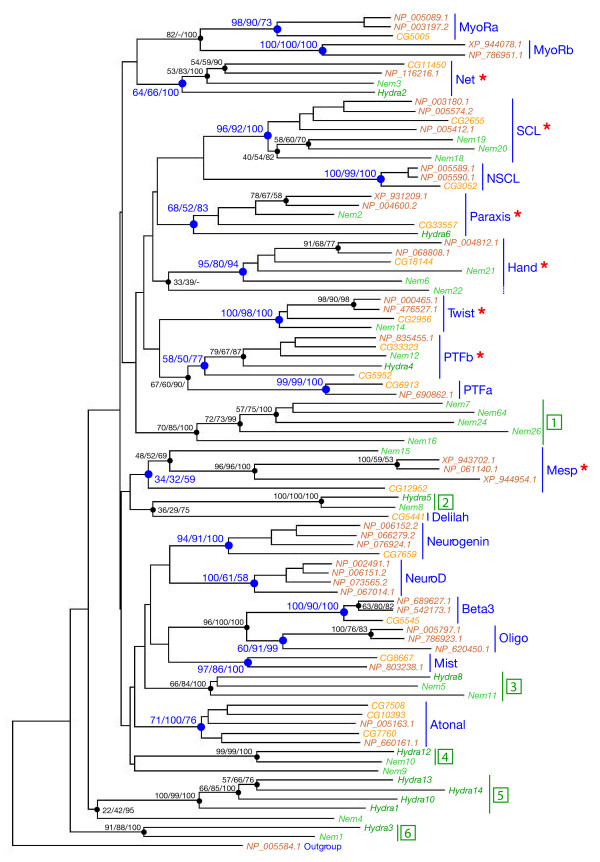
**Phylogenetic analysis of the cnidarian bHLHs related to the Twist and Atonal superfamilies**. The represented tree is a NJ tree, which has been rooted using a human bHLH sequence from the MyoD family as an outgroup. This tree is based on a multiple alignment that only includes the bHLH sequences of *Drosophila melanogaster *(in yellow) and *Homo sapiens *(in orange) which constitute the families belonging to the Atonal and Twist superfamilies [11] and their relatives in *Nematostella vectensis *(in light green) and *Hydra magnipapillata *(in dark green). The Atonal superfamily includes the Atonal, Neurogenin, NeuroD, Net, Oligo, Beta3, Delilah, and Mist families; the Twist superfamily includes the Twist, Paraxis, Hand, PTFa, PTFb, MyoRa, MyoRb, SCL, and NSCL families. Similar relationships (with similar statistical supports) were found when we used the entire set of bHLH genes of these 4 aforementioned species. The different metazoan families of orthologs are indicated in blue. Numbers above the internal branches are their statistical support values obtained with different methods of phylogenetic reconstruction: first number = bootstrap support in neighbour-joining analysis (10,000 bootstrap replicates); second number = bootstrap support in maximum-likelihood analysis (150 bootstrap replicates); third number = posterior probabilities in Bayesian inference-based analysis. Only statistical support values >50% are shown except for a few cases. Other internal branches (with statistical support <50%) should be considered unreliable. Statistical supports for the existence of the different families are shown in blue. We can see that 7 families (indicated by red asterisks) out of the 18 that are shown on this tree, have cnidarian members and that several cnidarian bHLHs cannot be assigned to any of these families. Some of these bHLHs form monophyletic groups comprising sequences from either one or both of the two cnidarian species (indicated by dark green numbers, from 1 to 6). Group 1 is comprised of only *Nematostella vectensis *sequences and groups 2 to 9 are comprised of at least one representative from both *Nematostella vectensis *and *Hydra magnipapillata*. Group 2 may correspond to Delilah genes, group 3 to Oligo/Beta3/Mist genes, and group 4 to NeuroD/Neurogenin genes (see additional files 13 and 14 for more details).

In the cnidarians *Nematostella vectensis *and *Hydra magnipapillata*, we found 68 and 33 bHLHs, respectively. The majority of these bHLHs could be clearly allocated to the families defined by bilaterian bHLHs (about 90% for *Nematostella vectensis *and about 70% for *Hydra magnipapillata*). The presence of cnidarian members for many families (Figure 1, additional file 1) indicates that these families were already present in the last common ancestor of cnidarians and bilaterians, the so-called Ureumetazoa. However, a significant proportion (10% to 33%) of the cnidarian bHLHs could not be confidently allocated to the defined families by our phylogenetic analyses. Most of these problematic bHLHs nevertheless belong to the higher-order group A (13/16 in *Nematostella vectensis*, 8/11 in *Hydra magnipapillata*) and tend to be associated with one or several group A families (especially to those families comprising the so-called Twist and Atonal superfamilies; [11]), albeit with low statistical support (Figure 2, additional files 13 and 14). As these families are also often lacking a clear cnidarian member (Figure 2, additional file 1), this suggests that the aforementioned 'problematic' cnidarian bHLHs may be divergent members of these families. Alternatively, these genes may constitute cnidarian specific families, as we observed monophyletic groups of *Nematostella vectensis *and *Hydra magnipapillata *genes (Figure 2, additional files 13 and 14). These monophyletic cnidarian-only families may be ancestral bHLH families of orthologs that have been lost in bilaterians or families that have been established in the cnidarian lineage after the divergence between cnidarians and bilaterians. The second scenario implies that the bHLHs from the higher-order group A have undergone partially independent diversifications in cnidarians and bilaterians.

**Figure 3:**
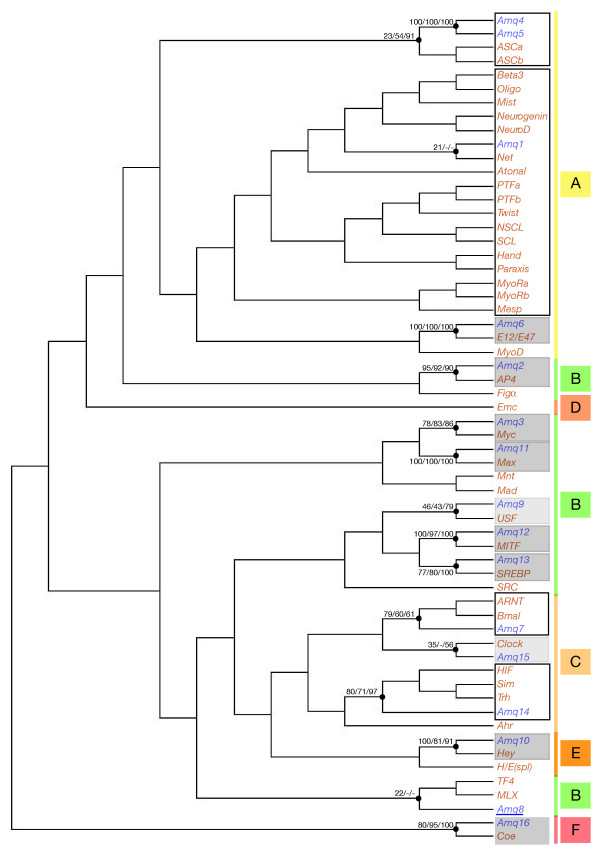
**Phylogenetic analysis of the bHLHs from *Amphimedon queenslandica***. The represented tree is a NJ tree whose rooting should be considered as arbitrary. This tree is based on a multiple alignment that includes all the *Amphimedon queenslandica *bHLH (named *Amq1 *to *Amq16*) sequences (in blue) and one representative sequence (from *Homo sapiens*) for each of the bilaterian families of orthologs (in orange). Similar relationships (with similar statistical supports) are found when we used the whole set of bHLHs genes from *Homo sapiens*. Numbers above the internal branches are as in Figure 2. We only show the statistical support for the internal branches that correspond to monophyletic groups concerning the *Amphimedon queenslandica *bHLHs. Higher-order groups (A to F) are shown. The *Amphimedon queenslandica *bHLHs that can be assigned to a family are in grey filled boxes (light grey denotes cases for which there is an uncertainty). *Amphimedon queenslandica *bHLHs that are associated with more than one family (and the concerned families) are in open black boxes. The single 'orphan' *Amphimedon queenslandica *bHLH is underlined.

We identified 16 bHLH genes in the genome of the demosponge *Amphimedon queenslandica*. In addition to the phylogenetic analysis of their bHLH domains, we assembled complete genes from the whole genome shotgun traces in order to detect potential additional domains that may help in the identification of the genes (see additional file 16 for gene assemblies). From these analyses, we were able to clearly assign 8 of the *Amphimedon queenslandica *bHLHs to previously defined bHLH eumetazoan families of orthologs (Figure 3; additional files 1 and 15), 5 from the high-order goup B (Myc, Max, MITF, SREBP, and AP4), 1 from the group A (E12/E47), one from the group E (Hey), and one from the group F (Coe). Two other *Amphimedon queenslandica *genes may be members of the group B USF and group C Clock families. Although the statistical support for their inclusion in these families was weak, we found an additional conserved region in the predicted genes whose presence supports their inclusion in the aforementioned families (additional file 15). Two other *Amphimedon queenslandica *bHLHs were strongly associated to more than one family, one to the ARNT and Bmal families and the other to the Hif, Sim, and Trh families (Figure 3, additional file 15). As the ARNT and Bmal families, and the Hif, Sim, and Trh families form well-supported monophyletic groups within the bHLH superfamily (Figure 3) [4,11], these *Amphimedon queenslandica *genes may be therefore single genes that are orthologous to several families in eumetazoans, i.e. correspond to an ancestral situation before the duplications that have lead to the families found in the Eumetazoa. A somewhat similar situation was found for three other *Amphimedon queenslandica *genes, with two closely-related genes being associated with the ASCa and ASCb families and one tending to be associated with the Atonal and Twist superfamilies (Figure 3, additional file 15).

### *Nematostella*-specific duplications of bHLH genes

**Figure 4:**
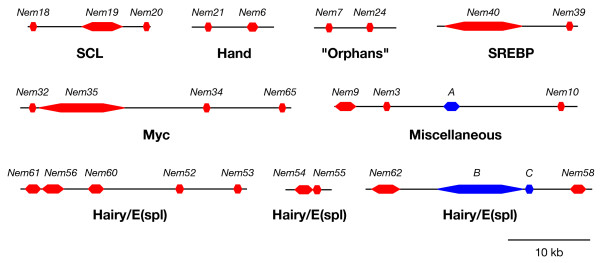
**Physical linkages detected between *Nematostella *bHLH genes**. The bHLH genes are in red, putative non-bHLH intervening genes are in blue. A = gw.168.63.1, gene similar to the uncharacterized *Drosophila CG13990 *gene; B = estExt_fgenesh1_pm.C_570004, gene similar to Q9QXA6 (Glycoprotein-associated amino acid transporter b0+AT1) from *Mus musculus*; C = fgenesh1_pg.scaffold_57000051, gene similar to *Drosophila CG18497*. See text and additional file 16 for details.

We have found that *Nematostella vectensis *has a high number of bHLH genes (68), higher than that of most of the bilaterian species we have studied (Figure 1). This large number of bHLHs in *Nematostella vectensis *is partially because many of the metazoan families of orthologs have more than one member in this species (about 30% of the families; additional file 1), while in most other species (with the exception of *Homo sapiens*), families with more than one member are rare (mean value is 13%; range 6% to 19%). This suggests that some of the *Nematostella vectensis *bHLH genes have been produced through lineage-specific duplications, as has also been suggested for homeobox genes in this species [20-22]. We therefore examined potential linkages between the different *Nematostella vectensis *bHLH genes that belong to families with more than one member (including the groups of 'orphan' bHLHs which significantly cluster together; Figure 2), using the recently available genome assembly. We identified 7 physical clusters of 2 to 5 bHLH genes without putative intervening genes and 2 additional clusters with one or two intervening genes (Figure 4, additional file 17). We therefore conclude that tandem duplications have had a significant impact on the *Nematostella vectensis *bHLH repertoire, in a similar way to that which has been reported for *Nematostella vectensis *homeobox genes [21,22].

### bHLHs from non bilaterians do not cluster with those of fungi

A recent report has shown that cnidarians may have retained some ancestral eukaryotic genes that have been lost in bilaterians [20]. We were interested to investigate whether some of the 'orphan' cnidarian (and by extension *Amphimedon queenslandica*) bHLHs may be such genes. We therefore retrieved a large set of bHLHs from fungi, which are among the closest relatives of eukaryotes (reviewed in [23]) and for which many completely sequenced genomes are available. We identified 98 bHLHs from 13 different species as summarized in the additional file 18. We first performed phylogenetic analyses on a multiple alignment of these sequences with those of *Homo sapiens *and *Drosophila melanogaster*. We observed that the fungi bHLHs form several fungi-specific families of orthologs that do not include bilaterian genes (not shown). We nevertheless noted that two of these families have a tendency to cluster with the MITF and SREBP families but with very low statistical support (not shown). We then made multiple alignments of the fungi bHLHs with those of *Amphimedon queenslandica *or those of *Nematostella vectensis *and *Hydra magnipapillata*; all analyses also included *Drosophila *bHLHs (used here as representative of bilaterians). We did not find any monophyletic groups that included both fungi and cnidarian and/or sponge bHLHs (not shown). We therefore conclude that the 'orphan' bHLHs from the cnidarians and from *Amphimedon queenslandica *are probably not ancestral eukaryotic bHLHs that were lost in bilaterians.

## Discussion

We report in this article the *in silico *identification of 695 putative bHLHs encoded by 12 sequenced metazoan genomes. Our choice of species represents a significant sampling of metazoan diversity, as we include representatives of the main metazoan evolutionary lineages (Figure 1). Our dataset therefore allows us to study the evolution of the bHLH superfamily at the scale of the whole metazoan kingdom. This is, to our knowledge, the first time that an entire gene superfamily has been studied at such a large scale. We must however caution that our analysis has probably not been carried out on a fully comprehensive dataset of bHLHs sequences. In most cases, our search for bHLH genes has been with unannotated genome assemblies or even on unassembled whole genome shotgun reads. It is therefore possible that we may have missed some bHLHs and/or that we may have included bHLH domains from some pseudogenes. Nevertheless, these data are sufficient for the purpose of this study, i.e. to obtain a qualitatively accurate assessment of the metazoan bHLH complement and of its evolution within this lineage.

### Ancestrality and stability of the bHLH complement in bilaterians

**Figure 5:**
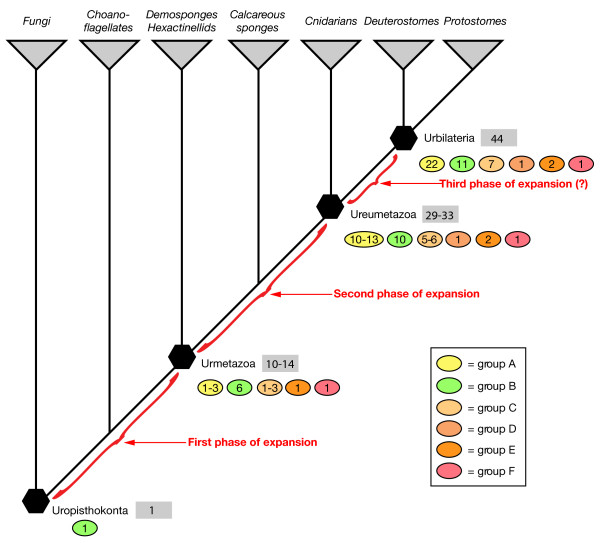
**A model for the evolution of the bHLH complement in metazoans**. A simplified phylogenetic tree of the opisthokonts is represented. The three main groups of opisthokonts – the fungi, the choanoflagellates and the metazoans – are indicated. For the metazoans, we have represented their main subdivisions: the two probable monophyletic groups of 'sponges', the demosponges + hexactinellids, and the calcareous sponges; the cnidarians; and the two main groups of bilaterians, the protostomes and the deuterostomes. Last common ancestors of opisthokonts (Uropisthokonta), metazoans (Urmetazoa), eumetazoans (Ureumetazoa), and bilaterians (Urbilateria) are represented by black polygons. The minimal number of bHLHs inferred to have been present in these ancestors is shown (light grey boxes). We also indicate the minimal inferred number of families for the different higher-order groups present in each last common ancestor.

We determined the existence of 45 different metazoan bHLH families of orthologs to which most bilaterian bHLHs can be allocated (additional file 1). Of these 45 families, 44 contain members from both the protostomes and deuterostomes. If we infer that every bHLH family shared by protostomes and deuterostomes was represented by at least a single ancestral sequence in their last common ancestor, an inference consistent with the phylogenetic analyses, then this ancestor (Urbilateria) possessed at least 44 different bHLHs (Figure 5). Since this ancestor which is estimated to have lived some 600–700 millions years ago [24,25], the bHLH complement in bilaterians is remarkably stable. Only a single new family of orthologs (Figalpha) has been added in one of the bilaterian evolutionary lineages and very few families have been lost (additional file 1). Indeed, with the exception of *Caenorhadditis elegans *and *Ciona intestinalis *(the low number of bHLHs found in these two species has been already discussed; see [4,11,12] and references therein), a quite similar number of represented families (ranging from 38 to 44) is found across the Bilateria (Figure 1).

In contrast, the total number of bHLHs representing each family of orthologs is much more variable between different species (ranging from 50 to 118). For example, *Branchiostoma floridae *has 28 bHLHs more than *Strongylocentrotus purpuratus *(78 versus 50) but each species has approximately the same number of families (44 versus 43). This difference in the total number of bHLHs is due to the specific expansion of a few families in *Branchiostoma floridae*: Hairy/E(spl) and Hey (17 genes in *Branchiostoma floridae *versus 5 in *Strongylocentrotus purpuratus*); MyoD (4 versus 2); MyoRa (4 versus 1); and PTFb (3 versus 1). Another significant example is that of *Drosophila melanogaster*, which has more bHLHs than *Daphnia pulex *(59 versus 57) but in which fewer families are represented (39 versus 41). The larger number of bHLHs in *Drosophila melanogaster *as compared to *Daphnia pulex *is mainly due the expansion of one family, Hairy/E(spl) (11 or 12 versus 5 or 6). We can thus conclude that the number of bHLHs found in a given species is a poor indicator of the diversity of its bHLH complement, as some families of orthologs have undergone species- or clade-specific expansions. This holds true for *Homo sapiens*, although in this case most of the families have undergone varying degrees of expansion; 36 of the 44 families (about 80%) have more than one member in *Homo sapiens *while in other bilaterians the mean value is about 18% (range 7% to 26%). The bHLH complement in other vertebrates [[11]; unpublished observations] is quite similar to that of *Homo sapiens*, indicating that an expansion of the bHLH complement occurred at some early stage of vertebrate evolution, probably related to large-scale duplication events, possibly of the whole genome (reviewed in [26]).

### Cnidarian bHLHs: a complex ancestral complement, lineage-specific duplications and losses

We have found that 29 to 33 of the 44 conserved bilaterian bHLH families of orthologs have cnidarian members (Figure 1). Following the same reasoning as we used to infer the minimal complement of bHLHs in Urbilateria, we can therefore conclude that the last common ancestor of cnidarians and bilaterians (Ureumetazoa) possessed at least 29 to 33 bHLHs (Figure 5). The estimate given here for the bHLH complement of Ureumetazoa almost certainly represents an underestimate because (i) it is likely that additional bHLHs will be found in the future (we may have missed some genes due to the fact that our searches were made within unfinished cnidarian genome assemblies); (ii) some cnidarian sequences that were included in this analysis could not be placed unambiguously into specific metazoan families of orthologs (and may represent divergent members of additional ancestral families); (iii) our analyses were done on the two cnidarian genomes available and we cannot exclude the possibility that some ancestral bHLHs may have been lost in both species. Ureumetazoa therefore possessed a high number and diversity of bHLHs, similar to that found in bilaterians. Although initially surprising, this is fully consistent with recent studies performed on other gene families, which indicate a complex eumetazoan ancestral complement, for example, of homeobox genes (at least 56; [22]) and Wnt genes (at least 11; [27]). Taken together, we infer that Ureumetazoa possessed an elaborate complement of developmental genes and as such may have been morphogenetically complex.

Besides these speculations on Ureumetazoa, what appears clear is that cnidarians, and in particular anthozoans such as *Nematostella vectensis*, are neither 'simple', nor 'primitive' organisms, as is still often assumed. *Nematostella vectensis *possesses 68 bHLHs, i.e significantly more than most invertebrate bilaterians (Figure 1). Several recent studies have shown that *Nematostella vectensis *also possesses 130 to 139 homeobox genes including probably 7 Hox genes [21,22,28], 14 Sox (Sry-related HMG-box) genes [29], 15 Fox (Forkhead domain) genes [29], 12 WNT genes [27], and 6 TGF-β genes and numerous antagonists of these [30-32]. For several gene families, the number of genes found in *Nematostella vectensis *is in fact higher than that found in invertebrate models such as *Drosophila melanogaster *and *Caenorhbaditis elegans *[21,22,27,33,34]. Interestingly, many of these genes are developmentally expressed in *Nematostella vectensis*, and these patterns show some similarities to those found in bilaterians (e.g. [27-32]). So far, very few bHLH encoding genes have been studied at the expression level in cnidarians [35,36]. It would thus be of particular interest to study whether the bHLH genes we have identified in *Nematostella vectensis *and *Hydra magnipapillata *are expressed during development and how these expression patterns may be related to those of their bilaterian counterparts.

It is also becoming clear, from this study and others, that in both the anthozoan *Nematostella vectensis *and the hydrozoan *Hydra magnipapillata*, several lineage specific genomic modifications have occurred. As already mentioned, the high number of bHLHs encoded by the genome of *Nematostella vectensis *is due to two factors, the inheritance of numerous (about 30) bHLH genes from Ureumetazoa ('ancestral complexity'), but also the expansion of some of the bHLH families through duplications ('lineage-specific duplications'). These two factors have contributed to the high level of complexity of the *Nematostella vectensis *genome, as has been previously suggested based on the analysis of a large set of expressed sequence tags (ESTs) [20] and is now clearly confirmed for two large multigenic superfamilies, the bHLH (this study) and the homeobox [21,22,34]. For both superfamilies, the lineage-specific duplicates are often physically linked, suggesting that they have been produced by tandem duplication, an apparently frequent event in the evolutionary lineage leading to *Nematostella vectensis*. It would now be interesting to see whether the duplications found in *Nematostella vectensis *are shared by other cnidarians. This is clearly not the case in *Hydra magnipapillata*, where only two metazoan families of orthologs (ASCa and Myc) contain more than one member (additional file 1). Although these families do also contain more than one member in *Nematostella vectensis*, the bHLHs from the ASCa and Myc families of the two cnidarians do not cluster in phylogenetic trees, suggesting independent duplications. *Hydra magnipapillata *has far fewer bHLHs than *Nematostella vectensis *(33 versus 68), again a similar situation to that reported for the homeobox genes (53 versus 139) [21]. From our analysis, we can conclude that *Hydra magnipapillata *has lost at least 11 ancestral types of bHLHs (Mesp, Twist, Hand, SCL, Mnt, SREBP, Mlx, TF4, Clock, Bmal, and Hey; additional file 1). By comparison, only a single metazoan family of orthologs (Ahr) has a member in *Hydra magnipapillata *but not in *Nematostella vectensis*, therefore indicating that this family has been lost in *Nematostella vectensis *(additional file 1). Loss of conserved developmental genes may be a general trend in *Hydra magnipapillata*, which could reflect a general simplification of the genome in this lineage.

### The *Amphimedon queenslandica *bHLH complement may reveal an intermediate step in the diversification of bHLHs in metazoans

The number of bHLHs and the number of represented families in *Amphimedon queenslandica *are markedly less than those found in cnidarians and bilaterians (Figure 1). This observation made on bHLHs can be extended to most developmental gene superfamilies: the genome of *Amphimedon queenslandica *encodes a considerable diversity of transcription factors and cell-cell molecules, but displays fewer members for most orthologs than cnidarians and bilaterians [[37]; B.M.D. et al., unpublished observations]. Prior to making any evolutionary interpretations from these observations, in particular in terms of reconstruction of ancestral properties, we must caution that (i) we only have data on one sponge species and we can therefore not rule out the possibility that *Amphimedon queenslandica *may have secondarily lost many genes (e.g. as we observed in cnidarians for *Hydra magnipapillata*) and (ii) the sponges represent a very complex and diversified phyla that is probably paraphyletic [38-40]. Therefore *Amphimedon queenslandica *(a demosponge from the Haplosclerida group) cannot be considered as representative for all sponges. In particular, calcisponges may be the sister-group of eumetazoans (Figure 5) [38-40], (i.e. more akin to eumetazoans than to the other sponges), and might significantly differ, in terms of genomic content, from demosponges such as *Amphimedon queenslandica*. Demosponges themselves constitute a complex group that is also probably not monophyletic. In particular the homoscleromorphs do not clearly cluster with the other demosponges in a recent analysis [41], and sponges from this group possess eumetazoan-like ultrastructural features, including an epithelium characterized by closely apposed cells with an underlying basement membrane and regularly distributed cell-cell junctions [42]. Interestingly, a report of a set of ESTs from a homoscleromorph species, *Oscarella carmela*, has been recently published [43]. We have identified 4 bHLH genes in these ESTs, with two corresponding to the Myc and Hey families also found in *Amphimedon queenslandica *and two to the Mad and Emc families for which no member can be found in *Amphimedon queenslandica *(unpublished observations). This highlights the importance for broad sampling of sponges in future studies.

Keeping this in mind, we can nevertheless infer that the last common ancestor of *Amphimedon queenslandica *and eumetazoans, and thus probably that of all metazoans (Urmetazoa) possessed a minimal number of 10 to 14 bHLHs (Figure 5). Indeed, we have found that 10 bHLH orthologs do contain clear *Amphimedon queenslandica *members (one member in each family; additional file 1), indicating that these families are ancestral to metazoans. In addition, 5 *Amphimedon queenslandica *bHLHs can be related to several metazoan families of orthologs but cannot be confidently allocated to any of these families: one to the ARNT and Bmal families; one to three different families (Hif, Sim, and Trh); one to many group A families, which constitute the so-called Atonal and Twist superfamilies; and two to the ASCa and ASCb families (Figure 3; additional file 1). These data suggest that at least 4 additional bHLH families are ancestral to metazoans, raising the probable minimal number of bHLHs of Urmetazoa to 14 (Figure 5).

**Figure 6:**
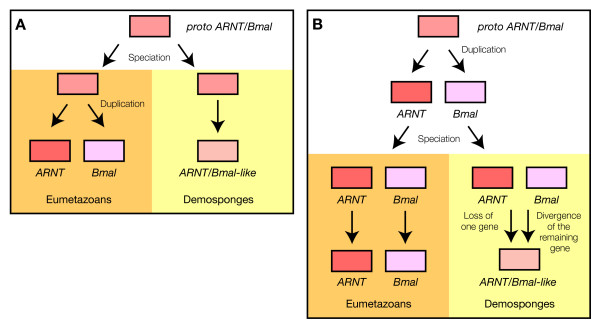
***Amphimedon queenslandica *bHLHs that are associated with more than one bHLH family**. We took, as an example, the case of the ARNT and Bmal families. In *Amphimedon queenslandica*, there is a single bHLH that clusters with both families as an outgroup to them (Figure 3). There are two main scenarios that can explain this situation. (A) The duplication that gives rise to the two families occurred after the divergence between demosponges and the other metazoans. No duplication occurred in *Amphimedon queenslandica*, which displays a single gene as in the ancestral situation. (B) The duplication that gives rise to the two families occurred before the divergence between demosponges and the other metazoans. *Amphimedon queenslandica *displays a single gene because one of the duplicates was lost. The remaining gene became quite divergent in such a way that it cannot be confidently related to either of the two families.

Our phylogenetic analyses indicate that a number of *Amphimedon queenslandica *bHLHs are related to more than one eumetazoan bHLH family. Their relationships with several families may either be a relic of an ancestral situation that predates eumetazoan-specific duplications or may be due to gene loss(es) that occurred in the evolutionary lineage leading to *Amphimedon queenslandica *(Figure 6). The second possibility seems to us less parsimonious and not as convincing, as we have to assume, in addition to the gene loss(es), that the single remaining gene diverged, such that it cannot be allocated with confidence to a recognized bHLH family, only to a larger bHLH clade. We therefore favour the first hypothesis and suggest that the *Amphimedon queenslandica *bHLH complement may represent an intermediate step in the diversification of bHLHs and that some of the duplication events required to generate the diversity of bHLHs found in other metazoans have occurred after the split between demosponges and the eumetazoans.

Noteworthy, there is a single bHLH in *Amphimedon queenslandica *(*Amq1*; additional file 15, Figure 3) that may represent the prototype of the numerous *atonal *and *twist*-related bHLHs found in eumetazoans, which are tissue-specific transcription factors involved in key developmental processes, such as neurogenesis and myogenesis (e.g. [4,44] and references therein). Deciphering the expression of the *Amphimedon queenslandica *relative would be therefore of particular interest. More generally, the reduced number of bHLHs found in *Amphimedon queenslandica *and the fact that gene expression analyses can be performed during the development of this species [37], offers an altogether unique opportunity to study the different functions assumed by this entire gene superfamily in a non-eumetazoan species. Most of the bHLH genes that we have been identified from the genome traces have now been cloned from developmental cDNAs, indicating that they are expressed during embryogenesis, and the analysis of their expression patterns is currently being performed [G.R and B.M.D, unpublished data].

### A general model for the diversification of the bHLH complement in metazoans

The model is summarized in Figure 5. Our phylogenetic analysis shows that there are no bHLH families of orthologs which are shared between fungi and metazoans, indicating an independent diversification of the bHLH complement in these two clades. The minimal number of bHLH genes in the last common ancestor of fungi and metazoans (Uropisthokonta) is therefore one. This single bHLH was most likely from higher-order B, as all of the fungi bHLHs are from this group (not shown). From our analysis of the bHLHs from *Amphimedon queenslandica*, we have inferred that the last common ancestor to all modern metazoans (i.e. Urmetazoa) possessed at least 10–14 different bHLHs, including members from 5 of the 6 higher-order groups of bHLHs. We can therefore infer that a first important expansion (both in terms of number and diversity) of the bHLH complement occurred after the divergence of fungal and metazoan lineages, but before the divergence of demosponges from the other metazoans. Although it is tempting to speculate that this expansion of the bHLH complement (and probably of many other gene families) has occurred in the early evolution of the metazoans and is related to the acquisition of multicellularity, we cannot rule out that it might have occurred even earlier. Indeed, fungi do not appear to be the sister-group to the Metazoa. There are other opisthokont eukaryotes more closely related, in particular the choanoflagellates [45]. Choanoflagellates are eukaryotes that share some similarities with one of the most prominent cell-type of sponges (the choanocyte) and are usually considered as unicellular, although some of them display some sort of multicellularity/colonialism [46]. It has been shown that choanoflagellates express relatives of a number of cell and adhesion protein families that have not previously been isolated from nonmetazoans, including cadherins, C-type lectins, several tyrosine kinases, and tyrosine kinase pathway components [47]. It is therefore possible that at least part of the diversification of the bHLH complement might have occurred in a common ancestor of the choanoflagellates and the metazoans. The completion of the genome projects conducted on two choanoflagellate species, *Monosiga brevicollis *and *Monosiga ovata*, will help to address this issue.

From our analysis of the bHLHs from the cnidarians *Nematostella vectensis *and *Hydra magnipapillata*, we have inferred that the ureumetazoan genome contained at least 29–33 different bHLHs, with representatives from each of the 6 higher-order groups of bHLHs. A second phase of expansion of the bHLH complement has therefore occurred after the demosponge lineage split from other animals but before the divergence of cnidarians and bilaterians. As demosponges probably do not represent the sister-group of the cnidarian + bilaterian clade, it would be worthwhile to study the bHLH complement in organisms with an intermediate position between demosponges and eumetazoans, such as calcareous sponges and ctenophores [38-40]. This second expansion particularly concerned the group A bHLH families, which contain tissue-specific transcription factors that control the determination, the specification and the differentiation of many cell types in bilaterians. We therefore suggest that this second expansion of the bHLH complement can be correlated with the increase in the diversity of cell types that occurred before eumetazoan cladogenesis.

Finally, a third phase of expansion, which almost exclusively concerns the group A bHLHs, may have occurred after the divergence of cnidarians and other eumetazoans. Indeed, there are several bHLH families of orthologs that have bilaterian but not cnidarian members, and several related cnidarian bHLHs that cannot be allocated to any of the defined families (Figure 2). Although we cannot rule out the possibility that the cnidarian bHLHs may be derived members of the aforementioned bHLH families of orthologs, the existence of these cnidarian 'orphan' bHLHs may indicate an independent expansion of the bHLH complement in bilaterian and cnidarian lineages.

## Conclusion

In this study, we identified the putative full set of bHLHs encoded by the newly sequenced genomes of 12 different species representative of the main metazoan evolutionary lineages, including three non bilaterian species, two cnidarians and a demosponge. Phylogenetic analysis of the sequence of the 695 identified bHLHs allowed us to conclude that (i) a first diversification of the bHLH superfamily has occurred in the pre-Cambrian and prior to metazoan cladogenesis; (ii) a second expansion of the bHLHs has occurred early in metazoan evolution before bilaterians and cnidarians diverged; and (iii) the bHLH complement during the evolution of the bilaterians has been remarkably stable. We suggest that these features may be extended to other developmental gene families and reflect a general trend in the evolution of the developmental gene repertoires of metazoans.

## Methods

### Retrieval of the bHLH sequences

As a starting point, we used the list of bHLH proteins we previously identified in *Homo sapiens*, *Drosophila melanogaster*, and *Caenorhabditis elegans *[11]. In order to confirm this list, we performed similarity searches for each sequence using TBLASTN and BLASTP algorithms [48] on the current assembly and the predicted proteins of the three aforementioned genomes maintained by the National Center for Biotechnology Information (NCBI) [49]. The whole set of *Homo sapiens *and *Drosophila melanogaster *bHLHs were then used for all the similarity searches using BLAST algorithm in the other species. The similarity searches using BLAST algorithm were performed at low stringency in order to obtain all possible bHLHs sequences, including divergent members relative to those of *Homo sapiens *and *Drosophila melanogaster*. All the retrieved sequences were used as queries to undertake similarity searches using BLAST algorithm against the NCBI protein database to ascertain that they corresponded to genuine bHLH domains.

In the case of *Strongylocentrotus purpuratus *and *Tribolium castaneum*, we used the BLAST server dedicated to these two species (on the web site of the Human Genome Sequencing Center, Baylor College of Medicine) [50]. We performed similarity searches using both TBLASTN (on the WGS assembly and on the individual WGS reads) and BLASTP (on the protein predictions) algorithms to retrieve the maximum number of bHLHs encoded by these two genomes. For all the other studied metazoan species, no genome assembly was available. We therefore downloaded the publicly available shotgun traces through the Trace Archive v3.0 at the NCBI [49], made banks of the traces for each species, and performed similarity searches using TBLASTN algorithm against these banks, all of them being hosted by the Belgian EMBnet Node [51]. We typically retrieved 6–7 overlapping traces that corresponded to the same aminoacid sequence. The nucleotide sequences of the traces were retrieved using the Ensembl Trace server [52] or from the banks hosted by the Belgian EMBnet node. Contigs were made for each set of overlapping traces by using CAP3 [53] at the web site of the Pôle de Bioinformatique de Lyon (PBIL) [54]. Aminoacid sequences were subsequently predicted using both Geneid [55] (through the web server at the Genome BioInformatics Research Lab, [56]), Genscan [57] (through the web server at the Institut Pasteur, [58]) and TBLASTN against the NCBI nr database. In some cases, only a part of the bHLH domain was retrieved. In these cases, the genomic sequences were extended through repeated steps of discontiguous Mega BLAST (at the NCBI) by using as query the left and right part of the previously assembled contig and then assembling new contigs with all the retrieved trace sequences (by using CAP3). The obtained contigs were analysed with Geneid, Genscan, and TBLASTN. This process was pursued until the whole bHLH domain was obtained or until it became impossible to further extend the contig. This workflow was successful in most cases. In the case of *Amphimedon queenslandica*, we systematically made larger gene assemblies using the same type of workflow. Preliminary phylogenetic analyses were then performed on the whole dataset for each species, and if some of the bHLH families of orthologs were found to lack a member in a given species, additional similarity searches using TBLASTN algorithm were performed specifically using members of the missing families as queries. This allowed us, in a very few cases, to retrieve additional bHLH domains missed in the first BLAST screen. Finally, each of the retrieved bHLH sequences were used to make similarity searches using BLASTP algorithm against the NCBI nr database (in order to see whether the corresponding gene may already have been cloned) and TBLASTN algorithm against the NCBI Expressed Sequence Tag (EST) database (in order to see whether there were ESTs corresponding to the identified bHLHs).

In the case of *Nematostella vectensis*, we made our initial similarity search using TBLASTN algorithm on the WGS reads. However, in a later phase of our work, genome assemblies became available on the web site of the US Department of Energy Joint Genome Institute [59] and on the StellaBase web site [60,61]. We therefore made additional similarity searches using BLAST algorithm on these genome assemblies and we assessed the presence of our identified bHLHs in the genome assemblies and retrieved the corresponding transcript models using TBLASTN algorithm. In order to study the putative physical linkages between some of the cnidarian bHLH genes, we used the genome browser available on the web site of the DOE Joint genome institute [59]. We also downloaded from the same source the nucleotide sequence of the corresponding scaffolds and used Geneid and Genscan [55,57] to predict the size and position of the putative genes contained in these scaffolds. In the case of the searches for the fungi bHLHs, we used the bHLHs from *Saccharomyces cerevisiae *that we had previously identified [11] and a selection of human bHLHs as queries for similarity searches using TBLASTN and BLASTP algorithms on the fungi WGS assemblies and protein predictions, respectively, that are available at the NCBI.

All the nucleotide sequences (traces, contigs, ESTs) we have isolated are available upon request.

### Phylogenetic analyses

Multiple alignments were performed with Clustal W [62] using the ClustalW web server at the Bioinformatics Center of the Kyoto University [63] or using ClustalW at the Belgian EMBnet Node and they were subsequently manually improved. Handling of the multiple alignments was done using SEAVIEW [64] or GeneDoc [65]. Unweighted maximum-parsimony (MP) and neighbour-joining (NJ) reconstructions were performed with the PAUP 4.0 program [66]. NJ analyses were done using the BioNJ algorithm [67] and 10,000 bootstrap replicates. MP analyses were performed with the following settings: heuristic search of over 250 bootstrap replicates; MAXTREES set at 3000, and other parameters set at default values. Maximum likelihood (ML) analyses were performed with PHYML [68]. PHYML analyses were performed using the Jones-Taylor-Thornton (JTT) amino-acid substitution model [69], the frequencies of amino acids being estimated from the data set, and rate heterogeneity across sites being modelled by two rate categories (one constant and eight γ-rates). The amino acid substitution model was chosen using ModelGenerator [70]. Statistical support for the different internal branches was assessed by bootstrap resampling (150 bootstrap replicates), as implemented in PHYML [68]. Bayesian inference was performed using the Markov chain Monte Carlo method as implemented in the MRBAYES (version 3) package [71,72]. We used the JTT substitution frequency matrix [69] with among-sites rate variation modelled by means of a discrete γ distribution with four equally probable categories. Two independent Markov chains were run, each containing from 1,500,000 to 3,000,000 Monte Carlo steps (depending on the number of steps required to get chain convergence). One out of every 250 trees was saved. The trees obtained in the two runs were meshed and the first 25% of the trees were discarded as 'burnin'. Majority consensus of the obtained trees was computed by means of the PAUP 4.0 program. Marginal probabilities at each internal branche were taken as a measure of statistical support.

All the alignments and the trees are available upon request.

## Authors' contributions

ES, VL, MV, GR, PK, and BDM retrieved the sequences used in the study. ES, VL, and MV made the sequence alignments. MV, ES, VL, MT-C and DC performed the phylogenetic analyses. MV conceived the study. MV and BMD participated in the design and coordination of the study. MV drafted the manuscript and all the authors participated in the editing of the manuscript. All the authors read and approved the final manuscript.

## Supplementary Material

Additional file 1 **The 45 families of metazoan bHLH defined by our phylogenetic analyses**. Families have been named as in our previous studies, i.e. according to the name (or its common abbreviation) of the first discovered or best-known member of the family [4,11]. The number of members per family in each of the different analysed genomes is reported. Each family has been tentatively assigned to the previously defined higher-order groups [2,4,11,17]. The number of 'orphan genes' is shown as a range due to the uncertainty in the allocation of some bHLHs to a given family. These uncertainties are indicated by '?' in the table. Explanations about all these uncertainties can be found in the additional files 4 to 15. In the cases of *Amphimedon queenslandica*, *Nematostella vectensis *and *Hydra magnipapillata*, additional explanations can be found in the main text and in Figures 2 and 3. The data from *Ciona intestinalis *come from [12] and have not been reanalysed in this study. Species abreviations: *H. sap *= *Homo sapiens*; *C. int *= *Ciona intestinalis*; *B. flo *= *Branchiostoma floridae*; *S. purp *= *Strongylocentrotus purpuratus*; *D. mel *= *Drosophila melanogaster*; *T. cas *= *Tribolium castaneum*; *D. pul *= *Daphnia pulex*; *C. ele *= *Caenorhabditis elegans*; *L. gig *= *Lottia gigantea*; *C*. sp = *Capitella *sp. I; *N. vec *= *Nematostella vectensis*; *H. mag *= *Hydra magnipapillata*; *A. que *= *Amphimedon queenslandica*. Click here for file

Additional file 2 **Information about the species used in this study**. Abbreviations are those we used in the figures and the other additional files. Taxonomy is according to the NCBI web site. Click here for file

Additional file 3 **List of all the sequences used in our study in fasta format**. For the identification of the sequences, we preferentially use, when available, the accession number of the proteins. In the cases where no protein sequences have been reported, i.e. most of the bHLHs we identified from whole genome shotgun traces, we indicate the identification of one of the trace sequences that encode the bHLH domain (the other trace identifications and the contigs we made are available upon request). In some cases, the bHLH was also found in ESTs and, in these cases, we also indicate the accession number(s) of the corresponding EST(s). For *Nematostella vectensis *and *Branchiostoma floridae*, we also indicate the identification of the transcripts models as determined using their genome assemblies. Click here for file

Additional file 4 **The bHLHs found in the genome of *Homo sapiens***. In this and the 11 following tables (additional files 4 to 15), we report all the bHLHs found in the indicated species, the family to which each of these metazoan orthologs bHLHs belong to, and the statistical support for their inclusion in a given family. For neighbour-joining (NJ), maximum parsimony (MP), and maximum likelihood (ML), the indicated numbers are bootstrap support values; for Bayesian inference (BI), the numbers are posterior probabilities. Details about the phylogenetic methods can be found in the Methods section. «?» indicate bHLHs that cannot be confidently assigned to any family (they are reported as 'orphan' genes in additional file 1). In this table (bHLHs from *Homo sapiens*), the phylogenetic studies have been done on a multiple alignment with all bHLHs from *Homo sapiens*, *Drosophila melanogaster *and *Branchiostoma floridae*. Click here for file

Additional file 5 **The bHLHs found in the genome of *Branchiostoma floridae***. The phylogenetic studies have been done on a multiple alignment with all bHLHs from *Homo sapiens*, *Drosophila melanogaster *and *Branchiostoma floridae*. Click here for file

Additional file 6 **The bHLHs found in the genome of *Strongylocentrotus purpuratus***. The phylogenetic studies have been done on a multiple alignment with all bHLHs from *Homo sapiens*, *Drosophila melanogaster *and *Strongylocentrotus purpuratus*. Click here for file

Additional file 7 **The bHLHs found in the genome of *Drosophila melanogaster***. The phylogenetic studies have been done on a multiple alignment with all bHLHs from *Homo sapiens*, *Drosophila melanogaster *and *Branchiostoma floridae*. Click here for file

Additional file 8 **The bHLHs found in the genome of *Tribolium castaneum***. The phylogenetic studies have been done on a multiple alignment with all bHLHs from *Homo sapiens*, *Drosophila melanogaster*, *Daphnia pulex *and *Tribolium castaneum*. Click here for file

Additional file 9 **The bHLHs found in the genome of *Daphnia pulex***. The phylogenetic studies have been done on a multiple alignment with all bHLHs from *Homo sapiens*, *Drosophila melanogaster*, *Tribolium castaneum *and *Daphnia pulex*. Click here for file

Additional file 10 **The bHLHs found in the genome of *Caenorhabditis elegans***. The phylogenetic studies have been done on a multiple alignment with all bHLHs from *Homo sapiens*, *Drosophila melanogaster*, and *Caenorhabditis elegans*. Click here for file

Additional file 11 **The bHLHs found in the genome of *Lottia gigantea***. The phylogenetic studies have been done on a multiple alignment with all bHLHs from *Homo sapiens*, *Drosophila melanogaster *and *Lottia gigantea*. Click here for file

Additional file 12 **The bHLHs found in the genome of *Capitella *sp. I**. The phylogenetic studies have been done on a multiple alignment with all bHLHs from *Homo sapiens*, *Drosophila melanogaster *and *Capitella sp I*. Click here for file

Additional file 13 **The bHLHs found in the genome of *Nematostella vectensis***. The phylogenetic studies have been done on a multiple alignment with all bHLHs from *Homo sapiens*, *Drosophila melanogaster*, *Hydra magnipapillata *and *Nematostella vectensis*. Click here for file

Additional file 14 **The bHLHs found in the genome of *Hydra magnipapillata***. The phylogenetic studies have been done on a multiple alignment with all bHLHs from *Homo sapiens*, *Drosophila melanogaster*, *Hydra magnipapillata *and *Nematostella vectensis*. Click here for file

Additional file 15 **The bHLHs found in the genome of *Amphimedon queenslandica***. The phylogenetic studies have been done on a multiple alignment with all bHLHs from *Homo sapiens*, *Drosophila melanogaster *and *Amphimedon queenslandica*. Click here for file

Additional file 16 **Gene assemblies for the bHLHs from *Amphimedon queenslandica***. Nucleotide sequences of the contigs we assembled for each bHLH gene are shown together with the corresponding predicted proteins using Genscan and Geneid. In some cases, we also report additional sequences from EST data and from PCR products. Click here for file

Additional file 17 **Summary of the study of putative physical linkages between bHLH genes from *Nematostella vectensis***. In this table, we report the different bHLH genes that are physically linked, the families and the genomic scaffolds to which they belong, as well as their position in these scaffolds. Click here for file

Additional file 18 **The bHLHs in fungi**. In this table, we report the list of the studied species, their taxonomy and the total number of bHLHs found in each species. Click here for file
