# Supplementary material for: Origin and diversification of the basic helix-loop-helix gene family in metazoans: insights from comparative genomics
Source: BMC Evol Biol. 2007 Mar 2;7:33. doi: 10.1186/1471-2148-7-33 (PMC1828162; doi:10.1186/1471-2148-7-33)
Supplement: Additional file 3 — List of all the sequences used in our study in fasta format. For the identification of the sequences, we preferentially use, when available, the accession number of the proteins. In the cases where no protein sequences have been reported, i.e. most of the bHLHs we identified from whole genome shotgun traces, we indicate the identification of one of the trace sequences that encode the bHLH domain (the other trace identifications and the contigs we made are available upon request). In some cases, the bHLH was also found in ESTs and, in these cases, we also indicate the accession number(s) of the corresponding EST(s). For Nematostella vectensis and Branchiostoma floridae, we also indicate the identification of the transcripts models as determined using their genome assemblies. [file 1471-2148-7-33-S3.doc]

***Homo sapiens***

>gi|58219048|ref|NP_001010926.1| hairy and enhancer of split 5 [Homo sapiens]

RLRKPVVEKMRRDRINSSIEQLKLLLEQEFARHQPNSKLEKADILEMAVSYLKHSK

>gi|7657154|ref|NP_055386.1| hairy/enhancer-of-split related with YRPW motif-like [Homo sapiens]

RKKRRGIIEKRRRDRINSSLSELRRLVPTAFEKQGSSKLEKAEVLQMTVDHLKMLH

>gi|5031763|ref|NP_005515.1| hairy and enhancer of split 1 [Homo sapiens]

ASEHRKSSKPIMEKRRRARINESLSQLKTLILDALKKDSSRHSKLEKADILEMTVKHLRNLQ

>gi|71274142|ref|NP_001025058.1| Hey-like transcriptional repressor [Homo sapiens]

SRVRTPVSHKVIEKRRRDRINRCLNELGKTVPMALAKQSSGKLEKAEILEMTVQYLRALH

>gi|6912414|ref|NP_036391.1| hairy/enhancer-of-split related with YRPW motif 2 [Homo sapiens]

TSQIMARKKRRGIIEKRRRDRINNSLSELRRLVPTAFEKQGSAKLEKAEILQMTVDHLKMLQ

>gi|66912172|ref|NP_001019769.1| hairy and enhancer of split 3 [Homo sapiens]

HLQISKPLMEKKRRARINVSLEQLKSLLEKHYSHQIRKRKLEKADILELSVKYMRSLQ

>gi|63055047|ref|NP_061962.2| hairy and enhancer of split homolog 2 [Homo sapiens]

ELRKSLKPLLEKRRRARINQSLSQLKGLILPLLGRENSNCSKLEKADVLEMTVRFLQELP

>gi|20149603|ref|NP_036390.2| hairy/enhancer-of-split related with YRPW motif 1 [Homo sapiens]

QILARKRRRGIIEKRRRDRINNSLSELRRLVPSAFEKQGSAKLEKAEILQMTVDHLKMLH

>gi|10863967|ref|NP_066993.1| hairy and enhancer of split 4 [Homo sapiens]

EHRKSSKPVMEKRRRARINESLAQLKTLILDALRKESSRHSKLEKADILEMTVRHLRSLR

>gi|4503299|ref|NP_003661.1| basic helix-loop-helix domain containing, class B, 2 [Homo sapiens]

KETYKLPHRLIEKKRRDRINECIAQLKDLLPEHLKLTTLGHLEKAVVLELTLKHVKALT

>gi|13540521|ref|NP_110389.1| basic helix-loop-helix domain containing, class B, 3 [Homo sapiens]

KDTYKLPHRLIEKKRRDRINECIAQLKDLLPEHLKLTTLGHLEKAVVLELTLKHLKALT

>gi|14211899|ref|NP_115969.1| hairy and enhancer of split 7 [Homo sapiens]

RDGPKMLKPLVEKRRRDRINRSLEELRLLLLERTRDQNLRNPKLEKAEILEFAVGYLRE

>gi|21361763|ref|NP_061115.2| hairy and enhancer of split 6 [Homo sapiens]

TRGDRKARKPLVEKKRRARINESLQELRLLLAGAEVQAKLENAEVLELTVRRVQGVL

>gi|32171182|ref|NP_002158.2| inhibitor of DNA binding 3 [Homo sapiens]

SRLRELVPGVPRGTQLSQVEILQRVIDYILDLQ

>gi|31982933|ref|NP_002157.2| inhibitor of DNA binding 2 [Homo sapiens]

SKLKELVPSIPQNKKVSKMEILQHVIDYILDLQ

>gi|4504573|ref|NP_001537.1| inhibitor of DNA binding 4, dominant negative helix-loop-helix protein [Homo sapiens]

SRLRRLVPTIPPNKKVSKVEILQHVIDYILDLQ

>gi|31317299|ref|NP_002156.2| inhibitor of DNA binding 1 isoform a [Homo sapiens]

SRLKELVPTLPQNRKVSKVEILQHVIDYIRDLQ

>gi|51972228|ref|NP_001004311.1| factor in the germline alpha [Homo sapiens]

LERRRVANAKERERIKNLNRGFARLKALVPFLPQSRKPSKVDILKGATEYIQVLS

>gi|21553337|ref|NP_660161.1| atonal homolog 7 [Homo sapiens]

AARRRLAANARERRRMQGLNTAFDRLRRVVPQWGQDKKLSKYETLQMALSYIMALT

>gi|4885075|ref|NP_005163.1| atonal homolog 1 [Homo sapiens]

QKQRRLAANARERRRMHGLNHAFDQLRNVIPSFNNDKKLSKYETLQMAQIYINALS

>gi|14249530|ref|NP_116216.1| atonal homolog 8 [Homo sapiens]

QQTRRLLANARERTRVHTISAAFEALRKQVPCYSYGQKLSKLAILRIACNYILSLA

>gi|22748877|ref|NP_689627.1| basic helix-loop-helix domain containing, class B, 5 [Homo sapiens]

QKALRLNINARERRRMHDLNDALDELRAVIPYAHSPSVRKLSKIATLLLAKNYILMQA

>gi|57529222|ref|NP_542173.1| basic helix-loop-helix domain containing, class B, 4 [Homo sapiens]

QRSLRLSINARERRRMHDLNDALDGLRAVIPYAHSPSVRKLSKIATLLLAKNYILMQA

>gi|38455396|ref|NP_006152.2| neurogenin 1 [Homo sapiens]

RRSRRVKANDRERNRMHNLNAALDALRSVLPSFPDDTKLTKIETLRFAYNYIWALA

>gi|31077092|ref|NP_076924.1| neurogenin 2 [Homo sapiens]

KKTRRLKANNRERNRMHNLNAALDALREVLPTFPEDAKLTKIETLRFAHNYIWALT

>gi|68989258|ref|NP_066279.2| neurogenin 3 [Homo sapiens]

RRSRRKKANDRERNRMHNLNSALDALRGVLPTFPDDAKLTKIETLRFAHNYIWALT

>gi|4505377|ref|NP_002491.1| neurogenic differentiation 1 [Homo sapiens]

FKLRRMKANARERNRMHGLNAALDNLRKVVPCYSKTQKLSKIETLRLAKNYIWALS

>gi|10863999|ref|NP_067014.1| neurogenic differentiation 4 [Homo sapiens]

FRARRVKANARERTRMHGLNDALDNLRRVMPCYSKTQKLSKIETLRLARNYIWALS

>gi|21314638|ref|NP_006151.2| neurogenic differentiation 2 [Homo sapiens]

SKLRRQKANARERNRMHDLNAALDNLRKVVPCYSKTQKLSKIETLRLAKNYIWALS

>gi|27475985|ref|NP_073565.2| neurogenic differentiation 6 [Homo sapiens]

VKFRRQEANARERNRMHGLNDALDNLRKVVPCYSKTQKLSKIETLRLAKNYIWALS

>gi|29126247|ref|NP_803238.1| class II bHLH protein MIST1 [Homo sapiens]

DSSIQRRLESNERERQRMHKLNNAFQALREVIPHVRADKKLSKIETLTLAKNYIKSLT

>gi|41281695|ref|NP_620450.1| oligodendrocyte transcription factor 1 [Homo sapiens]

QQLRRKINSRERKRMQDLNLAMDALREVILPYSAAHCQGAPGRKLSKIATLLLARNYILLLG

>gi|17978475|ref|NP_005797.1| oligodendrocyte lineage transcription factor 2 [Homo sapiens]

PELQQLRLKINSRERKRMHDLNIAMDGLREVMPYAHGPSVRKLSKIATLLLARNYILMLT

>gi|28411948|ref|NP_786923.1| oligodendrocyte transcription factor 3 [Homo sapiens]

QDLQQLRLKINGRERKRMHDLNLAMDGLREVMPYAHGPSVRKLSKIATLLLARNYILMLT

>gi|23097242|ref|NP_690862.1| nephew of atonal 3 [Homo sapiens]

VITYAQRQAANIRERKRMFNLNEAFDQLRRKVPTFAYEKRLSRIETLRLAIVYISFMT

>gi|30039710|ref|NP_835455.1| pancreas specific transcription factor, 1a [Homo sapiens]

AELQQLRQAANVRERRRMQSINDAFEGLRSHIPTLPYEKRLSKVDTLRLAIGYINFLS

>gi|4507741|ref|NP_000465.1| twist [Homo sapiens]

EELQTQRVMANVRERQRTQSLNEAFAALRKIIPTLPSDKLSKIQTLKLAARYIDFLY

>gi|17981708|ref|NP_476527.1| twist homolog 2 [Homo sapiens]

EELQSQRILANVRERQRTQSLNEAFAALRKIIPTLPSDKLSKIQTLKLAARYIDFLY

>gi|12545384|ref|NP_068808.1| basic helix-loop-helix transcription factor HAND2 [Homo sapiens]

RPVKRRGTANRKERRRTQSINSAFAELRECIPNVPADTKLSKIKTLRLATSYIAYLM

>gi|4758506|ref|NP_004812.1| basic helix-loop-helix transcription factor HAND1 [Homo sapiens]

RLGRRKGSGPKKERRRTESINSAFAELRECIPNVPADTKLSKIKTLRLATSYIAYLM

>gi|5031943|ref|NP_005589.1| nescient helix loop helix 1 [Homo sapiens]

RRATAKYRTAHATRERIRVEAFNLAFAELRKLLPTLPPDKKLSKIEILRLAICYISYLN

>gi|5031945|ref|NP_005590.1| nescient helix loop helix 2 [Homo sapiens]

RRATAKYRSAHATRERIRVEAFNLAFAELRKLLPTLPPDKKLSKIEILRLAICYISYLN

>gi|4507363|ref|NP_003180.1| T-cell acute lymphocytic leukemia 1 [Homo sapiens]

GPHTKVVRRIFTNSRERWRQQNVNGAFAELRKLIPTHPPDKKLSKNEILRLAMKYINFLA

>gi|4885619|ref|NP_005412.1| T-cell acute lymphocytic leukemia 2 [Homo sapiens]

NSMMTRKIFTNTRERWRQQNVNSAFAKLRKLIPTHPPDKKLSKNETLRLAMRYINFLV

>gi|34147558|ref|NP_005574.2| lymphoblastic leukemia derived sequence 1 [Homo sapiens]

QKVARRVFTNSRERWRQQNVNGAFAELRKLLPTHPPDRKLSKNEVLRLAMKYIGFLV

>gi|48255909|ref|NP_004600.2| basic helix-loop-helix transcription factor 15 [Homo sapiens]

VVVRQRQAANARERDRTQSVNTAFTALRTLIPTEPVDRKLSKIETLRLASSYIAHLA

>gi|89028323|ref|XP_931209.1| PREDICTED: similar to Basic helix-loop-helix transcription factor scleraxis [Homo sapiens]

GREPRQRHTANARERDRTNSVNTAFTALRTLIPTEPADRKLSKIETLRLASSYISHLG

>gi|89028747|ref|XP_944078.1| PREDICTED: similar to transcription factor 23 [Homo sapiens]

RSGSGRPAAANAARERSRVQTLRHAFLELQRTLPSVPPDTKLSKLDVLLLATTYIAHLT

>gi|4826840|ref|NP_005089.1| musculin (activated B-cell factor-1) [Homo sapiens]

CKQSQRNAANARERARMRVLSKAFSRLKTSLPWVPPDTKLSKLDTLRLASSYIAHLR

>gi|75677394|ref|NP_786951.1| transcription factor 23 [Homo sapiens]

RSEASPENAARERSRVRTLRQAFLALQAALPAVPPDTKLSKLDVLVLAASYIAHLT

>gi|38202237|ref|NP_003197.2| transcription factor 21 [Homo sapiens]

GKQVQRNAANARERARMRVLSKAFSRLKTTLPWVPPDTKLSKLDTLRLASSYIAHLR

>gi|89039288|ref|XP_943702.1| PREDICTED: similar to mesoderm posterior 2 [Homo sapiens]

GPAGGQRQSASEREKLRMRTLARALHELRRFLPPSLAPAGQSLTKIETLRLAIRYIGHLS

>gi|88954911|ref|XP_944954.1| PREDICTED: similar to mesogenin 1 [Homo sapiens]

VRMSVQRRRKASEREKLRMRTLADALHTLRNYLPPVYSQRGQPLTKIQTLKYTIKYIGELT

>gi|14149724|ref|NP_061140.1| mesoderm posterior 1 [Homo sapiens]

RSSRLGSGQRQSASEREKLRMRTLARALHELRRFLPPSVAPAGQSLTKIETLRLAIRYIGHLS

>gi|18765727|ref|NP_002470.2| myogenin [Homo sapiens]

KRKSVSVDRRRAATLREKRRLKKVNEAFEALKRSTLLNPNQRLPKVEILRSAIQYIERLQ

>gi|23111009|ref|NP_002469.2| myogenic differentiation 1 [Homo sapiens]

RKTTNADRRKAATMRERRRLSKVNEAFETLKRCTSSNPNQRLPKVEILRNAIRYIEGLQ

>gi|5031929|ref|NP_005584.1| myogenic factor 5 [Homo sapiens]

RKSTTMDRRKAATMRERRRLKKVNQAFETLKRCTTTNPNQRLPKVEILRNAIRYIESLQ

>gi|4505299|ref|NP_002460.1| myogenic factor 6 (herculin) [Homo sapiens]

KRKSAPTDRRKAATLRERRRLKKINEAFEALKRRTVANPNQRLPKVEILRSAISYIERLQ

>gi|46370082|ref|NP_996920.1| transcription factor 12 isoform a [Homo sapiens]

KIEREKERRMANNARERLRVRDINEAFKELGRMCQLHLKSEKPQTKLLILHQAVAVILSLEQ

>gi|4507399|ref|NP_003190.1| transcription factor 4 isoform b [Homo sapiens]

KAEREKERRMANNARERLRVRDINEAFKELGRMVQLHLKSDKPQTKLLILHQAVAVILSLEQQVR

>gi|33982|emb|CAA36297.1| ITF-1 DNA binding protein [Homo sapiens]

KDLRDRERRMANNARERVRVRDINEAFRELGRMCQMHLKSDKAQTKLLILQQAVQVILGLEQQVRER

>gi|27777636|ref|NP_003191.1| transcription factor 3 [Homo sapiens]

KAEREKERRVANNARERLRVRDINEAFKELGRMCQLHLNSEKPQTKLLILHQAVSVILNLEQQVRER

>gi|88952671|ref|XP_946059.1| PREDICTED: similar to Achaete-scute homolog 3 (bHLH transcriptional regulator Sgn-1) (Mash-3) [Homo sapiens]

YEYPFEPAFIQKRNERERQRVKCVNEGYARLRGHLPGALAEKRLSKVETLRAAIRYIKYLQ

>gi|4885665|ref|NP_005161.1| achaete-scute complex homolog-like 2 [Homo sapiens]

ETGGGAAAVARRNERERNRVKLVNLGFQALRQHVPHGGASKKLSKVETLRSAVEYIRALQ

>gi|20455478|sp|P50553|ASCL1_HUMAN Achaete-scute homolog 1 (HASH1)

SLPQQQPAAVARRNERERNRVKLVNLGFATLREHVPNGAANKKMSKVETLRSAVEYIRALQ

>gi|37931664|gb|AAP69222.1| achaete-scute-like protein 4; HASH4 [Homo sapiens]

PLDSAFEPAFLRKRNERERQRVRCVNEGYARLRDHLPRELADKRLSKVETLRAAIDYIKHLQ

>gi|10190680|ref|NP_065697.1| ASCL3 [Homo sapiens]

CEYSYGPAFTRKRNERERQRVKCVNEGYAQLRHHLPEEYLEKRLSKVETLRAAIKYINYLQ

>gi|30795240|ref|NP_848513.1| aryl hydrocarbon receptor nuclear translocator isoform 2 [Homo sapiens]

DKERLARENHSEIERRRRNKMTAYITELSDMVPTCSALARKPDKLTILRMAVSHMKSL

>gi|30795242|ref|NP_848514.1| aryl hydrocarbon receptor nuclear translocator isoform 3 [Homo sapiens]

DKERFARENHSEIERRRRNKMTAYITELSDMVPTCSALARKPDKLTILRMAVSHMKSL

>gi|17979652|gb|AAL50340.1|AF246961_1 brain-muscle-ARNT-like transcription factor 2b [Homo sapiens]

ISASSGSREAHSQTEKRRRDKMNNLIEELSAMIPQCNPMARKLDKLTVLRMAVQHLRSL

>gi|71852580|ref|NP_001025443.1| aryl hydrocarbon receptor nuclear translocator-like isoform a [Homo sapiens]

GRIKNAREAHSQIEKRRRDKMNSFIDELASLVPTCNAMSRKLDKLTVLRMAVQHMKTL

>gi|40254439|ref|NP_001421.2| endothelial PAS domain protein 1 [Homo sapiens]

TADKEKKRSSSERRKEKSRDAARCRRSKETEVFYELAHELPLPHSVSSHLDKASIMRLAISFLRTHK

>gi|4504385|ref|NP_001521.1| hypoxia-inducible factor 1, alpha subunit isoform 1 [Homo sapiens]

GGANDKKKISSERRKEKSRDAARSRRSKESEVFYELAHQLPLPHNVSSHLDKASVMRLTISYLRVRK

>gi|41281831|ref|NP_775182.1| neuronal PAS domain protein 3 isoform 2 [Homo sapiens]

TKPSFQQDPSRRERLQALRKEKSRDAARSRRGKENFEFYELAKLLPLPAAITSQLDKASIIRLTISYLKMRD

>gi|22027482|ref|NP_002508.2| neuronal PAS domain protein 1 [Homo sapiens]

PGLMVKAPSGPCLQAQRKEKSRNAARSRRGKENLEFFELAKLLPLPGAISSQLDKASIVRLSVTYLRLRR

>gi|21614542|ref|NP_005059.2| single-minded homolog 1 [Homo sapiens]

MKEKSKNAARTRREKENSEFYELAKLLPLPSAITSQLDKASIIRLTTSYLKMRV

>gi|23065538|ref|NP_690008.1| hypoxia-inducible factor-3 alpha isoform c [Homo sapiens]

MALGLQRARSTTELRKEKSRDAARSRRSQETEVLYQLAHTLPFARGVSAHLDKASIMRLTISYLRMHR

>gi|4827004|ref|NP_005060.1| single-minded homolog 2 long isoform [Homo sapiens]

MKEKSKNAAKTRREKENGEFYELAKLLPLPSAITSQLDKASIIRLTTSYLKMRA

>gi|22027471|ref|NP_002509.2| neuronal PAS domain protein 2 [Homo sapiens]

MDEDEKDRAKRASRNKSEKKRRDQFNVLIKELSSMLPGNTRKMDKTTVLEKVIGFLQKHN

>gi|4758010|ref|NP_004889.1| clock [Homo sapiens]

VEEDDKDKAKRVSRNKSEKKRRDQFNVLIKELGSMLPGNARKMDKSTVLQKSIDFLRKHK

>gi|4502003|ref|NP_001612.1| aryl hydrocarbon receptor [Homo sapiens]

KTVKPIPAEGIKSNPSKRHRDRLNTELDRLASLLPFPQDVINKLDKLSVLRLSVSYLRAKS

>gi|17402863|ref|NP_065782.1| arylhydrocarbon receptor repressor [Homo sapiens]

KQRPAVGAEKSNPSKRHRDRLNAELDHLASLLPFPPDIISKLDKLSVLRLSVSYLRVKSFF

>gi|4507447|ref|NP_003214.1| transcription factor AP-4 (activating enhancer binding protein 4) [Homo sapiens]

QRDQERRIRREIANSNERRRMQSINAGFQSLKTLIPHTDGEKLSKAAILQQTAEYIFSLE

>gi|74315995|ref|NP_001028253.1| l-myc-1 proto-oncogene isoform 1 [Homo sapiens]

DTEDVTKRKNHNFLERKRRNDLRSRFLALRDQVPTLASCSKAPKVVILSKALEYLQALV

>gi|71774083|ref|NP_002458.2| myc proto-oncogene protein [Homo sapiens]

DTEENVKRRTHNVLERQRRNELKRSFFALRDQIPELENNEKAPKVVILKKATAYILSVQ

>gi|127602|sp|P12525|MYCL2_HUMAN L-myc-2 protein

DTENWTKKKYHSYLERKRRNDQRSRFLALRDEVPALASCSRVSKVMILVKATEYLHELA

>gi|19923312|ref|NP_005369.2| v-myc myelocytomatosis viral related oncogene, neuroblastoma derived [Homo sapiens]

DSEDSERRRNHNILERQRRNDLRSSFLTLRDHVPELVKNEKAAKVVILKKATEYVHSLQ

>gi|4505069|ref|NP_002348.1| MAX dimerization protein 1 [Homo sapiens]

NNSSSRSTHNEMEKNRRAHLRLCLEKLKGLVPLGPESSRHTTLSLLTKAKLHIKKLE

>gi|5453734|ref|NP_006445.1| MAD4 [Homo sapiens]

KAPNNRSSHNELEKHRRAKLRLYLEQLKQLVPLGPDSTRHTTLSLLKRAKVHIKKLE

>gi|47717116|ref|NP_569157.2| MAX interactor 1 isoform b [Homo sapiens]

SNTSTANRSTHNELEKNRRAHLRLCLERLKVLIPLGPDCTRHTTLGLLNKAKAHIKKLEE

>gi|9945318|ref|NP_064706.1| MAX binding protein [Homo sapiens]

PGGIGTREVHNKLEKNRRAHLKECFETLKRNIPNVDDKKTSNLSVLRTALRYIQSLK

>gi|13775222|ref|NP_112590.1| MAX dimerization protein 3 [Homo sapiens]

AQDSGRSVHNELEKRRRAQLKRCLERLKQQMPLGADCARYTTLSLLRRARMHIQKLED

>gi|21704269|ref|NP_660092.1| MAX protein isoform e [Homo sapiens]

QSAADKRAHHNALERKRRDHIKDSFHSLRDSVPSLQGEKASRAQILDKATEYIQYMR

>gi|22538455|ref|NP_003734.3| nuclear receptor coactivator 1 isoform 1 [Homo sapiens]

PKGSPCDTLASSTEKRRREQENKYLEELAELLSANISDIDSLSVKPDKCKILKKTVDQIQLMK

>gi|32307124|ref|NP_006525.2| nuclear receptor coactivator 3 isoform b [Homo sapiens]

PCDTPGQGLTCSGEKRRREQESKYIEELAELISANLSDIDNFNVKPDKCAILKETVRQIRQIK

>gi|5729858|ref|NP_006531.1| nuclear receptor coactivator 2 [Homo sapiens]

ECPDQLGPSPKRNTEKRNREQENKYIEELAELIFANFNDIDNFNFKPDKC

>gi|4557755|ref|NP_000239.1| microphthalmia-associated transcription factor isoform 4 [Homo sapiens]

KERQKKDNHNLIERRRRFNINDRIKELGTLIPKSNDPDMRWNKGTILKASVDYIRKLQ

>gi|41393482|gb|AAS02009.1| unknown [Homo sapiens]

KERQKKDNHNLIERRRRYNINYRIKELGTLIPKSNDPDMRWNKGTILKASVEYIKWLQ

>gi|8659574|ref|NP_006512.2| transcription factor binding to IGHM enhancer 3 [Homo sapiens]

KERQKKDNHNLIERRRRFNINDRIKELGTLIPKSSDPEMRWNKGTILKASVDYIRKLQ

>gi|24307933|ref|NP_009093.1| transcription factor EB [Homo sapiens]

KERQKKDNHNLIERRRRFNINDRIKELGMLIPKANDLDVRWNKGTILKASVDYIRRMQ

>gi|64762402|ref|NP_001018068.1| transcription factor EC isoform b [Homo sapiens]

KERQKKDNHNLIERRRRYNINYRIKELGTLIPKSNDPDMRWNKGTILKASVEYIKWLQ

>gi|8923419|ref|NP_060296.1| hypothetical protein LOC54937 [Homo sapiens]

EKNKKISLLHSSKENLRRERIKYCCEQLRTLLPYVKGRKNDAASVLEATVDYVKYIREKI

>gi|22547195|ref|NP_004167.3| sterol regulatory element binding transcription factor 1 isoform b [Homo sapiens]

QSRGEKRTAHNAIEKRYRSSINDKIIELKDLVVGTEAKLNKSAVLRKAIDYIRFLQ

>gi|27477113|ref|NP_004590.2| sterol regulatory element-binding transcription factor 2 [Homo sapiens]

PPKEGERRTTHNIIEKRYRSSINDKIIELKDLVMGTDAKMHKSGVLRKAIDYIKYLQ

>gi|38201614|ref|NP_937848.1| transcription factor-like protein 4 isoform alpha [Homo sapiens]

KESYKDRRRRAHTQAEQKRRDAIKRGYDDLQTIVPTCQQQDFSIGSQKLSKAIVLQKTIDYIQFLH

>gi|4507847|ref|NP_003358.1| upstream stimulatory factor 2 isoform 1 [Homo sapiens]

RTPRDERRRAQHNEVERRRRDKINNWIVQLSKIIPDCNADNSKTGASKGGILSKACDYIRELR

>gi|46877102|ref|NP_996888.1| upstream stimulatory factor 1 isoform 2 [Homo sapiens]

RTTRDEKRRAQHNEVERRRRDKINNWIVQLSKIIPDCSMESTKSGQSKGGILSKACDYIQELR

>gi|46877105|ref|NP_997174.1| upstream stimulatory factor 2 isoform 2 [Homo sapiens]

RTPRDERRRAQHNEVERRRRDKINNWIVQLSKIIPDCNADNSKTGASKGGILSKACDYIRELR

>gi|14602433|ref|NP_116569.1| Williams Beuren syndrome chromosome region 14 isoform alpha [Homo sapiens]

PDSNKTENRRITHISAEQKRRFNIKLGFDTLHGLVSTLSAQPSLKVSKATTLQKTAEYILMLQ

>gi|40353216|ref|NP_055753.2| mondoA [Homo sapiens]

PKNVAALKNRQMKHISAEQKRRFNIKMCFDMLNSLISNNSKLTSHAITLQKTVEYITKLQ

>gi|57242784|ref|NP_001008541.1| MAX interactor 1 isoform c [Homo sapiens]

RAQKHSSGSSNTSTANRRAHLRLCLERLKVLIPLGPDCTRHTTLGLLNKAKAHIKKLE

>gi|38505159|ref|NP_006593.2| transcription factor-like 5 protein [Homo sapiens]

GRSQRRERHNRMERDRRRRIRICCDELNLLVPFCNAETDKATTLQWTTAFLKYIQERH

>gi|89038202|ref|XP_937820.1| PREDICTED: MAX dimerization protein 5 isoform 4 [Homo sapiens]

AEAFAYYRRTHTANERRRRGEMRDLFEKLKITLGLLHSSKVSKSLILTRAFSEI

>gi|8923419|ref|NP_060296.1| hypothetical protein LOC54937 [Homo sapiens]

EKNKKISLLHSSKENLRRERIKYCCEQLRTLLPYVKGRKNDAASVLEATVDYVKYIREKI

>[ref|NP_076870.1|](http://www.ncbi.nlm.nih.gov/entrez/query.fcgi?cmd=Retrieve&db=Protein&list_uids=31415878&dopt=GenPept) early B-cell factor [Homo sapiens]

ALNEPTIDYGFQRLQKVIPRHPGDPERLPKEVILKRAADLVEALY

>[ref|NP_001005463.1|](http://www.ncbi.nlm.nih.gov/entrez/query.fcgi?cmd=Retrieve&db=Protein&list_uids=53828926&dopt=GenPept) early B-cell factor 3 [Homo sapiens]

ALNEPTIDYGFQRLQKVIPRHPGDPERLPKEVLLKRAADLVEALY

>[ref|XP_944784.1|](http://www.ncbi.nlm.nih.gov/entrez/query.fcgi?cmd=Retrieve&db=Protein&list_uids=89028543&dopt=GenPept) PREDICTED: similar to Transcription factor COE2 (Early B-cell

ALNEPTIDYGFQRLQKVIPRHPGDPERLAKEMLLKRAADLVEALY

>[ref|XP_943975.1|](http://www.ncbi.nlm.nih.gov/entrez/query.fcgi?cmd=Retrieve&db=Protein&list_uids=89057992&dopt=GenPept) PREDICTED: similar to Transcription factor COE4 (Early B-cell factor 4) (EBF-4) (Olf-1/EBF-like 4) (OE-4) (O/E-4) [Homo sapiens]

ALNEPTIDYGFQRLQKVIPRHPGDPERLPKEVLLKRAADLAEALY

***Branchiostoma floridae***

>Bf1 gi|45752204|emb|CAE45569.1| putative transcription factor Coe [Branchiostoma floridae]

ALNEPTIDYGFQRLAKLVPRHPGDPERLPKEIILKRAADLAEAIY

>Bf2 gnl|ti|666267682 ATGN321946.g1 gi|66467132|gb|BW858916.1| BW858916 BW858916 Amphioxus Branchiostoma floridae unpublished cDNA library, neurula whole animal Branchiostoma floridae cDNA clone bfne057e09 5', mRNA sequence jgi|Brafl1|241095|e_gw.389.26.1

YKGGSDQRRAIHISAEQKRRFNIKVGFDTLHTLIPTLSSQGNVKISKAAMLQKTVDYTKKLQ

>Bf3 ATUP328173.y1 jgi|Brafl1|61958|fgenesh2_pm.scaffold_448000004

MKGEECPTMKAIERRRRDKINNWITKLSKIVPDCAQDHTKQGQSKGGILAKTCDYIHELR

>Bf4 ATWX107082.b1 gi|66494004|gb|BW879327.1|BW879327 BW879327 Amphioxus Branchiostoma floridae unpublished cDNA library, neurula whole animal Branchiostoma floridae cDNA clone bfne090m20 5', mRNA sequence jgi|Brafl1|96140|fgenesh2_pg.scaffold_279000045

TDSRKNVSRQNHSEIEKRRRDKMNSYIMELSAMIPMCNAMSRKLDKLTVLRMAVQHMKTLR

>Bf5 ATUP293107.g1 gi|66512072|gb|BW894157.1|BW894157 BW894157 Amphioxus Branchiostoma floridae unpublished cDNA library, neurula whole animal Branchiostoma floridae cDNA clone bfne124n01 5', mRNA sequence jgi|Brafl1|124387|estExt_fgenesh2_pg.C_1460068

KDENDKERFARENHSEIERRRRNKMTAYIAELSDMVPTCSALARKPDKLTILRMAVSHMKSLR

>Bf6 ATUP923594.y1 gi|66388355|gb|BW785860.1|BW785860 BW785860 Amphioxus Branchiostoma floridae unpublished cDNA library, gastrula whole animal Branchiostoma floridae cDNA clone bfga003f14 3', mRNA sequence jgi|Brafl1|60197|fgenesh2_pm.scaffold_201000003

DSEDSESKRSTHNILERKRRNDLKASFITLRDSVPELKDNERAPKVNILRKATDYIQSLD

>Bf7 AFSA661826.b2 jgi|Brafl1|75995|fgenesh2_pg.scaffold_61000089

DPRKKSRNYIEKKRRDRINTSLTALKSIIPECKSYGKKKLDKAEILEMTCRQQHFK

>Bf8 ASFW83280.g2 gi|66286920|gb|BW700349.1|BW700349 BW700349 Amphioxus Branchiostoma floridae unpublished cDNA library, adult whole animal Branchiostoma floridae cDNA clone bfad002c12 5', mRNA sequence jgi|Brafl1|117231|estExt_fgenesh2_pg.C_20386

PASPVQKVEKRSSHNAIEKRYRSSINDKIIELKNLVVGEEAKMNKAGVLRKAIDHIHHQ

>Bf9 ATWW175546.g1 jgi|Brafl1|143354|gw.349.31.1

GNPIKIARRIFTNSRERWRQQNVNGAFAELRKLVPTHPPEKKLSKNEILRLAMKYIRFLQ

>Bf10 AWYB5231.b1 jgi|Brafl1|247501|e_gw.492.5.1

KDRQKKDNHNMIERRRRFNINDRIKELGTLLPKTADPDMRWNKGTILKASVDYIRRLK

>Bf11 ATUP899552.x1 jgi|Brafl1|212110|e_gw.47.74.1

MSKCVQEKKRREQEKMYIEELAELISSNMENVMTIKADKCAILQEAVTQIKALR

>Bf12 ATGN56207.g1 gi|66451799|gb|BW843583.1|BW843583 BW843583 Amphioxus Branchiostoma floridae unpublished cDNA library, neurula whole animal Branchiostoma floridae cDNA clone bfne020j19 5', mRNA sequence jgi|Brafl1|188887|gw.35.272.1

DVDSDADKRAHHNALERQRRDHIKNSFSSLRDAVPALQGEKQASRAQILNKATDYIQYMR

>Bf13 ATUP889583.x1 gi|66513074|gb|BW894878.1|BW894878 BW894878 Amphioxus Branchiostoma floridae unpublished cDNA library, neurula whole animal Branchiostoma floridae cDNA clone bfne127m24 5', mRNA sequence jgi|Brafl1|91061|fgenesh2_pg.scaffold_205000025

RSQASSRTTHNELEKNRRAHLRNCLERLKAIIPLSPETPRHTTLGLLNKAKNEIRRLE

>Bf14 ATUP604749.x1 jgi|Brafl1|96736|fgenesh2_pg.scaffold_290000014

GKRRSGGYGTREVHNKLEKNRRAHLKECFEVLKRHIPNMEDKKTSNLCILRSALRYIQVNR

>Bf15 ATGN107746.b1 jgi|Brafl1|66197|fgenesh2_pg.scaffold_9000241

TRDKRIVANARERSRVHTISAAFESLRRAVPSYSYNQKLSKLAILRVACSYITALA

>Bf16 ATUP936576.x1 gi|66416162|gb|BW807946.1|BW807946 BW807946 Amphioxus Branchiostoma floridae unpublished cDNA library, larva whole animal Branchiostoma floridae cDNA clone bflv040h05 5', mRNA sequence jgi|Brafl1|128720|estExt_fgenesh2_pg.C_3260041

RREHERRIRREIANSNERRRMQSINAGFQSLKTLIPHSDGEKLSKAAILQQTAEYIFALE

>Bf17 ATUP588973.x1 jgi|Brafl1|113513|estExt_fgenesh2_pm.C_20027

PLKRPRGSANKKERRRTQSINSAFAELRDRIPNVPADTKLSKIKTLRLATSYIAYLM

>Bf18 ATUP615807.x1 jgi|Brafl1|73526|fgenesh2_pg.scaffold_45000032

QQRRTASERERARMKDMNRAYEALRTKLSHRREPGKKLSKIQCLKFAIEYITDLE

>Bf19 APWS104381.b1 gi|66501718|gb|BW886096.1|BW886096 BW886096 Amphioxus Branchiostoma floridae unpublished cDNA library, neurula whole animal Branchiostoma floridae cDNA clone bfne141i04 5', mRNA sequence jgi|Brafl1|121333|estExt_fgenesh2_pg.C_640081

QQAQRQAANLRERRRMQSINDAFDGLRQRIPTLPYEKRLSKVDTLRLAIGYINFLS

>Bf20 ATUP192345.y2 jgi|Brafl1|76470|fgenesh2_pg.scaffold_64000081

RKKQRLAANVRERRRMESINGAFDVLRKRVPTLAYERRISKADTLHLAIGYIRFLT

>Bf21 ATGN27483.g1 jgi|Brafl1|83592|fgenesh2_pg.scaffold_122000082

PVRLGINARERRRMHDLNDALDELRSVIPYAHSPSVRKLSKIATLLLAKNYILMQA

>Bf22 ATGN325586.g1 jgi|Brafl1|98840|fgenesh2_pg.scaffold_330000001

SKPAVPKDPAKTNPSKRHRDRLNAELDRLASMLPFTDDVLSKLDKLSILRLSVSYLRNKN

>Bf23 ATUP910329.x1 jgi|Brafl1|63636|fgenesh2_pg.scaffold_2000065

ERQSRNRSEKKRRDQFNILINELCSMVSSNNRKMDKSSVLQSTIAFLKRNKEITAQSEANEI

>Bf24 AWYB5604.g1 gi|66331678|gb|BW745030.1|BW745030 BW745030 Amphioxus Branchiostoma floridae unpublished cDNA library, egg whole animal Branchiostoma floridae cDNA clone bfeg037n07 5', mRNA sequence jgi|Brafl1|117200|estExt_fgenesh2_pg.C_20310

PDSSSTSSKRKIRSLAEKNRRDKLTSFISQLSTLLPLANTPDKKLDKCDVLRLAVNYLKVQK

>Bf25 ATGN333724.g1 gi|66423897|gb|BW815681.1|BW815681 BW815681 Amphioxus Branchiostoma floridae unpublished cDNA library, larva whole animal Branchiostoma floridae cDNA clone bflv062g21 5', mRNA sequence jgi|Brafl1|208339|e_gw.35.150.1

SYFDRNSEKRKEKSRDAARVRRSKETEIFYDLAHQLPLAHSKSAQLDKASIMRLAISYLKTRM

>Bf26 ATUP415643.y1 ATGN171077.b1 jgi|Brafl1|163191|gw.84.113.1

SVLALRKEKSRDAARNRRGKENYEFYELAKMLPLPAAITSQLDKASIIRLTISYLKMRD

>Bf27 ATGN307547.g1 jgi|Brafl1|192617|gw.39.276.1

FVFRRAEKSRFAAKARRDQEGEEIQALAQQLPFHKETIQHLDKASVLRLATSYLRMKQ

>Bf28 ATGN307547.g1 jgi|Brafl1|265033|estExt_GenewiseH_1.C_790104

MKEKSKNAARTRREKENYEFSLLAKLLPLPSAITSQLDKASIIRLTTSYLKMR

>Bf29 ATUP890808.y1 jgi|Brafl1|233300|e_gw.302.11.1

PQAVARRNERERNRVRLVNNGFATLRNHVPNGRVNKKMSKVETLKSAVDYIQQLQ

>Bf30 ATUP867093.g1

EPYMVARRNARERRRVQQVNDGFLRLRSLVPKAPKGKKLSKVKTLRAAIEYIEQLQ

>Bf31 ATWX21703.b1

YGFEPAFIRKRNERERQRVKCVNEGYARLREHLPSDISDKRLSKVETLRAAIKYIKKLQ

>Bf32 AFPZ426112.b2 jgi|Brafl1|233331|e_gw.302.22.1

QPRKMSRRNERERQRVRMVNMGFANLRNLVPDGRNNKKMSKVETLRSAMEYIRQLK

>Bf33 AFPZ597357.x1 jgi|Brafl1|127073|estExt_fgenesh2_pg.C_2400032

SARERNLRRLESNERERMRMHSLNDAFQGLRDVIPHVNADRKLSKIETLTLAQNYIVALT

>Bf34 ATWX90395.b2 jgi|Brafl1|105675|fgenesh2_pg.scaffold_503000005

DIIAKRREANARERQRVRNLNTGFAKLRRMVPSLPPNRKPSKVDTLHAAMDYIRTLQ

>Bf35 ATWW92958.g1 jgi|Brafl1|129916|estExt_fgenesh2_pg.C_3970015

GVSKQRQQANARERDRTHSVNTAFTTLRTMIPTEPADRKLSKIETLRLATSYIRCVQ

>Bf36 ATGN256834.g1 gi|88866761|gb|ABD57444.1| Mesp [Branchiostoma floridae] jgi|Brafl1|143026|gw.397.9.1

TGLSKQRQAANERERVRMQNLTAALGVLREHIPPPVAPKDKRLSKIETLKLAIGYIDYLR

>Bf37 ATGN344916.g1 gi|66501362|gb|BW885835.1|BW885835 BW885835 Amphioxus Branchiostoma floridae unpublished cDNA library, neurula whole animal Branchiostoma floridae cDNA clone bfne140m12 5', mRNA sequence jgi|Brafl1|180864|gw.766.28.1

PESLLRRLVANSQERDRMHGINDALDRLRRHIPLHLGPRRLSKIKTLRLAMAYIEALT

>Bf38 AWYB3178.g1 jgi|Brafl1|118748|estExt_fgenesh2_pg.C_180167

RRRRATAKYRTAHATRERIRVEAFNVAFAELRKLLPTLPPDKKLSKIEILRLAICYISYLN

>Bf39 ATUP403425.g1 jgi|Brafl1|90723|fgenesh2_pg.scaffold_201000028

EQPPKVRTAANLRERTRMRVLSKAFVRLKTTLPWVPADTKLSKLDTLRLASSYIGHLS

>Bf40 ATGN221283.b1 jgi|Brafl1|83347|fgenesh2_pg.scaffold_120000067

TGSPKHQRQAANIRERRRMLNINSAFEELRHHVPTFPYERRLSKIDTLRLAIAYIALLS

>Bf41 ATWX64684.g1 jgi|Brafl1|94676|fgenesh2_pg.scaffold_256000081

PEDRVKINRRQLRYSSKIRRGMQRLRGILPDEAFDTPHKVTKISTLRGAIMYLKVLQ

>Bf42 ASWX176839.x1 gi|66494704|gb|BW880027.1|BW880027 BW880027 Amphioxus Branchiostoma floridae unpublished cDNA library, neurula whole animal Branchiostoma floridae cDNA clone bfne092l12 5', mRNA sequence jgi|Brafl1|126806|estExt_fgenesh2_pg.C_2300043

KYRPRPNAAVKERERLVVFNQAFAELQDKLPERLQLSQEKRLPKKLILRFAVRYIKYLQ

>Bf43 AFSA715741.b2 gi|8926258|gb|AAF81766.1|AF271788_1 basic helix-loop helix transcription factor AmphiNeurogenin [Branchiostoma floridae] jgi|Brafl1|116938|estExt_fgenesh2_kg.C_7600001

VKKQRRRKANDRERNRMHNLNGALDQLREVLPTFPDDTKLTKIETLRFAHNYIWALS

>Bf44 AFPZ884435.x1 jgi|Brafl1|150646|gw.499.29.1

KFKMRRSKANARERNRMHGLNRALDRLREVLPCYSKNQKLSKIETLRLARNYLFALT

>Bf45 ATGN269748.g1 jgi|Brafl1|90724|fgenesh2_pg.scaffold_201000029

KPKPEQRNAANARERSRMRTMSKAFVTLKTTLPWVPPDTKLSKLDTLRLAASYIAHLR

>Bf46 AFPZ362769.x1 jgi|Brafl1|180696|gw.433.24.1

NILRYRRLAANARERRRMHGLNDAFDRLRQVVPGIGDDRQLSKYETLQMAQSYILALK

>Bf47 AFSA184659.b2 jgi|Brafl1|90721|fgenesh2_pg.scaffold_201000026

KQKPEQRNAANARERSRMRVLSKAFSKLKTTLPWVPPDTKLSKLDTLRLATSYISHMR

>Bf48 ATUP772592.b1 jgi|Brafl1|208776|e_gw.37.139.1

RERDRRAANNARERLRVRDINEAFRELGRMCSLHLNTDKPQTKLTTLHQAVEVITDLE

>Bf49 ATGN352005.g1

RKSVTVDRRKAATMRERRRLRKVNEAFEVLKRRTCTNPNQRLPKVEILRNAITYIESLE

>Bf50 gi|31544202|gb|AAN87801.2| myogenic regulatory factor 1 [Branchiostoma floridae]

KKTVPIDRRKAATMRERRRLVKVNEAFDILKKKSCANPNQRLPKVEILRNAISYIEQL

>Bf51 ATGN218584.g1

KKTVPIDRRKAATMRERRRLVKVNEAFDILKKKSCANPNQRLPKVEILRNAISYIEQLH

>Bf52 gi|31455588|gb|AAN87802.1| myogenic regulatory factor 2 [Branchiostoma floridae]

RKASRHDRRKAATMRERRRLVKVNEAFEVLKKKTHMKPNQKTPKVDILRNAIAYIEQLH

>Bf53 ATGN148610.g1 jgi|Brafl1|219453|e_gw.78.156.1

RPTGSNAARERSRVKTLRSAFLELQKTLPSVPPDTKLSKLDVLVLATTYIAHLM

>Bf54 ATUP813082.y1

RNPKPEQRNSACARDRSRMRTMSKACVTLNTTFPLVPPDRTLSRLDTLELAGTYIPQIR

>Bf55 ATGN147980.b1 jgi|Brafl1|75995|fgenesh2_pg.scaffold_61000089

RKKSRNYLDTHKNIEKKRRDRINTSLTALKSIIPECKSYGKKKLDKAEILEMTLSYLDKVH

>Bf56 ATGI197437.b1 jgi|Brafl1|99475|fgenesh2_pg.scaffold_342000049

SSHKIVERRRRHRINTCIAQLSQAIPAAFSKSVNRRRGLSGKLEKAEVLEMAVSYVKHIQ

>Bf57 ATUP614092.y1

QTPTSHKVIEKRRRDRINRCLVELSQSVPRAYARQGKLEKAEILEMTVEYVRYL

>Bf58 ATUP352604.x1 gi|66449231|gb|BW841015.1|BW841015 BW841015 Amphioxus Branchiostoma floridae unpublished cDNA library, neurula whole animal Branchiostoma floridae cDNA clone bfne013h11 5', mRNA sequence jgi|Brafl1|60376|fgenesh2_pm.scaffold_217000008

SFEDLQNQRVLANVRERQRTQSLNEAFSSLRKIIPTLPSDKLSKIQTLKLAARYIDFLY

>Bf59 ATWX18121.g1 gi|66334418|gb|BW747770.1|BW747770 BW747770 Amphioxus Branchiostoma floridae unpublished cDNA library, egg whole animal Branchiostoma floridae cDNA clone bfeg029i16 5', mRNA sequence jgi|Brafl1|256629|e_gw.806.17.1

SEEEVQELRLKVNERERKRMHDLNSALDGLREVMPYAHGPSVRKLSKIATLLLAKNYILMLN

>Bf60 ATUP772105.b1 gi|66448891|gb|BW840675.1|BW840675 BW840675 Amphioxus Branchiostoma floridae unpublished cDNA library, neurula whole animal Branchiostoma floridae cDNA clone bfne012i13 5', mRNA sequence jgi|Brafl1|210465|e_gw.42.243.1

PLSKYRRKSANSRERDRMHQINYAFEALRCVVPKLPPSATQDASHAGKMTKITTLRLAMNYISALQ

>Bf61 ATUP301311.g1 gi|66513012|gb|BW894826.1|BW894826 BW894826 Amphioxus Branchiostoma floridae unpublished cDNA library, neurula whole animal Branchiostoma floridae cDNA clone bfne127k20 5', mRNA sequence jgi|Brafl1|87840|fgenesh2_pg.scaffold_166000081

SKLRELVPTIPTNKKMSRIEILQHVIDYIQDLQ

>Bf62 ATUP605448.y1 jgi|Brafl1|103107|fgenesh2_pg.scaffold_426000048

NMQGTEEQRDLHRIVEKRRRDRINDCLANIRELLPEELVRQKSCGKAEILELTLMHMKHLQ

>Bf63 AFPZ37525.b2 jgi|Brafl1|109346|fgenesh2_pg.scaffold_681000009

NGMFELAEEPTPHKVIEKRRRDRINQRFEELRGLVPTARNSQGVKNDKVDLLHMTIEHLKTLT

>Bf64 AWYB5384.g1 jgi|Brafl1|99475|fgenesh2_pg.scaffold_342000049

KRDQKEISSHKIVERRRRHRINTCIAQLSQAIPAAFSKSVNRRRGLSGKLEKAEVLEMAVSYVKHIQ

>Bf65 gi|89242916|gb|ABD64731.1| AmphiHairyD [Branchiostoma floridae] jgi|Brafl1|127515|estExt_fgenesh2_pg.C_2610044

MESRKSSKPIMEKRRRARINESLTELKTLILEALNKDSSRHSKLEKADILEMTVKHLRNLQ

>Bf66 ATUP358569.y1 gi|66482996|gb|BW868319.1|BW868319 BW868319 Amphioxus Branchiostoma floridae unpublished cDNA library, neurula whole animal Branchiostoma floridae cDNA clone bfne085k09 5', mRNA sequence jgi|Brafl1|271445|estExt_GenewiseH_1.C_4620016

PSQSCQMSSRKKRRGIIEKRRRDRINNCLAELRRLVPTAFEKQGSAKLEKAEILQMTVDYLKMLA

>Bf67 gi|37576215|gb|AAQ93671.1| hairy E protein [Branchiostoma floridae] jgi|Brafl1|129457|estExt_fgenesh2_pg.C_3670032

PSEGRKSSKPIMEKRRRARINDSLNQLKTLILDALKKDSSRHSKLEKADILEMTVKHLRNLQ

>Bf68 gi|37576211|gb|AAQ93669.1| hairy C protein [Branchiostoma floridae] jgi|Brafl1|127513|estExt_fgenesh2_pg.C_2610041

LGETRKSSKPIMEKRRRARINDSLNQLKTLILDALKKDSSRHSKLEKADILEMTVKHLRSLQ

>Bf69 gi|37576207|gb|AAQ93667.1| hairy A protein [Branchiostoma floridae] jgi|Brafl1|267120|estExt_GenewiseH_1.C_2610051

ETQRRKSSKPIMEKRRRARINDSLNQLKTLILDALKKDSSRHSKLEKADILEMTVKHLRNLQ

>Bf70 gi|37576221|gb|AAQ93674.1| hairy H protein [Branchiostoma floridae] jgi|Brafl1|118470|estExt_fgenesh2_pg.C_140163

PHQGRKSSKPLMEKRRRARINSSLNELKNLILGTVKDDINAPHHSKLEKADILEI

>Bf71 gi|37576219|gb|AAQ93673.1| hairy G protein [Branchiostoma floridae] jgi|Brafl1|133083|estExt_fgenesh2_pg.C_9080002

PYQSRKSSKPLMEKRRRARINSSLNELKNLILDTYKNDSTHHSKLEKADILEIAVKHVRSLQ

>Bf72 gi|37576217|gb|AAQ93672.1| hairy F protein [Branchiostoma floridae] jgi|Brafl1|116941|estExt_fgenesh2_kg.C_9080002

SCERRKSSKPIMEKRRRARINESLNQLKTLILDALKKDSSRQNKLEKADILEMAVRYLRDIQ

>Bf73 gi|37576209|gb|AAQ93668.1| hairy B protein [Branchiostoma floridae] jgi|Brafl1|116916|estExt_fgenesh2_kg.C_3520001

PGESRKSSKPIMEKRRRARINDSLNQLKALILADLKKDSSHSKLEKADILEMTVKHLRSLQ

>Bf74 jgi|Brafl1|91235|fgenesh2_pg.scaffold_207000025

TVSSKIMVMEKRRRARINDSLNQLKTLIVDTVKKDSSRNNKLEKASTPRDAPAVKHLRSVLKQ

>Bf75 jgi|Brafl1|101172|fgenesh2_pg.scaffold_377000001

SEEELQELRLKVNHRERKRMHDLNSALDGLREVMPYAHGPSVRKLSKIATLLLAKNYILMLNS

>Bf76 jgi|Brafl1|126674|estExt_fgenesh2_pg.C_2240018

QTKPKTRRRIPTLAQRKAANIRERRRMFNLNDAFDKLRKRVPTFSYEKRLSRIETLRLAIIYIHFMK

>Bf77 jgi|Brafl1|129916|estExt_fgenesh2_pg.C_3970015

KLTGVSKQRQQANARERDRTHSVNTAFTTLRTMIPTEPADRKLSKIETLRLATSYISHLA

>Bf78 jgi|Brafl1|94617|fgenesh2_pg.scaffold_256000022

GRKILPEDRVKINRRQLRYSSEIRSAMQRLRGILPDEAFDTPHKVTKISTLRGAIMYHKVLQ

***Strongylocentrotus purpuratus***

>Sp1 lcl|GLEAN3_09465 unnamed protein product

RKKRRGIIEKRRRDRINNSLTELRRLVPAAFEKQGSAKLEKAEILQMTVDHLKYLH

>Sp2 lcl|GLEAN3_06814 unnamed protein product

KSSKPQMEKRRRARINDSLGQLKALILEATNKDSSRHSKLEKADILEMTVKHLRNIQ

>Sp3 lcl|GLEAN3_06813 unnamed protein product

ISLKPQMEKRRRARINDSLLQLKNLVLDALNKNNPRHSKLEKADILEMTVRYLRSIHRQ

>Sp4 lcl|GLEAN3_21608 unnamed protein product

PRTAKHLTERKRRARINDSLLQLKSMVFPVIKKDISRHPKMEKADILEMTVRYLKDVQ

>Sp5 lcl|GLEAN3_15712 unnamed protein product

RKSSKPLMEKRRRARINDCLTELQTILDALNPENNSTRQNKREKADILEQTVKLVKQLRQ

>Sp6 lcl|GLEAN3_15374 unnamed protein product

KLKQLVPTIPKNRKVTRVEILQHVIDYIQDLQ

>Sp7 lcl|GLEAN3_22554 unnamed protein product

EPHLVQRRNARERRRVQLVNDGFIRLRRKIPTEPRNKKLSKVKTLRSAINYILHLQQ

>Sp8 lcl|GLEAN3_09231 unnamed protein product

RRRATAKYRTAHATRERIRVEAFNVAFTELRKLLPTLPPDKKLSKIEILRLAICYIAYLN

>Sp9 lcl|GLEAN3_24918 unnamed protein product

KFKVRRVKANTRERNRMHGLNDALDLLRKVVPCYSSTQKLSKIETLRLAKNYIHALA

>Sp10 lcl|GLEAN3_14401 unnamed protein product

RVVTAGQRTAANVRERRRMFGLNDAFDNLRKEVPKFKHEKRLSRIETLRLAILYIEFLA

>Sp11 lcl|GLEAN3_07147 unnamed protein product

RRHRRLKANDRERNRMHNLNYALDGLREVLPNFPDDTKLTKIETLRFAHNYIWALS

>Sp12 lcl|GLEAN3_27623 unnamed protein product = lcl|GLEAN3_19444 unnamed protein product

RNSRRLESNERERMRMHTLNDAFQNLRNIIPHVRAERKLSKIETLTLAKNYILALS

>Sp13 lcl|GLEAN3_17287 unnamed protein product

PVDEAVMGNRKERRRTQSINTAFADLRECIPNVPADTKLSKIKTLRLATSYIAYLT

>Sp14 lcl|GLEAN3_17983 unnamed protein product

LSRSRRIVANARERNRVHTISSAFEGLRRAVPSYSHNQKLSKLAILRIACSYILALA

>Sp15 lcl|GLEAN3_16650 unnamed protein product

PRQRGAANARERDRTHSVNSAFVQLRDLIPTEPRDRKLSKIETLRLATSYINHLG

>Sp16 lcl|GLEAN3_02677 unnamed protein product

VRQRQAANLRERKRMSSINDAFEGLREHIPTLPYEKRLSKVDTLRLAIGYINFLA

>Sp17 lcl|GLEAN3_28093 unnamed protein product

KVVRRIFTNSRERWRQQNVNSAFSELRKLLPCHPVDKKLSKNEILRLTIRYINFLM

>Sp18 lcl|GLEAN3_26601 unnamed protein product

KQIRLNINARERRRMHDLNDALDDLRGVIPYAHSPSVRKLSKIATLLLAKNYILMQA

>Sp19 lcl|GLEAN3_02627 unnamed protein product

DQQDLRLKINSRERKRMHDLNKALDGLREVMPYAHGPSVRKLSKMSTLLLAKNYILMLR

>Sp20 lcl|GLEAN3_03681 unnamed protein product

DTDKRRLLTNHRERKRMRSLNDAMDRLRNVVPHYPSKRRLSKMETLLLAQSYIMALS

>Sp21 lcl|GLEAN3_00990 unnamed protein product

TEVKRESVNMRERDRMHQLRDAFELLRRVLPRYPVRQKCSKVDTLLLAQDYILTLQ

>Sp22 lcl|GLEAN3_12008 unnamed protein product

KPVQRNAANARERTRMRVLSKAFSKLKTSLPWVPPDTKLSKLDTLRLASSYISHLK

>Sp23 lcl|GLEAN3_03179 unnamed protein product

EKRIRREIANSNERRRMQSINAGFQSLKLLLPHNEGEKLSKAAILQQTADYICRMEQ

>Sp24 lcl|GLEAN3_16445 unnamed protein product

GIDDPNDPVNAARERSRVKTLRDAFLELQRSLPSVPPDTKLSKLDVLVLATTYISHLM

>Sp25 lcl|GLEAN3_21119 unnamed protein product

KNVAVDKRKAATLRERRRLRKVNEAFEALKRHTCANPNQRLPKVEILRNAIEYIEKLE

>Sp26 lcl|GLEAN3_22163 unnamed protein product

LTQAEKRAHHNALERKRRDHIKDSFSMLRDSVPNLQGEKASRAQILNKATDYIQFMR

>Sp27 lcl|GLEAN3_02448 unnamed protein product

RELLQRKTASDRERSRMRDMNNAFETLRIKLAHRKQPGKKMSKIQALRFAIEYINDLE

>Sp28 lcl|GLEAN3_06232 unnamed protein product

KKSSLFDKRRAATQRERRRLCKVNSAFEILKQRTCSNPEQRMPKVTILRNAIQYIERLQ

>Sp29 lcl|GLEAN3_16343 unnamed protein product

VVKEKERRHANNARERIRVRDINEAFKELGRMCQLHLKQDKAQTKLTILHSAVSVITTLEHQ

>Sp30 lcl|GLEAN3_08175 unnamed protein product

HAKERQKKDNHNMIERRRRFNINDRIKELGTLIPKHLDPDQRQNKGTILKSSVDYIRKMQK

>Sp31 lcl|GLEAN3_28148 unnamed protein product

LNQPAPAAVARRNERERNRVKLVNHGFANLRQQLPNGANNKKMSKVETLRSAVSYIRQLQL

>Sp32lcl|GLEAN3_00129 unnamed protein product

DKGHFARENHSEIERRRRNKMTAYITELSDMVPSCSALARKPDKLTILRMAVSHMKSL

>Sp33 lcl|GLEAN3_27935 unnamed protein product

DDSKKLSKQNHSEIEKRRREKMNTYIQELSAMVPTCSSMSSKLDKLTILRMAVQHMKTL

>Sp34 lcl|GLEAN3_01262 unnamed protein product

KSKNSEKRKEKSRNAARSRRGKETEIFYELAHTLPLAHNACAQLDKAGIMRLILSYLKVSK

>Sp35 lcl|GLEAN3_14249 unnamed protein product

RKERSRDAARSRRGKENYEFYELAKLLPLPAAITSQLDKASIIRLTIGYLHMRHFC

>Sp36 lcl|GLEAN3_13962 unnamed protein product

MKEKSKNAARTRREKENAEFFELAKLLPLPSAITTQLDKASIIRLTTSYLKMRA

>Sp37 lcl|GLEAN3_17407 unnamed protein product

EDSKDTTKRKSRNASEKKRRDQFNVLIQELCSMVSTKTRKLDKSAVLRATIHFLKAHN

>Sp38 lcl|GLEAN3_05022 unnamed protein product

NTIPANGTVRTNPSKRHRDRLNTELDRLASLLPFDSDTVTKLDKLSILRLSVSYLRNKS

>Sp39 lcl|GLEAN3_03166 unnamed protein product

DSEDCDRRANHNVLERRRREDLRTSFFKLRDQVPELASQERAAKIVILKKATDYVHHLH

>Sp40 lcl|GLEAN3_06583 unnamed protein product

NSNSSRSTHNELEKNRRAHLRTCLERLKEMVPLDGDMPRHTTLGLLTNAKDFIVDLEE

>Sp41 lcl|GLEAN3_26205 unnamed protein product

DKRAGIREVHNKLEKNRRAHLKDCFENLRNTVPNMEDKKAKTSNLSILRGALRFIQVLTR

>Sp42 lcl|GLEAN3_07253 unnamed protein product

PEKRKSHNAIEQRYRKSINGRIEDLKIMLFKENKKVSKSHTLQKCIDHLVGLRKMCQTLKQ

>Sp43 lcl|GLEAN3_05787 unnamed protein product

EAEAYKSYKDRRRNAHTAAEQKRRDAIKKGYEDLQLIVPTCQQPDQVGSQKLSKATVLQRSIDYIQYLI

>Sp44 lcl|GLEAN3_14332 unnamed protein product

VRDERRRATHNEVERRRRDKINNWIVKLSKIIPDCNIDHSKQGQSKGGILSKTCDYIHDLR

>Sp45 lcl|GLEAN3_08845 unnamed protein product

KEEERGGGKLSHISAEQKRRFNIRSGFDTLNSMIPSLAQNSSAKISKANMLVKGVEYTRKLQ

>Sp46 lcl|GLEAN3_04702 unnamed protein product

SLTEPTIDYGFQRLSKLVPRHPGDPDRLPKEIILKRAADLAEALY

>Sp47 >[Scaffold_v2_6447>](http://www.hgsc.bcm.tmc.edu/star/cgi-bin/fastacmd/?submit=Run&db=/data/services/web-blastdb/blastdb/Spurpuratus/versions/Spurpuratus_BAC_plus_WGS_Assembly_Version_2.0,_June_15_2006&id=Scaffold_v2_6447>)Scaffold_v2_6447

YDLEPSFIRRRNERERERVRNVNEGYARLREHLPCDNPEKRMSKVETLRMAIRYIKQLQ

>Sp48 [Scaffoldi2674>](http://www.hgsc.bcm.tmc.edu/star/cgi-bin/fastacmd/?submit=Run&db=/data/services/web-blastdb/blastdb/Spurpuratus/genome/Spur20060316-genome&id=Scaffoldi2674>)Scaffoldi2674 (29290-29135)

NQRVLANVRERQRTQSLNDAFTNLRKIIPTLPSDKLSKIQTLKLASRYIDFL

>Sp49 [Scaffold_v2_76963>](http://www.hgsc.bcm.tmc.edu/star/cgi-bin/fastacmd/?submit=Run&db=/data/services/web-blastdb/blastdb/Spurpuratus/versions/Spurpuratus_BAC_plus_WGS_Assembly_Version_2.0,_June_15_2006&id=Scaffold_v2_76963>)Scaffold_v2_76963

HPEKGSRFPGNKRRCEKRSSYIEELYELITASLSNPESLNMKPDKCALLQETLSQIKRIK

>Sp50 [Contig611590_Contig206081>](http://www.hgsc.bcm.tmc.edu/star/cgi-bin/fastacmd/?submit=Run&db=/data/services/web-blastdb/blastdb/Spurpuratus/extra/Spur20060316-unassigned&id=Contig611590_Contig206081>)Contig611590_Contig206081 8 58708 (45333-45103)

ITKRTEANARERDRVTYLNGGFEQLRRVLPWAYRGGRRVSKVDTLRAAISYIQFLQ

***Drosophila melanogaster***

>gi|62861862|ref|NP_001015077.1| CG17469-PA.3 [Drosophila melanogaster]

KDRQKKDNHNMIERRRRFNINDRIKELGTLLPKGSDAFYEVVRDIRPNKGTILKSSVDYIKCLK

>gi|24666964|ref|NP_730450.1| Helix loop helix protein 106 CG8522-PB, isoform B [Drosophila melanogaster]

KRSAHNAIERRYRTSINDKINELKNLVVGEQAKLNKSAVLRKSIDKIRDLQ

>gi|24639693|ref|NP_726931.1| Usf CG17592-PA, isoform A [Drosophila melanogaster]

RDDKRRATHNEVERRRRDKINSWIFKLKEMLPEASTSPSTSGSTKGGILIKACEYIKSMQ

>gi|24650606|ref|NP_651556.2| bigmax CG3350-PA [Drosophila melanogaster]

YKERRREAHTQAEQKRRDAIKKGYDSLQELVPRCQPNDSSGYKLSKALILQKSIEYIGYLN

>gi|24585633|ref|NP_724326.1| Mlx interactor CG18362-PA, isoform A [Drosophila melanogaster]

FQPRDTQRRAGHIHAEQKRRYNIKNGFDTLHALIPQLQLNPNAKLSKAAMLQKGADHIKQLR

>gi|24661088|ref|NP_523977.2| hairy CG6494-PA, isoform A [Drosophila melanogaster]

RRSNKPIMEKRRRARINNCLNELKTLILDATKKDPARHSKLEKADILEKTVKHLQELQ

>gi|17136808|ref|NP_476923.1| deadpan CG8704-PA [Drosophila melanogaster]

KTNKPIMEKRRRARINHCLNELKSLILEAMKKDPARHTKLEKADILEMTVKHLQSVQ

>gi|17737431|ref|NP_523599.1| similar to Deadpan CG10446-PA [Drosophila melanogaster]

KRTNKPLMEKRRRARINQSLAILKALILESTKTQNAKNGEGQAKHTKLEKADILELTVRHFQRH

>gi|24650229|ref|NP_524509.2| E(spl) region transcript m3 CG8346-PA [Drosophila melanogaster]

KVMKPLLERKRRARINKCLDDLKDLMVECLQQEGEHVTRLEKADILELTVDHMRKLKQ

>gi|17981745|ref|NP_536753.1| E(spl) region transcript m7 CG8361-PA [Drosophila melanogaster]

KVMKPLLERKRRARINKCLDELKDLMAECVAQTGDAKFEKADILEVTVQHLRKLK

>gi|24650222|ref|NP_524504.2| E(spl) region transcript m CG8333-PA [Drosophila melanogaster]

KVMKPMLERKRRARINKCLDELKDLMVATLESEGEHVTRLEKADILELTVTHLQKMK

>gi|24650224|ref|NP_524505.2| E(spl) region transcript mbeta CG14548-PA [Drosophila melanogaster]

KVMKPMLERKRRARINKCLDELKDIMVECLTQEGEHITRLEKADILELTVEHMKKLR

>gi|24650220|ref|NP_524503.2| E(spl) region transcript mdelta CG8328-PA [Drosophila melanogaster]

KVTKPLLERKRRARMNLYLDELKDLIVDTMDAQGEQVSKLEKADILELTVNYLKAQQQQ

>gi|17738211|ref|NP_524511.1| E(spl) region transcript m5 CG6096-PA [Drosophila melanogaster]

KVKKPLLERQRRARMNKCLDTLKTLVAEFQGDDAILRMDKAEMLEAALVFMRKQV

>gi|17738215|ref|NP_524513.1| Enhancer of split CG8365-PA [Drosophila melanogaster]

KVKKPMLERQRRARMNKCLDNLKTLVAELRGDDGILRMDKAEMLESAVIFMRQQKT

>gi|17933606|ref|NP_525094.1| HES-related CG5927-PA [Drosophila melanogaster]

EVFKPMMERKRRSRINRCLDFIKDLLQEVSHLDGETMAKMDMGDVLELAVHHLSKKN

>gi|17647503|ref|NP_523657.1| Hairy/E(spl)-related with YRPW motif CG11194-PA [Drosophila melanogaster]

KKRRGVIEKKRRDRINSSLTELKRLVPSAYEKQGSAKLEKAEILQLTVEHLKSLQ

>gi|24645680|ref|NP_524775.1| CG17100-PA [Drosophila melanogaster]

PLSHRIIEKRRRDRMNSCLADLSRLIPPQYQRKGRGRIEKTEIIEMAIRHLKHLQ

>gi|17136514|ref|NP_476748.1| spineless CG6993-PA [Drosophila melanogaster]

PPPKDGVTKSNPSKRHRERLNAELDLLASLLPFEQNILSKLDRLSILRLSVSYLRTKS

>gi|24654763|ref|NP_523872.2| trachealess CG6883-PA [Drosophila melanogaster]

KEKSRDAARSRRGKENYEFYELAKMLPLPAAITSQLDKASIIRLTISYLKLRD

>gi|28571685|ref|NP_524340.2| single-minded CG7771-PA, isoform A [Drosophila melanogaster]

KEKSKNAARTRREKENTEFCELAKLLPLPAAITSQLDKASVIRLTTSYLKMRQ

>gi|24651293|ref|NP_524584.2| similar CG7951-PA [Drosophila melanogaster]

KKEKSRDAARCRRSKETEIFMELSAALPLKTDDVNQLDKASVMRITIAFLKIRE

>gi|24660399|ref|NP_523964.2| Clock CG7391-PA, isoform A [Drosophila melanogaster]

DTKRKSRNLSEKKRRDQFNSLVNDLSALISTSSRKMDKSTVLKSTIAFLKNHN

>gi|24641346|ref|NP_511126.2| Resistance to Juvenile Hormone CG1705-PA [Drosophila melanogaster]

ANGREARNLAEKQRRDKLNASIQELATMVPHAAESSRRLDKTAVLRFATHGLRLQY

>gi|17530943|ref|NP_511160.1| germ cell-expressed bHLH-PAS CG6211-PA [Drosophila melanogaster]

QNGREARNRAEKNRRDKLNGSIQELSTMVPHVAESPRRVDKTAVLRFAAHALRLKH

>gi|24645243|ref|NP_731308.1| tango CG11987-PA [Drosophila melanogaster]

KERFASRENHCEIERRRRNKMTAYITELSDMVPTCSALARKPDKLTILRMAVAHMKALR

>gi|24667005|ref|NP_524168.2| cycle CG8727-PA [Drosophila melanogaster]

ARTSDENRKQNHSEIEKRRRDKMNTYINELSSMIPMCFAMQRKLDKLTVLRMAVQHLRGIRGSGSL

>gi|24583007|ref|NP_523521.2| taiman CG13109-PA [Drosophila melanogaster]

QINKCNNEKRRREAENGYIEQLSEILTLNKRGDMTSTKPDKAAILNQVVRTYREIC

>gi|21357543|ref|NP_649097.1| Max CG9648-PA [Drosophila melanogaster]

QAEKRAHHNALERRRRDHIKESFTNLREAVPTLKGEKASRAQILKKTTECIQTMR

>gi|17136268|ref|NP_476605.1| cropped CG7664-PA [Drosophila melanogaster]

EKRMRREIANSNERRRMQSINAGFQSLRSLLPRHEGEKLSKAAILQQTFQYIVELE

>gi|24639544|ref|NP_726879.1| Mnt CG13316-PA, isoform A [Drosophila melanogaster]

NGAGTREVHNKLEKERRAQLKECYDLLKKVLPMGDEDRKKTSNLTILDTAHKYVNSLS

>gi|24639496|ref|NP_525062.2| diminutive CG10798-PA [Drosophila melanogaster]

DTIEKRNQHNDMERQRRIGLKNLFEALKKQIPTIRDKERAPKVNILREAAKLCIQLT

>gi|24654863|ref|NP_523876.2| extra macrochaetae CG1007-PA [Drosophila melanogaster]

SKLKDLVPFMPKNRKLTKLEIIQHVIDYICDLQ

>gi|17136304|ref|NP_476623.1| lethal of scute CG3839-PA [Drosophila melanogaster]

EQLPSVARRNARERNRVKQVNNGFVNLRQHLPQTVVNSLSNGGRGTSKKLSKVDTLRIAVEYIRGLQ

>gi|17136424|ref|NP_476694.1| asense CG3258-PA [Drosophila melanogaster]

PLPQAVARRNARERNRVKQVNNGFALLREKIPEEVSEAFEAQGAGRGASKKLSKVETLRMAVEYIRSLE

>gi|17136616|ref|NP_476803.1| scute CG3827-PA [Drosophila melanogaster]

DQSQSVQRRNARERNRVKQVNNSFARLRDLTKGGGRGPHKKISKVDTLRIAVEYIRSLQ

>gi|17136654|ref|NP_476824.1| achaete CG3796-PA [Drosophila melanogaster]

FNGPSVIRRNARERNRVKQVNNGFSQLDLSNGRRGIGPGANKKLSKVSTLKMAVEYIRRLQ

>gi|24639385|ref|NP_525055.1| Helix loop helix protein 3B CG2655-PA [Drosophila melanogaster]

TGGVRKVFTNTRERWRQQNVSGAFAELRKLVPTHPPDKKLSKNEILRSAIKYIKLLT

>gi|24639715|ref|NP_476989.2| Helix loop helix protein 4C CG3052-PA [Drosophila melanogaster]

ATLKYRTAHATRERIRVEAFNVSFAELRKLLPTLPPDKKLSKIEILKLAICYIAYLN

>gi|17137454|ref|NP_477302.1| HLH54F CG5005-PA [Drosophila melanogaster]

VQRNAANARERMRMRVLSSAYGRLKTKLPNIPPDTKLSKLDTLRLATLYIKQLI

>gi|62473575|ref|NP_001014730.1| CG33557-PA [Drosophila melanogaster]

RRPPRQKINARERYRTFNVNSAYEALRNLIPTEPMNRKLSKIEIIRLASSYITHLS

>gi|24658978|ref|NP_523816.2| twist CG2956-PA, isoform A [Drosophila melanogaster]

FSNQRVMANVRERQRTQSLNDAFKSLQQIIPTLPSDKLSKIQTLKLATRYIDFLC

>gi|24583320|ref|NP_609370.2| Hand CG18144-PA [Drosophila melanogaster]

KRNTANKKERRRTQSINNAFSYLREKIPNVPTDTKLSKIKTLKLAILYINYLM

>gi|24645293|ref|NP_731326.1| salivary gland-expressed bHLH CG12952-PB, isoform B [Drosophila melanogaster]

RRTACDRERTRMRDMNRAFDLLRSKLPISKPNGKKYSKIESLRIAINYINHLQ

>gi|24650311|ref|NP_524516.2| delilah CG5441-PA [Drosophila melanogaster]

KYRRKTANARERTRMREINTAFETLRHEVPEAIKGEDAANTNEKLTKITTLRLAMKYITMLT

>gi|17647995|ref|NP_524124.1| target of Poxn CG7659-PA [Drosophila melanogaster]

KRFRRMKANDRERNRMHNLNDALEKLRVTLPSLPEETKLTKIEILRFAHNYIFALE

>gi|17647759|ref|NP_523592.1| Olig family CG5545-PA [Drosophila melanogaster]

TVRLNINARERRRMHDLNDALDELRSVIPYAHSPSVRKLSKIATLLLAKNYILMQQ

>gi|17647645|ref|NP_523611.1| dimmed CG8667-PA [Drosophila melanogaster]

ENMRRLESNERERMRMHSLNDAFQSLREVIPHVEMERRLSKIETLTLAKNYIINLT

>gi|17136194|ref|NP_477446.1| absent MD neurons and olfactory sensilla CG10393-PA [Drosophila melanogaster]

KRRLAANARERRRMNSLNDAFDKLRDVVPSLGHDRRLSKYETLQMAQAYIGDLV

>gi|24645007|ref|NP_731223.1| atonal CG7508-PA [Drosophila melanogaster]

KRRLAANARERRRMQNLNQAFDRLRQYLPCLGNDRQLSKHETLQMAQTYISALG

>gi|17137526|ref|NP_477344.1| cousin of atonal CG7760-PA [Drosophila melanogaster]

RRRQAANARERKRMNGLNAAFERLREVVPAPSIDQKLSKFETLQMAQSYILALC

>gi|17864454|ref|NP_524820.1| net CG11450-PA [Drosophila melanogaster]

TRERRIEANARERTRVHTISAAYETLRQAVPAYASTQKLSKLSVLRVACSYILTLS

>gi|17137250|ref|NP_477189.1| daughterless CG5102-PA [Drosophila melanogaster]

EKERRQANNARERIRIRDINEALKELGRMCMTHLKSDKPQTKLGILNMAVEVIMTLE

>gi|17737917|ref|NP_524322.1| 48 related 3 CG6913-PA [Drosophila melanogaster]

MAQRRAANIRERRRMFNLNEAFDKLRRKVPTFAYEKRLSRIETLRLAITYIGFMA

>gi|17737999|ref|NP_524376.1| 48 related 2 CG5952-PA [Drosophila melanogaster]

MQRQAANVRERKRIQRINSAFDELRVHVPTFPYEKRLSKIDTLRLAIAYISLLR

>gi|45580806|ref|NP_996177.1| 48 related 1 CG33323-PA [Drosophila melanogaster]

MAQQRQAANLRERRRMQSINEAFEGLRTHIPTLPYEKRLSKVDTLKLAISYITFLS

>gi|17136352|ref|NP_476650.1| nautilus CG10250-PA [Drosophila melanogaster]

TVDRRKAATMRERRRLRKVNEAFEILKRRTSSNPNQRLPKVEILRNAIEYIESLE

>gi|45549261|ref|NP_524813.2| knot CG10197-PA [Drosophila melanogaster]

ALNEPTIDYGFQRLQKLIPRHPGDPEKLQKEIILKRAADLVEALY

***Tribolium castaneum***

>Tc1 ref|XP_967112.1|

HVILELRKEKSRDAARSRRGKENFEFYELAKMLPLPAAITSQLDKASIIRLTISYLKLRD

>Tc2 ref|XP_967427.1|

FMNNEKRKEKSRDAARSRRSKETEVFTDLGNALPISQEQVSQLDKASVMRLAIAYLRVRD

>Tc3 ref|XP_967106.1|

DSKRKSRNLSEKKRRDQFNLLVNELSSMVATGSRKMDKSTVLKSTIAFLKNHN

>Tc4 gb|CH476264.1|_54|geneid_v1.2_predicted_protein_54|462_AA

EPSEKRHLHNNMERQRRIDLRNLFNDLKKLIPDISKKQRAAKVLILRGAAQYCRDLQ

>Tc5 gb|CM000277.1|_14|geneid_v1.2_predicted_protein_14|212_AA

KKKLSSAWPANASLRAHLRNCLEILKEIVPLGPEASRHTTLGLLTKAKRFIKWNR

>Tc6 unknown:g2369.t1 [gb|CH476566.1|CH476566.1](http://www.hgsc.bcm.tmc.edu/star/cgi-bin/fastacmd/?submit=Run&db=/data/services/web-blastdb/blastdb/Tcastaneum/genome/Tcas20051011-genome&id=CH476566.1)

RVATLAQRRAANIRERRRMFNLNEAFDKLRRKVPTFAYEKRLSRIETLRLAITYISFMS

>Tc7 unknown:g1529.t1 [gb|CH476319.1|CH476319.1](http://www.hgsc.bcm.tmc.edu/star/cgi-bin/fastacmd/?submit=Run&db=/data/services/web-blastdb/blastdb/Tcastaneum/genome/Tcas20051011-genome&id=CH476319.1)

DDSRHPQRNAANARERARMRVLSKAFCRLKTTLPWVPADTKLSKLDTLRLATSYIAHLR

>Tc8 4:g138.t1 [gb|CM000283.1|CM000283.1](http://www.hgsc.bcm.tmc.edu/star/cgi-bin/fastacmd/?submit=Run&db=/data/services/web-blastdb/blastdb/Tcastaneum/genome/Tcas20051011-genome&id=CM000283.1)

HKDKPAQVVARRNARERRRVQAVNSAFARLRKVVPLENTRGKRVSKVKTLQQAIEYIQALV

>Tc 9 [gb|CH476312.1|CH476312.1](http://www.hgsc.bcm.tmc.edu/star/cgi-bin/fastacmd/?submit=Run&db=/data/services/web-blastdb/blastdb/Tcastaneum/genome/Tcas20051011-genome&id=CH476312.1)

VKLRRCKANARERNRMHGLNAALDRLRKPITHLSSAPQKLSKIETLRLARNYIVAMS

>Tc10 6:g721.t1 [gb|CM000281.1|CM000281.1](http://www.hgsc.bcm.tmc.edu/star/cgi-bin/fastacmd/?submit=Run&db=/data/services/web-blastdb/blastdb/Tcastaneum/genome/Tcas20051011-genome&id=CM000281.1)

GTPVRQRSQANARERDRTHSVNTAFSTLRTLIPTEPKDRKLSKIETLRLASSYISHLG

>Tc11 gi|74477159|gb|CH476271.1|_9|geneid_v1.2_predicted_protein_9|535_AA

SKLKDLVPFMPKNRKLSKLEVIQYVIDYICDLQ

>Tc12 gb|CM000278.1|_1344|geneid_v1.2_predicted_protein_1344|384_AA

RKKSFHRQNHSEIEKRRRDKMNTYITELSAMIPMCHAMSRKLDKLTVLRMAVQHLKTI

>Tc13 gb|DT781459.1|DT781459 126370323 TH1 Tribolium castaneum cDNA clone 97C21 3', mRNA sequence KDRQKKDNHNMVERRRRFNINDRIKELGTLLPKNNDPYYEIVRDVRPNKGTILKSSVEYIKCLK

>Tc14 ref|XP_974195.1|

KEVKRSAHNAIERKYRTSINDKIVELKNIVVGTEAKLNKSGILKKTIEYIRFLQ

>Tc15 ref|XP_971935.1|

RRSNKPIMEKRRRARINNSLNELKTLILDAMKKDPARHSKLEKADILEMTVKHLQNLQ

>Tc16 ref|XP_972685.1|

RKVMKPMLERKRRARINRCLDELKELMVTALQSEGENVSKLEKADILELTVRHLHK

>Tc17 ref|XP_975187.1|

PRRANKPLMEKRRRARINQSLAALKTLILDSAKADNTKHSKLEKADILELTVRHFQRHR

>Tc18 ref|XP_973515.1|

CERVRKPLMEKKRRARINDSLEALKQILLDSKTTLKESSGKKSGQRTAKLEKADILEMTVRYVQHLR

>Tc19 gb|CH476262.1|_8|geneid_v1.2_predicted_protein_8|480_AA

IFQTHKPIMEKRRRARINHCLNEIKTLILEAMNKDPARHSKLEKADILEMAVKHLQNVQ

>Tc20 gb|CM000284.1|_207|geneid_v1.2_predicted_protein_207|347_AA

MSHRIIEKRRRDRMNNCLADLSRLIPTEYLKKGRGRIEKTEIIEMAIKHMKYLQQE

>Tc21 ref|XP_968161.1|

SRKKRRGVIEKKRRDRINMSLSELKRLVPSAFEKQGSAKLEKAEILQMTVDHLKMLH

>Tc22 ref|XP_975370.1|

YSQAEKRAHHNALERKRRDHIKDSFSSLRDSVPALNGEKASRAQILKKAAEYIVFMR

>Tc23 ref|XP_967737.1|

EKRIRREIANSNERRRMQSINNGFQSLRSLLPHHEGEKLSKAAILQQTAEYIYSLE

>Tc24 ref|XP_974297.1|

VMKKRRLAANARERRRMNSLNDAFDRLRDVVPSLGNDRKLSKFETLQMAQTYIAALH

>Tc25 ref|XP_974456.1|

VRDERRRATHNEVERRRRDKINNWITKLSKIIPDGQSKGGILAKACEYILELR

>Tc26 ref|XP_970422.1|

MRENHCEIERRRRNKMTAYITELSDMVPTCSALARKPDKLTILRMAVAHMKALR

>Tc27 ref|XP_967151.1|

YKERRREAHTQAEQKRRDAIKKGYDTLQELVPTCQQTDVSGYKLSKATVLQKSIDYIQYLQ

>Tc28 ref|XP_966542.1|

PSNSREMRNRAEKMRRDKLNSYIGELATLVPMVARSAKRMDKTSILRLAATHLRIYQ

>Tc29 ref|XP_972310.1|

KRRTTANKKERRRTQSINNAYADLRDCIPNVPPDTKLSKIKTLRLATSYINYLV

>Tc30 ref|XP_970709.1|

LRKRRLAANARERRRMQNLNQAFDRLRTFLPQLGQDRQLSKYETLQMAQTYITALY

>Tc31 ref|XP_971274.1|

ATQKYRTAHATRERIRVEAFNVAFAELRKLLPTLPPDKKLSKIEILRLAICYIAYLN

>Tc32 ref|XP_974243.1|

MKRRRLAANARERRRMNGLNEAFDRLRQVIPSLDADHKLSKFETLQMAQTYIAALR

>Tc33 ref|XP_972025.1|

TVAIDRRKAATLRERRRLRKVNEAFEVLKRRTCNNPGQRLPKVEILRSAIEYIEYLE

>Tc34 ref|XP_973102.1|

SKYRRKTANARERNRMREINQAFETLRRVIPHVQATQVPGSNEKLTKITTLRLAMKYIADLS

>Tc35 ref|XP_967920.1|

KNMTRERRIEANARERTRVHTISAAFDTLRRAIPSYSHNQKLSKLSVLRIACSYIMTLS

>Tc36 ref|XP_973186.1|

SKYRRKNANARERSRMREINQAFEALRRAVPQMGDHLHPSNEKLTKITTLRLAMKYISALS

>Tc37 ref|XP_969845.1|

QIQQRQAANLRERKRMQSINDAFEGLRAHIPTLPYEKRLSKVDTLKLAIGYINFLS

>Tc38 ref|XP_974793.1|

KSVRLNINARERRRMHDLNDALDELRAVIPYAHSPSVRKLSKIATLLLAKNYILMQA

>Tc39 ref|XP_967000.1|

FRAGTREVHNKLEKHRRAHLKECFDVLKKQLPQTQDEKKTSNLSILHSALRCIQSLK

>Tc40 ref|XP_969923.1|

TRGVVKKIFTNSRERWRQQNVSGAFAELRKLVPTHPPDKKLSKNEILRMAIRYIRLLS

>Tc41 ref|XP_967666.1|

KRKKSETKPQAQINKCNNEKRRREQENIYIEELAELISANFADMSSLSVKPDKCAILQETVNQIRSIK

>Tc42 ref|XP_967876.1|

PTPKDGVTKSNPSKRHRERLNAELDTLASLLPFEQNILSKLDRLSILRLSVSYLRTKS

>Tc43 ref|NP_001034496.1|

IQHQRVMANVRERQRTQSLNEAFASLRKSIPTMPSDKLSKIQTLKLAARYIDFLY

>Tc44 ref|NP_001034533.1|

GKTAPQPVAVARRNARERNRVKQVNNGFANLRQHIPNFIAAAFESNSRGGNKKLSKVETLRMAVEYIRSLE

>Tc45 ref|XP_971229.1|

RNLRRLESNERERMRMHSLNDAFEQLREVIPHIKMERKLSKIETLTLAKNYIMALT

>Tc46 ref|XP_970244.1|

KRVRRLKANDRERNRMHMLNEALDRLRCVLPTFPEDTKLTKIETLRFAHSYIFALT

>Tc47 ref|XP_973686.1|

SLNEPTIDYGFQRLQKLIPRHPGDPEKLPKEIILKRAADLAEALY

>Tc48 ref|NP_001034537.1|

PQQQPASVARRNARERNRVKQVNNGFATLRQHIPASVAAAFAPQGPSTGRGASKKLSKVETLRLAVEYIRSLK

>Tc49 ref|XP_971276.1|

GTSPYRVQRHAANIRERKRMLRSAIGPTGSINSAFDELRMHVPTFPYEKRLSKIDTLRLAIAYIALLR

>Tc50 ref|XP_973272.1|

AQREKERRQANNARERIRIRDINEALKELGRMCMAHLKTDKPQTKLGILNMAVEVIMTLE

***Daphnia pulex***

>Dp1 AZWZ643886.g1

KKSSHNAIERRYRNSINDKILELKNLIAGEEAKMSKSAILRKALEYVRYLQ

>Dp2 AZSH477146.b1

RDDRRRVSHNEVERRRRDKINNWILKLGKIMPDSVHNDAGKTGQSKGGILSKACDYITDIR

>Dp3 AZSH459046.b1

LYFRQNHSEIEKRRRDKMNTYITELSRVVPMCITMSHKLDKLTVLRMAVQHLKG

>Dp4 AZSH597319.b1

FLCRENHCEIERRRRNKMTAYITELSDMVPTCSALARKPDKLTILRMAVAHMKALR

>Dp5 AZSH484412.b1

KSYKERRREAHTQAEQKRRDAIKKGYSSLQDLVPTCQQQDPISGYKLSKATVLQRSIDYIQVN

>Dp6 AZSH461973.g1

EHRRVCHINAEQKRRCNIKNGFDTLQSLLPHGKASDVSKAAMLHRGIS

>Dp7 AZSH613178.y1

RATPKYRSAHASRERVRVEAFNSAFADLRKLLPTLPPDKKLSKIEILRLAICYIGYLN

>Dp8 AZWZ573259.g2

GGNMTRKRRRGIIEKRRRDRINHSLSELRRLVPSAFEKQGSAKLEKAEILQLTVDHLKMLH

>Dp9 AZSH557912.g1

KVMKPMLERKRRARINRCLDELKELMSSALASEGENLTKLEKADVLELTVRHLHKLR

>Dp10 AZWZ561292.b1

KVTKPLLERQRRARINRCLDELKELMSAALAAEGENLTKLEKADVLELTVRHLHQL

>Dp11 AZSH201690.x1

TNKPLMEKRRRARINHSLSVLKSLIIANSSNPASQSSRLEKADILELTVMHLRTLE

>Dp12 AZSH96665.y1

VRKPLMEKKRRARINQSLNDLKDLLMETDSVKKESRPTKLEKADVLELTVNYVQKLH

>Dp13 AZWZ426681.g2

RDPQSHRIIEKRRRDRMNNCLADLSRLLPSAYMKKGRGRIEKTEIIEMTIKHMKHLQ

>Dp14 AZWZ185698.b1

ITKPLLERQRRARINRCLDELKKELMSAALAAEGENLTKLEKADVLETDVRXTAQLH

>Dp15 AZSH429417.y1

KVTKPLLERQRRARINRCLDELKDLMAGALAAEHGEAGMAKLEKADVLELTVRHLHKLR

>Dp16 AZSH415006.g1

TIDRKSRNLSEKKRRDQFNILINELCSMVCTGKRKMDKSTILKSAISFIRNHN

>Dp17 AZSH519420.b1

PSPKESSGKSNPSKRHRERLNAELDTLASLLPYEASILSKLDRLSILRLSVSYLRTKS

>Dp18 AZWZ653193.b1

FSILEMRKEKSRDAARSRRGKENYEFYELAKMLPLPPAITSQLDKASIIRLTISFLRLKD

>Dp19 AZWZ550673.b1

MKEKSKNAARSRREKENAEFMELAKMLPLPSAITSQLDKASVIRLTTSYLKMRQ

>Dp20 AZWZ651271.g1

ANRNSEKRKEKSREAARCRRSKESEIFTDMANLLPVPSSLSSQLDKASIMRLTIAFLKAQS

>Dp21 AZSH580468.x1

KFARIRKSEKRKEKSREAARYRRSQEVETFNDLANLLPVSSSLISQLDKASIMRLTIAFLKVQ

>Dp22 AZSH530911.g1

RSSSREMRNRAEKQRRDKLNAYISELYSLVPSAAAAPRKLDKTSTLRLSANFLRIHQ

>Dp23 AZSH596961.g1

KPQAQINKCNNEKRRREQENVYIEELAELISVSIADVNSLSVKPDKCAILQETVNQIRKIR

>Dp24 AZSH88493.x1

QVDKRAHHNALERKRRDHIKDSFSGLRDSVPSLQGEKASRAQILKKAADYIQFMR

>Dp25 AZSH379850.y1

DSCEKRSQHNSMERQRRVDLRNAFEFLRSLIPDLEATDRAAKVVILKKAANFCQGLT

>Dp26 AZSH443152.y1

NRAGTREVHNKLEKNRRAHLRECFEFLRKQLPAIDDKKLSNLGILKSALRHIQVTK

>Dp27 AZWZ644716.b1

RRAQLRTCLEKLKDLVPLGPESARHTTLGLLTRAKHFIK

>Dp28 AZSH460813.b1

MKKRRTAANARERRRMNSLNDAFEKLREVVPSLGSDRKLSKFETLQMAQTYINALH

>Dp29 AZSH458121.b1

VKKRRLAANARERRRMNSLNDAFERLREVVPALGSDRKLSKFETLQMAQTYIGALA

>Dp30 AZWZ605972.g1

QVHQRQAANLRERRRMQSINDAFEGLRTHIPTLPYEKRLSKVDTLRLAIGYIGFLA

>Dp31 AZWZ657337.g1

EKRVRREIANSNERRRMQSINSGFQSLRTLLPQSEGEKLSKAAILQQTTEYIYQLE

>Dp32 AZWZ413457.b1

GKAVRLSINARERRRMHDLNDALDELRSVIPYAHSPSVRKLSKIATLLLAKNYIMMQA

>Dp33 AZSH631254.b1

TRERRMQANARERTRVHTISSAFEALRRAVPSFSHGQRLSKLSILRVASAYIAALG

>Dp34 AZSH607054.y1

SKLKELVPHMPKNRKVSKLEVIQHVIDYICDLQ

>Dp35 AZSH516907.g1

SKLRELVPNMPKNRKVSKVEVINNVIDYICDLQ

>Dp36 AZSH539988.g1

VLPPAPPASVARRNARERNRVKQVNMGFAVLRQHIPTAFCSSKSRKVSKVDTLRCAVEYIRSLQ

>Dp37 AZWZ169743.b1

SLGPDPPASVARRNARERNRVKQVNTGFAVLRQHIPVLKKNKMSKVETLRCAVEYIRNLE

>Dp38 AZSH432681.y1

SLGPDPPASVARRNACECNRIKKVNTGFAVLRQHIIPVLTSSSSKKNQMSKVETLRCAVEYIRNLE

>Dp39 AZSH539244.b1

MKKRRLAANARERRRMNNLNSAFDRLRDVVPALGNDRQLSKYETLQMAQSYITALW

>Dp40 AZSH629628.b1

KRTRRVKANDRERNRMHMLNHALDRLRTVLPTFPEETKLTKIETLRFAHNYIWALS

>Dp41 AZSH316988.y3

PLIVFPRKTFSNSRERWRQQNVSGAFSELRKLVPTYPPDKKLSKNEILRLAIKYIRLLS

>Dp42 AZSH630271.g1

QDVQSQRVLANVRERQRTQSLNEAFSALRKIIPTLPSDKLSKIQTLKLAARYIDFLY

>Dp43 AZWZ71195.b1

VATVAQRRAANIRERRRMFNLNEAFDKLRRKVPTFAYEKRLSRIETLRLAITYISFM

>Dp44 AZWZ645683.g1

SPYRVQRFAANIRERKRMLSINSAFDELRIHVPTFPYEKRLSKIDTLRLAIAYIALLK

>Dp45 AZSH626583.g1

SVNSAFTALRTLIPTEPADRKLSKIETLRLASSYIAHL

>Dp46 AZSH621422.g1

TRFSPTAANARERARMRVLSRAFCRLKTTLPWVPADTKLSKLDTLRLATTYIGHLR

>Dp47 AZSH640273.y1

KKSACDRERTRMRDMNRAFDSLREKLPYIKPPGKKLSKIESLRLAIKYIRHLQ

>Dp48 AZSH504674.g1

SKYRRKTANARERSRMKEINQAFETLRRAIPAAADGNCEKWTKITTLRLAMDYIEALT

>Dp49 AZSH17380.x1

KTGPRRHKANARERQRMHGLNGALDNLRPIVSDSQKLSKIETLRLAKNYIKLLS

>Dp50 AZSH550389.x1

SARERNLRRLESNERERLRMHGLNAAFEELRTVIPHIQVERKLSKIETLTLAKNYIMALT

>Dp51 AZWZ433346.b1

RRNTANKKERRRTMSINNAFSELRDCIPNVPADTKLSKIKTLRLATSYISYLM

>Dp52 AZWZ552243.g1

RFSPTAANAKKRARMRVLNRAFCRLKTTLPWVPADTKLSKLDTLRLATTYIGHLR

>Dp53 AZSH457531.b1

RFSPTAANAKERARMRVLNRTFCRLKTTLPRGPADTKLSKLETLRLAPTYIGNL

>Dp54 AZWZ179286.b1

RIRIRDINEALKELGRMCMTHLKSDKPQTKLGILNMAVEVIMQLE

>Dp55 AZSH519893.b1

VNEAFESLKRRTSNNPGQRLPKVEILRNAIEYIESLE

>Dp56 AZSH109103.x1

ALSEPTIDYGFHRLSKLVPRHPGDPEKLGKEIILKRAADLAEAVY

>Dp57 AZSH452699.g1

TVERRRRFNINDRIKELGTLLPKSNDPDVRQNKGGILKASVDYIRRLR

***Caenorhabditis elegans***

>gi|17570165|ref|NP_510453.1| UNCoordinated family member (unc-3) [Caenorhabditis elegans]

TLAEPGIEYGFQRLQKLLPKYPGDPERLPKDQILKRAAELAEALY

>gi|71996100|ref|NP_495938.3| Helix Loop Helix family member (hlh-3) [Caenorhabditis elegans]

TKQKRNERERKRVDQVNQGFVLLQERVPKAAGNKAKLSKVETLREAARYIQELQ

>gi|17538842|ref|NP_501445.1| Helix Loop Helix family member (hlh-12) [Caenorhabditis elegans] = C28C128

NTDRRSRANERERQRVSEMNGMFDVLLNLLPPSHFKTRLSRVQILREATSYIIRLH

>gi|17568263|ref|NP_508119.1| Helix Loop Helix family member (hlh-19) [Caenorhabditis elegans] = F57C123

LRNHLPKQLRDRKPSKAETLKSAAQYISHLL

>gi|71982463|ref|NP_495131.2| Helix Loop Helix family member (hlh-14) [Caenorhabditis elegans] = C18A38

QVARNERERKRVHQVNHGFDVLRNRLQPKNHTKKWSKADTLREAVKYIQQLQ

>gi|17567391|ref|NP_510837.1| Helix Loop Helix family member (hlh-29) [Caenorhabditis elegans]= F31A32b

KQKVKTKREQIRRSKQDICYTELGNFVHKNRLGNTEQRNKLERVTVLQIILEYICQMP

>gi|17567391|ref|NP_510837.1| Helix Loop Helix family member (hlh-29) [Caenorhabditis elegans] = F31A32a

NQTRKSVSERKRRDEINELLENLKTIVQNPSDSNEKISHETILFRVFERVSGVDLETKF

>gi|17535681|ref|NP_496204.1| REgulator of Fusion family member (ref-1) [Caenorhabditis elegans]= T01E82a

AHNRKTSQEKKRRDEINAKIKELQLLIQNESDNEKMTQGDVLNRAVEVVSRME

>gi|17535681|ref|NP_496204.1| REgulator of Fusion family member (ref-1) [Caenorhabditis elegans]= T01E82b

RKEVKKNREQDRRDRQGEAFDALKNFIIENKLMTSHQVEKMQRLNTLDIIIAYIQNKK

>gi|17531901|ref|NP_495062.1| Helix Loop Helix family member (hlh-26) [Caenorhabditis elegans]= C17C38b

IKKIKSDREQVRRNKRVAAYRELRKFIALNNLSSSEEIDKMENLKVLEIIFEVIRGKS

>gi|17531905|ref|NP_495065.1| Helix Loop Helix family member (hlh-27) [Caenorhabditis elegans]= C17C310b

RRKVKTEREKIRRKKQDDCYAELKFFILNKQMGSYEQRLKLERITILEIIIDYIKHNS

>gi|17531899|ref|NP_495063.1| Helix Loop Helix family member (hlh-25) [Caenorhabditis elegans]= C17C37b

RRKVKTEREKIRRKKQDDCYAELKFFILNKQMGSYEQRLKLERITILEIIIDYIKHNS

>gi|17531905|ref|NP_495065.1| Helix Loop Helix family member (hlh-27) [Caenorhabditis elegans]= C17C310a

NQTPKSGSERKRRNITNELINECKTIVQKSEEEHISQEVVLFRIVKLVTGVNLESNF

>gi|17531899|ref|NP_495063.1| Helix Loop Helix family member (hlh-25) [Caenorhabditis elegans]= C17C37a

NQTPKSASERKRRNITNELINECKTIVQKSEEEHISQEVVLFRIVKLVTGVNLESNF

>gi|17531901|ref|NP_495062.1| Helix Loop Helix family member (hlh-26) [Caenorhabditis elegans]= C17C38a

SHGHRSETEKQRRDDTNDLLNEFKKIVQKSESEKLSKEEVLFRIVKLLSGIQLHHESF

>gi|17567775|ref|NP_510223.1| MaX-Like family member (mxl-3) [Caenorhabditis elegans]= F46G106

SMDDDRRAHHNELERRRRDHIKDHFTILKDAIPLLDGEKSSRALILKRAVEFIHVMQTKL

>gi|17564408|ref|NP_505856.1| MaX-Like family member (mxl-1) [Caenorhabditis elegans]= T19B1011

PFDPKRHAREQHNALERRRRDNIKDMYTSLREVVPDANGERVQASRAVILKKAIESIEKGQSDSAT

>gi|17567895|ref|NP_508725.1| Helix Loop Helix family member (hlh-13) [Caenorhabditis elegans]= F48D63

EEPEERQTASIRERKRMCSINVAFIELRNYIPTFPYEKRLSKIDTLNLAIAYINMLDDVL

>gi|17568545|ref|NP_509367.1| Helix Loop Helix family member (hlh-8) [Caenorhabditis elegans]

NEVENVQQRACANRRERQRTKELNDAFTLLRKLIPSMPSDKMSKIHTLRIATDYISFLDEMQ

>gi|32566485|ref|NP_508410.2| abnormal cell LINeage family member (lin-32) [Caenorhabditis elegans]

MRRSAANERERRRMNTLNVAYDELREVLPEIDSGKKLSKFETLQMAQKYIECLSQILK

>gi|17536291|ref|NP_496070.1| Helix Loop Helix family member (hlh-6) [Caenorhabditis elegans] = T15H93

PYSSSVWKRNERERCRVRNVNDGYERLRKHLPVHFDEKRISKVDTLRLAIRYIKHLDNL

>gi|71988252|ref|NP_502928.2| Helix Loop Helix family member (hlh-17) [Caenorhabditis elegans]= F38C22

EPGVRLSINLRERCRMHDLNEALDDLRAVIPYAHGGSVRKLSKIATLLLAKNHIIMQMNNYKMRIED

>gi|17550984|ref|NP_508440.1| Helix Loop Helix family member (hlh-15) [Caenorhabditis elegans]= C43H68

TPKYRNLHATRERIRVESFNMAFSQLRALLPTLPVEKKLSKIEILRFSIAYISFLDNLLQ

>gi|17553356|ref|NP_497173.1| MaX-Like family member (mxl-2) [Caenorhabditis elegans]= F40G911

DRKKATHLRCERQRREAINSGYSDLKDLIPQTTTSLGCKTTNAAILFRACDFMSQLKTDISDADKQ

>gi|71980646|ref|NP_494798.4| Helix Loop Helix family member (hlh-1) [Caenorhabditis elegans]= HLH1

TKLDRRKAATMRERRRLRKVNEAFEVVKQRTCPNPNQRLPKVEILRSAIDYINNLERMLQQ

>gi|17566888|ref|NP_505401.1| ZK682.4 [Caenorhabditis elegans]

MCTRRYEANARERNRVQQLSKMFDQLRVCLPIEDDAKISKLATLKVASSYIGYLGAILQ

>gi|17551010|ref|NP_509952.1| HAND bHLH transcription factor family member (hnd-1) [Caenorhabditis elegans]= C44C108

QCINSAFEILQQHIPYLKSEERKSLPKIKTLRLAMQYIDHLKKLLGGNEM

>gi|17506431|ref|NP_492372.1| Helix Loop Helix family member (hlh-16) [Caenorhabditis elegans]= DY33

LLRNSINSRERRRMHELNDEFETLRECLPYPNEANSRRMSKANTLLLASNWIKQLANA

>gi|71994970|ref|NP_001023414.1| W02C12.3 [Caenorhabditis elegans]

RDRRKKDIHNMIERRRRYNINDRIKELGQMLPKNTSEDMKLNKGTILKASCDYIRVLQKD

>gi|71989325|ref|NP_001021581.1| Helix Loop Helix family member (hlh-2) [Caenorhabditis elegans]

KDTDRRSQNNARERVRVRDINSAFKELGRMCTQHNQNTERNQTKLGILHNAVSVITQLEEQVRQRNMNPK

>gi|17555978|ref|NP_499472.1| Sterol regulatory element Binding Protein family member (sbp-1) [Caenorhabditis elegans]= Y47D3B7

KTERRTAHNLIEKKYRCSINDRIQQLKVLLCGDEAKLSKSATLRRAIEHIEEVEHENQVLKHHVEQMRKTL

>gi|25150916|ref|NP_506391.2| T01D3.2 [Caenorhabditis elegans]

KSQAQQRRQMENYEFSQLANELPLARAISGQHIDKTTMVRLATAYIKLHN

>gi|71987258|ref|NP_001022626.1| F58A4.7 [Caenorhabditis elegans]

TSLDPDRRMRRQIANCNERRRMQSINAGFLALRALLPRKEGEKLSKAAILQQTADMVHQLL

>gi|17505242|ref|NP_492687.1| Aryl Hydrocarbon receptor Associated protein family member (aha-1) [Caenorhabditis elegans]= C25A111

MEDEMGENKERFARENHSEIERRRRNKMTHYINELAEMVPQCASLGRKPDKLTILRMAVSHMKGIRG

>gi|71993297|ref|NP_001023893.1| HIF (hypoxia inducible factor) homolog family member (hif-1) [Caenorhabditis elegans]= F38A63

RETSRHAARDRRSKESDIFDDLKMCVPIVEEGTVTHLDRIALLRVAATICRLRKTA

>gi|71981698|ref|NP_001021036.1| Aryl Hydrocarbon Receptor related family member (ahr-1) [Caenorhabditis elegans]= C41G75

NPSKRHRERLNGELETVAMLLPYDSSTISRLDKLSVLRLAVSFLQCKAHFQACLHNSQFL

>gi|25150717|ref|NP_506184.2| CKY homolog family member (cky-1) [Caenorhabditis elegans]= C15C82

QRSTRGASKQRRDQINVEIQKLRDLLPLSDLIKDRLFQLQMSSPMPRGID

>gi|17543564|ref|NP_500281.1| abnormal cell LINeage family member (lin-22) [Caenorhabditis elegans]

KPLMEKKRRARINKSLSQLKQILIQDEHKNSIQHSKWEKADILEMAVEYLQQLRSAQ

>gi|17552472|ref|NP_498115.1| C. elegans Neuro D homolog family member (cnd-1) [Caenorhabditis elegans]= C34E107

EISKRKVRRVKANGRERARMHGLNNALDMLREYIPITTQHQKLSKIETLRLARNYIDALQRMLQ

>gi|25152174|ref|NP_497465.2| Y39A3CR.6 [Caenorhabditis elegans]

KRRREEMNEVICEMSTLLPEDIRAHKLRRVDQKPRQLDKCFIIGSTVDIIRNSDYITLIRFV

>gi|17544050|ref|NP_500236.1| NeuroGeNin family member (ngn-1) [Caenorhabditis elegans]= Y69A2AR

VRRDKANARERRRMNSLNDALEHLRGILPALPDEPKMTKIETLRKAQEYIASLS

>gi|17554938|ref|NP_499150.1| T05G5.2 [Caenorhabditis elegans]

MPMVVAKRNARERTRVHTVNQAFLVLKQHLPSLRQFTKRVSKLRILNAAITYIDTLLKLI

***Lottia gigantea***

>Lg1 BGOB136856.b1

MKKCRRSKANDRERSRMHSLNDALESLRKALPTYPEDAKLTKIETLRLAHNYIWALT

>Lg2 BGNG172402.g1

MKKNRRDRANDRERNRMHSLNDALETLREILPNGADGKMTKIETLRTAYNYIWALS

>Lg3 AZZI367245.y1

KLKIRRYKANSRERSRMHGLNHALDDLRRHVPCYSKTQKLSKIETLRLARNYIFSLA

>Lg4 BGNG163680.g1

MTRERRVEANARERSRVHTISAAFDSLRRAVPSYSYNQKLSKLAILRIASSYIMALS

>Lg5 AZZI509617.x1

QLLRLKVNGRERRRMHDLNTALDGLRDVMPYANGPSVRKLSKIATLLLAKNYILMLS

>Lg6 BGNG192327.b1

RNMRRLESNERERMRMHSLNDAFQGLREVIPHVNLERKLSKIETLALAKNYIKALT

>Lg7 AZZI390987.x1

IHELRSKINGRERKRMHDLNLAMEELRNVMPYAKGPSVRKISKIATLSLAKNYISMLT

>Lg8 AZYG501927.b1

DDGKRLLANSQERDRMDKLNSALEALRRVLPNETFLHNRKMSKIKTLTTARNYIASLS

>Lg9 BGOB163993.g1

EEVRLRINGRERERMHDLNTALDSLRQVMPYSQGPSVKKLSKMSTLLLARNYIVMLT

>Lg10 BGNG20796.g1

KYRRKSANARERGRMSDMNLGYEQLQAALPEMGNSSKITKLTLLRLAMNYIKSLP

>Lg11 AZYG221774.g1

RKGTANKKERRRTLSINSAFSNLRGCIPNVPTDTKLSKIKTLRLATSYITYLM

>Lg12 BGNG164580.g1

TKPPRMSANARERDRTHSVNSAFVTLRTMIPTEPADRKLSKIEVLRLSASYIAHLN

>Lg13 AZYG943826.g1

AAQRRAANIRERKRMLNLNEAFDVLRRRVPTFKYERRLSRIETLRLAMGYISFMT

>Lg14 AZYG873783.g1

KVIRLGVNSRERRRMHDLNDALDELRSVIPYAHSPSVRKLSKIATMLLAKNYILMQA

>Lg15 BGNG236002.g1

VVQRRAANVRERRRMFQLNEAFDALRKRLPAFNYEKRLSRIETLRLAMTYISFMQ

>Lg16 BGNG29147.g1

QTQRVLANVRERQRTESLNDAFAQLRKIIPTLPSDKLSKIQTLKLASRYIDFLY

>Lg17 BGOB335872.x1

QQQRHAANMRERKRMQSINDAFEGLRTHIPTLPYEKRLSKVDTLRLAIGYIGFLA

>Lg18 AZZI347104.y1

QYKQRNADNMRERKRMQSSNDAFEGVRTHIPTAYEKRLCKMNTLRLAIGDIGVLA

>Lg19 BGOB329611.x1

QQQRVTANVRERQRTQSLNDAFTQLRQIIPTLPSDKLSKMQTLKLASRYIDFLY

>Lg20 BGOB183821.b1

GERGTANVRERKRMLSINSAFEELRCHVPTFPYEKRLSKIDTLRLAIAYISMLR

>Lg21 AZYH290074.g2

GQARDGATERERSRMHLLNDAFDQLRKVVPKSNLSEHQKLSKIATLRLAIHYISALG

>Lg22 BGNG187018.b1

HTVRRIFTNGRERWRQQNVNGAFAELRKLVPSHPPDKKLSKNEILRMSIKYIKLLN

>Lg23 BGNG74151.b1

KPVQRNAANARERSRMRVLSKAFGKLKTTLPWVPADTKLSKLDTLRLASSYIAHLQ

>Lg24 BGNG228767.b1

GRPVSNAARERSRVKTLRTAFLNLQEILPSVPPDTKLSKLDVLVLATTYISHLM

>Lg25 BGOB222187.y1

KYRRKTANHRERERMQDMNDAFEALRKAVPGPGESDPKQTKVTTLKLALDYIAALR

>Lg26 AZYG780875.g1

QTPVQRGAANLRERKRMFYLNDAFDALKKSLPKKDSKSRLSRIDTLKTAIDYIQGLS

>Lg27 AZZI450765.y1

HPVASSRRNARERNRVKQVNNGFDTLRQRVPNGKKNKKMSKVDTLRAAVEYIKNLQ

>Lg28 AZYH82340.g1

KYRRRSANARERCRINDMNLAYEQLRNVLPQMSPVYSKNKLSKQTILTLAMNYIAALR

>Lg29 BGOB299583.y1

KVGAVSRRNERERNRVKQVNNGFEILREHVPCIKKNKKLSKVETLRRAVEYIKLLQ

>Lg30 BGNG95392.g1

RRATQKYRTAHATRERVRVEAFNVAFSDLRKLLPTLPPDKKLSKIEILRLAICYISYLN

>Lg31 BGNG250147.g1

RPNEYVVKRNRRERERVDNVNQAFVNLRDHLPFRKNDNRMSKISILKSASEYIQSLS

>Lg32 BGNG220053.g1

QKKRRLAANARERKRMESLNVAFDQLRAVVPSIGKDRQLSKYDTLQMAQTYIAALK

>Lg33 BGNG206979.g1

PPHLVAKRNARERRRVQAVNSAFTKLRKHVPYEARHKRLSKVKTLQLAIDYIHALQ

>Lg34 BGNG125345.b1

VYQRRAANLRERKRMKSINDAFENLRTCIPATENADRRLSKVDTLRLAIRYISYLS

>Lg35 AZZI157967.y1

PNFIRRRNERERDRVRCVNEGYERLKEHIPLENNEKRISKVETLRSAIQYIRHLQ

>Lg36 BGNG194467.b1

VTIDRRKAATMRERRRLRRVNEAFEVLKRRTCPNPNQRLPKVEILRNAIDYIESLE

>Lg37 BGNG135406.g1

TVSVARRNERERNRVKLINMTFATLREHIPQLSKGGKSRKLSKVETLRAAIEYIRYLQ

>Lg38 AZZI723251.b1

REKVRRQANNARERRVRVRDINEAFKELGSMVSLHCSSGQPLTKLMVLQSAVTVITSLE

>Lg39 BGNG78125.b2

PRDDRRRATHNEVERRRRDKINNWIVQLSKVVPDCAAEQAKTGASKGGILSKACDYLTELR

>Lg40 AZZI804337.b1

DPDKKLRREIANNNERRRMQSINAGYENLKNLIPHHDGEKLSKVNIPLF

>Lg41 AZYG547467.b1

CNLKHQKHNHSEIEKRRRDKMNTFIVELSALIPMCNAMNRKLDKLTVLRMAVQHMKFL

>Lg42 BGOB129508.g1

FYLCSIRESHCEIERRRRNKMTVYINELCDMVPTCSTLARKPDKLTILRMAVSHMKTL

>Lg43 AZYH282587.g1

FSCRKTRNESEKKRRDQFNLLINELCAMVSMNKKKMDKTTVLKSTIAYLKNHQ

>Lg44 AZYG956173.g1

LEVRKEKSRDAARSRRGKENYEFYELAKMLPLPAAITSQLDKASIIRLSISYLRVRD

>Lg45 BGOB186473.x3

TEKRAHHNALERKRRDHIKGSFHSLRDSVPSLQGEKVSRAQVLKKASDYIQFMR

>Lg46 BGNG262944.b1

NCEGKRTLHNVLERRRRNDLKDSFYVLRDHVPELDSKEKAPKVLILRKASEYIHSLR

>Lg47 BGOB247676.y1

MKEKSKNAARTRREKENAEFYELAKMLPLPSAITSQLDKASIIRLSTSYLKMRT

>Lg48 BGNG244210.b1

ALTEPTIDYGFQRLMKLVPRHPGDPEKLPKQEIILKRAADLAEALY

>Lg49 BGNG91346.b1

SKLKDLVPTVNXGQKLSKKDLLQHVIDYILDLE

>Lg50 BGNG209195.g1

GEKRTAHNAIEKRYRLSINDKIIELKDLVAGKEAKVRESQLNKSAILRKAIDYVRYLQ

>Lg51 BGOB100712.x1

KKDNHNLVERRRRFNINDRIKELGTLLPKSTDPDMRQNKGTILKASVDYIRRLK

>Lg52 AZYG887508.g1

RKGRKLLAEKKRRNRINECLNQIKSLLTEDQDVKTDLGENKMEKVEILESAVEFIKK

>Lg53 AZYG708635.b1

NNKPLMEKRRRARINSCLTQLKSLVLQAMKKDSSQFSKLEKADILELTLKHLRALQ

>Lg54 BGOB321472.x1

QSNKPLMEKRRRARINECLVELKSLVLQALKKDSSRHSRLEKADILEMTVKYLHHVQ

>Lg55 BGOB102251.y1

IIEKRRRDRINSSLTELRRLVPSAFEKQQGSAKLEKAEILQMAVDHLKMLHQKG

>Lg56 BGOB252124.b1

AMSYKDKRREAHTVAEQKRRDNIKKGYDELQNIVPTCHQSDGLGSQKMSKANVLQRCKSYLL

>Lg57 AZZI617112.g1

RRAHLKECIDLLKVQVPYMEEKKTSNLSILRGALKFIQ

>Lg58 BGNG217559.b1

NTEKRKEKSRDAARCRRSKETEVYSDLSQNLPLPQNISNQLDKASIMRLSISYLNVCNIL

>Lg59 BGOB87711.b1

IYSNKCLNEKRRREQENFYIEELAEYILASNLDMSSFSKPDKCAILQETVNTIQQIS

>Lg60 BGNG159018.b1

RGEKSKIAAKARRDNESIEMSNLIEALPLRTEEVKKMDKCSILRLATTYIKVKE

>Lg61 BGNG144492.b01

RLSRNVCEKIRRDKLNFLIQELAKEVPIIASCQKKLDKSSILRLTVAYLKIHF

>Lg62 BGOB128255.y1

PTKDPPKSNPSKRHRLRLNSELDHLASLLPFEQSVISKLDKLSILRLAVSYLRTKT

>Lg63 BGOB260219.x1

KHLFCLQNGFDMLVVLVPSLTQNPKASKADMLRK

***Capitella sp. I***

>Cc1 BGYZ366356.b1

KKTRRLKANDRERTRMHSLNDALDELRVTLPTFPDDAKLTKIETLRFANNYIWALS

>Cc2 BHAZ442349.y2

LKVRRSKANTRERNRMHGLNEALELREYVPCYSKTQKLSKIETLRLARNYISSLA

>Cc3 BGYZ312054.b1

SRYRRKTANARERGRMQDVNAAFEQLRRVIPQFPEDRGRVTKITTLNLALNYIKALR

>Cc4 BGYZ12065.b1

GRRQAANARERRRMNSLNVAFDELRGVIPGLSDDRKLSKYDTLQMAQSYIDALK

>Cc5 BHAZ133509.y1

RKQRSAANQRERRRMVSLNTAFDQLRTRIPTFPHEKKLSRIQTLKYATEYIAVMA

>Cc6 AXOH3323.g1

VRQRSAANQRERKRMRTINDAFDGLRCRIPDAKEDKKVSKVDTLRMAISYINQLT

>Cc7 BGYZ93876.g1

LQHQRVMANVRERQRTQSLNEAFTHLRKIIPTLPSDKLSKIQTLKLATRYIDFLY

>Cc8 BGYZ512747.b1

TRERRVEANARERSRVHTISAAFESLRRAVPSYSYNQRLSKLAILRIAGSYITALS

>Cc9 BGYZ395024.g2

RNLRRLESNERERMRMHSLNDAFQGLREVIPHVKIGRKLSKIETLTLAKNYIKALT

>Cc10 BHAZ171155.g1

GKRKPGNNNKKERRRTHSINSAFASLRGCIPNVPSDTKLSKIKTLRLATSYIAYLM

>Cc11 BGYZ507451.g1

LQNLRLKINSRERKRMHDLNSALDSLREVMPYAHGPSVRKLSKIATLLLARNYILMLS

>Cc12 BGYZ448407.b1

LQDLRLKINGRERKRMHDLNSALDGLREVMPYAHGPSVRKLSKIATLLLARNYILMLQ

>Cc13 BGYZ129531.g1

AEELRLRVNMRERQRMHDINGALDALRQVMPYHNGPSVKKLSKMSTLLLARNYIILLS

>Cc14 BGYZ417121.g2

KHVRLGINARERRRMHDLNDALDELRSVIPYAHSPSVRKLSKIATLLLAKNYILMQA

>Cc15 BHAZ201444.x1

TEEQRLKVNSRERQRMHDMNGALDSLREVMPYAQGPAVKKLSKMNTLLLARNYIVLLG

>Cc16 BHAZ424555.y2

QQVHQRQAANLRERKRMLSINEAFEGLRAHIPTLPYEKRLSKVDTLRLAIGYISFLT

>Cc17 BHAZ415652.x2

QPYRVQRHAANIRERKRMLSINSAFEELRLYVPTFPYEKRLSKIDTLRLAIAYIALLK

>Cc18 BGYZ465306.b2

VITHEQRKAANVRERRRMMSLNEAFDQLRTTVPTFAYEKKLSRIETLRLAITYINFLA

>Cc19 BHAZ345125.b1

KTTPSQRSAANIRERRRMCSLNTSFERLRRRVPAFPHEKRLSRIQTLRLAIMYISFMT

>Cc20 BGYZ486564.b2

VITTEQRRAANVRERNRMFQLNEAFDSLRKRVPTFAYEKKLSRIETLRLAVTYIEFMA

>Cc21 BGYZ96572.g1

VISVEQRRAANIRERKRMFQLNEAFCVLRKRVPTFAYEKKLSRIETLKLAVTYIKFMT

>Cc22 BHAZ42317.x1

VINRVQRKAANVRERRRMVTLNDAFEHLKTRIPRGIKDKKLSRIDTLRTAISYITNMQ

>Cc23 BHAZ48213.x1

VEEIVQQRSIANIRERQRTQSLNEGFAHLRQIIPTLPSDKLSKIQTLKLATRYIDFLY

>Cc24 BGYZ395024.g2

RRATEKYRTAHATRERVRVEAFNVAFSELRKLLPTLPPDKKLSKIEILRLAICYISYLN

>Cc25 BHAZ93054.y1

KLKIHGANERERARMRVLSKAFGRLKTTLPWVPADTKLSKLDTLRLASSYIAHLR

>Cc26 BGYZ431107.g2

PVRKVHRRVFTNSRERWRQQNVNGAFVELRKLVPTHPPDKKLSNEILRLAMRYIRLLN

>Cc27 BHAZ76819.x1

QNPPIIRSSANARERDRTYSVNSAFITLRTLIPTEPADGKLSKIETLRLATSYISHLH

>Cc28 BGYZ501215.g2

CSGLEPAFIRKRNERERDRVRCVNEGYTRLRQHLPFERKDKRVSKVETLRAAIRYIHHLQ

>Cc29 BGYZ246868.b1

CEKPPQVVEKRNARERKRVQTVNGAFVRLRKFIPYENRHKRLSKVKTLRKAIEYIDHLQ

>Cc30 BHAZ159599.x1

REKPPHIVARRNARERRRVQAVNSAFVRLRRHIPNENKKKRLSKVKTLRTAIEYIEGMQ

>Cc31 BGYZ175058.g1

SARKASSVARRNERERNRVKQVNQGFERLREHVPNGSANKKMSKVDTLRSALEYIKYMQ

>Cc32 AXOH28658.g1

SGQLMMNRRVTGHVNEAFRELREVLPGLHPTQPLSRVEILRMAANYIAYLH

>Cc33 BGYZ157930.b1

SRDDRRRATHNEVERRRRDKINSWIVQLSKLIPDCAVEHSKSGQSKGGILAKACDFIQELR

>Cc34 BGYZ271779.b2

MHDQEKRLRREVANSNERRRMQSINAGFQSLRNLIPHGDGEKLSKQAAILQQTVGYIAALE

>Cc35 BHAZ108151.x1

EEGEGKRAQHNVLERKRRNDLKYSFCTLRACVPDISSAERTPKVTILKKASEHVISLKR

>Cc36 BHAZ86551.x1

LKLCLQAEKRAHHNALERKRRDHIKESFHNLRDSIPSVQGEKASRAQILKQATDYIQFMT

>Cc37 BGYZ514809.g1

RRAHLKECFDNLWNQIPHGDLGKTSNLNILRCALRFIQLIK

>Cc38 BHAZ348743.b1

WAKERRKKDNHNSVERRRRFNINDRIKELGTMLPKNVDPDMRHNKGTILKASCDYIRRLR

>Cc39 BGZA101965.b1

NGGRGEKRTAHNAIEKRYRLSINDKIIELKDLVVGPEAKLNKSAVLRKACDYIRFLH

>Cc40 BHAZ83491.y1

SYKDRRREAHTQAEQKRRDAIKKGYEDLQNVVPMCQQPDALGSQKLSKAAILQKAIDYVQYLI

>Cc41 BGYZ281215.b2

DRQQYKDMRRVSHISAEQKRRFNIKTGFDTLHQLVPSLNQNPNAKVSKAAALQKGRSPEIL

>Cc42 BHAZ376692.y1

SLSFKSRKSRNLSEKKRRDQFNMLINELFSMVATSNRKMDKTTTLKTTIAFLRQH

>Cc43 BGYZ320772.g2

QFIQFIRRQNHSEIEKRRRDKMNTYITELSSLVPMCCAMNRKHDKLTVLRLAVQHMKTL

>Cc44 BHAZ143381.x1

PLGLVARESHCEIERRRRNKMTSYINELCDMVPTCSTLARKPDKLTILRMAVSHMKNL

>Cc45 BGYZ126233.b1

RESHCEMNGRRRSKMASYVNELCDMVPACSTLARKPDKLTILRLAVAHMKSL

>Cc46 BGYZ192920.b1

IFILRLSRNESEKLRRDRLNAYIGELAKVVPLVMQSDKKMDKASILRLTVTYLRIHH

>Cc47 BHAZ3226.y1

HFGDTQGKSRDAARSRRGKENYEFYELAKMLPLPGAITSQLDKASIIRLTISFLKLKD

>Cc48 BHAZ392731.y1

GGGVMKEKSKNAARTRREKENSEFFDLAKMLPLPAAITSQLDKASIIRLTTSYLKMRT

>Cc49 BGZA74913.b1

FIFFFRAEKSRLAAKDRRDREVEELANLAQLLPFDPSVTAKLDKGAILRLTIDFFRMKS

>Cc50 BHAZ132123.y1

ALTEPTIDYGFQRLMKLVPRHPGDPERLPKEIILKRAVDLAEACT

>Cc51 BGYZ421748.g2

LKLQDLVPSLPQEKKVSKVQLLQHVIDYILDLE

>Cc52 BHAZ68187.x1

LLVQVIEKRRRDRINQSLGELRRLVPSAFEKQGSAKLEKAEILQMTVDHLKILS

>Cc53 BHAZ168115.y1

FVSQVVEKRRRDRINQSLGELRRLVPSAFEKQGSAKLEKAEILQMTVDHLKMLH

>Cc54 BGYZ457224.b2

QLIKHLVEKRRRGRINQCLEELRCLVLEAMNKQPEQYEKMEKADILEMAVQHMRHVR

>Cc55 BGYZ194481.b1

KKKTVAVDRRKAATMRERRRLRKVNEAFEHLKRRTCPXPNQRLPKVEILRNAIEYIESLE

>Cc56 BGYZ186494.b1

QNSKPLMEKRRRARINASLHQLKVLVLDALKKQSARFSKLEKSDILELTVKHLDPTV

>Cc57 BHAZ178191.g1

RKIRKPIIERRRRERINRCLDQIKSLVLKALNQDKYEKMDKADILEMAVRHLLDNE

>Cc58 BHAZ406017.x2

SNKPLMEKRRRERINKCLDQLKAILMEVTKKESKLEKADILEMTVKYLKNMK

>Cc59 BGYZ390667.g2

GKNKCLNEKRRREQENIYMEELAELISASITDMNNFSNVKPDKCAILQETVNQIRRI

>Cc60 BGYZ508236.g1

KPPMKDPPKSNPSKRHRERLNGELDHLASLLPFEQSVISKLDKLSILRLAVSYLRTKS

>Cc61 BGYZ287377.g2

RRAHLRHCLERLKGIVPVGRESSRHTTLGLLTKAKTFIK

>Cc62 BGYZ414856.b2

EVSGTDTTERERTRMHMLNDAFDDLRKVVPKSNLSEHQKLSKIATLRLAISYISALN

>Cc63 BHAZ404137.y1

RVRVRDINEAFKELGRMCSIHMSTDKPQTKLTILQHAVNIITGLEEQVRGRLLS

>Cc64 BHAZ345849.x1

KKYRQRHAANQRERRRMRTINEAFEGLREKIPAVCHNKKLSKVDTLRMAIRYIQHLA

***Nematostella vectensis***

>Nem1 ATSY9507.y1 jgi|Nemve1|213043|fgenesh1_pg.scaffold_168000006

MKRIRRLRANDRERRRMKSLNRALDSLKKCIPVPQSKRRVTKLEILRIACNYIKSLSDT

>Nem2 AOWB239975.b2 gb|DV082260.1|DV082260 327-384-34_M02_KS Nematostella vectensis normalized cDNA library 327 Nematostella vectensis cDNA clone 327-384-34_M02_KS, mRNA sequence jgi|Nemve1|29762|gw.53.120.1

SKQRQAANARERNRTHSVNAAFDALRLLIPTEPSDRKLSKIETLRLASSYIAHLSTI

>Nem3 ASYG11675.b2 jgi|Nemve1|120156|e_gw.168.51.1

SNRSRRLIANARERSRIHTMSEAFESLRKAVPSYSQDQKLSKLAILRLATSYISALADL

>Nem4 AFIK510207.g2

EARLRRLRANDRERRRIQSINVALEALRKAVPNTRSSGKLTKLDTLLLARDYIKHLNEI

>Nem5 ATSY137355.x1 gb|DV097167.1|DV097167 327-384-9_H14_T7 Nematostella vectensis normalized cDNA library 327 Nematostella vectensis cDNA clone 327-384-9_H14_T7, mRNA sequence gb|DV092171.1|DV092171 327-384-45_K04_M13-FP Nematostella vectensis normalized cDNA library 327 Nematostella vectensis cDNA clone 327-384-45_K04_M13-FP, mRNA sequence jgi|Nemve1|220027|fgenesh1_pg.scaffold_469000003

VRRFRQLRRNARERERQGRLNSAFDVLRGVIPDYLSGKGPERKLTQIETLRLATHYIMALSEM

>Nem6 AUNF9182.g1 gb|DV094734.1|DV094734 327-384-52_I10_M13-FP Nematostella vectensis normalized cDNA library 327 Nematostella vectensis cDNA clone 327-384-52_I10_M13-FP, mRNA sequence gb|DV091433.1|DV091433 327-384-43_H18_M13-FP Nematostella vectensis normalized cDNA library 327 Nematostella vectensis cDNA clone 327-384-43_H18_M13-FP, mRNA sequence jgi|Nemve1|186169|estExt_GenewiseH_1.C_800124

SDTPKKRYTANRKERKRTQTMNTAFEDLRNHIPNVPPDTKLSKIKTLRLAISYIRYLMDI

>Nem7 ATWA316566.b2 jgi|Nemve1|39563|gw.46.215.1

SKQRRLANARERSRVHTLNANIDRLKDILPLFPDEKPSKTETIRIAAVYIAHLTEL

>Nem8 AFIK383795.b2 jgi|Nemve1|39308|gw.5.333.1

RPRNSLKKRLLVNARERERMRVLNNAFQSLRDALPCYIADGHMAKITTLRLAINYIKALTDV

>Nem9 ATWA373493.g1 jgi|Nemve1|233271|fgsh_est.C_scaffold_168000002

KKYAHLRSNRECATARERSRMHSLNDAFDSLRKAIPKTNYNQEEKPSKIATLRLAIHYIAALSDI

>Nem10 ATWA322039.g1 gb|DV093965.1|DV093965 327-384-4_L23_T7 Nematostella vectensis normalized cDNA library 327 Nematostella vectensis cDNA clone 327-384-4_L23_T7, mRNA sequence jgi|Nemve1|19204|gw.168.50.1

RKWALLDRSRRLKASARERKRRHVLNNALELLRKKVPCVDQNPQKLSKIEVLRLAIDYIAMLSCY

>Nem11 AOWB750482.x1

QGNARSTRQLRRNERERGRKARLNAAFQVLKSVVPGNIATGTTDRKLTQVEILRLAKNYILNLTEL

>Nem12 ATWA332866.g1 jgi|Nemve1|97005|e_gw.44.68.1

KSQNQQIVQRHAANLRERKRMQSINEAFEGLRKHIPTLPYEKRLSKVDTLRLAIGYIGFLTEM

>Nem13 ASYG89172.b2 jgi|Nemve1|210540|fgenesh1_pg.scaffold_123000040

TLAFHMEPSAVARRNERERNRVRLVNDGFSSLRQHIPYFPEKKKLSKVETLRCAVAYIKHLQSL

>Nem14 ATSY27043.b3 gb|AAR24458.1| twist family bHLH transcription factor [Nematostella vectensis] jgi|Nemve1|234699|estExt_fgenesh1_pm.C_530001

NDQRAIANVRERQRTQALNEAFNKLRKIIPTLPSDKLSKIQTLRLASRYIDFLCQV

>Nem15 ATWA127733.b2 jgi|Nemve1|200765|fgenesh1_pg.scaffold_23000130

LSKQRQTANARERNRMRSISDALLHLRYHLPQTVVAKDKKLSKIQTLRLAIRYISDLFEI

>Nem16 AFIK341079.x1 jgi|Nemve1|99666|e_gw.53.242.1

RRLTGVSKQRRTANERERNRVQQVNAAFETLRNKIPLRALEKKPSKIDTIRLATRYIQDLTQL

>Nem17 ATWA64238.b2 gb|DV089934.1|DV089934 327-384-31_B24_T7 Nematostella vectensis normalized cDNA library 327 Nematostella vectensis cDNA clone 327-384-31_B24_T7, mRNA sequence gb|DV084838.1|DV084838 327-384-14_C04_KS Nematostella vectensis normalized cDNA library 327 Nematostella vectensis cDNA clone 327-384-14_C04_KS, mRNA sequence jgi|Nemve1|136184|e_gw.370.17.1

KQKEPAVVARRNARERKRVKLVNDGFMRLRKHVPTDPKNKKLSKVKTLRSAIEYIRHLQHL

>Nem18 AOWB695453.y1 jgi|Nemve1|90352|e_gw.23.7.1

PLDDRRHAYMKRLYTNSRERWRQQHVNLAFAELRKLIPTYPPERKLSKNEILRFAMKYIKFLENI

>Nem19 ATSY329016.y4 jgi|Nemve1|200659|fgenesh1_pg.scaffold_23000024

DNSSFHKRLFTNSRERWRQYQVNLAFAELRKLLPTYPPDKKLSKHEILRSTMKYIKFLDGL

>Nem20 ATSY442076.b1 jgi|Nemve1|200658|fgenesh1_pg.scaffold_23000023

SLPTLGFPSRTVTNKKERWRQQSVNLAFAEIRKLLPTYPPDKKLSKVEILRTAVKYIQFLDGV

>Nem21 ATSY293972.y1 jgi|Nemve1|106073|e_gw.80.307.1

KRKARPIKTTTSKKERRRTENINAAFAELRKHIPNVPSDTKLSKIKTLKLAMSYIHHLELQ

>Nem22 ATSY450014.g1 jgi|Nemve1|199398|fgenesh1_pg.scaffold_14000163

KTKLNRLLANEHERRRVAQLNGAYQDLRQLIPGYQCDTKLPKIKILRYAINYIAHLDNI

>Nem23 AFIK349526.y1 gb|DV097107.1|DV097107 327-384-9_E23_T7 Nematostella vectensis normalized cDNA library 327 Nematostella vectensis cDNA clone 327-384-9_E23_T7, mRNA sequence gb|DV091550.1|DV091550 327-384-43_N06_M13-FP Nematostella vectensis normalized cDNA library 327 Nematostella vectensis cDNA clone 327-384-43_N06_M13-FP, mRNA sequence jgi|Nemve1|106438|e_gw.81.71.1

CNEKEHVSVLRRNERERNRVKLVSDGFAALRKHIPTTPVNKKLSKVETLRTAIEYIEHLQRI

>Nem24 ATSY481185.g1 jgi|Nemve1|39186|gw.46.206.1

VSRQRRLANTRERHRVQVLNAYIDRLRHLIPLFPGEKKPSKTETVHLAALYIEHMTEI

>Nem25 ATSY80672.y1 jgi|Nemve1|207162|fgenesh1_pg.scaffold_77000059

MVREQERRYANNARERVRVRDINEAFKELGRMCDMHMKGDKPQTKLVILHQAVSVITSLE

>Nem26 ATWA305668.g2

GVSKLRKAANARERARVKTLNERIVQLKDILPMSDKAVKPTKTDIIWMAAEYIADLREM

>Nem27 ATSY467375.y2 jgi|Nemve1|141592|e_gw.588.6.1

YDYNLEPAFIRKRNERERIRVRHVNEGYARLREHLPEEPSDKRMSKVETLRAAIRYIKHLESL

>Nem28 ATWA224195.g4 gb|DV089051.1|DV089051 327-384-28_L19_T7 Nematostella vectensis normalized cDNA library 327 Nematostella vectensis cDNA clone 327-384-28_L19_T7, mRNA sequence jgi|Nemve1|110858|e_gw.102.51.1

RDEKRRATHNEVERRRRDKINGWITKLAKVVPDCSSDQTKTGQSKGGILAKTVDYITDLRAA

>Nem29 ATWA523682.b1 jgi|Nemve1|148786|e_gw.2616.3.1

RDEKRRATHNEVERRRRDKINGWITKLAKVVPDCSSDQTKTGQSKGGILAKTVDYITDLR

>Nem30 AOWB528579.y1 gb|DV087532.1|DV087532 327-384-22_P07_KS Nematostella vectensis normalized cDNA library 327 Nematostella vectensis cDNA clone 327-384-22_P07_KS, mRNA sequence jgi|Nemve1|112080|e_gw.109.84.1

REKRLRREIANSNERRRMQSINSGFQALRMLIPNTEGEKLSKAAILQQTSEYIFTLEQD

>Nem31 ATSY460349.x2 jgi|Nemve1|201969|fgenesh1_pg.scaffold_31000129

SDETDRSKRLNANLIERRRMQNINSGFATLKKLLPPSERKQTKAAILQQVKTRTTPSLGR

>Nem32 AFIK55762.y1 gb|DV092880.1|DV092880 327-384-47_K08_M13-FP Nematostella vectensis normalized cDNA library 327 Nematostella vectensis cDNA clone 327-384-47_K08_M13-FP, mRNA sequence jgi|Nemve1|38935|gw.6.240.1

SEDSEISRATHNVLERQRREDLKCRFQLLRDSIPELEDNERAPKVAILKKAREFVHQLIGE

>Nem33 ATSY441902.b1 jgi|Nemve1|122473|e_gw.190.114.1

HDCESKRAVHNVLERKRRNDLKTSFHQLRAEVPELEENERSPKVTILRKARDYVEQLKGE

>Nem34 AOWB504078.x1 jgi|Nemve1|82891|e_gw.6.263.1

EPDNFRISHNDLERKRRNELRSRFNSLRKSIPELENNEKTAKIAILRKAYELVPRLQKE

>Nem35 ATSY468811.y1 jgi|Nemve1|178332|estExt_GenewiseH_1.C_60232

DSEYTRATHNVLERKRRNDLKLKFQKLRDAVPELKDNERAPKVSILRKSWEHIVQLKED

>Nem36 ATWA374977.g1 gb|DV088582.1|DV088582 327-384-27_C21_T7 Nematostella vectensis normalized cDNA library 327 Nematostella vectensis cDNA clone 327-384-27_C21_T7, mRNA sequence jgi|Nemve1|80696|e_gw.3.684.1

SGDDADKRAHHNALERKRRDHIKDSFSHLRDSIPSLQGEKASRAQILNKATDYIQFMRRK

>Nem37 ATSY163174.x1 jgi|Nemve1|240935|estExt_fgenesh1_pg.C_370042

GGAGTRETHNKLEKNRRAHLKECFDVLKREVPTLEDKKTSNLNILRSALKHIQILKKQ

>Nem38 AFIK312820.y1 jgi|Nemve1|240998|estExt_fgenesh1_pg.C_380031

RDRQKKDNHNMIERRRRFNINDRIKELGTMLPKQDPDSRQNKGTILKASVDYIRNLK

>Nem39 ATWA2510.g2

KQIRSYSHNQIEKRYRDKISNKMNELKDVVCGPEAKLHKETVLRKAVDKIRHLEKE

>Nem40 ATSY364913.x2 jgi|Nemve1|95178|e_gw.38.6.1

KTDINAKGNRSNHNEIEKRYRNSINNRINELKDLVCGPETKMNKAGILKKALDYIRYLQ

>Nem41 ATWA345539.g1 gb|DV081753.1|DV081753 248-2_O06.abi Nematostella vectensis non-normalized cDNA library 248 Nematostella vectensis cDNA clone 248-2_O06.abi, mRNA sequence jgi|Nemve1|45212|gw.3653.3.1

KQNRRFAHSVAEQKRRDAIKKGYDDLQSIVPTCQHSTSAGSPKLSKAIILQRSIEYVSFMHHQ

>Nem42 ATWA175069.g4 jgi|Nemve1|160110|estExt_gwp.C_120110

SESKRVNRNMNEKKRRDRFNVLIGELASIISPSSRKVDKSTVLKKAIACLKSQKDL

>Nem43 ATWA350295.g1 jgi|Nemve1|214146|fgenesh1_pg.scaffold_193000011

FCFRTTRNESEKRRRDKLNVYITELAAMVPMCASSRKKLDKTTVLQMAVNYMKIHNGK

>Nem44 ATWA488796.b1 jgi|Nemve1|132249|e_gw.307.53.1

NHRQNHSEIEKRRRDKMNTYINELSTMIPMCNAMSRKLDKLTVLRMAVQHMRALRGS

>Nem45 ATSY336052.x1 gb|DV081726.1|DV081726 248-02_G08.abi Nematostella vectensis non-normalized cDNA library 248 Nematostella vectensis cDNA clone 248-02_G08.abi, mRNA sequence jgi|Nemve1|116698|e_gw.140.42.1

VKDKFARENHSEIERRRRNKMNAYINELSDMVPSCTGLARKPDKLTVLRMAVNYMKTLRAP

>Nem46 ATSY217219.y3 jgi|Nemve1|96877|e_gw.44.108.1

ELRKERSRDAARNRRGKENAQFDELARLLPLPAAITSQLDKASIVRLTISYLSMREFA

>Nem47 ASYG25069.b2 gb|AAS48917.1| trancription factor COE [Nematostella vectensis] jgi|Nemve1|164975|estExt_gwp.C_550106

LVEPTIDYGFQRLSKLIPRHPGDPERIPKEIVLKRAADLAETLYQMP

>Nem48 AFIK157599.y1 gb|DV089909.1|DV089909 327-384-31_A17_T7 Nematostella vectensis normalized cDNA library 327 Nematostella vectensis cDNA clone 327-384-31_A17_T7, mRNA sequence jgi|Nemve1|240954|estExt_fgenesh1_pg.C_370087

NVPVGRRISKVEILQYVIDYILDLQTALENQTANRRRRRSNRVNRSP

>Nem49 AFIK848885.b2 gb|DV084293.1|DV084293 327-384-11_O01_KS Nematostella vectensis normalized cDNA library 327 Nematostella vectensis cDNA clone 327-384-11_O01_KS, mRNA sequence jgi|Nemve1|241632|estExt_fgenesh1_pg.C_480076

REYAIREALNHLNSLLPLDNPNRKLSKNMILQTAIEYIRSLQEE

>Nem50 AOWB27765.x1 gb|DV086550.1|DV086550 327-384-1_F03_T7 Nematostella vectensis normalized cDNA library 327 Nematostella vectensis cDNA clone 327-384-1_F03_T7, mRNA sequence jgi|Nemve1|28948|gw.58.85.1

TNARKRRRGLIEKKRRDRINRCLVELRRLVPTALEKEGSSKLEKAEILHLTVEHLKWLRST

>Nem51 AFIK856349.g2 gb|DV086587.1|DV086587 327-384-1_H05_T7 Nematostella vectensis normalized cDNA library 327 Nematostella vectensis cDNA clone 327-384-1_H05_T7, mRNA sequence jgi|Nemve1|47054|gw.201.93.1

RKWSKPVMEKRRRERINRSLEELKRLVLEAQHRDCSRYTKLEKADILEMTVKHLRTLQSQ

>Nem52 AFIK779571.b2 gb|DV091206.1|DV091206 327-384-42_N06_M13-FP Nematostella vectensis normalized cDNA library 327 Nematostella vectensis cDNA clone 327-384-42_N06_M13-FP, mRNA sequence jgi|Nemve1|67328|gw.49.191.1

KSKKPQMEKLRRARINDSLNELKSLVLEAMKKDASRYSKMEKADILEMTVKYLRSAPEK

>Nem53 ATSY291518.x1 gb|DV095121.1|DV095121 327-384-55_N23_M13-FP Nematostella vectensis normalized cDNA library 327 Nematostella vectensis cDNA clone 327-384-55_N23_M13-FP, mRNA sequence gb|DV085197.1|DV085197 327-384-15_M21_KS Nematostella vectensis normalized cDNA library 327 Nematostella vectensis cDNA clone 327-384-15_M21_KS, mRNA sequence jgi|Nemve1|98450|e_gw.49.35.1

KRKAKKPLMEKMRRARINDSLNELKSLVLQSLNKDASRYSKMEKADILEMSVQYLKEIGKQE

>Nem54 AFIK828415.b2 jgi|Nemve1|202232|fgenesh1_pg.scaffold_33000076

GRKSSKPMMEKRRRARINQSLNELKILILEAMKKDTSCYSKLEKADILEMTVKYLRAMK

>Nem55 AFIN75279.g2

SYFQSSKPMMEKRRRARINQSLNELKILILEAMKKDVSEVVRFQKQLLLNVVANIHLF

>Nem56 AOWB216956.g2 jgi|Nemve1|241655|estExt_fgenesh1_pg.C_490007

PKVKRRRIMKPITERLRRERINSSLKELKFLVLSALGQDVSRYSRMEKADILEMTVSYIRKMQ

>Nem57 ATWA406099.b1 gb|DV090381.1|DV090381 327-384-3_A24_T7 Nematostella vectensis normalized cDNA library 327 Nematostella vectensis cDNA clone 327-384-3_A24_T7, mRNA sequence

MEKKRRSRINTSLNELRCLLLDHRQLDELQVALMEKAEILETAVQFLKEHGLGR

>Nem58 ATSY340427.x2 gb|DV096416.1|DV096416 327-384-7_B13_T7 Nematostella vectensis normalized cDNA library 327 Nematostella vectensis cDNA clone 327-384-7_B13_T7, mRNA sequence jgi|Nemve1|242121|estExt_fgenesh1_pg.C_570052

KASKHILERQRRARINQSLAELKNLVLSSLYHDNPEVYMDKSRERLDKAEILDLTVNFLKHHITG

>Nem59 ATSY486556.x2 jgi|Nemve1|246249|estExt_fgenesh1_pg.C_2060037

SECSHKVIEKRRRDRINSCLSELAQLIPSAQNGKQVGIFYSAQPFLSPPTDTVSFLINQLV

>Nem60 jgi|Nemve1|241656|estExt_fgenesh1_pg.C_490008

DRRKAKKPMMEKLRRARINDSLNELKVLVLELLNKDASRYSKMEKADILEMTVGYLRAAQ

>Nem61 jgi|Nemve1|164325|estExt_gwp.C_490010

RRKTKKPLMEKLRRDRINNSLNEMKLLVLESLNKDVSRYSKMEKADILEMTVKFLKEVN

>Nem62 jgi|Nemve1|242118|estExt_fgenesh1_pg.C_570049

KSSKPLLERQRRARINHSLNELKTLVLSSLYQNCPQAEQNCEKMEKAEILELTVNFLKVIQ

>Nem63 jgi|Nemve1|161959|estExt_gwp.C_260085

PRKSRDVQKKCKSRDAARSRRGQQNDEFAELSHQLPLPKSISSQLDRLCIMRLTNSYIKIKR

>Nem64 jgi|Nemve1|204664|fgenesh1_pg.scaffold_53000003

RGPRLTGVSKQRRLANARERNRVHILNTNIQVLRELIPLPPQEKEPTKTEIIWMAAKYIALLS

>Nem65 jgi|Nemve1|159087|estExt_gwp.C_60235

EDKIIRSLHNDIEKQRRDHMKLRFDNLRKATPKLENCEKASKIQILKEAVHLVKILENEGIRLEI

>Nem66 jgi|Nemve1|238559|estExt_fgenesh1_pg.C_60164

SLSAQIEPDLRNQLERERRNDLNTKFQKLKSCLPAMANCKKASKIAILREATNFTTFLRKQEVDLEKEIQTQK

>Nem67 jgi|Nemve1|241780|estExt_fgenesh1_pg.C_510063

KSARRSPSYSRTTHNQLEKNRRAHLRDCLELLKELVPAPPEHQKATTLALLQSAQQYIQVLQ

>Nem68 jgi|Nemve1|26800|gw.101.75.1

EHRRMSHISAEQKRRCNIKMGFDQLASMVPTLASQKSSKVSKATVLQKTVDYTTRLQ

***Hydra magnipapillata***

>Hydra1 1098761154641 gb|CV565854.1|CV565854 taj78f01.x1 gb|CV563795.1|CV563795 taj78f01.y1

TNCSRIKRLRANDRERRRVHLINCAMESLRNVIPGMKEKRKITKLELLRAANRYIWLLDET

>Hydra2 1098609773614 gb|CN633913.1|CN633913 taf54b03.y1 gb|CN633660.1|CN633660 taf54b03.x1

SQIQRRLVANARERSRVHALSNAFNLLRTSIPSYSPEQKLSKLTILRVAINYISALEEI

>Hydra3 1099567141326

HQDPKQSCRRFRANDRERHRMNSLNGALQTLKRCVPLYHGKRRVTKLQILQFACHYISDLS

>Hydra4 1099993107371

DSAYARTRSAANFRERRRMQCLNEAFEGLRTHIPSLPYEKRLSKIDTLKLAISYIQFLA

>Hydra5 1099610406775

MSTQKRMLVNARERERMRVLNKAFESLRDALPCYIADGHMAKITTLRLAINYINALNQVL

>Hydra6 1099414211465

TLLYLRQVNIKERSRSHSVNDAFTHLRTLIPTDPPSRKLSKIETLRLATSYINHLSSLL

>Hydra7 1099610630444

GEPGFIRKRNERERMRVRNVNEGYARLRDHLPLEPNEKRLSKVETLRGAINYIKLLQ

>Hydra8 1099405246074

YRQMKRNERERARQNRINNAFDVLRKMIPNHLTPCKSGQKLTQIETLRLAKYYIASLKELL

>Hydra9 1099067051035

ANMDPAAVARRNERERNRVKQVNDGFDELRQRVPFLPDKKKLSKVEILRCAALYIRDLKDIL

>Hydra10 1099549730234

LWIKRIVANPRERRRVSSINIAIEFLKNVIPGVKEKIKITKLELLTAAKRYIELLEGILQ

>Hydra11 1099288331077

KLKEPAAVARRNARERRRVKMVNDGFLRLRRHVPTDPKNKKLSKVKTLRLAIEYIHHLQHLL

>Hydra12 1099993608462 gb|CN559063.1|CN559063 tad79c10.y1

KVDRSKRLKASARERRRRHVLNDALENLRRKVPVINEKSKHKLSKIEVLRMAIDYIAMLSYY

>Hydra13 1099972736746

LMIRRLVANAKERRRVSSINIAMEALRNIIPGMKEKKKITKLEFEKL

>Hydra14 1098761005233

HLMIRRLVARAEEERRVSSINIAIEFLRNIIPCIQEKVVTELELLSVAKKYIEFLEEIF

>Hydra15 1099742300382 gb|CX634878.1|CX634878 taj44c07.x2 gb|CO538244.1|CO538244 tah81e12.y1

YLRESERRHANNARERVRVRDINEAFKELGRMCSLHLKNENPQTKLTVLHQAVTIINSLES

>Hydra16 1099993216402 gb|DT606289.1|DT606289 ACAG-aaa30e10.g1 gb|DN138479.2|DN138479 ACAE-aaa15i02.g1 gb|CV566185.1|CV566185 taj97c01.x1

SSRDERRRATHNEVERRRRDKINTWIMKLATVVPDCQMDQSKQGTSKGGVLSKALDHIIKLR

>Hydra17 1099166430885 gb|CN563076.1|CN563076 taf82h02.x2 gb|CN560853.1|CN560853 taf82h02.y2 gb|CN550970.1|CN550970 tad55e09.x1

RPLENRKTHNHLERKRRDELKRKFDDLRKSLPELELHEKAPKVIILTKGIDHIKQLEN

>Hydra18 1099563286887 gb|CX835588.2|CX835588 ACAC-aaa71b12.g1

KERFARENHSEIERRRRNKMNAYINELSDMVPSCNGLVRKPDKLTVLKMAVNYMKSLHG

>Hydra19 1099993121758 gb|DN811566.2|DN811566 ACAC-aab41e15.g1

DEKRSKRAAANVNERKRMQCINNGFESLRELLNLPYKARLSKSAILQYSADLLQTTI

>Hydra20 1099549945599

LDPTSRRASHNVLERKRRIDLKRSFEKLRECVPNLEREEKAPKVVVLKKALMYILALKTE

>Hydra21 1099405372197

DETEPELSKAAHNVLERQRRNELKLRFNFLRDEIPDLAMNDKAPKIQILKRGQEMLKDLKAQ

>Hydra22 1099993505185 gb|CV887639.1|CV887639 tak87h07.y1

VLSRTTHNVLERQRRNDLKIRFNILRDNIPELASNEKAPKIQILKKGLEHLNELKAQ

>Hydra23 1097263625432 gb|CV464404.1|CV464404 taj19h08.y1

STADKRAHHNALERKRRDHIKDSFTGLRDSVPSLEGEKKSSRAQILHKATEHIQYMRRKN

>Hydra24 1096159494148

VVQRDYHNELERRRRQLISSKFMLLQNALPENAFPVKTNLEKVSRCSILNAACRYMSSME

>Hydra25 1099993532280 gb|DR437770.1|DR437770 ACAB-aaa75g12.b1 gb|DN136244.2|DN136244 ACAB-aaa75g12.g1

RSQTYRATHNQLEKNRRAHLRDCLVSLRDLVPNSPDTSKVTTLSLLQSAKQYIKVLENHDR

>Hydra26 1099728050456 gb|CN772910.1|CN772910 tae01d05.y1 gb|CN626611.1|CN626611 tae97f04.y1 gb|CN625949.1|CN625949 tae53b06.y1

KDRQKKDNHNLIERRRRYNINDRIKELGTLVPKLDNDFKQNKGTILKSSVDYIRKLK

>Hydra27 1099288588996

KVKAPAAKKTRVEHVVIEKRYRMKITDSLSELKKLLPCSEKSKVLFVLGERYIFS

>Hydra28 1099414281030

RLLNSKSVNPSKRHRDRLNKELDNLVNLLPFSEDVIRRLDKLSILRLSVSYLRNKSYF

>Hydra29 1099327161039 gb|DT620535.1|DT620535 ACAH-aaa23a05.g1 gb|DT620250.1|DT620250 ACAH-aaa20d07.g1 gb|DT608537.1|DT608537 ACAG-aab16c05.g1 gb|DN245142.2|DN245142 ACAE-aaa54a07.g1

MEQSKRNKSRDAARQRRGKQNGEFGELACQLPLPKGVAENLDRLCTMRLSNSFIKIKHV

>Hydra30 1098589776912

YLGEPSLDYGFSRLTKLLPRHPNDPVKLTKEDLLKRAADVVEFMYSLPSS

>Hydra31 1099993718159

NLTRIQRRTRERKAFKKLKDTVPTLREKRKWTNRLDIIKHTCDYIKELQKLL

>Hydra32 1099993662384 gb|CN770080.1|CN770080 taf75e01.y1 gb|CB890202.1|CB890202 taa51d03.x1 gb|CB890187.1|CB890187 taa51b03.x1

MKEKRRANKPLLERKRRARINNSLNDMKHLVLSFLNKDATKFTKMEKVDILDMTVHYLQQ

>Hydra33 gb|DN242706.2|DN242706 ACAD-aab12l12.g1

TNMDPTAMARTKEPEKNRVKQVNDGFDDLMQRIPFLPDKKKLSKVEILRCAALYIRDLKDIL

***Amphimedon queenslandica.***

>Amq1 BAYB278482.g1

RRMQANKRERKRMHTVNSAFDDLRDLVPTYPSNRKLSKIETLRLACAYIEDLA

>Amq2 BAYA382363.x1

KRQIANSNERRRMQSINSGFHTLRMLMPHLQGEKLSKVSQRKQTNNCSSLT

>Amq3 BAYB348149.g2

RRASHNVLERKRRNDLKNSFDILRTGIPDLEENIRAPKVVILRKAVEYIKFLQ

>Amq4 BAYB465.b2

LRQQKNSKERSRIKSIGQKYNELRRILGFDLTKKRYCKHKILKAAIEYITKLQ

>Amq5 BAYB312071.b1

QRKQKNSQERKRVKSIRLKYNELRKVLGFDTTKKLCKQKILDAAIEYITKLQ

>Amq6 BAYA134873.x1

RRSANNQRERIRVRDINEAFKELGDICHQYLQSERAQTKLMILHQAVAVINSLE

>Amq7 BAYB360646.g1

RRSQHCEVEKRRREKMNRYMSELAQMIPACNAVPRKLDKLSILKMAVDHMKNLR

>Amq8 BAYB315261.g1

YLDSHKSIEKKRRDRINNGLQTLKDIVPNCRQYSSQVRLHLIIMMILHYF

>Amq9 BAYB353653.b1

MSDKITILSFILVERRRRDRINELIKILAEVVPGCQKKDSSNGSKGTVLEKTVEYVREL

>Amq10 BAYB153272.g1

VVSRKKRRGIIEKRRRDRINNCLMELRRLVPAAFEKQGSAKLEKAEILQMTVDHLRHLH

>Amq11 BAYA127499.y1

QSLDKRAHHNALERKRRDHIKDSFTNLRDCIPSLSGEKVSSRAHVLNKATEYIRQMQ

>Amq12 BAYA416750.x1

KMREACSFGINPIERRRRFNINDRIKELGMLLPSSESEARQNKGSILKASVDYIRKLQ

>Amq13 BAYB23754.b1

PGGVVKKKSHNLIEKKYRTSINDRIGTLRDIVSKDDKKVQKSAVLQKTIDYIRYLE

>Amq14 BAYB257706.g1

AAGFRFDIERVVMAVSRRQKENDTFNDIAGTLPIEESAQELDKASVLRIAIHYLKLRD

>Amq15 BAYB204525.b1

GARKRGKVRKESEHKRRLMMNQYFDELVILLSMVTETVSSRKMDKVTTLHEAVSLFKLYYDLDQ

>Amq16 BAYB125174.g1

APDDQIFENSFCRIERVIRQQDDPDQLPKDLVLQRAAELLESCF

***Fungi***

>ref|NP_116692.1| Pho4p [Saccharomyces cerevisiae]

KRESHKHAEQARRNRLAVALHELASLIPAEWKQQNVSAAPSKATTVEAACRYIRHLQ

>ref|NP_009447.1| Rtg3p [Saccharomyces cerevisiae]

KREFHNAVERRRRELIKQKIKELGQLVPPSLLNYDDLGKQIKPNKGIILDRTVEYLQYL

>ref|NP_010408.1| Ino2p [Saccharomyces cerevisiae]

RKWKHVQMEKIRRINTKEAFERLIKSVRTPPKENGKRIPKHILLTCVMNDIKSIR

>ref|NP_012594.1| Cbf1p [Saccharomyces cerevisiae]

RKDSHKEVERRRRENINTAINVLSDLLPVRESSKAAILACAAEYIQKLKETDEANIE

>ref|NP_014533.1| Ino4p [Saccharomyces cerevisiae]

IRINHVSSEKKRRELERAIFDELVAVVPDLQPQESRSELIIYLKSLSYLSWL

>ref|NP_014574.1| Rtg1p [Saccharomyces cerevisiae]

SCGANFKNDRKRRDKINDRIQELLSIIPKDFFRDYYGNSGSNDTLSESTPGALGLSSKAKGTGTKDGKPNKGQILTQAVEYISHLQ

>ref|NP_014675.1| Hms1p [Saccharomyces cerevisiae]

GRVSHNIIEKKYRSNINDKIEQLRRTVPTLRVAYKKCNDLPITSRDLADLDGLEPATKLNKASILTKSIEYICHLE

>ref|NP_014989.1| Tye7p [Saccharomyces cerevisiae]

QKQAHNKIEKRYRININTKIARLQQIIPWVASEQTAFEVGDSVKKQDEDGAETAATTPLPSAAATSTKLNKSMILEKAVDYILYLQ

>[ref|XP_502856.1|](http://www.ncbi.nlm.nih.gov/entrez/query.fcgi?cmd=Retrieve&db=Protein&list_uids=50550767&dopt=GenPept) hypothetical protein [Yarrowia lipolytica]

GRSSHNMIEKKYRTNINDKISALRDCVPALRCALKGTKDDEELDGLTPASKLNKATVLSKATEYIKHLK

>ref|XP_501357.1| hypothetical protein [Yarrowia lipolytica]

KKANHIASEQKRRQAIREGFERITKIVPNLDKSQGRSEAIVLNKTVAFLKNL

>[ref|XP_505311.1|](http://www.ncbi.nlm.nih.gov/entrez/query.fcgi?cmd=Retrieve&db=Protein&list_uids=50555806&dopt=GenPept) hypothetical protein [Yarrowia lipolytica]

RESHNAVERRRRDNINDRIQELSTLIPENFLNEPIIGGSPSLGPMGSPPPSQTPGTKDGRPNKGTILIKSVEYIKKLQ

>[ref|XP_500836.1|](http://www.ncbi.nlm.nih.gov/entrez/query.fcgi?cmd=Retrieve&db=Protein&list_uids=50546733&dopt=GenPept) hypothetical protein [Yarrowia lipolytica]

RRHAHILSEQRRRENINGGFQQLRNAVPYCRGTQLSKAVILKKAVEYIAALE

>[ref|XP_501404.1|](http://www.ncbi.nlm.nih.gov/entrez/query.fcgi?cmd=Retrieve&db=Protein&list_uids=50547869&dopt=GenPept) hypothetical protein [Yarrowia lipolytica]

RKTAHSAIERRRRSKMNEEFDSLKQLVPACRQSIAAEGGDAGLHKLTILQATVEYVRYLQ

>[ref|XP_505019.1|](http://www.ncbi.nlm.nih.gov/entrez/query.fcgi?cmd=Retrieve&db=Protein&list_uids=50555221&dopt=GenPept) hypothetical protein [Yarrowia lipolytica]

KRTSHKLAEQGRRNRINNALADLGKLLVPESASTSKANTVENAIDYIRKLK

>[ref|XP_503220.1|](http://www.ncbi.nlm.nih.gov/entrez/query.fcgi?cmd=Retrieve&db=Protein&list_uids=50551493&dopt=GenPept) hypothetical protein [Yarrowia lipolytica]

RRDNHKEVERRRRETINDGINTLAELIATSEKNKGQILKNAIEFIKQLK

>ref|XP_501786.1| hypothetical protein [Yarrowia lipolytica]KLVHNQIERKYRSAINLGLLRLKSLVPWYQLVEGEDQEVAIKREEEWTEGGAGPSGSGANQMSNSGLHPSQDPSGCPYSLDELATLSSKAGILQVSAEYVQYLQ

>[ref|XP_749262.1|](http://www.ncbi.nlm.nih.gov/entrez/query.fcgi?cmd=Retrieve&db=Protein&list_uids=70988817&dopt=GenPept) HLH transcription factor [Aspergillus fumigatus Af293]

KKRAHNVIEKRYRANLNEKIAELRDSVPSLRASYKQANGNSGDDDDDGVTSASKLNKASILSKATEYIRHLE

>[ref|XP_753074.1|](http://www.ncbi.nlm.nih.gov/entrez/query.fcgi?cmd=Retrieve&db=Protein&list_uids=70996638&dopt=GenPept) hypothetical protein Afu1g17060 [Aspergillus fumigatus Af293]

RASHNVIEKRYRTNMNAKFTTLENVITTCRNKQKASTIRPCSMKKCEILTSAIKCIQDLE

>[ref|XP_746533.1|](http://www.ncbi.nlm.nih.gov/entrez/query.fcgi?cmd=Retrieve&db=Protein&list_uids=70982009&dopt=GenPept) hypothetical protein Afu4g03460 [Aspergillus fumigatus Af293]

KRAAHNIIEKRYRTNMNAKFVALEKAMSGSGVQKPTKGGSGPASLKKSEILTNAIAYMQELQ

>[ref|XP_747924.1|](http://www.ncbi.nlm.nih.gov/entrez/query.fcgi?cmd=Retrieve&db=Protein&list_uids=70984836&dopt=GenPept) HLH transcription factor [Aspergillus fumigatus Af293]

KRTNHKLAEQGRRNRINNALKEIESLIPSAFIQMKQTKENVASHVKGDKEKEKEKAGAPTISKASTVELAIDYIKALK

>[ref|XP_753834.1|](http://www.ncbi.nlm.nih.gov/entrez/query.fcgi?cmd=Retrieve&db=Protein&list_uids=70998216&dopt=GenPept) hypothetical protein Afu5g08020 [Aspergillus fumigatus Af293]

RKNNHKEVERRRREAINEGINQLARLVPNCDKNKGAILQRTIEYICQL

>ref|XP_752334.1| HLH transcription factor GlcD gamma [Aspergillus fumigatus Af293]

RRASHNLVERRRRDNINERIQDLSHLVPQHRLEDDKVRKQLVNNSAMSGNGSS

>ref|XP_752546.1| HLH transcription factor [Aspergillus fumigatus Af293]

RKTAHSLIERRRRSKMNEEFATLKDMIPACRGQDMHKLSILQASIEYVNYLE

>ref|XP_755812.1| HLH transcription factor Hpa3 [Aspergillus fumigatus Af293]

LRVTHKLAERKRRSEMKDCFEALRMRLPQSQNNKSSKWETLTRAIEYIGQLE

>ref|XP_755563.1| HLH transcription factor [Aspergillus fumigatus Af293]

KRNNHILSEQKRRNLIRQGFDDLCSLVPGLKGGGFSKSAMLTQAADWLEDI

>[gb|EAA61847.1|](http://www.ncbi.nlm.nih.gov/entrez/query.fcgi?cmd=Retrieve&db=Protein&list_uids=40742657&dopt=GenPept) hypothetical protein AN7661.2 [Aspergillus nidulans FGSC A4]

AHNVIEKRYRANLNEKIAELRDSVPSLRASKGNGVLDDEDEGVTPANKLNKASILSKATDYIRHLE

>[gb|EAA61422.1|](http://www.ncbi.nlm.nih.gov/entrez/query.fcgi?cmd=Retrieve&db=Protein&list_uids=40742232&dopt=GenPept) hypothetical protein AN7170.2 [Aspergillus nidulans FGSC A4]

RAAHNIIEKRYRTNMNAKFVALEKAMSVKTSGVSKAVSSTNSSSNGVKSSASLKKSEILSNAITYMQELQ

>[gb|EAA60173.1|](http://www.ncbi.nlm.nih.gov/entrez/query.fcgi?cmd=Retrieve&db=Protein&list_uids=40740983&dopt=GenPept) hypothetical protein AN5078.2 [Aspergillus nidulans FGSC A4]

RASHNIVEKRYRINLNSKFRKLHEIVFCRTDPTFVPDIANANGSSNSANSNSEGPASAPVSAIATQANRSQPPKASIIDSALNYIESLQ

>[gb|EAA59009.1|](http://www.ncbi.nlm.nih.gov/entrez/query.fcgi?cmd=Retrieve&db=Protein&list_uids=40739819&dopt=GenPept) hypothetical protein AN8271.2 [Aspergillus nidulans FGSC A4]

KRTNHKLAEQGRRNRINTALKEIETLIPAAYVQMRLHKEAATCNGKGEKEKERTGNQPISKASTVEMAIDYIKSLK

>[gb|EAA61249.1|](http://www.ncbi.nlm.nih.gov/entrez/query.fcgi?cmd=Retrieve&db=Protein&list_uids=40742059&dopt=GenPept) hypothetical protein AN7734.2 [Aspergillus nidulans FGSC A4]

RKNNHKEGTCSVERRRREAINEGINQIARLVPNCDKNKGAILQRAIEYINQL

>gb|EAA62133.1| conserved hypothetical protein [Aspergillus nidulans FGSC A4]

LRVTHKLAERKRRSEMKDCFEALRLRLPSSQNNKSSKWETLTRAIEYINNLE

>gb|EAA62917.1| predicted protein [Aspergillus nidulans FGSC A4]

KRKQHAQAQKLQRDRMKSALDRMARMLATGTGGVHAGTASCGTKAELVEAAVEYIERL

>gb|EAA64503.1| predicted protein [Aspergillus nidulans FGSC A4]

RRLLHIIAERNRRLHQNRMYDELYKMVPGLENSSRSTKREVLMRTVDFLAEL

>gb|EAA65926.1| hypothetical protein AN0897.2 [Aspergillus nidulans FGSC A4]

KKNNHIASEQKRRAAIREGFDRLTELVPGLEGQGRSESIVLKKTVDFIHA

>gb|EAA66232.1| hypothetical protein AN1114.2 [Aspergillus nidulans FGSC A4]

RKTAHSLIERRRRSKMNEEFSTLKNMIPACRGHEMHKLAILQASIDYVNYLE

>gb|EAA66495.1| predicted protein [Aspergillus nidulans FGSCA4]

RRASHNVVEKRYRENLNRKFHLLETIVNKGTEPYSCSPCSSPRSSPSSSGSRKGNTTFSSSSSSSARRQYTSPKATIIDSALSYIESLR

>gb|EAK93265.1| [Candida albicans SC5314]

KNSHNMIEKKYRTNINTKILALRDAVPALRIAAGCDDVSIADLEGLTPASKLNKASVLTKATEYIKHLE

>gb|EAK94880.1| [Candida albicans SC5314]

RREFHNAVERRRRDLIKERIKELGVIVPPSLLNPTLSAVQNFQRKGSIESGELSELIGSVKVKETKPNKSTILNRSVDYINHL

>gb|EAK96568.1| [Candida albicans SC5314]

RRENHKEVERKRRESINTGIRELARLIPTTDTNKAQILQRAVEYIKRLK

>gb|EAK97511.1| [Candida albicans SC5314]

KKASHKLAEQGRRNRMNNAVQELGRLIPQSYHDEVSIPSKATTVELASKYITAL

>gb|EAK97566.1| [Candida albicans SC5314]

KKASHKLAEQGRRNRMNNAVQELGRLIPQSYHDEVSIPSKATTVELASKYITAL

>gb|EAL01056.1| [Candida albicans SC5314]

KQLHSIIEKRRRIKINREFEALKYLIPACRNCNTGSSGGSATPTSSTKKASTNSNNNGNKIDGMYKLTILKSSVEYILYL

>gb|EAL03501.1| [Candida albicans SC5314]

QKKAHNKIEKRYRININAKIAGIQKIIPWVAFEKTAFETGEENETEAEAKNNTRLNKSMILEKATEYILHLQ

>gb|EAL04710.1| [Candida albicans SC5314]

QNDKDVGNERKRRDNINDKIQELLTLIPSEFFQSNTDNNTKTGTKVVKENSPEDDAVKNSGTKDGKPNKGQILTKSVEYLQYLQ

>[ref|XP_567526.1|](http://www.ncbi.nlm.nih.gov/entrez/query.fcgi?cmd=Retrieve&db=Protein&list_uids=58260232&dopt=GenPept) hypothetical protein CNJ02310 [Cryptococcus neoformans var. neoformans JEC21]

HNKTERRYRQKVQAAQADLRDAIPALRLLYGTSTPEQLATTDIRAPDGTVDGLGEVTRPNASAKATILIGARVYIELLQ

>[ref|XP_567552.1|](http://www.ncbi.nlm.nih.gov/entrez/query.fcgi?cmd=Retrieve&db=Protein&list_uids=58260284&dopt=GenPept) hypothetical protein CNJ02620 [Cryptococcus neoformans var. neoformans JEC21]

RRECHNLVEKRRREHINAKIEELGTLLPEKYNQIDEPAEEEDEDGKTSAKKKKSKRGGNTSAKSQKDAAHCKGRILSQSVNYIRYV

>[ref|XP_570115.1|](http://www.ncbi.nlm.nih.gov/entrez/query.fcgi?cmd=Retrieve&db=Protein&list_uids=58265918&dopt=GenPept) hypothetical protein CND04370 [Cryptococcus neoformans var. neoformans JEC21]

RKDNHREVESRRRQAIADGIAEIAQLLPSPPAPKEGKGQLLKRAVTYIHEL

>ref|XP_567168.1| [Cryptococcus neoformans var. neoformans JEC21]

LRVSHKLAERKRRKEMKDLFDELRDELPADRGMKASKWEILTKAIEHIKHTK

>ref|XP_567224.1| [Cryptococcus neoformans var. neoformans JEC21]

RRLAHLMSEQKRRESINSGFQALRATLPTSLTTDSKAVILRKAVSRISYLE

>ref|XP_571110.1| [Cryptococcus neoformans var. neoformans JEC21]

RKVNHSLIERRRREKINAALNELRRMVPSLGENGGKGGEFKLEVLEKTVEHMKDLK

>ref|XP_572235.1| [Cryptococcus neoformans var. neoformans JEC21]

KRSQHNAIERARRETLNSKFLDLARLLPSLASSRRPSKSAIVNGSISHLTYQRKQRLLAAKLLKQVR

>[ref|XP_461840.1|](http://www.ncbi.nlm.nih.gov/entrez/query.fcgi?cmd=Retrieve&db=Protein&list_uids=50426487&dopt=GenPept) hypothetical protein DEHA0G07381g [Debaryomyces hansenii CBS767]

SHNMIEKKYRTNINSKIVALRDAVPSLKIVAGNNNVSISDLEGLTPASKLNKASVLTKATEYIKHLE

>[ref|XP_462194.1|](http://www.ncbi.nlm.nih.gov/entrez/query.fcgi?cmd=Retrieve&db=Protein&list_uids=50427163&dopt=GenPept) hypothetical protein DEHA0G16060g [Debaryomyces hansenii CBS767]

REFHNAVERRRRDLIKERIKDLGFLVPPSLLNPQLCAVQNLQRNSQLNSREINDLLASIKVKETKPNKSTILNKSVDYIIHLK

>[ref|XP_459897.1|](http://www.ncbi.nlm.nih.gov/entrez/query.fcgi?cmd=Retrieve&db=Protein&list_uids=50428235&dopt=GenPept) hypothetical protein DEHA0E14190g [Debaryomyces hansenii CBS767]

KDDPQVNERKRRDNINEKIQELLTLIPAEFFQENPPATNQPQQQQTQQSENDAAIAAAVKNSGTKDGKPNKGQILTKSVEYLQYLQ

>[ref|XP_460142.1|](http://www.ncbi.nlm.nih.gov/entrez/query.fcgi?cmd=Retrieve&db=Protein&list_uids=50423123&dopt=GenPept) hypothetical protein DEHA0E20361g [Debaryomyces hansenii CBS767]

KSAHNVIEQRYRNKINDKFTVLQNTVPSLRVAKRKLKGLASPDFKQDYDEDPINDYSGSEDEDLEGLEPAKKLNKGTILTKSIEYIKFLE

>[ref|XP_458014.1|](http://www.ncbi.nlm.nih.gov/entrez/query.fcgi?cmd=Retrieve&db=Protein&list_uids=50418987&dopt=GenPept) hypothetical protein DEHA0C08481g [Debaryomyces hansenii CBS767]

KKASHKLAEQGRRNRMNMAVQELSNLIPQAYHDEVSIPSKATTIELASKYIRDL

>[ref|XP_457129.1|](http://www.ncbi.nlm.nih.gov/entrez/query.fcgi?cmd=Retrieve&db=Protein&list_uids=50412396&dopt=GenPept) hypothetical protein DEHA0B03740g [Debaryomyces hansenii CBS767]

RRENHKEVERRRRESINQGIKELAALIPTNDTNKAQILQRAVEYIKRLK

>ref|XP_457173.1| Debaryomyces hansenii CBS767]

KKAHHIASEQKRRENIRSEFDKIVSLTPTLNELENRSELNILTKSADYIDQLK

>[ref|NP_985240.1|](http://www.ncbi.nlm.nih.gov/entrez/query.fcgi?cmd=Retrieve&db=Protein&list_uids=45190986&dopt=GenPept) AER385Cp [Eremothecium gossypii]

RMSHNIIEKKYRTNINDKILQLREIVPALRVASKREDGVTVGEDDIKQLDGLEPARKLNKASILTKTIEYIKHLE

>[ref|NP_984466.1|](http://www.ncbi.nlm.nih.gov/entrez/query.fcgi?cmd=Retrieve&db=Protein&list_uids=45188243&dopt=GenPept) ADR370Wp [Eremothecium gossypii]

AHNKIEKRYRININTKLAKLQQIIPWVASEATAFEVDDALRQKSSPTMMDTATLTSATPKLNKSMILEKAVDYILFLQ

>[ref|NP_984849.1|](http://www.ncbi.nlm.nih.gov/entrez/query.fcgi?cmd=Retrieve&db=Protein&list_uids=45190595&dopt=GenPept) AEL012Cp [Eremothecium gossypii]

ERKRRDNINDRIQELLNVIPEEFFQDYYQKKKDQESESGTPGALPKNKGTGTRDGKPNKGQILTQAVEYVTYLQ

>[ref|NP_984163.1|](http://www.ncbi.nlm.nih.gov/entrez/query.fcgi?cmd=Retrieve&db=Protein&list_uids=45187940&dopt=GenPept) ADR067Wp [Eremothecium gossypii]

RREFHNAVERRRRELIKSKIKELGKLVPPSLLNYNDEGKEVRLNKGIILLRTVEYLEYLR

>[ref|NP_983973.1|](http://www.ncbi.nlm.nih.gov/entrez/query.fcgi?cmd=Retrieve&db=Protein&list_uids=45187750&dopt=GenPept) ADL123Cp [Eremothecium gossypii]

KKEVHKVAEQGRRNRLNNALAELNDLLPPELKESAQVPSKATTVELACKYIRQL

>[ref|NP_985961.1|](http://www.ncbi.nlm.nih.gov/entrez/query.fcgi?cmd=Retrieve&db=Protein&list_uids=45198932&dopt=GenPept) AFR414Wp [Eremothecium gossypii]

RKDSHKEVERRRRENINTAINKVAELLPVKESSKAAILSRAAEYIQELK

>ref|NP_983985.1| [Eremothecium gossypii]

QRERFLELERERREEKKRQIEELARILDKKITDYQIAALAGRVSEFQESLQ

>ref|NP_984781.1| [Eremothecium gossypii]

KRENHVTSEQRRREILREYYDELVRLVPDLQESENRSEWQIYMKTRNYL

>ref|NP_986724.1| [Eremothecium gossypii]

RRWKHVYCEKQRRTIFRQAFDDLISMVRFPRPTDLEISCLRGPRACTEDRVRKKRAPGDGKRIPKHVLLRYIAEDIE

>[ref|XP_454706.1|](http://www.ncbi.nlm.nih.gov/entrez/query.fcgi?cmd=Retrieve&db=Protein&list_uids=50309397&dopt=GenPept) unnamed protein product [Kluyveromyces lactis]

RTSHNVIEKKYRTNINNKIVQLKEIIPSLCVTMKREEGIPVTELDHLRLDGLQPAKKLNKASILVKTIEYIQHLE

>[ref|XP_454514.1|](http://www.ncbi.nlm.nih.gov/entrez/query.fcgi?cmd=Retrieve&db=Protein&list_uids=50309017&dopt=GenPept) unnamed protein product [Kluyveromyces lactis]

HNDSEKKYRNNINTHFQFLRNSVPTLRWCDDNSIPVESLEGLAPPSKVNKVQILSKSHEYIKHLE

>[ref|XP_456120.1|](http://www.ncbi.nlm.nih.gov/entrez/query.fcgi?cmd=Retrieve&db=Protein&list_uids=50312177&dopt=GenPept) unnamed protein product [Kluyveromyces lactis]

RVTHNMIEKRYRININTKIGKLQKIIPWVACEDTAFVVDNKVLSAGEDSLPLKKVKLNKSMILEKAVDYILYLQ

>[ref|XP_453770.1|](http://www.ncbi.nlm.nih.gov/entrez/query.fcgi?cmd=Retrieve&db=Protein&list_uids=50307581&dopt=GenPept) unnamed protein product [Kluyveromyces lactis]

QKQVHKVAEQGRRNRLNNALKDLESLIPKELKDVAAIPSKATTVELAAQYIRQL

>[ref|XP_452143.1|](http://www.ncbi.nlm.nih.gov/entrez/query.fcgi?cmd=Retrieve&db=Protein&list_uids=50304387&dopt=GenPept) CBF1_KLULA [Kluyveromyces lactis]

RKESHKEVERRRRQNINTAIEKLSDLLPVKETSKAAILSRAAEYIQKMK

>ref|XP_454241.1| [Kluyveromyces lactis]

RREFHNAVERRRRELIKAKIKELGTLVPPTLLHFDDFGKKVKPNKGTILKRTIEYMDCLK

>ref|XP_455740.1| [Kluyveromyces lactis]

RGNINELIQQFLSIIPEEFFEEYYRKNSDPKIKDEPVSKSKGTSANANKPTKGQILTQAVEYVNSLQ

>ref|XP_455958.1| [Kluyveromyces lactis]

RRWKHVYLEKQRRNTFKREYDELIGMIRYPRPVWTEVHKNLPKSKEIISLDYKLPKKEGKRITKHTLLNYIVQDIQ

>[ref|NP_595694.1|](http://www.ncbi.nlm.nih.gov/entrez/query.fcgi?cmd=Retrieve&db=Protein&list_uids=19112486&dopt=GenPept) hypothetical protein SPBC19C2.09 [Schizosaccharomyces pombe 972h-]

KTAHNMIEKRYRTNLNDRICELRDAVPSLRAAAALRCGNSLDDEDLGGLTPARKLNKGTILAKATEYIRHLE

>[ref|NP_595229.1|](http://www.ncbi.nlm.nih.gov/entrez/query.fcgi?cmd=Retrieve&db=Protein&list_uids=19112021&dopt=GenPept) hypothetical protein SPBC354.05c [Schizosaccharomyces pombe 972h-]

RSAHNMIEKRYRSNLNDKIAELRDAVPTLRSGYNSTTADELKGTYVPLSRKLNKATILSKATEYIKSLQ

>[ref|NP_593944.1|](http://www.ncbi.nlm.nih.gov/entrez/query.fcgi?cmd=Retrieve&db=Protein&list_uids=19114856&dopt=GenPept) hypothetical protein SPAC3F10.12c [Schizosaccharomyces pombe 972h-]

KRLSHKEVERRRREAISEGIKELANIVPGCEKNKGSILQRTAQYIRSLK

>[ref|NP_593230.1|](http://www.ncbi.nlm.nih.gov/entrez/query.fcgi?cmd=Retrieve&db=Protein&list_uids=19114142&dopt=GenPept) hypothetical protein SPAC56F8.16 [Schizosaccharomyces pombe 972h-]

LRTSHKLAERKRRKEIKELFDDLKDALPLDKSTKSSKWGLLTRAIQYIEQLK

>[gb|EAA70763.1|](http://www.ncbi.nlm.nih.gov/entrez/query.fcgi?cmd=Retrieve&db=Protein&list_uids=42547920&dopt=GenPept) hypothetical protein FG08403.1 [Gibberella zeae PH-1]

KTAHNMIEKRYRTNLNDKIAALRDSVPSLRIMTKSARGEDTTEDREELHGLTPAHKLNKATVLSKATEYIRHLE

>[gb|EAA71859.1|](http://www.ncbi.nlm.nih.gov/entrez/query.fcgi?cmd=Retrieve&db=Protein&list_uids=42549016&dopt=GenPept) hypothetical protein FG02814.1 [Gibberella zeae PH-1]

KTAHNMIEKRYRTNLNDKISALRDSVPALRVMVHRLEQPCVEGMEEDGDEDLGGLTPAHKLNKATILSKATEYIAHLE

>[gb|EAA76055.1|](http://www.ncbi.nlm.nih.gov/entrez/query.fcgi?cmd=Retrieve&db=Protein&list_uids=42553212&dopt=GenPept) hypothetical protein FG09308.1 [Gibberella zeae PH-1]

RTAHNDIERKYRTNLKDKIAELRDAVPALHSIPEDGDNNEAEGDSQRAPKVSKGTVLTKATEYIQHLE

>[gb|EAA69380.1|](http://www.ncbi.nlm.nih.gov/entrez/query.fcgi?cmd=Retrieve&db=Protein&list_uids=42546537&dopt=GenPept) hypothetical protein FG00041.1 [Gibberella zeae PH-1]

QRPHYAIEKRYRAGLQERFEALRDCVASLKKTQHEQRLPGTNEDLAEGDDGGSVSDRATVGRMNKAEVLNQATLCIRQLQ

>[gb|EAA68581.1|](http://www.ncbi.nlm.nih.gov/entrez/query.fcgi?cmd=Retrieve&db=Protein&list_uids=42545738&dopt=GenPept) hypothetical protein FG00545.1 [Gibberella zeae PH-1]

KRTSHKIAEQGRRNRINSALQVMAGLLPGGDKTDIVDEGDKKDGKQANAQNSKASVVENAIVHMKSLE

>[gb|EAA75111.1|](http://www.ncbi.nlm.nih.gov/entrez/query.fcgi?cmd=Retrieve&db=Protein&list_uids=42552268&dopt=GenPept) hypothetical protein FG05567.1 [Gibberella zeae PH-1]

RKDNHKEVERRRRETINEGINELAKIVPGCEKNKGSILQRAVSFISQLK

>[gb|EAA31130.1|](http://www.ncbi.nlm.nih.gov/entrez/query.fcgi?cmd=Retrieve&db=Protein&list_uids=28921855&dopt=GenPept) hypothetical protein [Neurospora crassa]

KTAHNMIEKRYRTNLNDKIAALRDSVPALRIMSKSARGEDTTEDREELHGLTPAHKLNKATVLSKATEYIRHLE

>[gb|EAA36266.1|](http://www.ncbi.nlm.nih.gov/entrez/query.fcgi?cmd=Retrieve&db=Protein&list_uids=28927312&dopt=GenPept) hypothetical protein [Neurospora crassa]

KTAHNMIEKRYRTNLNDKIAALRDSVPSLRIAAMRMESGNYDDEYEGEEGDLSGLIPAPKLNKATILSKATEYISQLE

>[gb|EAA28655.1|](http://www.ncbi.nlm.nih.gov/entrez/query.fcgi?cmd=Retrieve&db=Protein&list_uids=28919156&dopt=GenPept) hypothetical protein [Neurospora crassa]

RTAHNDIERKYRTNLKDKIAELREAVPALHTISEAGGVEDDGSQNSRAPKVSKGTILTKATEYIHQLE

>[gb|EAA34721.1|](http://www.ncbi.nlm.nih.gov/entrez/query.fcgi?cmd=Retrieve&db=Protein&list_uids=28925710&dopt=GenPept) hypothetical protein [Neurospora crassa]

KRTSHKIAEQGRRNRINSALQEIATLLPKAPAKEADREDKREKDKDKAGGGIPNSKASTVEMAIEYIKQLQ

>[gb|EAA29791.1|](http://www.ncbi.nlm.nih.gov/entrez/query.fcgi?cmd=Retrieve&db=Protein&list_uids=28920423&dopt=GenPept) hypothetical protein [Neurospora crassa]

RKDNHKEVERRRRETINEGINELAKIVPGCEKNKGSILQRAVQFITQLK

>[gb|EAA35166.1|](http://www.ncbi.nlm.nih.gov/entrez/query.fcgi?cmd=Retrieve&db=Protein&list_uids=28926182&dopt=GenPept) predicted protein [Neurospora crassa]

RVNHNQVEKQYRNRLHEYFDNLLKVLPDNPGMMEPKAEPENDDESQSSSSASGRKSRNWSKAEVLERACHHIHELQ

>[gb|EAA34934.1|](http://www.ncbi.nlm.nih.gov/entrez/query.fcgi?cmd=Retrieve&db=Protein&list_uids=28925940&dopt=GenPept) predicted protein [Neurospora crassa]

RKTAHSLIERRRRSKMNEEFALLKSMIPACTGEMHKLAIL

>[gb|EAK85602.1|](http://www.ncbi.nlm.nih.gov/entrez/query.fcgi?cmd=Retrieve&db=Protein&list_uids=46100369&dopt=GenPept) hypothetical protein UM04317.1 [Ustilago maydis 521]

RVAHLLSEQKRRESINTGFEDLRQAIPACRDGQDSKATILKRALEYIRELE

>[gb|EAK85976.1|](http://www.ncbi.nlm.nih.gov/entrez/query.fcgi?cmd=Retrieve&db=Protein&list_uids=46100743&dopt=GenPept) hypothetical protein UM05721.1 [Ustilago maydis 521]

KVAHNAIERRYRNNINDRIAALRRAVPALREIRPRKTPSGRRSRKAQQEEDLVDGVPAATKLNKATILGKATDYIKYLK

>[gb|EAK83487.1|](http://www.ncbi.nlm.nih.gov/entrez/query.fcgi?cmd=Retrieve&db=Protein&list_uids=46098254&dopt=GenPept) hypothetical protein UM02449.1 [Ustilago maydis 521]

RKDNHKEVERRRRSAINDGIVQLSHIVPGCDAKNTNKGAIIHAAVRYIQDLK

>[gb|EAK84122.1|](http://www.ncbi.nlm.nih.gov/entrez/query.fcgi?cmd=Retrieve&db=Protein&list_uids=46098889&dopt=GenPept) hypothetical protein UM02950.1 [Ustilago maydis 521]

RRESHNAVERRRRDNINGKITELATLLPEAMLQAKPNKGIILRKSVEYIRHL

>[gb|EAK87065.1|](http://www.ncbi.nlm.nih.gov/entrez/query.fcgi?cmd=Retrieve&db=Protein&list_uids=46101832&dopt=GenPept) hypothetical protein UM06227.1 [Ustilago maydis 521]

RVSHKLAERKRRKEMKELFDDLRDQLPVDKGPKTSKWEILSKAVEHIAQL

>[gb|EAK86419.1|](http://www.ncbi.nlm.nih.gov/entrez/query.fcgi?cmd=Retrieve&db=Protein&list_uids=46101186&dopt=GenPept) hypothetical protein UM05486.1 [Ustilago maydis 521]

RRATHSQIERRRREKINDRLVTLRSIVPACAKELEDRRRQKQEEQDEAARIAAGGAPKTYIDAAT
