# Supplementary material for: Origin and diversification of the basic helix-loop-helix gene family in metazoans: insights from comparative genomics
Source: BMC Evol Biol. 2007 Mar 2;7:33. doi: 10.1186/1471-2148-7-33 (PMC1828162; doi:10.1186/1471-2148-7-33)
Supplement: Additional file 16 — Gene assemblies for the bHLHs from Amphimedon queenslandica. Nucleotide sequences of the contigs we assembled for each bHLH gene are shown together with the corresponding predicted proteins using Genscan and Geneid. In some cases, we also report additional sequences from EST data and from PCR products. [file 1471-2148-7-33-S16.doc]

**>Amq_1**

CGGCAGGGAAAGAGAGTCTACCAGCTAATATTAAGAGAGGCAGGAGGTACTGCTGTACTGCTACAGTGTTAACCTGTCCTACACTTCTGAGTTTCAATAGAGCTCTCTTGTAGATAAGTAAAGTTTGTAATTAAGAGCATTTTAACAGGAAAACAGAGGCAGAGGCTAAATAATAAAGACATGATGTCTGGTGGAGAGAACAGGCTATATCCGAACAGCAGCAGCAGCAGCAGTTCCTCTTCACTCCACAGATACCAGTTGAACGTGGCTGGCTCTCAACAGGACCCTATTGACTATACTCTAGGTCCTACCATTCCTTCAAGCAGCAGCAGTTTAGCTAGCAAAGGAGCCGGAAGCTCTTTCTCCCGTAGCTCCAGCAGTTCCAGCACTTGCAGTACCACGAGCAGTAACCTAATGTTGAGAAGGATACCATACGAGCCTAACCCGTCACCCCCAGCCACTGCTGGAGGCCTTAGCTCAGACGAGAGCCTTCAAGATGACTCTGTATCTATAGGAGAGGGAGAGGGAGACCTGGGGCCATTGACTGGAGACCAGAAGAGAATGAGAAGGATGCAAGCTAACAAAAGAGAGAGGAAAAGAATGCACACTGTTAATTCAGCTTTTGATGACCTGAGAGACCTAGTACCAACTTATCCATCAAACAGGAAACTCTCTAAAATAGAAACCCTCAGACTGGCCTGCGCTTACATAGAGGATCTTGCCAAGTTATTGAGAGAGTCTACTGGAGCTGCTGTTGTTCATGGGGAGGATGTGAACTTGCACCATCCACACCTGAACCCACCTCTCCCTCACAGCACTGGGGACACCACTGGGTTCCTCCCTCCTCCTCGTACCTACTCCCCCATTAAGAACGAATACAACAGACTTCAGTCACAAGACTACAGCACCTGCAACTTCCAGCACTATAGGGTACCACTTTCATATGTAAGTACAGCAGCATCATTAACTTCATTATCATTATTATTATTGTTATTCATAGGATTACGAGAGCTGTCCTTCGGATCCTGAGATTCAGTCTCCCCCTACCTACCGTACAGGGAGTGGCCCTCCTGTCTCGTACATGTCAGCTGCTGGTGGTTTGGCTGTTAAGCCGCCAGCTCTAGCACGCGTTAGTTCAGATACTGCAGTTTCTCTTCAAATGGCACAAACTTCACTAAACACTAGTAGTTCAGGTTACTGTGGGAGTCCTTCCGTCACTCCGTGTTCACCGTTGACCAGAGTACAGCACTCGACACCATCAGCTGCTCCAGCGTCCCTCGTATCACGTAATAATATACCATCGTCACACTTTCTACCATCGTCCACCACTTCAAATTATTATCATCATCACAGCTTACCCACCCCTGGCTTCACGCAGTGCTACAGTCAGTGAATGCTCTTTCAATCAATTACCAGGTTATTATTATTATTGTTATTATTATCTTTGTTAAAATTATAATATTTTGTACAATATATTGTTATTATTTTTAAGAGAATTAATCTCTTTTATGATTTTTGTTTAGAATTATTAATTATTATGATAATAGCAATCAATAGACAACATTCACTTTACATAAAGTACAGTTAATCACTTTTAAAATTATAATTTCACTTTTTTGATATAATTTTTAAATATTCAAATATAATTACGTCCAGACAGTCTTCAGTGAATAGAGTCCCAGTGGTATATCCAGTGACAACAGTCTTATTGATGTAGTCAATTAGCAAGTTTGTATAAATCAGTGTAAAATAGACTAACTCATAGACACTTCACTCTAATCTGCTGATCACTGCTATAGATACACTAGTGTCTGGACTAGTGTTGATTGATGAACCTTCACACACGCTCACTCACTGAGTATAAGAATCCTTTATGATGCATCAATACAAAAGTACCTCTGAAATGAGGACACCTCTGTATAGAGGATAGGTATTAGATTCTAAAGTAATCCATTGATATACAAACTACAATGACAGAATGACCAAAACTATGAAAGATAAATCATCATTATGTTATAAAAACGGTTCATGTATAGTTACAAAGTTACAAAGGAGTATCATTAGAGGAAGCTTGAGGGACAGAGTGAGGATCAATCATTGGTTCATCTGTTTGACCAATAACGGCAGACAAGCTGACTAGCCTGTCCCTTCCACTGTCTGTCTCCTCAATGGCCTCAAGAGGAGAAGCTACCAGACCAGTCCTACTGCTAGTGGGAATCGTAGGAAATGATGGAGTCGTATGACGTCTCTGGAAAGTAGATCTCCTTCCCTTCCTCACCTGTAGTCTCTTGCGAAGTTTAACTTTCTGAAGATGATGATCAATTTCATCATTTTCATCATGAATTATAACATGATTGTAATGATCTTCATGAGGCTAATGATGATTAATAATAATAATAATAATAATAATAAATTATTAATTAACACACGTTAAATCGATTTGCTTTCAAAATCTCATCAAGAATAGGAACAGCATCTTCACAACTTTTAGCTGCTTCACAAACTTCATCATCTTAAAAATAATAATAATGACGATTATTAAAATATTTGTATTGTTGAATAACAATCGGCAGACAGTCTAAGATAATTTTTTTGCTACTATATTCCCTTTTATGGACATAATGCGAGACACATGTGCCCTCCTCTACTGTTACCATGGCAACTAACGTACATACATCTAAACATGTAACACATAGTGAGAAAGAGAGAGAGAAAGGGAGGGCCATTGTTTACTGGTTTATAATAATCACATTAAAATGAAGACAGTCGGTTATAATGAGTCTGTCTGTGTGTGTCCTTGAGTGTGTGTGGGGGTAAGGGTAGAAGGAGATTGGTGCATGCCCAGACGTACACAACCTGTTTTTAATAACAATATCCCTCTCTCTCTCTCTCCACCACTGGCTAATATAGAATAACAGCTACTCAAATGAAACAAGAACTGGATGGGTATACAGCAAATAGTAAACAAAGTGCATCAATAATCCAGCGACAAATAAGGACTGTTTATTGAAGGATTAAATATTTTAATAATTGCTCGCCGTGACCACAAGAATATGAATATGATTATAGTACACACCTATTGGCATTCTTAACTAAACCGCAAAATAAATAAAATTAATAATATTTAAATAAAGATAAAAATTACTGTATAGCAACTCACTCAATAGTCTGTGTTCTTCATGAGTCTAGAGTAGATTGGAAAAAAAAGGATATGAATTATATAAAAATAAACAAGTGACAGATGAAGATATACTAGTCTCCCTTTCTCTCTCTCCTACTCAGTTGGCTGTGTTTATAGTAAAAGGTACTCTTTGTAAGTAATCCTACACAATATGGCCACATCAAACCAATAGAACCAAAACCACAGGAAATACCTGTAGCTCTACATAAAGGACAATTATCAGTAAATCACTTTTGGAGTCATTTCCTTTGGTAGTAATGACAGGTATTGGATGACATAGACATGTAATATAATATACTAATAATATACTAATGATATACTAATAGTATGCAATAGCTGACAATATGTACATAACTGAGAAATGAAGACACAGAAACAAACACACAAACACCACACACTCCTCCACTTACACTCTACATGTACTCCAATTATTAATACATGTACTATAACTACAATATTATGTATTGTCTTGGTAGCATTTACTTCTGTATTGCAGTGATGAGATTCCACTTCTTGAGTTGATTCTCTATTAATTAAAAACAATTAAAAATAATTGAAACTACATCAACAAAGCATGTCAATACTAATGCGACAAGTGTGTGCATTAATTAGAGATACTATAGGATTAAATTGAATCTCATTGTGTGGATGATGAATCTCTCCCAAAAATGTAGCTGAGGTACACCACTATCCACAAGCAAATACAACAATATCTTAATATCACTTTATGTCTGGAGGTACATTCACTAAATAATCCAAGGACAGATGGGAATCGGTTTCTAAAAGTTGCTTGAGTATATCATTTGATATTCGAATACAATCAAAGGCAGCTGGTTCAGAATAAGCTCATTACACAAAACTAACAATAGTGATAATATTAAAAATATTATAATAATATTGAAAATTAATATTTGTTGAAAACAACCACGAGAAATATTGCAGGTGGACATATAAAGTGTAACACGAGAGATTGTTGGAATGTGATGTTTACTACGAGTAACCTACCACACTCTGTCCTATAGAGTGATTAATACAAGCAACACTCCATTAGAATACAGTCTGCCACACTCTGTCCTACCTCTATACTGAGTAGTAGCAGTGTACATGCAGCTAATACTGCTAGGTCGTAGTGATCACTCATACCAGCTCTAATCTCATTATCTTGATACACGAATATACTTGTACTTACTTAAACTGCAATTTCTTAAATTTGTTAATTATATCTCGATCTTTGGCCTCAGGAAATCTCTCAAATAGTTTCCGGAAATATTTAACATCAAGAAACTGTAGGATTTCCTTTAAATAGAGATCAATAGGGTATATATGGTATGTGTAGGTACCACTAATCTGTTGAATCCAAAGGTACCAGCAATATCCTGCATACCAGAATTAATGTGCTCCATTGTCTATGGCAATTAATTAATATCGATTAATTAACTATGAATATTAATTACATCATTATGAATGACTTCGTTCAGTGACACTTGATGGACATTCTCAATCTTAACTTTAATCATTCTCACTAGTGGCTTTATGGTGGTGCCCTAGGAGGTAGATATGGTTAGTGGGTGTGGTCAATAACTATGAATATTAATTACCTGTACAAAGACAGTAAAGAGGAGGAGGGCAGTGACTGCAGTGACCATTCGCTTCCTCAGAGTCCTCTCCCCCTCAGGAAGGGAAGACTGACCCTCTTCAAACAATCGTGTCAACGCCAGAGAAAAGGCAACAGCTCCTCTTAGGCCACTGAAGGACTTTATAACACATAACTGACCAATCAGATTGCTTCATTAGCTAACCATTATAAAGGTGTCCTTGAGTGTGATTTTATGAAAGCGGAGCCTATTAAGGAGCAGAGTTAAGACAAACGTACCTAAAGTGGGTGTGGTTTTAATAAGCGGGTGTGGTTATATTGAGTAGGTGTGGCCTACCAATAGGTCTGAATATTAATGTCAGTACAATGGTCCAGAGGACAAGACCAGTGTCCCAACTGTGGAAATGATCATCATAAAGAGCGATACCAAGATAAAGGAATATGACCATCTCCGCCAACACACTAAATTAGTCAATTAATTAAATTAATTAATACATTTGTTAATATTTACTTTATCATTTTCAGGAAATACTTAAAAGTGATGTTTGACTTGAATGACAAGTTTGGAGGGACGTAGTGCTTGACCATTA

>genscan_predicted_peptide

MMSGGENRLYPNSSSSSSSSSLHRYQLNVAGSQQDPIDYTLGPTIPSSSSSLASKGAGSS

FSRSSSSSSTCSTTSSNLMLRRIPYEPNPSPPATAGGLSSDESLQDDSVSIGEGEGDLGP

LTGDQKRMRRMQANKRERKRMHTVNSAFDDLRDLVPTYPSNRKLSKIETLRLACAYIEDL

AKLLRESTGAAVVHGEDVNLHHPHLNPPLPHSTGDTTGFLPPPRTYSPIKNEYNRLQSQD

YSTCNFQHYRVPLSYDYESCPSDPEIQSPPTYRTGSGPPVSYMSAAGGLAVKPPALARVS

SDTAVSLQMAQTSLNTSSSGYCGSPSVTPCSPLTRVQHSTPSAAPASLVSRNNIPSSHFL

PSSTTSNYYHHHSLPTPGFTQCYSQ

>geneid_v1.2_predicted_protein_1|385_AA

MSGGENRLYPNSSSSSSSSSLHRYQLNVAGSQQDPIDYTLGPTIPSSSSSLASKGAGSSF

SRSSSSSSTCSTTSSNLMLRRIPYEPNPSPPATAGGLSSDESLQDDSVSIGEGEGDLGPL

TGDQKRMRRMQANKRERKRMHTVNSAFDDLRDLVPTYPSNRKLSKIETLRLACAYIEDLA

KLLRESTGAAVVHGEDVNLHHPHLNPPLPHSTGDTTGFLPPPRTYSPIKNEYNRLQSQDY

STCNFQHYRVPLSYDYESCPSDPEIQSPPTYRTGSGPPVSYMSAAGGLAVKPPALARVSS

DTAVSLQMAQTSLNTSSSGYCGSPSVTPCSPLTRVQHSTPSAAPASLVSRNNIPSSHFLP

SSTTSNYYHHHSLPTPGFTQCYSQ*

**>Amq2**

AGGGATAAAATTGGGATTTTTTTCCTTTCAAAAATTTTAAACAAACCCCATCAATTTGAATGTATGTTTTTCTTAGGGCGAGGAAAAATAGATCCGGGGTCTTGACCAGTTTTGTCTTTTACGCATCCGGAAAAGGTCGTGAATCCGTTCATCTTTTGCAAGACGGGCAAGTCGCCCTTCTATTTTTTACAAACACTTCCTTATACTGTACCGATGCATACATTTTTGCATCTAGGTCGGTAAATTCATCGATAGACCACATCGTGGTCTTGCAAACTTTGGGGGGAAATTTTCCGCATGGATTTGTAAAAATCAATTTTTTGCGAAAGAGTGTGAATGTGGGTGTGTGTTTGGTTAGATATGAAGCGAGAAAATATTTCATTCTGAATGCCGTCGAAGCAGATAAATAGTGATAGCATGTGTGATTCACTGCATTTGTGTATTAAATTTAAAGAAATTCTGAGAGAGAGAGAGAGAGAGAGAGAGAGAGAGAGAGAGAGAGAGAGAGAGAGAGAGAGAGAGAGAGAGAGAAAAGTAATAACACTCTATATTAATCATAATCATAAATAATTAATAAAACTATGATAAACAGTGTAAGCATAATAAATAATAATTAAGTAAAGTTGCCTAGTAATAATATTACAAGGATATTAAAAATAAAAACCAAATAAATACAATCAAAAATCCCTTACATTAATACTTGTCCATCCATGGTCCCACAAGCCAGGAGAGGAGAAAGAGGATGAGAATCAATAGCTAAGAGATTCTCATCATGGAAGCTGATATGAGGACAAGGAGGAGCAGCCCCACCTTGACTCATTGTATTCAGTTATGAATGAGAGAGCAGCTGCTGCACTAGCACCTGTTTACTTAGACTGTCAAAACCTTCAAAAAAGAAAGGAGGAGTTATATTATATTACAGCTTTACCCTATGGAGTATGGACATTGATTTCACAAAATAATTGCAAAGTAATAGCTACCATTAAACACACACACACACACACACACACACACGCACACGCACACACACACACACACGCACGCACACACACACACACACGCACGCACACACACACACACATACAACTAAAAATAAGGTTTGGTTTATACAAGTACTTTATGCATCAGAACAAGCTCATTCTTATACACAGCATACATACAATGCACAATTATATTAGGCAGCTATGTGTCACATGTTCTCTTTATAACTTGATGAATGAAAACCTGTAATGACTAATCACATGCCTGCATTTTATTGAATAGGACAGTAATAGTATTAATAAACAGTTATAAGAACCATATAACCAGTAATAATAATAGTAACAGCATAACTAATGTGAGTGCTCAAATAAAATTATTATGTAAGAAACAATAATCCTTACTATTACAGCCACATGCATAATGTAGACGGGTTCAGGGCTACCAAGTCTGATTTATGGAATGCCACACGTTAACAGAATCTTTAAGATCGTTATCAATGGCTGAAGACTGGCTCCAACAAGCAGGCCATTGTTCCTCCTTCCACGGTATTGTAAGCATCAGGTGAATGTCAGCTGATGCAGGCTGCCAGCTGTGTGTTTTATTAGGCATTGCAACATTTGACAAACACGGCCTGAGAAAACGAAGCATAATTCATTAGGCCAATAATAGTAAATGTGACCAGTAAGCAAACTGAGACACATTATGTGCATTGTGGGGGCTATCTGTTAGAAACAGAGACGGGTTGTGTAACAAGAACAGCTGTATGTTCAAGGTTATACAAAGTTCATTCAAATGCATTACATAATGTACACTAGGCCCGTGTCTTGTGGCTCTGTCATTCTATGCCCATATAAGGAGATTCACGAGGTGTATTGCGCAATAAACGCGCGGTTGCTAATTCCCTCTAAGGAATTTGATTGGTCAGTACAATCCGCATACCTCCTGACCGCATACTCGCCGCCGACGATCGAGATATCCCGCCATTTTCCACGCCATTGTTGCCCGGCAAGGTCAGTGAAGACGCGCCGTCTTAAGCTTTTAGCCTTTTCCTATGAGCTTAACACCATCAAAACTACACCAAGAGAACCCTAGGACATTGGAGAACCTATTACAAGTGACCAGAAGCGTATCTGGGAGCTTTTTACAGTAGAAAAAGGAGAGCGTCCCGTCTTTGCCAGTAGTCAGCTGATCCCATCCATCTCCCCCACCAGGCTGAGTAAACAATCTTGGACCAAAAAACACCACCAAAACACACTTTACTCAACTCCCCTCTCATCACTAAAGGCTTTTCCTCATCTCTACGAAGGACAGAGGCAGAAGAAGGGATGGATCACACTTCAGCTTTCACGGGGTTTTACTCGCCTCTAGCTACAGCCATACCGCCCAGTCTTCGCTATCCTTATCCACCCACCCCATCCAGTACACCAGCAAGGAGTGTTGGAGGAGGAGGTGCACCTACTCCTTCTGGAGGTATCCTCTATTATCCACAACAGCCTCTATCTTCCCCATCCGTCATTGAGAAGGGCAACCAATCTGTACAGGAGGGGCAAGCAAGGTGAGAAAGAGAAGGCTAGGGAAAGAGAGATAGCAAAGCTAGGTATAGATGGCAAAACTTAGTTTAAAACCTTTAAAAGTTATTTATTATAGAGAGAGAGAGAGATTGTTACTTTGGTACTTTTTAAAATATACAAACGAAAACTGAAATTCGAAATTTTTAGTTGTCATTTACAAAAACTTTCTTTTCTCACTCTCAGTGCTACAACAAGTCAAGATGATGAGGATGAGTCACAGAAGAGACCGCCTCTTGACAAGAAGCTCAGACGACAGATTGCCAATTCAAACGAGAGGCGAAGAATGCAAAGTATCAATTCTGGTTTCCACACGCTCCGAATGCTAATGCCACATCTACAGGGAGAGAAACTCAGCAAAGTAAGTCAAAGAAAACAAACCAATAACTGTTCTTCACTTACATAAATTAGTAGTACTCTCTTACTAGTATTACATTTTCTAGCAAAATTATTTCTGTTTCACACCTTCCTTTAACTTTCTTTTTATTTGTAATTTCTCCTCTCTCTCTCTAGGCCTCAGTTTTACAACATGCAGCTGAGCACATCTTCCGTCTAAATCAAGACAGAGACAGGCTCATACAGCAAAACACCACGATGAGAATGATGCTCACCAAATACTGGACAAACGATTCTAAAGGAGGCGATGGAAAGATGACAGAGGGAGGGAAAGTCAGCAAAGCCACGAGCCCCATTACAGTCGATGAGGAGAAAGCAACTGCTAAAGAGCTCGAACAAATATTTACAGCAGAGCACGAGCTGCAGATGATAAAGGAAGAGCTACAGCGTGAGAGGACTTTGAGGATCCAGGCAGAGACAAAGAACCGTCTACTGCAAGAGAGGATGGGCTCCGGAGAAGATTCTGAAGAAGGATCGGACGTAGAGTTCTATAGCAGTCGCAAGAAGTCCCGAATCACCCCAGAACCACGCAACAACCTTGATATAATAGTACGGGCCATCCGTCAGATTGAAGGAGATGCATTCAGTCGATCAACTACTCCTACGTGTTCAACTCAAGAAAGAGCAGCACTTTTCGTACCCATCACTACATCTAATGATACGTCAAGAGTGAGTCCTGTTACACCAGTGTTTGCTTCATGATATCAAATGACTCTCATTTTCCTTTCCTTCCCACTTCTCTCTCTATAGCTCTAGCTCTCTTTCCTTATCAATTCTTCTCATCATCATAATAATTGTTATCATTATCTCGCACTCACTCAGTAGCAGCTGTACCATAATATGCCTACATATACACACACCCACACAGTATAATTATGCAACAACAAACATTTGATTATAGAAATAAAAAGAACTTGATTGATTCCCCCTCTCTCTCTTTATTTCTTTCCTCTTCTTTTGACCATTGTAATTATTCTTTCTTTTGAGATGATTATAGACCAGATTGATTATGTTAAAAAATAATTATGATGACTCTTTAATAATAATAATAATTTCAAAAAACAATCATAAAATAATTGTTCAATATTAATGCAAGCGATCACAACATAACAACTACACAATAACCTTTCACTCATTCAGTGGAACAATCCCTTACAATCCACTTCTGTCTATTGCTATTCAATCAATCTTTCACATGCAATAGCCAGTATAGTAAGAAGTCTAATAGTATTATTCACTGAAATACAAGTATAGACAATGACTGGATTACAAAGAACTCAACCATACAGTGGTAGCATTCTAAACTACAATGTGTACCATTGTTCAATAGTTGATTCATGTTAAATGAAGTACAGACAATAATCATACATAAGATTAGTATATGACACACATGGATGTTATCATACTGAAAACAGCCAGCACATTCATACTATATTATCCATAGCTCATCATAACAAAGTCAGCTGGTGTATGGATTACAATGAAAACTGCACACAAAAGCTATTCCTTAACATCATTCAAAGGCTGAGGGGCTGATGCAATTGAAATATGAGAATTATGAGTATTCCCAGAGGGAGAGAGAAATTCAAAATTGACTCAATGAATGTGGGTAGAGGAGCAGGAAGTGCTGGTTATAGCAGCTGTTTGGGGATTTGTTCTTATGTACACAGAGAGGGGCTCTGTATTTACTATTAGGAGAACCCTTAGGAGGACCATGAGCTCCACTTACCACACACATACACACACAGAGTGAAATACATAAAACTCAAGACAATCTTTCATACTAGGAAGAACTATTAGAATATTATAAGAGCATGATGCAGGGGGCACAATTGTTCCCAAAATGGATTCCCACTTCCAAACATAAGGGAGGAAATTAGATTGAGATGAGACATCACAGCAAAACAGTGACCGAGACAGGCAGGCTCCATCAACATAGCCAATGGAGGAATTTGTATGCAGAGGGTATTATCATGATCAGGTAATTTAGCAATCGGTGAAGGAAGGGTGTGTCTTCATTATTTAATAAAAGTCCTGATCAGATGCTATCACTATTGATGTAATAATGATGATGAAGCAACAAGTTTACAATGCAAACAGTAGGAGAGATTTCATTTTAGAAAGAGGATAGCGATGACACCTAGTTCAAACTTTAAAATACAAATAGGCTGGAATAGTTTGGATAAGGGACAATGGGTCTTGAAATTAGTACTTTTTTGAATTCTTAAAAACAACCAATAAACAGCATTTATAGTACAATTATCTCTAAAAGGCTCTTTAAATCACAATGAAAAGCATTCATTATCTGGCCATCATAGTATCCTTGATGATCTAATGCCAACATTATGAGTCATAATGTAGTATGGGATACTCATAAATGTAGTAAACTGTAATGCATAGTAGTGAGCCAAAGTGTATGGCATTACTCATTAATACCTCAAGGTTATAA

>est_CL2932648

GTTGCCCGGCAAGGTCAGTGAAGACGCGCCGTCTTAAGCTTTTAGCCTTTTCCTATGAGCTTAACACCATCAAAACTGCACCAAGAGAACCCTAGGACATTGGAGAACCTATTACAAGTGACCAGAAGCGTATCTGGGAGCTTTTTACAGTAGAAAAAGGAGAGCGTCCCGTCTTTGCCAGTAGTCAGCTGATCCCATCCATCTCCCCCACCAGGCTGAGTAAACAATCTTGGACCAAAAAACACCACCAAAACACACTTTACTCAACTCCCCTCTCGTCACTAAAGGCTTTTCCTCATCCTACGAAGGACAGAGGCAGAAGAAGGGATGGATCACACTTCAGCTTTCACGGGGTTTTACTCGCCTCTAGCTACAGCCATACCGCCCAGTCTTCGCTATCCTTATCCACCCACCCCATCCAGTACACCAGCAAGGAGTGTTGGAGGAGGAGGTGCACCTACTCCTTCTGGAGGTATCCTCTATTATCCACAACAGCCTCTATCTTCCCCATCCGTCATTGAGAAGGGCAACCAATCTGTACAGGAGGGGCAAGCAAGTGCTACAACAAGTCAAGATGATGAGGATGAGTCACAGAAGAGACCGCCTCTTGACAAGAAGCTCAGACGACAGATTGCTAATTCAAACGAGAGGCGAAGAATGCAAAGTATCAATTTTGGTTTCCACACGCTCCGAATGCTAATGCCACATCTACAGGGAGAGAAACTCAGCAAAGCCTCAGTTTTACAACATGCAGCTGAGCACATCTTCCGTCTAAATCAAGACAGAGACAGGCTCATACAGCAAAACA

>EST traduction

MDHTSAFTGFYSPLATAIPPSLRYPYPPTPSSTPARSVGGGGAPTPSGGILYYPQQPLSSPSVIEKGNQSVQEGQASATTSQDDEDESQKRPPLDKKLRRQIANSNERRRMQSINFGFHTLRMLMPHLQGEKLSKASVLQHAAEHIFRLNQDRDRLIQQN

>genscan_predicted_peptide

XFSSSLRRTEAEEGMDHTSAFTGFYSPLATAIPPSLRYPYPPTPSSTPARSVGGGGAPTP

SGGILYYPQQPLSSPSVIEKGNQSVQEGQASATTSQDDEDESQKRPPLDKKLRRQIANSN

ERRRMQSINSGFHTLRMLMPHLQGEKLSKASVLQHAAEHIFRLNQDRDRLIQQNTTMRMM

LTKYWTNDSKGGDGKMTEGGKVSKATSPITVDEEKATAKELEQIFTAEHELQMIKEELQR

ERTLRIQAETKNRLLQERMGSGEDSEEGSDVEFYSSRKKSRITPEPRNNLDIIVRAIRQI

EGDAFSRSTTPTCSTQERAALFVPITTSNDTSRVSPVTPVFAS

>geneid_v1.2_predicted_protein_1|319_AA

MDHTSAFTGFYSPLATAIPPSLRYPYPPTPSSTPARSVGGGGAPTPSGGILYYPQQPLSS

PSVIEKGNQSVQEGQASATTSQDDEDESQKRPPLDKKLRRQIANSNERRRMQSINSGFHT

LRMLMPHLQGEKLSKASVLQHAAEHIFRLNQDRDRLIQQNTTMRMMLTKYWTNDSKGGDG

KMTEGGKVSKATSPITVDEEKATAKELEQIFTAEHELQMIKEELQRERTLRIQAETKNRL

LQERMGSGEDSEEGSDVEFYSSRKKSRITPEPRNNLDIIVRAIRQIEGDAFSRSTTPTCS

TQERAALFVPITTSNDTSR

**>Amq3**

GGAAATCACTCCCAATTTTTAATACATTTTCTGACACTAATTGATGTTATCTGATCTTCATTGCATGAGATCTAATTTGCAACTGGTGAATAATAAACTCTATCAACCAATAGATATAAATCTAGATCTATGTCAGTAGTACTAGCACTAACATTACTGACTTGATATTATTATTTTAGACACAGACACCTTTTTGGAAACTTGATGCAGCCTGATCAAATTTGTAGGTAATCATATCATGTCATTAAATAGTGTTTCACTAAACAGATCTAGCCTTTTTACTATCTTACTATTGGAACAAAAGAAAAAAGGCAGTTCTGTAGATCTATAAAAAATCCAGGACCTTCCGCGAGGGCCCACGGGACTTTTGTATAAAAAGACGTGACTGGTGCGGGATCAAGGCTGGCTGGTTGACCGTAAAACCTAACAAAAGACTACTGTCACTGAGAGAGGCCCTGGAAGTTTTGAGCAGCTCCAAAGAAAGAGAATTTCAAAGATTCAAAAAGAGAAAGGGCAGCCGTTACACTAGATCTACAGCTAGGAGGAACTGAACTGTACCTGTTAGCCTGATCATTTGGATACAAGATCCAGTAACCCGAGCAGATATAAATAGAGAAGTCTCAAATTTAAAGTAAGTCGTGCATTGAAGAAGCTTATGGTTTTACAGGAGAAAGCTCAACAGAGCTGTAGCCCTCCTTTGCAAGGCCAGTCTCTTGTGGATCTAGACTAGTCAGTAGTATTGAGGTTTTTAGACAAAAGGTTTTTGTAGTGTTAAGATATCATGTTTTTTTTGTCAATGTCCGGTAGGCTTGCTTAAATGTTGTCCTTTTCTTCATTCTCTGCAGGTATTTTTAAAGCAAGCAACATGGCATCCTTAGTAGAATCAGGAGCAGAGCTCCTGCCTCACTTAGACGAAGAGAAGATGCAGTACATGGCTTTCACTGAAGCGATATCATTCGCGGAACAGTTCGAGTCCCCTCTATACGACCTTCATGAAGAGACGTTTCGAGAGCTCTACCTTCGCCCTTCTCCTCCACTATCTCCCGACGACTGCGAGCCGTCTTGTAGCCAATCACCAGTAGAAGAGACTCCAACGAACAGTCAACCGAACCAAGAGCAGGCGAGAGTGCAGGACAACATCTCCACTGATGCTATGGCTGACTCGTTGATATCCAACATCATGGAGTATGAACAGATCATGGATGCGCTTCAAGAAGCCGAATTTGCTCTTTCCTCTGGCTCTAGTCAGCCTGCTGAAGACTTACTGATACAGGATTGCATGTGGAGTGCAAGCAGCAGTGATGATAATAGAACAAACTCGGGAGGGACTGATAAGAACAGTACTGATAGCTCCTCGACATCTCCAGCAACTGGTACAGAACCTTCTTCTTCATCTGCTGAGTGCTGTGTCGTAAAACCTTCTGCTGTCTTTCCCAACCTACATCATGCGGCACCACAAACTCTACGAGTACCTAACAATACTCCTGTAGTTCTCTCCAATAGACTCCAATCTATACAATCATCATCAGAATCAGGTAAGAAATTGTAATAATAACAATAATAATAATGTACAGTACAGTAGTGTATCTCTGTGTGTGGAGTAGGACCATGGTGTATTGTGAATCGTTTGAGAGAGAAGAGAGAGAGAGAGAGATGTGGACAAGACAGCTTAGCTTTGTGGATAAGATGGGTGTGTGTCAGTTACAGACACTTTATGCAAGTCATTCACTGTAGATTAATATATTATTTATGATTAAGAAGAATTGTATAGTATCTTAATCTTATAATGTATAACCTCAGCCTAGACAGTTTCAACCTCACTTCAGTGATCTATACACACACAGTGTCCAGTGTGCTACATGTAAGCTGATCATTAATGATGTTATGTATAGTTATGTAATGGATGGGGGTTCTTCAAGATTAAGAGTGCTTATGCCTCTCAAAGAATGAAACTTGTTTTAAAGAGAGAGAGAGTGAGAGAGAGACTCTTGAACTTTAAGGAGATCATTTATTGCCAGTTCTCCTATTAAACAATAATATTGAGTCCCCAAAGCCTTCAGTTATGACAACCTATTACAACTCCCCACCTCCTTTCCCCCTGTAAACCTGTTGATCCCTAAGACCTACCTCTTTCAATAGAATGACCCCATTACCAATGGCTATCCATTATACACAAGTACATGTATCCATTCATATCCTATAGCAGTTCTCCTGCCCTCAAAGCCCTCCTGCTAATGGCTGGCCTAGTTTTATGACAATACCTTGTGACTATTTATCACATTATTATGTTCAAGTGTTGAATGATAGGACAGATATAGCAACTCCTCCCAGTTCTGTTGCTTCCTTTTCATAGAGTCAACTTGTAAGGGCAGTCGACATTGTATTACATGAATTATTGTCATTGTTACCATCATGTAATGGCATTGGATGAAAAACAAGTTTAAAATAGAAATTTTAAAATTATTAAGTAACCTCTTGACTAACTATACCTAATACTCTGATACTTTCTTTTTAATTAATTGTTCTCTTCTCTCTCTTTTCTCCAGAGGAAGAAATTGATGTAGTAACGGTAGATCATAATCGCTCGGACTCACACGAAAGCGACGACCCACCAAAACGACAATCCTCCATTCTACAGACGCAGCAGCAACACTCGAAACCCGTCAAACCGGTTTCAAACGTGCAGCACGGCCAATCAAAAACAACCAAGAAGACAGCAACTCCGACGACACAGGCTCGAAGCGTCCAATCTCCAGCAATAGGAGGAGGAAATAAAAGCACGGCAAGAAAGAAGAGAGCTGGGAAGAGACACTCTCGACTGGGTTCTAATGACGGAGGGAATGAAAGTGATGAGGAAGCACGGAGAGCATCACACAACGTCTTGGAAAGAAAGAGACGGAACGATCTTAAAAACAGTTTCGATATTCTACGGACTGGGATACCCGATTTAGAAGAAAATATTCGTGCACCGAAAGTCGTGATATTAAGAAAAGCAGTTGAATACATTAAATTCTTGCAAGTTAACGACAGAAAGATTGAATGTGATTGGAGCAGAGAACAGAAACGATACAACAAGCTCCAGGAGAAGCTCTTGCATTTGAAGAAGAAACAGATCGTGACCATATAACAGAATTAGATGCTCTTCTTTTTGGAGAGGAGTTTTTGACCAAGCAATCAAAACCTCGAATCAATTTTCCCAATTGCTATGCTTACTAGGAGTTAATTTCATTGTGGATTTTCACTAACTTTTATCTTTCTCTTTCTCTAACCTCACCACCAACACTTCCTCTTTCACTCTCCATTCACTCTCTCTCTCTACTTCAGCCACCAATCACAACAGCACCTCTAGGTATCAATTTATTGTGTTCTTGCAACGTTTTATGCTGCCACGCCCTCTCTCTCTCCACTCTCTCGTATCACTTTTAAAGTTTTAAAAATTTTTACGCTTGAACGGAAAGAACAATTCCTTCACTTCGCCCTCCCCCCTCCTCTCCCACTGTTGCTGTTTCAACGTTGTAAAAATACATTTTAGTAACAATTATTAACACACACACACTCCTACCTTGTATTATATTATTATTATTATTATTTGTCTGTAACTTGTAATTTAGACTTTAAAGTCACTTTTTGAACATATTTATTCAAATTTTACTTTAAATTTTAATTTTAAAATGATCAATTGTGTGATCTACATAGCGTCTATCGAATCAAATGGGACTGTACTAAAATGTCCCAATTTTAGCTGTTGTCAATGAAGGAGCTTTCAAATGTTACATAGTCCCAAAAATCACTGATGTGGAGCAGAGATTTAAAAAACTGATCGTCTTCTGAAATAAAGACATCAATTTTCACTTTTGAAATAAAGAAATTTATCTTTTCCTTTGTCCAGTAGAGTGAATATACCAGATTGCTAAACAGGAAATTCACTGCAAATCTAATTATGATACCACCAAATACAATGAAAATTTCTAATGTCTATCATAATATGGTAAATTGTATCTATACTACCCACCACTTAGTAACTTAATCTTACTATTAATAATTATTGTGCAATTGCTAATCTTGTCAGTTGTGGTGTCCTCTCAAGATTATGATATAACTGCTAGCAATAACATAAGGTTTGACCTTTAGCAGTATACTGTATCATATATACTGGTATAGAGTTACATGATGTAGCTCTACTGCTAGATATTGTGAAATAATACAATCAGTAATAATAATAATAAATATAATTATAATAAGGACTGAATGACAGTGCAGTATCTAACTACATGTATGTAAGAGTAGAGGTTGGAATGATCTATGTAACTCCACCCAACTAGATCACCAAACCTATTACAACAGGAATTGTGTTCTCAATACACTGAATGATAGTTCTCTGGTCATTCATAGATTAATAATATTAACTTATGAGTTTTGGTAAGTAAAATGAGCCACTCAGAACAGTACAGACTACAATAATAATAATGTTGATAATAATATCAACTGAGTGATTGTTCTCTGGACCAATTAGAAACTATACAATATTATGACTTAAGAGTAAAATGAAGCACTCACATCCTTTTCTCTGAACAATACTACTCTACAGTCTATCACTGTTGTTTAACTCAATACATCATTGTGGATTTCAGCAGCTATCATTGTATCAAGTCATTCAGTGATCATTGTGTGATCCGGTTAGTCAAAGCCACCAAATCTAAAGGCAAGAAAAAATAATAAAAATAAGTGCCTCAACCTGGGACATGATACTGAACAATAGTTACATGTTAGGCTACACCTCAACATGAAAACTCATGTAACTAGTTTATATAATAAACAATAGACACTGAGTAATATACTATGGAACAATAATAATAATAATAACTAGAAATGATGATGGTTGAAGCTTGCAAGCCAAGAACTTACAAATTCAAACCCGTTCAGTATTGGAAACAGAATTGCAGCACTTTCTGCATTAGTTATACAACCAACCCAGGGTTAATAATTAGCTACAGCTACTTCTTAAGTAGTACAATAGATGCTAGGTATACTTAAACCAGTAAGTCAAAAGTTTTATAGACAGGAAATGCCAGTTGAGGTGAATGCAATGGAGTTAAGCTACAGTTACTTATCTACTGGTATAGCTGTGTTTCAAGAATGGTCTGCCTTAGTTCCATTGAATTAAGAATTATAAGTTAAGCAATACAATAGTATCACTTTAGTGTACAGTACAGTACGTTTAATGTTGAAGAACTGGAGGAAACTTATTGGTAGGAGGAACAGATTCCTTTATTAAAGTATAGTCTCTCTTAATGGACACTCTCTATAGCGTACACCTCCATTTAACGGACAGCACCTAGTGTACCAATTACATTCCTTACTGTATAAAATAACCTCCACATAAAGGACACCTCTGTATAACAGAGACACAGTGTCCATTATAGCAAGGCTAGTCTCTTAACACAACTGAGTGTGGAGTGTATATACATGTACATGTATGTATACAGTGTCTCCTTCACACCTCATTAGTCAGTATATGTATGTGTACATTTGAATTTAAAAGACCCAGTTAGTCTACAGAGTAAGAAGGACAAGAGATGACAAGGTATGAAATACTATTATGTAATGCTTAGTAACACTGTAACCAGATTGAGGAAGGACTATATACCATTAAGATAGGGAGTAGCATTACATGTTGCTTCATACTATCACAGTATCATTCTAGTGTATTCTCCCCAGCTTACTTTCAAAACAGAGCAGCTGCAATAATATGGAGAGGCTGCATATCCATAGAAACCTTATGATATGAGAAAGGTCAGGGCTGGAGACTATCATAGCTACTACACAATGCAATAACAGCTACCCAANCACAGAATAAGATTCACAGAGAGGGACTACCTGAGAGAGAGAGAGAGAGAGAGAGAGAGAGAGAGAGAGAGAGAGAGAGAGAGAGAGAGAGACTGTTGCACAGGTTTATATTTAAAAAAGCAATAACACAAAAATGAGTGTTTGTCATTTTGTATATTAACTAATCAATAAACAATAAACATGTATGTTTACATGTATACATATCGCCAAGGGAACGCGGTAGACATCAAAGAAAAGATAAAAGGTCTGTGAATCTTTTCAAAAATGAAGAACATATTGAATAGTGTAGAACATCCAAAAAAGGATAAAACGAGAAAGAGAAGAGAGAGAGAGCAACAGCAGGTGAACTTGTTTGTGTGGAATGGATGAAATGTCTCACTGATGATGATTGGGAGATGATCCCGTGGGAATACACTCAGTGACTATTAGAAAATAATTAGGACACAAAGAGCAACCGCCCACGCACTAACAGACAAATAGACACACTGGCCATGAATTTGAAAAATTTATGACGACAGAAATGACAGAGAAATTAATGTAATACAGACTAGCCTCTATATAATGGACACTGTGTCTCTGTTATACAGAGGTGTCCTTTATGTGGAGGTTATTTTATACAGTAAGGAATGTAATTGGTACACTAGGTGCTGTCCGTTAAATGGAGGTGTACGCTATAGAGAGTGTCCATTAAGAGAGACTATACTGTACATGTACTGTACCTTCCAATGACAGCCCCTTCAGCTGTAATTAAACTGATGCATTTACGAGCATCTTGAGAACCAGCTAGTTCCGAGGCTCCGCCCACACTTGAGGAAGGGGCAGGGTCAAGTGGTATGAGACGAACACACGGAGGCCACTCACTCATTGGAGGAGGACGATCAGGCAATGAATCATCATCTGAGGGGGAGGGGGAGGGGGAGGAGGAGGGGGAGGGGGAGGGAGGGAGAGGAGAAAGGGAGGGGGAGGGGGAGGGGGAGAGAGGGAGAGAGAGAGGGAGAGATTTCTTTTTNTTCTTTTTTTTTTGTTTGTTTGTCTCCCCCCTCCTCCCTCTTCTTTTCCTTCATCTCCCTCCTTCTTTTCTCCTTTTCTTTTCTCCTTTCTTCATTTTTTTCTTTAGTTCGCCCCTTTCTTATTAACCTTTATACATCCCTCTATATTTTTTTCCTTATTTATCCTCTTCCCTTTTGTTCCTTCACTTTCAATCATCTCTCCTTTTTCATTATTCTATTTGATTCTTCACGATACCTACGCTCATTTCTATTTTACTTTTATTTTACAACTACCTAATTCTTCTCCCTCCCTCATTTCTCATCTTGCAAGCTCCTCTATCACTCTCTATCTCTCTTTTCCTCATTGCTCTCCTCTATTATCTATTCCTCTATCCCGTC

>est_CL2942026.2

ACGGACTGGGATACCAGATTTAGAAGAAAATATCCGTGCACCGAAAGTCGTGATATTAAGAAAGGCAGTTGAATACATCAAATTCTTGCAAGTTAACGACAGAAAGATTGAATGTGATTGGAGCAGAGAACAGAAACGATACAACAAGCTCCAGGAGAAGCTCTTGCATTTGAAGAAGAAACAGATCGTGACCATATAACAGAATTAGATGCTCTTCCTTTGGAGAGGAGTTTTTGACCAAGCAATCAAAACCTCGAATCAATTTTCCCAATTACTATGCTACTAGGAGTTAATTTCATTGTGGATTTTGACTTTTATCTTTCTCTTTCTCTAACCTCACCACCAACACTTCCTCTTTCACTCTCCATTCACTCTCTCTCTCTTTCTCTACTTCAGCCACCAATCACAACAACAGCACTTCTAGGTATCAATTTCTTGTGTTCTTGCAACGTTCTATGCTGCCACACCCTTTCTCTCCACTCTCTGGTATCACTTTTAAAGTTTTAAAAATTTTTACGCTTGAACGGAAAGAACAATTCCTTCATTTCGCCCTCCCACCTCCTCTCCCACTGTTGCTGTTTCAACGTTGTAAAAATACATTTTAGTAACAATTAACACACACACACTCCTGCCTTGTATTATATTATTATTATTATTTGTCTGTAACTTGTAATTTAGACTTTAAAGTCACTTTTTGAACATATTTATTCAAATTTTCCC

>EST traduction

RTGIPDLEENIRAPKVVILRKAVEYIKFLQVNDRKIECDWSREQKRYNKLQEKLLHLKKKQIVTI

>genscan_predicted_peptide

MASLVESGAELLPHLDEEKMQYMAFTEAISFAEQFESPLYDLHEETFRELYLRPSPPLSP

DDCEPSCSQSPVEETPTNSQPNQEQARVQDNISTDAMADSLISNIMEYEQIMDALQEAEF

ALSSGSSQPAEDLLIQDCMWSASSSDDNRTNSGGTDKNSTDSSSTSPATGTEPSSSSAEC

CVVKPSAVFPNLHHAAPQTLRVPNNTPVVLSNRLQSIQSSSESEEEIDVVTVDHNRSDSH

ESDDPPKRQSSILQTQQQHSKPVKPVSNVQHGQSKTTKKTATPTTQARSVQSPAIGGGNK

STARKKRAGKRHSRLGSNDGGNESDEEARRASHNVLERKRRNDLKNSFDILRTGIPDLEE

NIRAPKVVILRKAVEYIKFLQVNDRKIECDWSREQKRYNKLQEKLLHLKKKQIVTI*

>geneid_v1.2_predicted_protein_1|398_AA

MQYMAFTEAISFAEQFESPLYDLHEETFRELYLRPSPPLSPDDCEPSCSQSPVEETPTNS

QPNQEQARVQDNISTDAMADSLISNIMEYEQIMDALQEAEFALSSGSSQPAEDLLIQDCM

WSASSSDDNRTNSGGTDKNSTDSSSTSPATGTEPSSSSAECCVVKPSAVFPNLHHAAPQT

LRVPNNTPVVLSNRLQSIQSSSESEEEIDVVTVDHNRSDSHESDDPPKRQSSILQTQQQH

SKPVKPVSNVQHGQSKTTKKTATPTTQARSVQSPAIGGGNKSTARKKRAGKRHSRLGSND

GGNESDEEARRASHNVLERKRRNDLKNSFDILRTGIPDLEENIRAPKVVILRKAVEYIKF

LQVNDRKIECDWSREQKRYNKLQEKLLHLKKKQIVTI*

**>Amq4**

TCATAAAAGTAAAGAGCATTGCACTGCATGAGTATCTGATAGTAGTAGGGGCCACTTCATTAACTCAAATTAGCTAACCAGTCCAGCTGTGCAGTGTTATATAACTTTATTCATACTATAATATGTGTATATATGCCTACCACAGATATTGGTTTATTGTATAGTAAAATGGGAGCACTCCAGATTGATTGTCTGCATTCTTAGCTAGTTTATCCCATAAATGAGCATGATAGTCTTCT

TGATATTTAAATCAATTGATTGATAATCTTTTTTATGTGTATCAAATAACTGTCACTCAGTAAAGCGTCTCATCTAATTAAAAAAATCAAGCATTATACTGTTATTGCTTTTAGCAGAGTATTGATTGATAGTAGAAATCCTTATATCTGTTACCCATAGGGAGACACCAGTGGAGATTACAAGAGAGTTTTGTTAGCTTTGGCTGGAGAGTACTAACTATAGGAAACAGTTTTGTCAAATTATTATATCAGTTAATTTTATTTTTGTGTAGTTTGATTTTTATATAATACTTTTAGTGCTCTGTAGGAATTTTAATATGCGTGGGCGTAATCTTGTGGAAAGCTGTTAAATTAATACTAATATGTTCTTTCCCTTCATTTCACAGCCATTAATCCCAATCTTGTACCATAATAATTTGATTTGTAAAATTAATTTTTTGCAAATCTTCAAAATTTGCAAAATCCATGAAATTTACAGCCCTTAATTTTGGGCTATGCAGTACTTGCGTGTTTTGGTCTTAAAGAAGATAAGGTTTATTTCTCATTAATAAATTCCAAAGAGTTTTTCTCACTACTTGTACTAACATATATATGGTACACAAAAATAAAACACCAGAAATTTAGAAATGACTACAATGTATTTTTAACCATTTAATTAGTTTTCATGGAGTTGCTGCTATTTTTGTTTTAAGCACTTGTTTCTCTGTCAGCCAGTCAGTTGTGTTTCATTAGTAAGGAGTAAATGAAGTAGTTCTATCCTCCACTAAATTAAACTCTGGATGCATTATGTACCATTGACCATTGACGGGCACTGGTGAGATGCAGTTAATTATGAGTGCAGGATATTTGCGATTATCGCAGCTGGTTCTTTCTCTTGCAGGAGTAATTATGATTTGATTTTGTGAAAGACAGTGAGTATAGCAGAGAGGTTCGTCAATTTATTTTTTTTAATTGTTACTATAAAGTTGTATTTAGAGGCTTTGTGTGGAATAACTTAAAGACCTGTAGGCGTAGTGTGCTACTGGAAAATATTGTTAATTGAATATATATGTATTCTAATTAAAACTGGTGCTTTTTGGACTCTTGATAAAGGAGAGAATCCAGTTTAATGTCATGAGAATGATCGATAACCCATGTATGAAGCTGAATGTTATCAAATACATGATCTACAAGCCAGGTAGTGCAGCTACAAGAAACACCTGTAAAACAATTGCAGTAATTTTGGTATAAGAATATCTCTGGTGCCCGCCAGCAGCGCACTGAAGGACAGCAAGTCAGCAAGATTATTCCCGGGAGGATGGATGTGTACTGGGCATTGCCTAAAGTGCAAGTGGATCGTCTGGATGAAGACGAAGATGACAATAGGCCGACAGAAGCAGCCGTTGTTATTTACAACTGCCAGCCATCTTGTAGTTTTCACTGTGAACTGCAACAAGAGAGTCAAGAAGAAATTGGAGATAGGAAGAAAGGGAGAAAGAAAGGGAAAAAGAAAGGGAGGAAGAATAAAACTAAAAAGGTGAGTGAGGAATCTGCAGCCTTACTGAGACAGCAGAAAAATTCTAAAGAGAGAAGCAGAATCAAGAGCATTGGGCAGAAATACAATGAACTGAGAAGGATTCTTGGCTTTGACCTTACTAAAAAACGGTATTGCAAGCACAAAATTCTTAAAGCTGCAATAGAGTACATTACAAAACTGCAAGAGCTACTATGTGATGAAACAATCCACTCTAGTGGGAGCAGTAGCAGTAGTACTGAGTCCTCACCAGTTCAGTCTTGTCTCTTGCCTCCTGGCCCTCTTCATCATGCTAATCCTGGGCTTAGACCAGTCCCTTTTTCTTATCATGGTATTGGAAGGTATCCATCCTTGTCTTCCCCTCCTCATTGTTTACTAATGGAGTTCTGTAATAATAATAATAATAATAATGGAGGACAGAGGAAGATGGAGTTCCCTTGCAATCCTGGTGCTATTATGCCTCCTGCTGGATCTCCAACATTCCCTTTCCGTCACGTCTTAATTAATTAAGACTTTGTTTATGTTTTTGGACTTTCATTTAATACTGATGTATGTCAGTTGCTTTTGTATCATTAATGCTTTGTTTATGTCTTTGGACTTTCATTTAATACTGATGTATACAGGGTCGTAGCCAGGAACTTGAAACCATTTTGCTGCGCGCGAAGCGCGCAGCGAAAAGTTTGACGAAAACGGGCGTGGTTGGTAGGCGTGGTATTAATTTACGCATCTCAGCCATGTGCTGACTCAGCTAAAATGGCTGTCTGTGTTTCTCGCTGTTGCCAAAATGCTAATCCTGCTTTTGCAGTGCTGTTCTTCATGACAGGAGCTAATCTAAAACTTTTTTGCAGCTGCAACAGGGGGTTCGTCCGAACCCCCCCCCCTGGCTACGGGCCTGGTATATATGCGAGTTGCTTTTGTGCCACTAATGTTTGAGTAGCCGGATCATGATTCATGAATTAATGGTCGGGCGTGGCTCAGTGGCTGAGGTACGTGTTAGTGTACTTGTGTTTGTATATCTATGGTGTAAGTGTTCTATCTAAAACGTTTAATGTGAAATCATGTCAAGTAAAAAGAGAGTAAAGCTGCTAAAGTAGCTATAAAGAGAAATAATGGCTAGTGTACAAAATGAAGAAGTAAAAAGACCTAGTGAAGGAGCAAAGTCAAAGAGGTAAATCATGTGATCGGAGACTAATCGGATATGTATACATGATACCAAATTTTTTTCAGTCAAAAAATTTTTGTCAAAATCTTAATTTCTTGTTAAATTAATTTCCACTTTATAAGCACTATATATTGGAGGGCTAAGGGAACTAGGAGATAGAGAGAAGCGAGAAACCATGTACTGATTTTTGAGCAACTTTAAAAATTTGTAAGAAATATAATTTTGATTGGAATCTGCTCAAACTTTTTATATGCATCAGGAAAAATATTAAAATAGAAATTATCTCTTTGGAATTTTCACAAAATTTATCCAGAGAGACCATTCGCCCAATTTTTTGAGCAACTTTAGAAATTCACAAAATATCTAATTTTGATCTCATAGGCAGCATGCATTCTTGTCTCTTTTAATGGGCTATTTGGTTGATGGTTGGTAATATTTGCTCTGTTCAGACTCTATACGTATGCAGTTAATGGCCTCACAAACAGAACAAAATGTTCTTTTCTCTCACATTAGACTATGACATTTATTTATTTTAAAAATTGTTGAAATTACATAAATGTCAATCAATGACTCATGCTCGCCCCCTCAATGGTTTACATTCACCTAGTAATCATTATTAATACACTATAAATGAAAGGGAGGGAGCATGAGGTACTAT

>genscan_predicted_peptide

MDVYWALPKVQVDRLDEDEDDNRPTEAAVVIYNCQPSCSFHCELQQESQEEIGDRKKGRK

KGKKKGRKNKTKKVSEESAALLRQQKNSKERSRIKSIGQKYNELRRILGFDLTKKRYCKH

KILKAAIEYITKLQELLCDETIHSSGSSSSSTESSPVQSCLLPPGPLHHANPGLRPVPFS

YHGIGRYPSLSSPPHCLLMEFCNNNNNNNGGQRKMEFPCNPGAIMPPAGSPTFPFRHVLI

N

>geneid_v1.2_predicted_protein_1|242_AA

MDVYWALPKVQVDRLDEDEDDNRPTEAAVVIYNCQPSCSFHCELQQESQEEIGDRKKGRK

KGKKKGRKNKTKKVSEESAALLRQQKNSKERSRIKSIGQKYNELRRILGFDLTKKRYCKH

KILKAAIEYITKLQELLCDETIHSSGSSSSSTESSPVQSCLLPPGPLHHANPGLRPVPFS

YHGIGRYPSLSSPPHCLLMEFCNNNNNNNGGQRKMEFPCNPGAIMPPAGSPTFPFRHVLI

N*

**>Amq5**

TAACTGCTGTTCATTGTACAGCAGAGCCTAGCACTCGTAATTCTTCTAAATGGAGTCATTATATTCAACAATACGAGAATGCTATCAGTCTGCAGGGTAATACAAGTCACACCTACACTTGTGGTGGAGAATATATGGGGGTGTTGGAATCTGATCTTGGAGTCTGGAGGGAGAGAGGAGGGATAAAGAGAGAGGAGTTCATCCATGCTAAATCAAAAGGTGTTCATTATCAGATTGTCAATGGGAAACTCTACAGAGAGAAGGACTGTCTTTTTTCGTTTAGATGCAAAGGAGTCGAGCACTTTATTTTAAATATCATCGAGGATTTACCAAATATGGAATTAATTATTAATGTATTCGACTATCCTAAAGTTAGTAATAAACAAATAATAATAATAATTGTTGTTGTTCTTTTAGTCTCATAAATATCATTCGCCATTGCCTGTGTTTTCATTCAGTAAGGTAAATTATAATAATTATTATGAATTATGATAATAGTAGTAATAATGATGATAGACGGTACATTATTGGGACATTATGTATCCTGCTTGGACGTTTTGGTCAGGAGGTCCGGCAGTGAGCGTTGAGCCAACTGGTCTTGGACGTTGGGACTTGAAACGAATATCAATTACCAAGTATTGACTAGCAATGCTAAAAAATAATAATAATAATTATTATTATCATAGATCTGCTAAGCAATGGCCATGGGATAAAAAGAAATCATTGTTATTTTTCCGTGGCTCAAGGTACTGAATGCATTATTCATGATTGTTTTATTTACATTACATTGCATGTACAATCATTTCTGTTATTTTATTCCTGTGTGTCGTATGCATGTTACGTTAGGACAAGTAGCGAGAGAGACTCTTTGATATTATTATCGAGAGATAAGCCTCATCTTGTTGATGCTGCGTATACTAAAAATCAAGCTTGGAGGTCTAGTAAAGTAATCCTTGCTTTTGCTTATCATTGTAATGCTGCTTTGATACTCTTAACTTTACAGGATACGCTAAATGCTCCACCAGCCGATGAAGTTAAGCTTGAGGAACACTGCCAATACAAGTATGACTGTTATTGATTTAAAATGTGCATGGAGCCTGTTCGTTCACACCTTGTTAGTCCCTTCAAAGTTTCAAGGGTATTATTAGCAGGGTGTTGAAACAAACATAAGCACAAATATTTTTATGACTACATCTATCATTAGGTATTTAGTTAATTTCCGTGGAGTTGCTGCTAGTTTTCGTTTTAAGCACTTGTTTCTCTGTCATTCAGTTGTGTTCCATGTCGGTAAGGAGTGGATTGAGTTCTTCTACCCTGCACTAAAACCTTGGATACATTATGTACCATTGACCACAGACACTGTTGACATACAGTTAAGTATGAGTGCAGGATATTTGTGATTTATCTGGTTCTCTCTCTCTCTCTTGCAGGGATATGATTGATTTTGTAAAAGACAACGATGACATAGCGAAGAGCATAGCAGTGAGGTTCGTCAGTGTATTTTTATAATAAAATTGTTATTAAGTTGCATTTAGAGGCTTTGAGTTTGTATGGAATAACTTAAGACCCGAAGATGTAGAGTGCTATTGGAAAAGATTGTTAATTGAATATTCTAAACTATTGCTTTGGACTGTCGATAAGGAGAGAGATTACAGTTTGATATCTTGAGAATAATCATTGACCCATGAAGCTGTATCTTATCAAAGTACATGATCTACAAGCTAGCTACAAGAAACACCTGTAAAACCCTTGCCTTGCATGCACATGTACCTTGGTATAAAAATAGTGGGCCTTTGGCGGGCTAACTGACCAGCAGCTGCTGAACAGCACTAGGAGACCACTCGGACTAGACTCAAGAACTGCAGGGGATAGATGGATCTCTACTGGGGATGGCCTAGAGTACAGGTGGATCTGGATGAAGATAATAGAAATGCCAATGCCATACCGATTGAAGCGGCTGTTGTCTTTTACAACAGCCAATCTCAATCATCTTCTTATAGTAGTTTTCCTACAGACTGTTGTGATGATGATGATGAGGAGGAGGAGGAGGATTTTGAACTGCAAGAAGAGGATCAAGAGATTGGAGCTAGTAAGAGGGGAAGGAAGAGAGGGAGGAGGAATAAAACTAAAAAGGAGAAGGAGGACTCTGAGGCCTCACAAAGAAAGCAGAAAAATTCTCAAGAGAGAAAAAGAGTGAAGAGTATAAGGCTAAAATATAATGAACTGAGAAAGGTTCTTGGCTTTGACACAACAAAGAAACTTTGCAAGCAAAAGATTCTTGACGCTGCAATAGAGTACATCACCAAACTGCAAGATATGATCAAAGATGACAGGGAAGAGGAGCCTCTTCTTCTATGTGATGAGACCATTCACTCTATCGAGAGCAGCAGCAGCGGCAGTACTGAGTGCTCATCAGTCTTGTCTCCCGCGCCTACTTCTGAGCTTAGCCCGGTCCCTTTTTCCTATTCCTATGGAATTGGACGGTGTCCTGCTTTAACTCCATCATTATGCTCCCCTCCTCAGGCTCAGTTCACACTGATGGAGTTATACAATCATGGAAGATGGAGGGCTACTCCTGAGATGGAGTTTCCCTGCAATGTCGGGGTCACTACGCCTCCTGCTACTTTTGATTCATCGTATACTATTTCACCACCTCCAACGATCCCTCTCCATCACATGATGTTCAATGAAAACTAGTTCATAAAAGAGAGTTTCAAAACTAAAGTTTAAACATGTTTTTGTATAGATATTTTCATGGTTTTCAGGTGTTTTCACTGTTTTGTGAGAAAGAAAGTTGCGTCTTTGCGAAAGACAGTGTGTTGTTTGCGTAGGCGGCTCATAAAATTATTCTTTTATTGAAGGCGTGGTTGAGGTGTGGTTGTAATTGTAAAATGGCATAGAGTGAAGACGAGATATTGAAGAGAGAGTAATGGCCAGTAAAGCTCAATCGGCAGCTGTAAAGAGAGGTAATGGCCAATGTATACTGTATAAATGATTGCTAGTAGCTATAGCTAAAAAGAAGAAGCCAAAGAGACCCAATGGAGGAGCTAAGAAACCAACATCATCACTGAAGAGGTAAGTCACGTGACGAATCACGTGATGCATATACATTCTATAGTCCTGAAGCACAGTCTGAAGATGAGTATGACACAACCGACGATGAGCAGGAAGATGCTGCTGATTATTGTAAAGGTACCGGTAGAAAATTAATATAATAATAATTATTATTATTATTGTTAGGGGGCTATCATTTAGTTGAGATTGGTGAAGTCTACAATGGTCGCTATCAGATAATTAGGAAGCTAGGCTGGGGCCATTTCTCTACCGTCTGGCTATGCAAGGATTTGAAGTATATCCAAGTAATCAAACTGCTCTATTTGTGATATTGTTTGCTAGGTGTGGGCGATTCGTTGCACTAAAAGTTGTTAAATCGGCAAAGCAATACATGGAAGCATCTCTTGACGAGATTGAGCTACTCAGAAAAGTAATGAGAACAAATATACCACTTTGACTTATCCTTTGTCTTTTTAACAGGTTGCTACTGCTAATAGAGCTAATCCTGGTTTAAAACACGTGGTTGAAATGTATGACAGTTTTAGGATATCAGGACCCCATGGCAACCGTATCCTATGAAATCCAGAGAAGGGGAAAGAGGGAGGGAGGGAGGGACTGGTTGTGGGAAAGGAATATTTTTTCTTAATTTCATTATAAATAGATATGGTAATGGTATTTGAAGTCTTAGGTTGCAATCTCCTCAGACCAATTATTAAGTATAATTACAAGGGGCTTCCTCCTTCATTTGTCAAACTAGTCACTAAACAAGTTAGACTGAACCAAAATCCTCCTTTATCCCCTCCCTCTCTCCTCAGGTATTACTAGGATTGGATTACTTACACACAGAGTGTGGTATAATTCATACCGACATTAAACCAGAGAACATATTGTTTTGCGTCAGTGATGAACATGTTAAAAGTTTAGCTAGAAATAGAGTGTCATCAAAA

>est_CL2933838

AGGATTTTGAACTGCAAGAAGAGGATCAAGAGATTGGAGCTAGTAAGAGGGGAAGGAAGAGAGGGAGGAGGAATAAAACTAAAAAGGAGAAGGAGGACTCTGAGGCCTCACAAAGAAAGCAGAAAAATTCTCAAGAGAGAAAAAGAGTGAAGAGTATAAGGCTAAAATATAATGAACTGAGAAAGGTTCTTGGCTTTGACACAACAAAGAAACTTTGCAAGCAAAAGATTCTTGACGCTGCAATAGAGTACATCACCAAACTGCAAGATATGATCAAAGATGACAGGGAAGAGGAGCCTCTTCTTCTATGTGATGAGACCATTCACTCTATCGAGAGCAGCAGCAGCGGCAGTACTGAGTGCTCATCAGTCTTGTCTCCCGCGCCTACTTCTGAGCTTAGCCCGGTCCCTTTTTCCTATTCCTATGGAATTGGACGGTGTCCTGCTTTAACTCCATCATTATGCTCCCCTCCTCAGGCTCAGTTCACACTGATGGAGTTATACAATCATGGAAGATGGAGGGCTACTCCTGAGATGGAGTTTCCCTGCAATGTCGGGGTCACTACGCCTCCTGCTACTTTTGATTCATCGTATACTATTTCACCACCTCCAACGATCCCTCTCCATCACATGATGTTCAATGAAAACTAGTTCATAAAAGAGAGTTTCAAAACTAAAGTTTAAACATGTTTTTGTATAGATATTTTCATGGTTTTCAGGTGTTTTCACTGTTTTGTGAGAAAGAAAGTTGCGTCTTCGAAAA

>EST traduction

DFELQEEDQEIGASKRGRKRGRRNKTKKEKEDSEASQRKQKNSQERKRVK

SIRLKYNELRKVLGFDTTKKLCKQKILDAAIEYITKLQDMIKDDREEEPL

LLCDETIHSIESSSSGSTECSSVLSPAPTSELSPVPFSYSYGIGRCPALT

PSLCSPPQAQFTLMELYNHGRWRATPEMEFPCNVGVTTPPATFDSSYTIS

PPPTIPLHHMMFNEN

>genscan_predicted_peptide

MDLYWGWPRVQVDLDEDNRNANAIPIEAAVVFYNSQSQSSSYSSFPTDCCDDDDEEEEED

FELQEEDQEIGASKRGRKRGRRNKTKKEKEDSEASQRKQKNSQERKRVKSIRLKYNELRK

VLGFDTTKKLCKQKILDAAIEYITKLQDMIKDDREEEPLLLCDETIHSIESSSSGSTECS

SVLSPAPTSELSPVPFSYSYGIGRCPALTPSLCSPPQAQFTLMELYNHGRWRATPEMEFP

CNVGVTTPPATFDSSYTISPPPTIPLHHMMFNEN

>geneid_v1.2_predicted_protein_1|275_AA

MDLYWGWPRVQVDLDEDNRNANAIPIEAAVVFYNSQSQSSSYSSFPTDCCDDDDEEEEED

FELQEEDQEIGASKRGRKRGRRNKTKKEKEDSEASQRKQKNSQERKRVKSIRLKYNELRK

VLGFDTTKKLCKQKILDAAIEYITKLQDMIKDDREEEPLLLCDETIHSIESSSSGSTECS

SVLSPAPTSELSPVPFSYSYGIGRCPALTPSLCSPPQAQFTLMELYNHGRWRATPEMEFP

CNVGVTTPPATFDSSYTISPPPTIPLHHMMFNEN

**>Amq6**

CAACAACACTTAGCACTTGAAGACCTAAAAGTCACCCTGGAGAGCTAATATTTGCATTTCTTAGCTCTTACTGTATCAACTGTGTAATTACTTTGTACATGTATAAATTGTGGTAATAAGCCTTTGATTGGCCTGATGTGATTATGATAGACCATGACATAATTTCATTAATCGAACATTTTTATTTTTACATGTAGGTATCTAATACAATTGTACAGCCGGATTTCATGGGCGGAGCCTCTGAATCTAACTCAAGTGATCTAAAAGGTTAGTTAATGTACATTAATGTAAGTGACACCTGGCTAGTCTATGCTATTCCATTACTGACAATGCTCTCATACATCTCTAACAATGGGTTTTATAAGGTGTTGCCTGGAGTTATTCAGTGGACTACAATTGATTAATTACTAAAGGTTACTGAGAGTGAAGTCTTATGAATATACTATACTCACTTAGCCTAAGAAAACAAGTAACAAAATGAGGGCCGGCCTATTTGTAGTATAATTGATATAGTACTGCAATAGTTGGGAATGCATTTCTGTATTGAGGTGTCGGTTTGTCAGTTTGTGGTCAGAGCCTAGCACCAACACCCTTCATTCACTGCTTCAATACAACTAAAGGGCCTGGTATATCTCTTACTCAGAATGATTGTTATTGTGGCGGTTGAAGTGACCAAAGGCTTGTGTGATCTCAGAGTTATATGTCACATGCACATTTGCTGTCATAACTATCTTTATTAGGTATAGTTTAATGTACACTTTGACAGTGCATATGAGTACAGACGTGTGCTATGTATATGAGTTCATGTCATTACTTCCACAATAATAGCGATTAGTGTGTCATTATTACCTGTGTCGGTGATGTGTATAGTCATAGCGTGTCATTATAATAAGTACATGTTAATTATTTTAGGTTAAATAGTTAGTGTGTACTGACTAATACCTTGTACACAATCATCCGGTTTGTTGATAGAAGCAAATAATGATGGAATTCTTTGCATTTTATTCACAAGACTTGTTTGTCTCTTTGTTTACTGCATGGGGAGCATGTTTGAGAATTCAAATCCTGTTTTGAAAACTCATTCCCTGTTGCTTGTCAGACTTATGCTAAAGTCTATTGTAGTCCGGACCACAATGTGGTCAGACAGACAAGAAGCTTCTGAAAAGCTCCCCTTCTCCACAAACTGATTAATATCTAGAAAATAGATTTTTCATGATAGGGAAAAGAGAATTCAAGAGCCAGGATAGATTTGTGGGAAGATAGATCAATTCAGTGTATGTGTTTTGATAAGTTTGTCTTTACTCTTGTGTCTCTGAAGGCAGATCATATTACCCCTGAGAGAGGAGGATAGTTATGGATAAGCTGACAGTGTTGTTATAACTTGTTACATTAGCCTGTAGTAGGAGTAGCTGACAAGAAGACCAACTCTTCTATCTGCTAATAATACAATTTCTGGTTTTGACCTTTTAATATCCTCTTGGCCTCTCGTGTGATACCTTGTCCTTCCTTTGGTCATGATTAGGTCTCCATGTTTTGTCCAGCTTCCTGTATTATAAGATTGAATTCTTGTTTGTAAATGACACATTGAAAGATTACTTGGTATAGTGGTATGTTAGTTCATACCCTTGTTAAACCAGATCAAAGAATGTGTGTGTGAACTTACTGTAAAGATGACCCTCAAATACTCTGTAGAGTTCTCATACATGTCATGTACTAATGTAATTTGTCAGGTCTTAAGGTCACACCATTGTACTTAGTGGCTTTGAATCTCAAAATAAACTGATTTATTTAACAATCTTAGTTTATTTAAAAATAATTCAGGGCTCTAATTGTAACATTATGATACTAGGTATAACTGCTTTGCACCCTCTGATGTGGTCGGTTGTTTTTTATTTCTTTAAATAACAATAAACTCGTCTTGCGAATATAAAAGGCATGCTAAATACGTTAGAATTACAGTTGGTAGTAGTGGACGTTCAGATTACGATCATCTTTTCATATGCTTAGGTCAGCAGAAAGTGACTAGTTGTAGAGTTGAGAAGGTTCGTGTGCCTCAGTTACATTTGAATACTAAAGGTTAGCAATTATTATGTACTAGAGTCATATGTGTATATATGTGTGTGTGTGTGTGTGTGTGTGTGTGTAGTGTCTATAAAATATGTGTCTGTGTCTGACTAACTTATGCAATTTGTGTTTAGATATTGCTTCAAATAATCAAATTTCTATTGTAAAGCCAGAAAAGGACAAAAGAGCAAAGAGCTGCTCATCAAGCAAAGGTATGCCAGGCAGTTGCATAGCTACTAGAATGTCAGTTGTTTACTGTTACCCTCTCTATTTTGAGCTTACACTGTCTGTTATTACAGCACTCTAATGCTATGTAAATGCTTTGTTAGAAATATGCGCATTGTTACTGTAGTAACAACATAATGCTTGAGCTATTACGCAGTTAAGAGCATTTATTCATAGTTATAAGCATTCCATGAGTCTTGAACCTTGCAACACAACTCATATATATTCCAAATAAGGATAAACATAAACCTACTTCCTGTCACATTAACGATAGTTTGTAGACATGCCTATCATGTCAGTCATACCTTGTGGCTGAAGCAACAGCTACTAGTAAACACCATGTGTATATATAGGAGCACTGTTTGTCCCATTGCCTCTCAGTTGCAGTCTGTTCTGTTGTTTAGGTTGGCAGCAGAAGCAAACTACCTCTCCAGTCACTACTCCAGCTTCAGTTGCATATCTTGAGGTAACTCAATCATTATTGTTTGTGTGTGTATAATTATTTTTTGTTTTGTTCTATTATTTGTTGTTGTGCCAAGTGTTCAAATATCTTTTTAAATGATATCTCTCTCTCTCCAGTCTCCAGGTACTGTAAAGCCATTCAATCCTCCTGTACAGCAATCACTCCCTGAACCAAGGCTCTATCACTCAGGAGACTTCTCTTTATCATCTGACTTTGGACCAAATTCTAGCTCCACTTTCTCATCTCAAACATTTCTTCCAGTTATGGGTATGTGTGAGATCAATCTTTGAGATCAGTCTGTGATAGTGTTATCATCATCATCAATTATTCATTATATTATTTTTATTTTTACTACTCTTGTAGATGGTCATTTGCCATGGCCTACTGTTCCTCATCCCCAAGGGCCTCAAACTCCAGCTCCACCACCCCATTTTGTGCAACAAGCAAACTTTAGAATGCCAGTTGGTCCACCTCCTTCTTATCCTGGCACTACTGGTGGTAAGTACAGTGTGTGTCAGTTAATCGAATACAAACTATCATTCATTCACTCTCACAGATCCTTTCCACCCTCAGCAAGTTGATGCTCAACAAATGGTCCTGCTTCCTCAAGAATTACAGGTACTAGTCTACTAATAATATTTATTATTATTATTATATTTATTATTATTATTATTAATCATCATCATCATCATTTTATTATCATTCACTTTCTTGCTTTAATAGTATTATGAGCATCCAAGCCAGCAGCAGTTGCCTCCAGAGTATCAGGATCATAATTTCATAGCTGGTCCCAATCCCTCTGATGTACCAGTTGATGGATTATCAAGGCAGTGCAATAATGAGTCTATCCCACCACTCCTGTACCCATCTGAAGGTACGAGTCATAATGTGTTTACACAGTACATTCTAGCTATCATTAATTGTAGGACCATGGCAACAGCCTCAAGTCTCTACTGCATGTCCACCATTGAGATCACAGTATGATGAAGCCACTCCTCACTGCATGCAGCCTTCTTCACCTTTCTTTCCCTCTGATATAATATCTGAAATACCAGTAAGTGATATTCAGCTGTTGTTGTATAAATTGACTTGTTATTATTCTGTAGCCTCACGAGTTGAGAGGGGAATCTCATGATAGCCTCATATCTACTCCAAGTAAGCTTTAGAAAAGACTGGAGAGTATAGAGAGAGAGAGAGTGAGAGAGAAAATGTATTTTTTGCAACGTGTAGTTGCATGTTTGTTTATCCGTAATAGCTTTTAAATTGCTGTTTTGTTTTTGGTTACATAGATTCTATTGGTGGTACTACATCCAACAACTTAGTCTCTTCGCAGTCTTCATCACCTACAACCACTCTATCAGTAATGGACTCAATGGAAGGACCGGAATCAGTTGGAAGTAATGGCAAGAAAATTCGTGGTGGGAGTGGGAAAAGGGCCAGGTCCATGACAGATGATGAGCGCCGTATGAAAGAAAAGGAAAGACGTAGTGCCAATAATCAGAGAGAAAGGTATGACTGTCTTGAGTTTAGTAGTACCTCTATGTTGAATTGTTCTATTAGGATAAGAGTGAGAGACATTAATGAAGCATTTAAGGAGCTGGGAGACATCTGTCACCAATATCTCCAATCAGAAAGAGCTCAGACTAAACTAATGATACTCCATCAAGCAGTTGCTGTCATTAACAGCTTAGAAGTACAAGTAAAAGGTATACATGATATTTTAAGTCGTGCTTCATTAACATAATTATGCCTTTATGATTAGATCGGAATCTCAATCCCAAGGCTGCTTGCTTAAAGAGGAGGGAAGAAGAGAAGGCACTACGTTTGAATGCTCTTCCAATGGATCCCTTGGGAACAAGTCTTGACCAATTAAACTCACCAAACTATCTCACTTCTAATAGTTTACTCACAACTTCCTCGGCGATGCCATCAGTTTGTTACAACACAACATCTCCAACCCTACCTCCTAATAGTAGTTCTTCAGTCCACTCGCGTCCCTTAGTCTACACTCCCTCTCCTCCTTCAATCGCTTCCACTGACAAAATACCACGAAGAAGTGACCAACCAATGAAACGTAGGAGCAGTGGAAACTTAAGCCCAACTTTACGCAAGAGTAAAGGTCGTAATTTACCAATCCCTAACAATCTGGTCGTTAACAGTTCTGATGTAACAGGTGTTCTTGATCCATCCTCTTCCTCAGCTTATCCAGCATCAATGACAACTTCGTCTTGATTAAAGAGGGAGTGATGGAGGCACAGACTACTCTAGTATGTGTGTTTTGGTCTAGTACATGATAATGTTTTCATAAATCTTTTATTCTGTCACCACAAACATTAATACACCAAAAATTATATTATTTTTTTTGAGCACAAAACACATCATTTCCTCAATTCACCAACATTACAACACTCTTTGTAATAAACTGTATTGATACTTTTGTATTACTAGTGTAGTCTTTTTTTCAATTAAATTCTGCAGTCTAGACTTTACTTTTGTATTTTTTATGTTTTGTAATTATTATTTTGTGATCACTTCAATCATTAAATTCAATTTGTCAGAGTGTGATAGCCAATGGTAACACGATAAACTAAGGCATTCATTATGTGAAGTTTTACAAGTGGACAACATTCTGCTAGTAAATACAGAATCAAAATCTACCAAATTCACAAAAATTAGGCTCATTTAACTCCTCAACGCAATTAATACTTCAATGGCAATGCTAATGAAATATTTACTACACTAATCAACAAAACAATGCCTTGTAAAATAGTATACTGCTCACACTTCCTCCATATAAGAGTCAGGTAACACAGGAAGTATGGCATAACAGCTGCAGGTACAAGTTTCTTCTTGAAAGCCACTGTGGTTAATTTGGAGTCCATAGGACCACAAGGCTGACCCACATAACCAAAAACTCACCCAATATTAAGCACATGACCTTCATCCTTAAAAAAATGAAGTAATAAAATAATGTAAATTTTAGAAGCATTATCACTTAAACTGGGCTGTGACATAGATCTGTATACTTGTGACAAAATCAGACTATATACACAAGTACATTTCACACATATCAATTTCACTCAAGGTCGTCTGTGTGCATGTGAGCACCACAAATCTCAAACAGTCCATGCAGTATGTACACACATTTCTGTAGTACACGTGCCAATAATTAAAGCAAAGGCAACTATGTATGCTGAAAATGTGAGAGATATTCTTTAATGACAATTTTTAAATATCATGCTTTCTAGTGATCAAAAATTCAGAGGAAAAGAAAAAAGAGTGACACACAATCATAATTAATATACATACAAACTAGAATGGCATGATAGTCACAAACGCCAATTAATAATGTCAAATTAGGTTTCGATTTCGAAAGGAAATGGAACTTCTGGCTCATTTGGTTGGTCTAGCATAGAATAAGGATAAAAGAGACAGTTACATATTACACGGTTACTTTCTGATGGAACTGCCAATGGGCTCTTGGTTCCCTGAGGTACAGCAACACTTCCCTCAGCAATAATGTCGGGTATTGGTGGTAGCCTTGTTGGTGGAGTACGAGAATAAGGAGAATCACGTAACTGGAGGAGCATCTTCCTGTCATATATAATACGTGTACCTAAAAATGCAGTAAATAAATGCTGTGACTCGCAAGGAATGCATCCTTTTATAATCTATGACATAGATTAAAAGGAATGGGTAAAATGGCCTATGTATGATAGACTAATTGCTTTACAAGTAACAAAACTTCCTGATACTAGTAAACAAAGCAAATAACGAAAAATTCCCACTACAACGGTAACTTGAGCTTAATGCCAGGGAGTCATGACATACAGTACCATAAAAGGAATACATACACACTGGTAAACAAAGAACATGACGGGATAGAAAATGCACATTCTACATACATGCAAGCATCATATTGACAGTCAATGATACCCACCCACATAATTAGCTACTTGTTATAAAAGGAAATTTACCATGAGAGCGTTTATAACATATCAAAAACAAATAATTATCACTGGTTGATTTTTAAATCAAAATAATATAATTACCCAAAGACAAAACTATTCCTCTCTTACCATAACAGTCATTACAGTTTATACTATCTAAACTGCTAAAATTCATTATTAGCCAGTAAGAGAGCAATAAATAATGTCTCATGCAGGTGGAATTTGAGCAAAGGTGGGAGGCAGTATTTGTTTTTGGAATGGTTCATTGACTATAAACTGTGAACATAAGAACTACATTCAAAACTCAATCAAGACACAGGGAGAAACATGTTGCCTGATAACTGAGCAAACACTTTGAAAAATGATTGATCTGAGAAGAGTAAAAAACAAACAACTAGCAATAATAATAATAAATTATAATACTTATTGGCACATGATTATATGCAGCTCTACCCCAAGTAGCAAAATAGTGACAACAGACACATCAGAATAAGACAAAAATAATAAGCAAACACATTATTATTAGAAATTAGTTAATGTAGAAGATAAAATAAGGAGAGGCTATACTTAGGCCAGAAGTTATCATAATAGAGAGGCCAGTTAATATAATGAAATTGAATAGTTTATTTGTCCACTCATTGGGCCATTTGAGTGGGTGAACCCACAATAATCATAATAATTAATCATATAAAATTTCATTATTTTGTAATGGCCCACAGGCTAATATTACATGGCCGGCCTACATGAATTCATTGGTCGATTGGCAATAATCAATAAACATTGTGATGGACTGATGCTTCATATAATAGGTGACAATAATCTGTTATGGCTATGTTGAACACAAAGCCTTTCATGTTAAATAATAATTTATAATTAATGTCACTATTGTGATTACTATTAGGAATTACACTAGCAAAATAAAAACCACGGAAATATTTAAAACTAAAGAGTCTTAAAAATTGTTTTGACTACCAGCAATATTTTATTCATTCTCCTAAATGTAACTCCCAACCACACTAAAACTTTTCCTAAAAGTTCAAAATTGTGTGGCAGTGTGCATAGTTTCATTGCATTTGATCAGTATAATACACCATTAACAATGACTTAATAAGATCAATGCGCCCTACATTACAACCAACCAAATGTAAGCGTAAATGCAATTTTCCACCTCTAGCAACTAACGTTACCACATATATATATATATATGACACTTTCAGTTACTGGTCATAAAGTGGAATTAAAGTATACATGAAACAATTATTTTAGCTCTATTATACTCTCATACTCTCAACATATCA

>genscan_predicted_peptide

VSNTIVQPDFMGGASESNSSDLKGWQQKQTTSPVTTPASVAYLESPGTVKPFNPPVQQSL

PEPRLYHSGDFSLSSDFGPNSSSTFSSQTFLPVMDGHLPWPTVPHPQGPQTPAPPPHFVQ

QANFRMPVGPPPSYPGTTGDPFHPQQVDAQQMVLLPQELQYYEHPSQQQLPPEYQDHNFI

AGPNPSDVPVDGLSRQCNNESIPPLLYPSEDSIGGTTSNNLVSSQSSSPTTTLSVMDSME

GPESVGSNGKKIRGGSGKRARSMTDDERRMKEKERRSANNQRERIRVRDINEAFKELGDI

CHQYLQSERAQTKLMILHQAVAVINSLEVQVKDRNLNPKAACLKRREEEKALRLNALPMD

PLGTSLDQLNSPNYLTSNSLLTTSSAMPSVCYNTTSPTLPPNSSSSVHSRPLVYTPSPPS

IASTDKIPRRSDQPMKRRSSGNLSPTLRKSKGRNLPIPNNLVVNSSDVTGVLDPSSSSAY

PASMTTSS

>Amq_1|geneid_v1.2_predicted_protein_1|364_AA

VSNTIVQPDFMGGASESNSSDLKDGHLPWPTVPHPQGPQTPAPPPHFVQQANFRMPVGPP

PSYPGTTGDSIGGTTSNNLVSSQSSSPTTTLSVMDSMEGPESVGSNGKKIRGGSGKRARS

MTDDERRMKEKERRSANNQRERYDCLEFSSTSMLNCSIRIRVRDINEAFKELGDICHQYL

QSERAQTKLMILHQAVAVINSLEVQVKDRNLNPKAACLKRREEEKALRLNALPMDPLGTS

LDQLNSPNYLTSNSLLTTSSAMPSVCYNTTSPTLPPNSSSSVHSRPLVYTPSPPSIASTD

KIPRRSDQPMKRRSSGNLSPTLRKSKGRNLPIPNNLVVNSSDVTGVLDPSSSSAYPASMT

TSS

**>Amq7**

CTCAAAATTAGGAGGAATACTGGTATCATACTCCAGGCCAGCACTCCTCAAACACTATGGACTATTAATGGCCGACCTTCCTCAGATACACGTTGATATACAATACAATGCAACTGTACTTCAGACTCCATTACACTCCACAATACTAGCAACTGTTAATAGAGTCGGTGAAGATTTTGTCACTTGTACCTGGGCAAAATGCATGAATATAGTGATACCCTATACCGAGATGGAGGGGTCTGTGATGCTCAGTGAGGGGGACCCTGTATTAGTGAATATTACAAGGGTTTCTATTGAAAACGATGAGTATGTGCTATTTGGAAGGCTATTATTGACTAATGCAATGCGGCAATCGAATAGTAAGCTTGTAATTACTTTTAAATAGAATTTTATTTGAAATTGTAGGTGAAGAAGGCACAAGAGGATCAAAGGCTAATGATGATGAGAAGGAGGTTTTGTCAAAAGAGAAGAAAAAAAAGAGGAAGAGGAAGCATTCTGAGCTAGCAACTGAAGCAGAAGAAGATATTGAAACGAGTAGATTTAGTACAATGGAAGAAATGGTACCTGGTAGTGATGATCAAGATTCCCAAATTTGTCACAAGGTGTGTAATAGTATACTATCGCCAAAGAAAAAGAAAAGAAAGAAAAAGGATTCATTAGAAGGACCTCCTTCAATGGAACATTCATCAAAGAAAAAGAGAAGAACCAAACATTAGTTAATAATGCATTATTTTTTAATTATTAAAATTTAATATTCATTAGTCATTAATTTTATTGGAATTATGTTTGAGGGTGTGGTCATAGTATACTAATCCGTAAATTTATATCCGTATACTGATTGAAATCCAAAAAGAAGGACACTCATTCTAGCCGAAGAACAAGTAGTATTTCTATTACAGAAAGAGACATTAGTTAACTGTCATCATGATGACCGACCCATTTATGCCAGGAGGGAAGAGACCTAAACTCGAGTAAGTTAGAGTCACGGAAAAGCCAGGCTATTTGCCTTTTGGTGCGTGTGTCCAGATAAATGTGCCCATCTGTTAAGTACTCGGTTAAGTGACAACTCCATTTATTCTCTTTGCTGTTAATTTGTAGTCTTTTCCACATAACCTGTAAATTGTGTATGATGTTGTTGACTATTCACCGTTAGGGAGATTTTTGTATGCTTTATGTACATTTCCTTTTACTATATCTTTCCATTTCCTTGAAATTCCTTCCATCTTTACTTTTTTTGTGTGTGTTTTTATGTGTCTTGTGTAGTTTCTCACAAGAGCTCATGGCTGGTTCCCATCATCCTCATCCCTCAATTGGGTCGATGATGTTGCCAATGTTACCACTGGATCATGATAGGTTATCTGATGATGAGGAGTCTCACAGACACAAGAACATGTCTGGATCTAAACTATCAGCAGAAGCAAAGTAAGGAGGGGGAGAGGGAGGCCAATAACTTATGTGTTTCATCTTATTCTCCTTCGCCTCCTCCTCCTCATCTCTCTCTCTCTCTCTCTCTCTCTCTCTCTCTCTCTCTCTCTCTCTCTCTCTCTCTCTCTCTCTCTCTCTCTCTCTCCCTCTCTCTCTCCCTCTTCCCCTCTCTCATAGGCGTTCACAGCACTGTGAGGTTGAGAAGAGAAGAAGAGAGAAAATGAACCGCTACATGTCTGAGCTGGCTCAGATGATACCAGCTTGTAACGCAGTACCACGTAAACTTGATAAGCTGTCAATTCTAAAGATGGCAGTTGACCACATGAAGAATCTAAGAGGTACAGTATTTAATTACACTAAGAAAAGTAGACCTACATATACTCTTGATATCACCTGCACCATGTACATGTACTGTGTAATGTATACTAATTCTTGTCATTATTTTTATGATTAATGTCATGTACACAACTTAAATCAAAGAGGATACATTTTATAATAGTGTTTACTTATTATGTATATCCATCTTAATAATGTTAATCTCTACATATGCACACGTACATGTATGCACATGTGCTATGCATGTATGCAATGCTCTTTCTTATTTGTGGATTATTATCATTCACATTCTGTTAGTTTCCCACTATTTATCTCTTTCCTTTTTGGTAGGTGACCCTCATTGTAACTCTGATTATAAGCCTGGTTTCCTAACAGATGACGAACTGAAACAACTAGTTGTTGAGGTATTGTAATAATTATTATTTAAAGTATTTTTAATCCTTCTCCTTAGGCTGCTAATGGATTCATGATGATTATTAGCTGTGACAAAGCCTGCGTCTTATTTGTGTCTGATACGATATCTGATGTGCTTCACGAGCCAGCTGTAAGTCACACGATAGTCACTTGATAGTCACATGATCTTACATTAGGAGAACTGGATCGGCTCAACGCTCTATGACTTACTGCACCCAAAGGATATTCAAAAGGTTAAGGAGCAGCTAGCATCATTTGATGTTGAAGAAGGTAAGGGATGATGATGTGTAGGAGGCACGATTTTTAAATTTATTTAAATTCTTTTTTTAGCTCTTGCTGCTAATTCAAAACACAACAGTTCAAAATGTAAGGCCATTGTCCACATGTACTAAACACTTTATCACTTGTTTACTCATTGCTTCCTTTACTTTAATAATTTTTTCTTCCTTCTTTCTCTCTCTCTCTCTCTCTATAGCTCCATTGGTAATGCACCCACAGTCAATGAATGGACTGAGAAGATCATTCTTGTGCAGAGTGAAGAGGGGCCAGTCAATAATGGCTTCACCTGATTCAACATCCTCTTCTAATGTAATTACCTCTCATAACCTTTGTTTACTATCCCTCTATCTCTCTACCCTAATGTGGTTAACCAATGTCACACTGTCTCACACACATACTTGTATGTGTATGTACATCACCTATACTTATTATACTGAAGCTATTGTGCGCATGAAAACAATTCTAATATGGAGGTGTATGGTATAAATAATAATAATATCTGTAGATGTAATTTAATTAATATATGTGATATAGTCATCTTTGATGTG

>genscan_predicted_peptide

MADLPQIHVDIQYNATVLQTPLHSTILATVNRVGEDFVTCTWAKCMNIVIPYTEMEGSVM

LSEGDPVLVNITRVSIENDEYVLFGRLLLTNAMRQSNSEEGTRGSKANDDEKEVLSKEKK

KKRKRKHSELATEAEEDIETSRFSTMEEMVPGSDDQDSQICHKLMAGSHHPHPSIGSMML

PMLPLDHDRLSDDEESHRHKNMSGSKLSAEAKRSQHCEVEKRRREKMNRYMSELAQMIPA

CNAVPRKLDKLSILKMAVDHMKNLRGDPHCNSDYKPGFLTDDELKQLVVEAANGFMMIIS

CDKACVLFVSDTISDVLHEPAENWIGSTLYDLLHPKDIQKVKEQLASFDVEEX

>geneid_v1.2_predicted_protein_1|195_AA

IHVDIQYNATVLQTPLHSTILATVNRVGEDFVTCTWAKCMNIVIPYTEMEGSVMLSEGDP

VLVNITRVSIENDEYVLFGRLLLTNAMRQSNSEEGTRGSKANDDEKEVLSKEKKKKRKRK

HSELATEAEEDIETSRFSTMEEMVPGSDDQDSQICHKVCNSILSPKKKKRKKKDSLEGPP

SMEHSSKKKRRTKH*

>geneid_v1.2_predicted_protein_2|154_AA

MAGSHHPHPSIGSMMLPMLPLDHDRLSDDEESHRHKNMSGSKLSAEAKRSQHCEVEKRRR

EKMNRYMSELAQMIPACNAVPRKLDKLSILKMAVDHMKNLRGTVFNYTKKSRPTYTLDIT

CTISIGNAPTVNEWTEKIILVQSEEGPVNNGFT*

**>Amq8**

AGGTTTTAAATCTCACGATTATTTTCTCTCCCTCTCTCTCTCTCTCTCTCTCTCTCTCTCTCTCTCTCTCCCTCCCTCCCTCCCTCCCTCCCTCCCTCCCTCTCTCTCTCTCTCTCCCTCCCTCTCCCCCTCTCTCTCTCTCCCTCCCTCCCTCTCTCTCTCTCTCTCTCTCCCTCTCCCTCCAGATCAGCTAAGAAGAGGACCCCACTAAACTGGCAGCCTAGTTCAGACGAAGAAGATGTGAAGCCGATGACGTACGACGAGAAGAGACAGCTGAGCCTCGACATAAACAAGCTCCCTGGTGTGACGTTAAATAGAGTAGTCCACATAATCCAGATGAGAGAGAGGACGATCAAGGATGGGAACCCTGACGAGATTGAGATTGATTTTGAAACACTAAAGCCAGCGACGCTGAGGGAGTTGGAGAAGTATGTGAATTCGGTTCTGAAGAAGCAAAAGCGACCACCAACCAGTAAGTGACTAAGTGGCGTCCATTTTGTTACCATGTAAAGCTATTTTGTTTTTAACCGAAAGACTTTCCATTTTAGCTATAAAATTACACATGTGTTTAATTAAAATGGGCGTGGTACTTAACCTGGATTTAGCCACACCCATTAACTATTATAATTCCTGTATAACTTGCATTATACCTGCAGTATCTCACAGTGTGTAGTGTGTATTGTACAGGGACAGTGAATTCATGAGGTATTTCTCCGTGTGGACATGATTCATCTAGTTCATGTCTCACTCCTTAAATATATCTCACATTAATACATGTACATGTACTGTATATATATGTCGTTATTATTATTGTTATTATTGTTATTAAATTATTGTTATTATCATTATTTTTAAAATTCGTAAAAAATCCAATTTTGATCGGAATCAACTCAAACTTTCTACCCAACATAAGTACATGTATATGCATCGGAAAATGTAATAAAAATGGAAAATTATCTGTTCTTTTTGTTGAAAATTGGCCCAAGTTTTTGTACTATAGTACAGGGACTTCGGCCCGATTTTGCAGCAACTTCAAAAATTTAATTTTGATTAGAATCGGCTCAAAAATTTTGTACAATTATTATTATTATTATTATTATTATTATTAATATTATTATTATTATCATTATTAGATAAGTCAATGGATGCCGCAAAGAAAAGAGCCGAACTGGAGAAGAGATTACAGGTAGGTGGAGTGACCACACCCACCACTAATTAATAAACATCCTAGGACGTCAGTGGCAAGTTGGGCGGGGGCAGCGGAATGCCAAAACTACCAAAAACCAAAAAAAGCAAAAGTAACAAATAAAAATATTTAATTTTATAAAAAATATTCTATCATATCTTGCTTAACCTTTTTTCCTCTGTTCTCAAGGTGACGCTAAAGGTTCTAAAGATCTTAAATCCTCACGATTGAGCGATTCCTCCAGCTCTTCAGACGATGATAGCGCCAACGAAGAAGAAAGCTCCAGCGGAACCGGATCAAGTACGAGCTCATCAAGCGACGAAGAGAAAGATGGTAATAAGAACTGAGAGCTGACCCCACAAATTGTAATAGAACAATAATAGAAGGAATAAGAGAGTAGCTGGCAGTTGATTGCTACTAACTAATACGTGTATTATAATAACATTGAGATAGTATAAAGGACTCTTAGTTTGAGCATTCATTAACTTGTGTATCATTTAATTAATATATACAAGTCTTGTATTTATTTGGACAATAATATCTGTGTACATATTGTGTTGAAATGGAAGCACTCTCTCTCTCTGTGTTTATGTGTATATAACTTGATTCACTCATTTATTTAATATAAAAACCGCTTAAATTAAATAATTGGTTAATTATAAAGTGGGTGGGGCCTCAGTTTCATAAGGGGAAGTCCCTCAATGTCTGTACGTGCTGTGTGTTGTTGTTAATTATGTGTGTGGGCGTTATCATAATGTTTTTTGTTTCTATAGTTAAACCAGTACAGAAGGCAAGGACACCTCAGGCAACATCAAAGGTAAGTGGCCACACCCACTGACCCACCCATTAATTATAATAATTAGCCGATTGCTCCAGCTTATCAGACGACTTCTGCGGCTTCAATTATCGGTAAATAAGCCACACCTACTTTTTATTAATTAATTAATATTTATGTATTAGGTCGTAGCCAATCAAAAAGCTCTCTTACTACAGTGCCTGCGGGCGGAGCTGTGGCCCCACCCCCTACTAAAGATGCTGCCCCGCCCAGTCCTGGTTTTCAAGAGCCGATAGAAATGGGCGGAGTCAAGAAGAGGGCGATACCCTCGGCTGATTCAAGTTTTGCACAATTTAAGAAACAAGCACTTGAGAATGCTGAACGGGTAATAATAATAACGATAATAATAATAATAATAATAATAATTAATAATAATAATAATAATAATAATAATAATAATAATAATAATTCATTTTTAGGAGAAGGCGAACAAGCAGCAGGAGGACCTAAGGAAGCAGCAGATCATGTTAATGCAACAAAACAAGAAAGAGACAGAGAGCAAGTGAGACTCTTAGTGCATCAATATGTATTGGGAAATGTAATAAAATTTGGGGTGATTTCGGAGCCATTTTTATGGAAAAAAAAACATGGGCTATAGCCTTGGGCATTTTCCAGGATTTTGGCATTTTCTTAAAATGGCTGTAACTTGAAAAATTGAAGTCGGAATTACCTCAAACTTTCTACACAGCTTAAGGGCATCAATATGTATCAGAAAACGTAATAAAAATTTGGGGTGATTTCGGAGCCATTTTTTGAAAAAAGCCTTGGGTATTTTTCAGATTTTTGACGTTTTCTTAAAATGGCCGTAACTTGAAAAATTGAAGTCGGAATCAGCTCAAACTTTCTACGCAGCATAAGTACATCAATATGTATCAGAAAATGTAATAAAAATTTGGGGTGATTTTGGAGCCATTTTTTGAAAAAAAACCATGGGCTATAGCCTTGGGTATTTTTCAGAATTTTGGCATTTTCTTAAAATGGCCTTAACTCGAAAAATTGAAGTCAGAACAGCTCAAACTTTCTACGCAGCATAATCGAAGTCCCTCGTTAATAATGATTATTATCATTAATTAGGAGTAATACTGCATCTCCGGTTGGAGGAGGGGGCGGGGCTGACAGTGGGGGCATGAACAGCCCAGTTGATGAAGTCGAGAGAGAAAGATTGAAAGAGAAACAGAGACAGGAGAGAGAAAAGGTAGCCGTTACCATAAATAGTAACAAATGTAGTAATTATTATTATTTTATATTAGATGAAGCAACAAATTGATATGACTTACCAAAATGATCTGATGAGTCAGTTTGAGATGACCATGTACTGAAGACACGATGACGTCATAATTACATCATTATAATAATCTCGTTTCATGTTATTATTATTATTATTATTATTATTATTATTATTATGTATTACATGTACGATGATTAAGGACACATTGATTAAATTGATTATCGTCTTTCTTTTTGTAATTATTATTATTGTTATTATTATTTATTATTGTTGTTGTTGTGACTCTTACGTTCATGTTGAACTTTATTTATTATTTGACTGTAGTCAAGTTCTTTCATTATCTCAAACTTTATCAAGAATAATCTGAATAAAACAATTAAATTTTGGCAAAAAATAATTACTTAATTATTATTCATAGATTAAAGACACGCCCATTCAATTGGGAACGCCCATTCCAAAAAATACGCATCCTGTTTGAAGAGGGTGTAGTAAAAAATGGCGGACCTTTTCTTCACGTGAGCTTTGAGCCATATCTCGTGTAACTCTTCTCCAAGTACATCCGTGGCGGCAAATCACAGAACCTTTTAAACAAACCTCACCTTCTGGGCTAGGACTACTTGCAAAAACTAGAGCAGAGCTGAGAAAAAGCTTAAATACTGGTGACCCGCCCACCCCTTGTTGCGTTTGAAAGAAAGAGAAAGATGGAGTTATCCACTCGGGCTGGAGATGATGGGGTAGACCAGAGCGTGGTTGAGACTGTAGAGGTGGTGGCTGGCTCTAGTGGGACAGGAGGGGCTGGCGGAGGGGAACAAAGGGTCCTGAGTCCAGGAGGAACTGAAACCACTTTAAGGATAATCAGTCCGCAACTCTCCATTCCATCTTCTGGGGTAAGACCACACCCATTGGCCCACCCATTTGTACATGTTCATGTATAATAATAGATTTGTCATTCATAAGTATTAACACTTGAACTTACGTGTACGTGTTTTTGTGTTATGTCATGTTTTTGTGTTTCCGTTAGATCATTGAGATTGATCCTCAGGCACTTAGTTCTCTTGTTACTCCCAATTTTGACGTTTCCACGGTGACCTCGCTCTCCCTAGCAACGAACCCGGCAACCACCCTCTCTGGTTCCGGACTACAAGAGAGTCTTGATAGCAGAGTAAAGAGAACACATGATGAAATGTGAGGATCTCTCTCTCTCTCCCTTCCTCCCTCTCTCCCCCCCCCTCTCTCTCTCTCTCTCTCTCTCTCTCTCTCTCTCTCTCTCTCTCTCTCTCCCTCCCTCTCTCTCTCTCTCTCTCCCTCCCTCCCTCTCTCTCTCCCTCTTTCTCTCTCCCTCTCTCTCTCTCCCTCTCTCTCTCTCCCCTCTTCCTTTTTCTTTTATCAACACAAATGTGCATACTTTCATAAAATGCATTAGTTTGTTCATTGCTACAATGCTGTTATTCTAATTAAATGCTTTGTTTGCCTTTTAGGATAAGAGATACTCCCACTGCAGGAACAACTCCTGATGTACCCAGGAAGAA:TCTAGAGTACGTGGGAGAGAGAGAGGGGAGAGGAGGAGAAGGATTAGTATAGTTTAATTTTGGCATTATTGTACTGAAATTGTTACGATAAAGACATGTTACTGTACATGTACAAAGCTATGAATTAGTCTATGCATGTACATGTACATTGCTGTAATGCTGTGTGTGCATCAGTATGGACTGTGAATCTGTGAATATGTTCATGATCTCTCATCTAGTATTACCTTGACTCTCACAAGTCCATTGAGAAGAAGAGGCGTGACAGGATTAATAATGGACTACAGACTCTTAAAGATATTGTACCAAACTGCAGGCAATACTCATCACAAGTTAGACTCCATTTAATAATCATGATGATCCTTCATTACTTCTAGTCTACTGTCTTCTCTTTCTTCATGATTGTATTACATGTAACTCTATTTCCTTCTCTCCCTTTTCCTTCCTCCCCTCTCTCTCTCTCTCTCTCTCTCTCTCCCTCTCTCTCAGGGTAGTAAGAAGTTAGACAAAGCTGAGGTCCTAGAGATGACGATTGATTACATTCAAAGACTTCAACAAAACCAACCAAGCAACACAGGTATGAACTATTTATTTATTTATTTATTTATTTATTTATTTATTTATTTATTGGTTTATACAAGTAGATATTGAGGCGTCACAGAGAGAGCTTATAAATGATTTGATGTCCTGGATATTTCAAAATAAATTGTTATACACCGGACCCAATGGAGTAGAGCAGTTCTCTAATGCTTTACTCCTTCACCTTCAAAGCTTGAGCTCTTCACCAACTGTCAGCACTGCTACTGGTCTCATGTATAACCAGGTAGTGTTGTATGTACATGTACAGTGTAGCCTCTCTTAATGGACACTCTCTATAGCGTACACCTCCATTTAACGGACAGCACCTAGTGTACCAATTACATTCCTTACTGTATAAAATAACCTCCACATAAAGGACACCTCTGTATAACAGAGACACAGTGTCCATTATATAGAAGCTACACTATATTTATTATTATTATTATTGCATTTATTTATTTTTACTTTTTCCTTAGTATTCTGATGGAGGAGGTAGATCAGCATCAGAAGATGAGCAGCATTCCTTAGCTAACACTAACAATGTTCTCCTTCAGCAGCTAGCCAGTGCTACTGGGAATACCTCAGAAACTGCCGAAGAAAGTAACGAAGAACTCTCCGAGAAAGAAACCAACGAGACTCAAGGAGCCACGCCCTCTACTGCTGGACAAGGAGGCGTGTCTGGTAGCGTGAGTCAACAGCAGAACCAGCTGGCACAGCTACAAGCGTTACTGTTGTTACAGCAACAACACCAGCAGCTGTATCAAGGGGGCGGGGCAGGGGGTGGGGCTACTGGTGGAGGAGCTGAGGGGACAGGGGGAACTGGTAGTGCTAACGTCGACACCCAACAAGTAAGAGAAGAATTAAAATGAAAAACTAGAGACGATTAGAATTTTAAATTATAATGTCAATTATTATTAATAGCAGTCAATGAATATCATTATCATTAATTAGATTCAGCAGTTACAACAGCTCCACCAATTACAGACTCTTCTACTTCAACACCTCACGACTGAGAAACAGCCAACGCCCATACAGGAAGTGGGCGGGGACGGCAGCATCAACGTAACACCCTCACAGTCTGTAATGGTTAGATTCTTTATTTAATTATTTATTTTATTACTGTTTCAATTTGGCTGATGGCCATTTGTTGTAAAATTGGTGGGTTTTGTATTTGGCAGCAGCTGTAGAATACAATTCTATGTATAATGTTTAAAGAATCATGTACATGATCCAAAATTGATAAAATAAATAAAGAAAATAATTAAGTACATTTTTGTGATAATCCATGCCATTTTACTGACTCTCTCTCTCTCTTCTCCAGGTCAGTACTGGCGGTATAGACAGCACTCATGATTCTGTTGGTCTCTCCCTAACGAGTGAACTAGGATCCACCACTGCTGTGTACGCCACTGATGAGTCTCTGGTAGTGGGTGGAGCTTCAAATGAAGGGTCTTCACAGATGTGGTCTCAGACCAGTAACACTGTACATCATGGTATGTCATTAGGGGGGCAAGGGGGCATGAATTTCTGTGAAATTTTTATGCTAATTTTGATGATAAATTTGGCGTATTTTTTTTTTCAGAGTCGCCGCTGCTGATGGAAAATGAAGGCGGAGTTCAGGAAGAGGTGGAGCTACTTGAACAAGACAAGGCTAATTTTTAACCCTTTCTGAACACAGATAGTATTATTAATAGACACCCATCCTTTACAGACTAGAGCTACGCACTCGGAACACTTTTTTAATAAAAAGCTTTTTATGAGATTTAATTAATAAAAATTATTGTTATTAATTTAATGGCGTGGGCGTTATGCAGCCTTTCTAATGACCTGTTTTTATATTCATTATAATACTGACTGGCTATTATTGGCATTTTCTTAAAATGGTCGTAACTTGAAAAATTGAAGTTGGAATCACCTCAAACTTTCTACACAGCATAAGGACATCAATATGTATCAAAAAATGTAATAAAAATTTGGGGTGATTTCGGAGCCATTTTTTGGAAAAAACCATGGACTATAGCCTTGGGTATTTTTCAGATTTTGGCTACTTTTTAAAAATGGCTGTAACTCGAAAAATTGAAGTCGGAATCACCTCAAACTTTCTACACAGCATAAGTACATCAATATGTATCAAAAAATGTAATAAAAATTTGAGGTGATTTTGGAGCCATTTTTTGGAAAAAACCATGGACTATAGCCTTGGGTATTTTTCAGATTTTGGCTCATTTTTTTAAATGGCCATAACTTGAAAAATTGAAGTCGGAATCACCTCAAACTTTCTACACAGCATAAGTACATCAATATGTATCAGAAAATGTAATAAAAATTTGGAGTGATTTCGGAGCCATTTTTTGGAAAAAAAACCACAAACCCATGGTTTGTACTTTGTTCCCTAAAATTTGCTACTTAAAAATGTTCATAGATAACTCTAAAAATCAATTTTATTTGGAATCAGTAATTAAATTAGCTTAGAGCCACGCCCATCATGTGATTGTGATTGCAAGCTGGTGATAAATGGTAATATCGTTGTACTCAGTATTGGCAGATGAACTGTTTCTCTTCCCTTTTAGGCCAGCCTCAATGAACAACTGATAGTCAACGAAGAAGAAGAAGAGAAGACAGACAAGAGTCACCACATCATGTCTTCAAGTGAGAAGAGCGAAAATGAGAGAGAGAGAGGGAGAACCTAAAATTTGAATATTGCTGCAGCATTTTTGTTGCTTCCTCCCCCTAAAACGAGCCCTAAAAGGCTTATATATATGTATAAGTTATAATATTTGTCTCAATTTTTGAAAAGTCATTTTCTTTTTTCAGTGGCAGCTGGTCTTGAAGATGACAGTACTAGTGAAGCAACTCCATTATTGTATCGACGTGTCACTGTCAACCATACAAGAGTAGACCCAGAGGTGGCCATATTGCAGGCTTCCGTATTTATTGAAGACGGAATCCATTATAGATCAATTCATCACAAGATTGATCCTTTGTAAGATAACGTTGTGTTTATTTTATCTCTACTTTGATTGTTTAGTAAACATCATCAAGTTATACTTTCTCTCCCTCTCCCCTCTTCCCATCTCTCTCTCTCTCTCTCTCTCTCTCCCTCTCTTTATCCAGGTCACTAAAGTTCTACAATATATATCATTCTTTTCTGGTCCAGTGTTTTCTAAACATTACAGTCTTTATAATCCTGATCCTCGCCTTCTTTGAATACCCAACCTCGTTGAAACTCTCCTCTGATTATCGCTACGCTAACATCACGCCCACACTTCGTGAGCCGCCATGTGGTGCCACAGAATTAGTCGAAATAGTCTGCCTCGTGATATTTCTAATTAAAGCCCTCATACAGTGTTGGCTGTTGGGTTTGAAAAGGTTCTTCAAGCAACCCTGGCTTGTTTTGTATTTCGTGATGGTCGTGTTATCTTTTTTTGATCTCTCGATATCCTTTGGTTTTTGTGTCCATTCAGGTCAGTCTTCTCTCGGTTCTACTATCCGTATAAGACGATTCTTTCGTCCGTTTTTTTTCCTCGTCCCGTCCAGTATCATGAAGAAGTTTGTGAAAGCCGTCATGCGAACTTGTGTACAGATATCGAGTGTCTTAGTACTTCTGGTCATTCATTTATATGTGTTTGCGATGATTGGAATGTTGATATTCCCGAGACCACTCCCTCACAGACATGTGAATACTACGGATTGGGACGACAATGAGGAAGAACCCAGAGGGTTTTTATCTCGATATGGCGACTTTTCCGATAAAGAAGGCCAAAGACGTTTTAAATCTGTAGAAGATTCCTTGATATCTTTACTCGTGTTTCTAACGACGGCTAATAACCCAGACGTGATGACACAGATATATCAATACAACAGACTCTCTTTTATTTATTTTTTTATCTTCCTTTGCATCGGTCTCTATTTGATACTCAATCTTCTAACTGCGGCTGTTTATTCAGAGTTTCGCGGCTTCCTAGAGCAGTCGATGCAGTCCAGTTTTGTTAGACGGGTAGTGGCCTACAGAGCTGCCTTTACTGTCCTCGCTCAGTGTTACAGGAGTAATAGTATGACTGTCCAAGTAACTAGTAAAGATTTGGTGAGACAGTTACTGCGAAAAGCTAAAATACCCAAAAACCACTTACCGGCAATGTACACAGCACTGGAAACCGAGGAAGGCTCTTCTGTAATGTGGACAGAGTTTCGGGTGATATTCAATATCATTTCTAAAGACTCAAATAGTCGTTTAGGCGAAGATGTTCATTATTATTCCCGATTTAAAGTATTGGAGATATTGCAAAAACTAGTTCGACACAACGCTTTTCAATACTTCACAATTTGTATGACCCTGATCCACATAATAATAGTAACAGTCGAAATGGAAAGCGACTATTACAGTGTAGTTAGACAAACTGATTCCGCACTCGCCATTGTAAATTTCATCTTCTTCTTTTATTACATTTTCGAACAGCTCCTAAAAATTATTGGGCTTGGAGGACGAATTTATTTTAAACATTTTTTGCATATTTTTGAAGGCGTCGTTACCATAGCGATTGTCGTCACGGAGATAACCATCCTTGCCATGTTTGGGCATCCTTTCCATCACTCCGAGTCAGAACCAGCAAACTATGCCACTTTGATTCGAGTAATGAACTTATTCATTGTATTCCGAATGCTACGAATCATACCACAAGTTAAATCAGTTTCTTTCGTGTTCGGAACTATGGTCGAAATCGTGAAAAACCTTCGAGCTTTTGCCGGAATTATCATCGTCATTTATTACCTTTTTGCTCTTCTTGGAATGGAGATATTTG

>genscan_predicted_peptide

XSAKKRTPLNWQPSSDEEDVKPMTYDEKRQLSLDINKLPGVTLNRVVHIIQMRERTIKDG

NPDEIEIDFETLKPATLRELEKYVNSVLKKQKRPPTSDAKGSKDLKSSRLSDSSSSSDDD

SANEEESSSGTGSSTSSSSDEEKDGRSQSKSSLTTVPAGGAVAPPPTKDAAPPSPGFQEP

IEMGGVKKRAIPSADSSFAQFKKQALENAEREKANKQQEDLRKQQIMLMQQNKKETESKK

RKMELSTRAGDDGVDQSVVETVEVVAGSSGTGGAGGGEQRVLSPGGTETTLRIISPQLSI

PSSGIIEIDPQALSSLVTPNFDVSTVTSLSLATNPATTLSGSGLQESLDSRVKRTHDEIL

SSSPTVSTATGLMYNQYSDGGGRSASEDEQHSLANTNNVLLQQLASATGNTSETAEESNE

ELSEKETNETQGATPSTAGQGGVSGSVSQQQNQLAQLQALLLLQQQHQQLYQGGGAGGGA

TGGGAEGTGGTGSANVDTQQVSTGGIDSTHDSVGLSLTSELGSTTAVYATDESLVVGGAS

NEGSSQMWSQTSNTVHHESPLLMENEGGVQEEASLNEQLIVNEEEEEKTDKSHHIMSSMA

AGLEDDSTSEATPLLYRRVTVNHTRVDPEVAILQASVFIEDGIHYRSIHHKIDPLSLKFY

NIYHSFLVQCFLNITVFIILILAFFEYPTSLKLSSDYRYANITPTLRQSSLGSTIRIRRF

FRPFFFLVPSSIMKKFVKAVMRTCVQISSVLVLLVIHLYVFAMIGMLIFPRPLPHRHVNT

TDWDDNEEEPRGFLSRYGDFSDKEGQRRFKSVEDSLISLLVFLTTANNPDVMTQIYQYNR

LSFIYFFIFLCIGLYLILNLLTAAVYSEFRGFLEQSMQSSFVRRVVAYRAAFTVLAQCYR

SNSMTVQVTSKDLVRQLLRKAKIPKNHLPAMYTALETEEGSSVMWTEFRVIFNIISKDSN

SRLGEDVHYYSRFKVLEILQKLVRHNAFQYFTICMTLIHIIIVTVEMESDYYSVVRQTDS

ALAIVNFIFFFYYIFEQLLKIIGLGGRIYFKHFLHIFEGVVTIAIVVTEITILAMFGHPF

HHSESEPANYATLIRVMNLFIVFRMLRIIPQVKSVSFVFGTMVEIVKNLRAFAGIII

>Amq_1|geneid_v1.2_predicted_protein_1|690_AA

MTYDEKRQLSLDINKLPGVTLNRVVHIIQMRERTIKDGNPDEIEIDFETLKPATLRELEK

YVNSVLKKQKRPPTSDAKGSKDLKSSRLSDSSSSSDDDSANEEESSSGTGSSTSSSSDEE

KDVKPVQKARTPQATSKYSDGGGRSASEDEQHSLANTNNVLLQQLASATGNTSETAEESN

EELSEKETNETQGATPSTAGQGGVSGSVSQQQNQLAQLQALLLLQQQHQQLYQGGGAGGG

ATGGGAEGTGGTGSANVDTQQVSTGGIDSTHDSVGLSLTSELGSTTAVYATDESLVVGGA

SNEGSSQMWSQTSNTVHHESPLLMENEGGVQEEASLNEQLIVNEEEEEKTDKSHHIMSSS

QSSLGSTIRIRRFFRPFFFLVPSSIMKKFVKAVMRTCVQISSVLVLLVIHLYVFAMIGML

IFPRPLPHRHVNTTDWDDNEEEPRGFLSRYGDFSDKEGQRRFKSVEDSLISLLVFLTTAN

NPDVMTQIYQYNRLSFIYFFIFLCIGLYLILNLLTAAVYSEFRGFLEQSMQSSFVRRVVA

YRAAFTVLAQCYRSNSMTVQVTSKDLVRQLLRKAKIPKNHLPAMYTALETEEGSSVMWTE

FRLLKIIGLGGRIYFKHFLHIFEGVVTIAIVVTEITILAMFGHPFHHSESEPANYATLIR

VMNLFIVFRMLRIIPQVKSVSFVFGTMVEI

Note: Predicted proteins does not contain the bHLH domain

**>Amq9**

GCTTGGAAGGAGCTACATGCCAGGAGGTAATTGAATACTACCAAACAGCAGCATATACACTTGTACTGCTAATTGTGTCTATGATAATCTTCAAATGTCTTTAGATGCCACAATGAAGCCTGGTCCAGGCGCTTATAGCCCTGAAAGAGTTTACCTGAACAAGACCCAAGCACCAAGGTTCTCTATGGGAATCAGGCACTCAGAGTTCATTACTCCGCTCATATTGGAGGTCCGTTGATAATAAAGAAGGATAAAACAAACCATTAACTATAATGATTTGTCTCTAACGCTAATACCATAGAAGCTGTATTGCTGTAGTCTAACTCTATAATAGTACATAATTTTATTTTAACTATGATACTTGATAATTACATAAAAATAAAGATTATCATAATATGCACATAATAATTATTCTATGATTCTGGATGAGGACGTTTACGTTTACTGACATAGGGTTGTACAACAAGAGGAATAGGTTGTTCTTTAGTCTGTTGAGATGATTGATAAAGCTTGCTTTCAAATTCCATCCTTTTGAATAAAAAGAAATCAATTTCTTGACAATAAAACTAAAATACATACTTTGTATGAGAACGAGGTCCCGCATTCCACACTCCGCCATAAGAAGGAAGCCTATACTAAGATAAAATTAAAGACACAATAAAAACTAAAAATTACCATTTCGGATTATCTGCATCAAGTTGACCTGATTTTCTGAGATTGCTATGAAAGTTGGCACCAATGCGTTTAGGATTTCTTTTCCCTTTACCATGTTGATCTCCTACAAAATTATCACAAAAACGTCATCTCTTTCTTTCTCTCTTTCATAAAAGATCGCTATGCTTACTCCAATGTAAGAAATCATCTTCTGATGGGCCGATTGGATGCTTATAACAAATACCGCCATGATGATTATCTTTCTCTTCTTCTTCATCTTCATCATCATTTAGTAACCAAGGTGGTGTAGCTCCAGTGTGAATATTGCCTCTTTTTGAAGCTGTTACTCTGCGGTTAATCAAAGTAAATGAACAGCACTTCAAAAAAACTTACTGAATTTTATCCGACACAGAAGCACAAGCAGATGATGCAAAGTGCTAAAATAAAAATCATATTATATTAATGGTGCATGCATTATCTCTCATGAAAAAAACCTTTTGAACTCTTGTTTCTGCTACAACTTTTTGCTGTGCTTCTTCTACCTCTTCTATTTCCTTAGCAGACTATAAAAGGGTCCTTCCATAAAAGACAGTAGAATACATAACTAAACCTCTTTGAGTCTCTTGTTCCTGTTAGCATTAAGTTTGTCTAATTTTTCGGCAACAAGTTCCTTAAATCTAAAAATAATTACTCGTGTGTACATCAGTAAATAGTACGCATTACTTATCCAGCTCTGCCTGGTAGATAATTAAGTTTTGCATCTGTGACTTTTTGATACCATTTTCCCAGCAGTAATATCTGGTTTTCTTATGATGACTCTCACTATTATATGTTAAGGAATTATTCTACTAATTTGCAATGAGGATACTTTGCATATTGACGGCTTAGAACAAATCCCTATTGCTATAAAGTATCATGTTGCATTACCTAGTCATGTGTTCTAGCAATCCGCCCCATTCTACAGTTGTAAAACCATCAGATATGTGCTTATTAATCTCTTCTTGGCAGGAGTAACACCACACTGTAATGCCAGGCTCAAGATCCCCATCAACAATATTAGGCTTTTGCAATAGACACCTAGCACTATGTACCTGGAATTAAAGGGACACGTTCACGGAAATAGAATGCACACGTTACTGTAGAGGAAGTAAACCTTAAATTGGGCGTGCAACCCAATATCCGTGTATAATATCTATGGTGCAACAGATCATTAATTAATAGTTATGACATGTGTTTAGACACGCGTAGAGAGTAGAGACAGCAATCAGTCGCAGAACCAGGAGTAGGCATCTGTAGAACACTTCTTTCGAACAGAATTAGCTCTTTTGCCACCCTAAGATACTCTTTTTAGTGAAGAAACACCAGGAATATTTACAAGTAGCCACTCGAGTAGCCATTTTGCCTTAATTATTAGGAGCGCAGCCACTAAACGTCATCATTAATGATGTCATCTTGAAATTGAAAAACGGGCCAGTTTTTCAAGATGACCTGCTTCCGTGCATGTGTCCTGGCCCTTTAAATGTGCTAATTATAAATCATTGATGATACATTACTTTTTTATTAAACTTGTCAAGAATGAGCTTCAATTCGGTTTGATGCTTCTTGGAAAATATGTGTCCTTCCCTTCTGTCATTGTGGTTCTTTCTACAAACAGAGCAAAAGGTGTAGTCGTCCTTTGTTTTGGATTTTTCTATTTTCTTGCTCTTGGAGGCCATTTTATTTTAGCCCCGCCTATAATATAACTATTAGCCCCACTCATCACGTGTTGAACACGTGAGAGCCATAACGCCAAGAGTCACGTGACACTTTACCATCACGTGACTTAGCCCCGCCATAAGAGGAGTCCGCCATTGCGCTGCTGCTTCTTCTTGTCAATCTTGTGACCTATCTCGTGACATTTGACTGACCAAAGAGAAAGAGGAGCGGTTCCAGGACTATGAGTCGTGGCGGCGATGGGAGTGACTTTTCTGATGAGAATGGAGGAAGAGGTGGCGAGTATCTTCAGAGAGGAGATGATCCAGGAGACCTGCACCAGTCTAAGAGGCTGAAATTAGGCGGCGGTGACTCGCCTACCCGTATACTCAGCCTACACACTGACTGGGTCCAGAGAGAGCCAGAAGATCGAGCAACTACTGCTTGTATGCAACAACTATAATTATTATTGTCAAGAGTATTTATGCATAATTAATTGTTTTTTTTATGCCTCTAAAAGGTGTGAATTGGGTGTATTTTATCGTCTAAAATTTTTTTAGATGTAGGAGGTGATCCCAGTGATGGTCCTATCCCAGTTCCTCAGTCCGACTTTGAAATTGAGATCAACAGTACGGGCCAGGAAGGAAATGGAGGCTCGCCAACTCAAGAAAGTGGCATCACTTTAACATCTGCTTCAATCACGATAGAGCCAAGAGTAAGTAATAAAAATAATGATGATAATGATGATGATGTGCAATTGTGTGGAAGTGCAAAGGAATGTTTGCATTCTTTACACGTTTCGAAATTGCGTTAAACTCTTTTGAATGGTGATGATAGCATAATGGCGCTAGTAACTTTCAACTTTAAAGGAGATAACGATCACTAGAATCTTAATGAAATACCATTGTACCTTGAAAGTATTTTTTAACCTCTTATATCAGATATTGACAATTGATTCAAGACTACCATCGTCTGTTCCTATTACTACAACAACGGATTCATCGTCATCAGGAGCAGCTATACCTGTTCACAAGATGATTGCTCACCTCAGAGATGAGGAGGAGGAGGAGGAAGAAGAAGATGAAGATGAGCCTTCGATGTCTAGCACAATGGTCACAAACCTCTCTACTACTGAACCCGTTGTACAAGCTCCTCTTGTGGAAGACATTCCAGTTTCAATGGTGGCTTCTCAGACTCCTGATTTCTCTGCTCTAATGGCTGGTGCTAAAGGGATGTTACAGGTACATGTATGTATAGGTACATAAACTAAAATGTAATATTCTTTAATAAAAATTTTAGGTACCTACTGATCAGCAATCACTGATACATCAACATCCTGACCTGTCTGGTGGGGGCCAGTATATGCAGGCACCCGGTGATGGAACTGGGACTGGAGGTATAAGATATAATAAGAATTAATCCTTTTATTATAATGTCTTAAAGGCCCGTTGTATGTGATGATACCACCCACTACTAACAGTGCTGAGTTATTGCAGGTGATGCAGCAAGGACAGGTTCAAAGACCATTAATGCCTCGTCTCTCTACTGGTGCTCTAGTGTAAGTCATTAAACAATGTTTGTAGTACTGTAACTACTGTACATAATAAATGTTTCATGGTTTTTCCTATATGGATATAAATTATCATTTTTGGAGCTAGACTTAACAATTTGTCTCTTGTAGTTCTCCCATATCAGATGATGGTAGAAAACACAAAGAACTAATTCGTAAACAGACTCACAATGAAGGTTAGTCTCTCTCTCTATGTCTGATAAGATAACAATCCTTTCTTTCATCTTAGTTGAAAGAAGGAGAAGAGATCGTATCAATGAGCTGATCAAGATATTAGCTGAGGTTGTCCCAGGCTGTCAAAAGAAAGACTCATCTAATGGAAATATTGTTGGGGTAAGGAGGAGTAAAGGACTTTATAATATCATATTGAGTCTCCTCTTCTTTCCTTCCAGTATGTGTACAGCAAAGGTACTGTATTGGAGAAGACTGTAGAGTATGTGAGAGAACTCATTATGCAGAACGAGCAGCTGGTAGCAACAGCTAAACTAGCCGAGAAGTCCGCCAGTGCACTTCAGATCCTACAGAACCAAATAACAGTTTTAGAGAAAGAGAATACATTCCTTCGTGCTCAGATGGTTCAGCTAGGGATTGATACTTCATCTACTGCACTCAGCGGTGCTCGCTCTCTTCTTTCTAATCCTTTAGCTCAGAGTCTTTTGAATACTCAAGTCACTCCTCCTCCTCCTCCTCCTACTACTAATACGTCACAACTGCTAGTGTCTTTAGCTCAGACCCTTACCAGTAACCCACTCCTGGCTTCTCTTTCTCAACTCAACAACAATCCCAGTAGTACTTCTGCTGTTAGTCCAGTGGTCTCTAATGAAAGCCCAGTATGTTTCTCTTAGTAGATGATTTATTTAATCATTCGTATCTGATATAGGTTACTGCCTCTGGTCAGTCTTCTCAGCTTGTGCCTCCAAACAGTCAAGCGGCTATTCTTATGAACTCTCTTGTCCAAACACTTGCTACCATCGCCAATAGCAACACTCCAGCCCCACCCCCTCCTCCTCAGGTATTAACATGTCATACTCTTTAAAAATTCTTACACTTTCTTTAGTCATCAGGTTTATCTGCATCTGCAGCAATGTCTCTCCTTAATTCTCTCATGATGGCTCAGAACGCTCTCTCTGCTACAACAACTCACACAACACAGGCCATACCACTCTCCTCTCATCCTTCTGCTTCTCCTACTCCTGCCAGTCTATTAGTCTCTCCACACGCTCAAGATAACAATAAGATCACACCACCAATGTGATATACCATATACTGTCAAGAAAATCACACAATTATATGCTCTCTTGTCTTTTGCACTTGTACATATCTTGCTATAATAATATATTATGATGTTTGTTTGTGACATTTTAGGAATATTGCTATTATAGTTTTTTAATATAATACATACTGTATATTTTTTTGTTGGCTCAGCAGTACCTTTTCAATCATTTTAGAGGTTGTGTTTTAAGTAATGCTAAACGCTGCCACATTTTTACTTACTTAGATATATATAAGAAACGAAAACAAGAAACATGTGATAACAATAGGAGTTTGATTTTTTTTCAGTACTTCTCTAATCTCCTTGCCATAAAGACAGATAACGTTGCTTGACAGCATGTGCAGCTAGTTTAGGTTGTCTCTCACGAGTGAGGAGTCCTTTACGATTACCATCAACTCTACCAGCAGCTGCATGAAAGAATATAAACAGAATTAGTACTTCAAAAAAATTCCACCTTACCCTGAGCTGTTTGAAAATCGGCAAAGTTCCATATCAGTTCTCCTATGAAGAAAGTTTTTCGGAACTCGTCAAAAACCGGCCAGTAATCCATTATTGCACTCAACTAATATAATAGGAAGAGAATATTCTTCATTTAAAAATAAGCAGTTGGTGATAGTTATACCTGAAACTCTTCAGTCCACATGACTGGAGGGTCCTTGTGGAATCCAGCGATTGCTCCAGCTCCATACTCCGATATCATGATTGGCTTCTTGTGCACTTCTCTCCATTGCTGTAACTCATAGCGCATTGGATCAGCAGCTGTGTCAGCTCTTCCGTTATTATCGTACCAATTGAAGTATCTGTTCAACATTATGACATCCATGAACTGGTTCTGCAGGGAGTTGAAAAATAGAGACAAACTAGCTCATGAAGGCAATCATAATTATGAAAAGACTATTTCATGCAACACATTAATGTTAAATGACTCACAGCTTTATCAGAATAGTAAGCTTGATTGCAGGCAAAAGTGATTGGTCTTGATGGATCCAGGTCACGTGTGGTAAAGGCTACAGTTCTAAGGAAGACAAAAATTTTGACAACCTCATACCCTCAATAGAATACTAAGAATAGGGTATAAAATAACAGTGCAAAAACAGCTGAGCATCAACATTCCTATTTTTATGCCTTTAGTAAGATGTACACTTCGATTTTATTAATAAATTGCATAACACATATGGTCAATGTTTGAATTGAATTTTAAATAATTATGAGGACCTCATTATACACCGCCTGCAATAAGAATATCTAAAATTATCGTCTACAATCGTACTTGAAATAATTCTCAGCAACTGGAAGGTCAGAATGAGGCTCATTGGCTATTGACCACATCACAACTGATGGATGGTTCTTATCTCTAGTAATCATTTCTCTCATCACCTCAAGATGATGGTGTAGAGTCACGTTAGCAAAATTATCTTCTCTATTATAATGATAAAAGGTTGATAAAATTTGTGTTTTCCTCCTTACAATTGTAATCCAACTGCAGGGCTTTCTCCAATGACTACAATCCCTTCCTTGTCACAGAGGTCAAGCATTTCTTCAGCATAAGGATAATGACTGGTCCTGAAAGCAGCCACATTCATCCACTTAAGTAAATTAATGTCCTTCATGAGAAGAGCATCGTTTATGCCTTTACCAAGTATCTATGTATTCAAAACAACACTTTATATCATTTGCTTTCAATTTGTCTTACATCCCAGTCTTCATGCTTGCTGACACCATGAAAGTAAAAGGGTTTGCCATTAATGAGAAACTGATCCTCTGTCACTTTAACCTCTCTCAGACCAACAGATTGTCTATAAACATCAGTAAGACCACCAGTAGTGATCTGTACCTGCAAATACACAGCATGTAGAAGACTATAATAAAATAACACAGTTTAATACTTGGAATGATTGCATAGCTCCAGGAGTAGAGCTAAGAGTCCAAGGCCACCATAGGGTAGGATCAGTAATCTTAAGAGTACCTTTAGCAAGTCCATCAGTTACAGTTCCTACATATTATATTAATGGATGACAATAGCAGTATGATAGTACCTGAATCCTTCACTCCTGATAGACTGACTTCATAAGTTGCTTCATTATTTCCTTCTACTCCTCCTACCATTACTGTATATGCAACACTAGCACTCCCATCTTCCTCCAGTTCAGTAACAACACTGATATCAGATATATATGCCTTGGGAGTGGTATAGAGTTTTACTGAACGATGAATACCAGCATAATTAAAGAAATCAAATTGCCCATCGTATGAGTAGTAAGGATAAGAATAACTATGACATGGCAACATTTTAAAAAGCAAAACAATAAATTGTTGTTTTACCGAGGGTTGTCATGGAATGTAATTGCAGCTGGGGGCAGAGTATGAGCATCAAGTGTATTGTTAACAGCAACAGTCACTCTGTTTGGTTGTCCTTTATCCAAGAGATCAGCAATATCAGCTTCAAATGGTAAGTGACCTCCATCATGTGTCATCACTTCATTACCATTGACCCACTACAAGTTATAAACTAAGAGACAGATGATACGTGTGTACTTACCACTATCGTGTAGTAGTGAGCACCCCCGAATCGTAACACTACGCGGGTTTTCCCGTCTAACCAAGAATCGGGTACCCAAAAGTTCCTGTCATACCAAGCCCAACCAACGTAGTCTCTTATGCTAGGATCTTGAGTAATGTCATTGAAACTGGCAGGGACTGGCATTAAAATAGTCTTTCCTGTCTGATTATATTAAGTTTGACAATTTTATGTGTATCATTTTGAAACTTACTGATTGCAAAGCCTGGGAATACCACTTCTCTTTAAATCCTTCATCCCTCAGTTCTGACCTGTCTATTCTAAAGTGCCATATCCCATCCAGGTCTTTAACAAGTCTGGAAGAGGAGTCCTTTGGATAAAGGAGGCCTTCACAGTAGTTGTTAACAAGTATTAACAAAGTGACAACAAGAAGAAGCGAGGGAGCATTCGCCATTCTACGAGCCATTCTAAGTAGTAGTAAAGTAACTTCACTAAAGTATTGTAATTGTGGTTGCCTGTAACAACTTCCCGTTTCTTTGCCGCACCTATTTACTTACGCCCACACTATCAAGCGAGTAGGTAAGAAATGAAGCCACTTCTTGTTCTTTTTCTATGTCTTCTGAAAGGAAGTGAGTCCATGACTCTGTAATTTTTACTCCTCTGGCCTATGCATATGCTATGTAAAGTTTTTTAAAAGCTTAAAATTGCAAAGAATGATTCTGACTTTGCAATTTGTAATATCTATTGAGTTGGAAGACAGTTTCTGACTTGAAATGAAAATGAATAGATTTATGCCCTATATTTTGCCTGTATATTAATCTGGTCTTTTAAATCTAATTTCAGTTAATGCTGAACTTGAAGTTAGTATTCATCGTATTCCCGATCAACCGATCCAGTTATACTCTGGGGGCAGTGTAGTGCTAACTTGCACTGTCACAACAAGTTCTCCAAACATCACTGTAACTGTGGAATGGTTCAAGAGTTTGCAGTCACTACAACACGTCACTAACAGTATACACTTATCTCCTCCTTATCGACTACAAGAACATATATGGCAGTCAAGTATTGATCTTAGAGTACTCACTGTCCAATTTTCCGGTTGGTATTACTGCCTTGCGACAACTGTTGATGGTAGTTCCACTCAAGCTGCATATTATCAAATAAATGTGGAGGAATCTGGTAATGAAATTTCTGATATCACTTTGCATGATACATATTCTATAGGTAACAAAGCAACTGTTGAGTTATCAGTTTCACCAACTTCACTATATGCTCAAGTACCTTTTACTGTTACTTCAATCCTGCATTTACCAGCAGTCATATCTCCACAAGTGAGAGTGGAATGGTACAGGAATGGAATGAAGAAGAACAATGATGATGTTAGAACTTTCTTTGTTAATGCTACAACAGTAAACAGTGTTCTACAAGTCACAGAGCCTTTTCCAGCATCTGGGATTGCATACACTTGTGTATTCAATGCTGAAATGTATGGAACTAGCATATTCAGTTTGAATGGGTCAACCACTGGATTGAGCATTGAAAGTGAGTTCCATCAATTTTTCAAAAGTAATTTATAATTATTATTATTGCTGTAGGTAAACCCACTCCTCAAGTAACAATACTCTCCAATGAGCCAAGCACTCCTTCTTCATCTTATAGTGTGACATGCTTGGCTCTCGTGTTAGTCAATGGGACACTGAAGCTAACATGGAAAAAGGATGGACTATTAGTGAAATCAGTTGATAATGTACAGTTAGGGCAGCCCATGAAGACACATAATAGTACAAGCTTGAGGATAAGATTTGTCAATGGGATCAGCCAAGAAGATGAAGGAAGATACATTTGCTCTGCTTCACTAGAAATGAAAGAATACGATACCATTTTAACAAAAACTGCTTCTATTAATATTTTAACACCAAGTAAGTAGGGAAATATTTCTAGAGTATTATTTTTTTAATAAATTTTATAGCACGCATTCTTAGCATACAGACAAGAAGTATACCGTCCAATACTAAATGGATCGCTGGGAGTGATGCCTCATTACTGTGCACAGTACAGACTAATAGAAAAGCTAATGTTAGCATATCATGGGAGACTAACACTGGGTCAATCAGTACTAACCATCGCTACTATACTAATGCTCTTACTAACACACTTTATGAAAGTGTACTGGAGCTACAATCAGTTCTTCACTCAGAGTTGTATTACTGCATTGTTTCAATTAATGGATCCCCAATGAGTGAGGAATGGTACTATCTCAATGTATTGAGCAGCAGCAGTAAGTACTTTTTTAT

>genscan_predicted_peptide

MSRGGDGSDFSDENGGRGGEYLQRGDDPGDLHQSKRLKLGGGDSPTRILSLHTDWVQREP

EDRATTAYVGGDPSDGPIPVPQSDFEIEINSTGQEGNGGSPTQESGITLTSASITIEPRI

LTIDSRLPSSVPITTTTDSSSSGAAIPVHKMIAHLRDEEEEEEEEDEDEPSMSSTMVTNL

STTEPVVQAPLVEDIPVSMVASQTPDFSALMAGAKGMLQYVYSKGTVLEKTVEYVRELIM

QNEQLVATAKLAEKSASALQILQNQITVLEKENTFLRAQMVQLGIDTSSTALSGARSLLS

NPLAQSLLNTQVTPPPPPPTTNTSQLLVSLAQTLTSNPLLASLSQLNNNPSSTSAVSPVV

SNESPVTASGQSSQLVPPNSQAAILMNSLVQTLATIANSNTPAPPPPPQSSGLSASAAMS

LLNSLMMAQNALSATTTHTTQAIPLSSHPSASPTPASLLVSPHAQDNNKITPPM

>Amq_1|geneid_v1.2_predicted_protein_1|776_AA

MSRGGDGSDFSDENGGRGGEYLQRGDDPGDLHQSKRLKLGGGDSPTRILSLHTDWVQREP

EDRATTAYVGGDPSDGPIPVPQSDFEIEINSTGQEGNGGSPTQESGITLTSASITIEPRI

LTIDSRLPSSVPITTTTDSSSSGAAIPVHKMIAHLRDEEEEEEEEDEDEPSMSSTMVTNL

STTEPVVQAPLVEDIPVSMVASQTPDFSALMAGAKGMLQYVYSKGTVLEKTVEYVRELIM

QNEQLVATAKLAEKSASALQILQNQITVLEKENTFLRAQMVQLGIDTSSTALSGARSLLS

NPLAQSLLNTQVTPPPPPPTTNTSQLLVSLAQTLTSNPLLASLSQLNNNPSSTSAVSPVV

SNESPRIGSAAVSALPLLSYQLKYLFNIMTSMNWFCRELKNRDKLAHEGNHNYEKTISCN

TLMLNDSQLYQNINAELEVSIHRIPDQPIQLYSGGSVVLTCTVTTSSPNITVTVEWFKSL

QSLQHVTNSIHLSPPYRLQEHIWQSSIDLRVLTVQFSGWYYCLATTVDGSSTQAAYYQIN

VEESGNEISDITLHDTYSIGNKATVELSVSPTSLYAQVPFTVTSILHLPAVISPQVRVEW

YRNGMKKNNDDVRTFFVNATTVNSVLQVTEPFPASGIAYTCVFNAEMYGTSIFSLNGSTT

GLSIESKPTPQVTILSNEPSTPSSSYSVTCLALVLVNGTLKLTWKKDGLLVKSVDNVQLG

QPMKTHNSTSLRIRFVNGISQEDEGRYICSASLEMKEYDTILTKTASINILTPSK*

**>Amq10**

GCTGTTACTAAATATATCAGTACATTATGCTTTAAAGGGTGTTACTAAATATAGCAACAAGTCCTAAAAATAGCAACAAACCTCAGTTTTAAGAGGATAGTTCCTGAGATCACAGTGATATATCATTCCAGGAGTGAGGAATGACATGAAGCGATAAAATATCTCGGACTGATTCCGTTTACCGGAATAGCCGGTCACTGATCCCATGTCAAGTGGAAAGTTAGTTATAACTTTACCATACATATCGTAGAGCATAAGAACTGACTGGAGGGAGGGAGAGGGAGAGAGAGAGGGAGAGAGAGAGGGAGAGAGAGAGAGAGAGGGAGAGAGAGAGAAGAAAGGAAGGAGTAAAGAAATCACAATAGAGAGTGCTAGTGTGAAAGTGTATATGCTCCTACCTTGACATCTCTAAGGAAACAGAGGACCAGGTTATTATCATTGGCAGCTGCTGCCCACTCCAGGACATCAACCTCTCCACCTGGCACCAACACACGCCAATCAGACTAAATAATGTATTTTAAATAATAATACTAATATTAATGTACATGTAATCTGTAATAATAATAATATTAATGTACGTGTATGTGTACAGCCATGCATGAAACTGAAATAATGATTAGAGAGAGTTCTATGTGTGTGTGTGTGTGTGTGTGTGTTCCTTGCCTTGTTAGGTTTGTTGATGTCAACATTGATCAGTTGATATTTAGGTGCTTGATAATCAGTCTTTAGTGTAAATACAGTACCATCATTAGCAATGAACTGATAGAGGAAGAGAGAGAGAGAGAGAGAGAGAATAATAAAAAAGGAACGCGAGGATTTTTTGGTTTACTTACATCATATGAAGACTCAAAATCATCATCAATCAGTTTCACCATTGGCAATATGCCTACAATTCATTTATTATACATGTACATGTACACGTAATACAAAATGTATCGCAATGAAATCAATATTCTATCTCTCTCTCATCCCCCCCTTTCTCTCTCCCCCCTCTCTCTCACCCCCCCCTCTCTCTCTCACCCCCCCTCTCTCTCTCTCTCTTCCACTCCCCCTCTCTTTCTCTCTCCCCCCTCTCTCTCCCCCCTCTCTCCCTCTCTCTCTCTCCCGCTCTCCCTTCTAATTCTTGTACATGTGTACCTTCAATCTTATATCCTACAGTTTCCAAGTCGCAGTAATAGAGTTTGTTCTTTGGTTCAGCTCCCCTCACTACATACAAGATGAGGTAGCGTCCTTCATCGCTTAACTCACACGAACTGAGGGAGACAAACAAAAAGAAATGAAAGATAATATTTGTAATAAGACATTTGAGAAGACACATAAATGTACGTGTACACGTAGCTTCTACCCAACGGCAATTAGAGAAGAAAACAATGTAAAAATTAAAATTTATTACAGTGACATGAACCCTATAATTAGCCTATTAATTGTAACTAAACATGTATAATGTACATGTACATGTAATACAAAGCTTATTATGTAGCTATTATCATTATCAACAATTACTTTAGCCAGTCTGGGTGGTCAGGAAACTCGTAACAAAGGACATCATCTGACTGGTCATTGCCAAGGAGATGATAATAGAGCTGTAAGAAGGAAAGGGGGCATAGAGGAGGAGGAGAGGGGGAGAGAAACAATGAGACATAATGATAAAGAAAATACACTATAATCAAAACAAATAATCGAATTAATAATAATAATAATAATAATAATAATAATAATAATAACCTTTTGATTTAATACTTGAGATGTTTCAGTCCCTGCAGATTTAGTATCAGGCTCTGGGAATCTCTATAATAATAAAATAATAAATAAATAATAAAAAGTTAAAGTAGGCATTATAAAGATTAGGAATGATAGAAATTCCCTTGCTTTGTACTAGATGTGAACCACATATGAACTACATGTAGTTCCTTAACTACCAATTCAGTATCCGTACAATACATGTATGTGTGGTGCTTTTCCATTTAAAGGATAAAAAAGAGCATGTAGCCATGTACCACGTACGTACTACATACATGTACATATACATGTACATGTACTTATGTATCATACATATACTTGTACATTTAGTATATACTGTATATATGCATAATGTGGAAGCTTACTGCATGTACGCGTACATGTACACAATAAGCTGTATATCAATCATTACGATATTGCTCAAGTTCAGGAGTACACAATTTCCCCTATCATAAGAAAAATCCTACACATAGGAACCGGTACATCAAAAAGGAACTGGATCTATTCCAGGTGACTGATTAGTACTGCTTGTATGTACATGTACATGTACACGTAGTTCTAGCGAAGGCTATAGTAATAGTGAAACAGTTTATGATGAAACTGAGTTGAATGTGGGCATGCACAGAATATCATTCTAAGTATTCATAACATACAAGCATAGCCAATTAATTATACTATATTAACTTCTACAAACTAGTGACTGTACATATATAAAGTATTATACAGATTGTTATAAAGGATATATTGTGGTTACCATACGACTTAGATATAGTTGTACTATGTTACAGCTGGGGATTCTTATACCGAAAGCATAGACAGGAGCCATAAGTGGTACACACACGTGTGTGCATTTTGAAAGGTTTGTGTTGAAGGATTACGTAAGGCTTGATTCATCAATGCTTGCTCATAATCTACTATCCAATAATGGAGGACTAGAGCAACAGCTTTCATGTGGTTACTGATAATTGAATAACAAAATAGTAATATTCCCACCACCGGCAAAAGGAGCCATATACTATATTCTAACGACAAAACAAATACTATTATTACTATCATGTGTGTTTGTGTGTATATGCGTGTCAGTTATGGTGAGATCAGATAGAAAATTATTAGCAGGGGGAGTTTTTGTACAAGTTGTGAAATAGGTAGGTTAATACCACAGAACGAGACTCTGCATGGCTACATAGCTAATGCGGAGTTTTATAGCATAGCCGAATCATCTCAAATTAAATAATAAAGCCAAAATTCCATTAATATACATGTAAGATATAGTACTATCAAATTATGTTGATGATATAACATACATGTACATGTACATCTAATACGTGAGCGGGCACATTTTCACAGCCATAGTGAGACGGACAATACCAAGAGAGAATTTTATTGTCCAATAGACGTACTACATTATACTAGCAGTGGTCAGACTAAGGAAGAGCCTGTCTAGAGGTCAGGGAAGTGATTTACTCAAGCATGTAATACAGATACGGAGCTGGCAACCAAAAGGTGTTTCTGGTCACCAAAAGGTGTGAAGTACAACTATACACTGTACACAATACATGCACCACAAATACCTGTACATGCATATACAGTACTGTACGGGCACATGTACATGTACAATAATACAATAACCCACAACACAAGTGTATGTATAGACAATAATAATGATAGAAGCCTCTCCTTGGCTTACAAGTGAGTCTGTGGTGTCTCACTTGATAAAAGAAGCCTTTGTTATCATGAGTCCATGCTATGCTTGAGAATTTGACCCACTTCAGTTCATCCTTCAATAACTCCTTGCTGGCCACATCTTTAACCTGGGTTATTGTAAAAAACACTTTTATTATCTGTGGTCGATCCTATTCTATACACTCTCCATCTCCTTCTGTACAAATGCAACCACTAGAGACTCAGTCTTTCATGTGTATTCATTCACTGTCTGCCAGTCTGTCTACATAATACTCTCTTTGTCTCTCCCCTCTCTCTCTTATCTCTCTCTTTCCCCTGGTTCTAATCCTTCCCTTTACTATGCCTCCTGGTTTCCTACTTAACCTCTCATCAGGTCTATATTGACCAGCTGACCCTCCTTCCATATCAAGATTAAAAAATGATACATTCTTATCTCTTTCTTTTTTCTTTCCTCTCTCTCCCTCTCTCCCTCTCTCTCTCTCTTTCCCTCTCTCTCTCTCTCTCTCTCTCTCTCTCTCTCTCTCTTTGGGGGTGTACTTACTCGTACTGTTGTCCAATCAGACCCACTCTCACTGACAGTGTAAGCACAGTATGCTCCATCCTCAGAGAAGGAGCATTGCCCAAGAGAGATTGTACCATCCTTGGACCAATCATTAGGATCAATGAATACCTCAGGATCAGCATCTATTGTCTCTTGAACATAGAGGACACTGAGAGAGGGAAGAGGGGAGAGAGAGAGAGAGAGATTGATAGAGAAAGTAAGTATTTAATAGTTCCTTGCCAGAGTATCATAATACATATTAATGCATGTGCACAATAGCTGAATGAATTGTATTATTATCATTATTATCACAATTATACTCATCTATCAGCTAGTGTATACATTGTATGTCAATGGACTGACTTTTGATTCTGTAGTCCAGTGTTGTAGTAATAGAAGTATCTGCTCCCTCGCTTAAAAGGGCAAGAATATTTTGGATAATTATGCAACTCTGACATCCTATAATGTACAGATAAGAAATAACTGTAAAGTACAGTACACAAGGCATGACAGATAGTCTGGCACATGATTTGTTACATTTTAATCTTATTACAGTACATATTTCACATGCATACGTTTTGTCTATCCAATTTACTATTATGTACTTGTACATGTACATGTACATGCATTAGAGCACTAAAGAAGGCTCACATGTCCTCGCATGATCAAGTTACACGTGTCAGCTATTACATGTACGTGTACATGTAAATCAATTAATGAATGCATGTAAGTAATATGAGGGATTCATTCTCACAAAGTGAGCTAATAAGAGCTAAACAGAGTGGGCTCTCCAACTTTTGGGAGAAACTTAAAAAATTCGTAAAAAATCCAAACTTGATCGGAATCGGCTCAAACTTTCTATGCAATATAAAAACTGTATATGTCTTTTTAAAAATTATAAAACTGAAGAATTCTCTCCTACCCAATTTTAGTGATTTTTGGGCAAAGTCCCCGTACTATAGTACAGGGACTTTTGCTCAATTTTAGAGCAACTTTAAAAATTCGTAAAAAATCCAAACTTGATCAGAATCAGCTCAAACTTTCTACACAACATAAGAACTTGTATATGCATTAGAAAAAATTATAAAAATGAAGAATTCTCTCCTACCCAATTTTGGTGATTTTTGGCCAAAGTCCCTGTACTATAGTACAGGGACTTTTGCACGATTTTAGAGCAACTTTAAAAATTCGTAAAAAATCCAAACTTGATCTGAATCAGCTCAAACTTTCTATGCAACATAAAAACTTGTATATGTCTTAGAAAAAATTATAAAAATTAAGAATTCTCTCCTACCCAATTTTGGTGATTTTTGGCCAAAGTCCCTGTACTATAGTACAGGGACTTTGGCCTGATTTCGGAGCAACTTCAAAAATTCATTAAAAATCCAAACTTGATCGGAATCAACTCAAACTTTCTACGCAACATAAGTACATGTATACGCATCAGAAAAATATATTAAAAGGAGAATTCTAACCTACCCATTTTAAAATAAAATACCATGTCCCACATGTACAGGTACATAAGAAATCTTTCAAAGTCATTTATGGTGAAAAGCCATATGATTTTAGGTCAACATTTGTAACACACAAAACCGAAAATTTAAAACTCACTTCATCTTTAAAATGATACTAAAAACAGCATTACTCAATTTACCTTTCGTGGAATTTGTCTCTCACGGGAGAGTCTTTAAGGTAAGGGGTAGTAATGGCGTTCTGGGCACGCACAAATTCCCTCGTCTCTTCACTGTCGGGATCTTCCAGCCATCGGTAAGGGTCTGAGACTACTACCCCACCTGCTCTTTCTTCACGGACGCTCTCATCTCGCCTCAATTTTGGGTACTGCCATTCAGCCGCCATTTTGTAGGCGTGGCTCATTAAGGTGGTGGGCGGGGTTTTAAAGTTAATAATTAATTTTTCTCAAAATCATTAATAAATTGAATATCAGTAATTATTATTTTTCTTTTTCTATGGTCTCACTCTCTTGGCGGATTTTTAATAATTGAGCAGTCCACAGCTGCACAGTTGAACTTTTGAAAATATCTAAATTAATTAAAAATTGATTAATTGATAATTTAAAATAAATTATTAAGAGCTACAAAATTTAACAAGATAAGCAATAATACGAATACCTTTTGTTGAATCAAAGAATTGCTGCAGTTCACCCTTTCTACCTTGATCTAAAATAAAATAAAGACAAACAATCCAACTCTTCCTCTTGTCACTGGTTCTCTCTCTCTCCCTCCTCTCTCTCTCTCTCTCTCTCTCTCTTTCTCTCTCTCTCTCTTTCTCTCTCTCTCTCTCTCTCTCCCTCCTCTTTCTCTCTCTCTTTCTCTCCCTCCTCTTTCTCTCTCTCCTCTCTCTTTCTCTCTCTCTCCCTCCTCTTTCTCTCTCTCTCTCTCCCTCCTCTTTCTCTCTCTCTTTCTATTCTTTCCTGTCTCGTATTCATATGCCTGAAATATCATATCAAACTTGTCCAGATCATGAACTAATACTGCCTCTGGTGACAAACACTCTTCATAGTCCTAAAAGAAATTAAATTTACACCAAATCCAAACAACTATACACATGGCGCACGCTCTACAGAACACATGTACATGCATCATGATGTAAGTCACATCTTGAAAGCTTCTATATGCACATAGATAACATAGAGTGGTGATGTATGATGTCTAATTGACTGCAATAGAAGTGAAGCACAAAGCTCTATAAGTTTAATGTACATGTTCGTATGTTGAGTGAATTTATGGAAGTATGTCACAAGTTGCGATAGTTCTAAATCTACATGCATAAGATAGTGAGTGGCTCCCATACAGCTGAAGCATGAAGCTCTTAGCTTAACGTGCCCGCATGAATTGAAATAAGATATTTGCTGCTTATGAAGAAGAAAAAAGGAAGTCAAAAAGAGCTAAACTGATACACAATACGATACAAGATAGTCATAATCTTACCGTCCAGAGCGAGTATAATTCCTGACCAACCTGAAACATAATAATAATAATAAATAAAAGTCGGCCCTTACGTAATCAATCATGTAAAACATTATTAAACATTTGTTAAGTCTGGTACGTGACAATATACAGTTCTGCTATGACTGGGAGCAATGGATAAACAAGTGGTAATGCAATATGTAAAAAAAGCCTTCTTGGGCTTGCCATTGTACACCATTTTAGGAACAATGGAATACAGTCAATAAGATTAGAAAGGCACACGTATACATATAAGCATATCATTCATTACCTCTTTGGGAACAAGCTCAGAAATCTTTACCATAGCATCCTAATAAAAGTAAAATACAGGTACATACATGTAAACACACTATAGGTACCGTACACACACCACCACAGCTGTACTATAAGCACAGCTGTAAGTCAAACATACGCACACACACAAAACTACTTCTATGGCTTACATGTCACAGCAAACACATGTAGACCATGTTCAAGCTGCACGTGGGCCTGTATCCCTGGTTGCCAAGGAGGAAGCCCGATAATATAAAATGCAATGTTGTAGTAGCTGTATCTCCCCCTTCTCCATATACTTCCCCTACTATTGAATTATATTGTCTATACTGTGACCACATTAAAGTGGTCTGCGGTTATGAAAATGAATGAGATGACATAAAAATTTCAGGAACTGAGTCACCACACTGGAATAGCTATGTAGGCAACTGCGTGAGGCAGGGAGGGTTTTTACTTGCTCGTTTTGGGACTTTGGAGGCCCACAAGGTAGAATACCCACCAAAACACAANNNNNNNNNNNNNNNNNNNNNNNNNNNNNNNNNNNNNNNNNNNNNNNNNNNNNNNNNNNNNGAGAGGGTGATCAGATATCATACTTTCTCTCTCTGATGCTTCTCTTCTTTACTGACATTACAAGACGGAGTCAAGTCCCCAACAATACTCTCAGCTAGATCGTGAACCAGTGACATCTTAACACATCTACACATCCAATTAATAATAATAATAATAATAATAATATTTGCACAATGCATAACCAATAATAAAAATACAATTATTTGCAGCACGTAATTAAATTATTCAATAAGGTGCATGCACAATGACACACTTATTGTAATCAATCGAGTTGTCTTTAGAGAAGAGGAAGGACATTAGACTCATTCTGTACATGTGCCCCGCAACTGTCTCTGGCTCTTTGACGTTATGATTAACCCAACCAGTCCTTTTTAAATGCTGAGGGAAAGAAAACAAAAAGAGAAAGAAAGAGAAAGAGAGACAGAAAGACAGAGAGAAAAAGAGACAGAGACAGAGAGAGAGAATTAAAGAGAAAGGAAAAGAGAAGGCAAGTGAGGAAGAAAAGACAAAAGAGAAAAAGGACATAAAAGAGTGTGAAAGAGCAAACAAAGGAGTGACAAAAGGAATGACAATAGAGGAAGGGAGGGAGGGAGGGAGGATGGTGCCAGTAAGCTAGGGATGAAAGGGAAGCCAGTTCATTATAACAGCTGCAGGTTGGCTTGCTGTGGAGTGGATCCTAACACCAATCATTTCATTAGGAGTGAGGCACACATAACGTTATGTAATGACATATAATTTAACACCTTTGATCAATAAGACTGCTACACACACAATCTCACACTTTAATATCTTCAAACAAAAAAGGAGGTATTAAGAGGAAGACAATGTACAAGAGAAAGAGGGAGAGAAAGAGGGAGAGAAAGAGAAAGTGAGAGAGGAGAAGGAGAGAGAGAGAGAGAGAGAAGAGAGAGAGAGAGAGAGAAGAGAGAGAGAGAGAGAGAGGAGGAGAGATCAATGAAAAGTTCAAAAGTATGTCTACCAGTTCTGGTATGTTGATACATAAGTGTGTCAGCGTGTGTGCTCGTGTCTATGTTGTGTCTGCTATGTTTTCCTGTATATATTACCAAACATACACAACAGAGTGTGTGTACGAATGAGTATCAAAGCACCTCTCCAGTCAATATGCCAAATTAGTCATTTCATGAGGTACAACAACAAAGCGTACATAGACATACTTGCTAGTGACGCATTCTTTTTTATATCGGAGTAAACGAGACAATATTTTGCCTGGAATGGAAGGTCTCTTCCTTCGATTCCGAGTAGTTCAAAGTTTTGATGTGCCTGCTAATGCATACAGAATTCAAAATAATTTTGGAGGAAATACGGGCTGGCTGTACTATCAGCCAGAGGCTATTGGCTAATACATGTATTAGGTCAGGCATGGCATCAAATTGCATCATCACGTTTAGTAATAAAATAATAATAATAACAGCATAGGCATGGACACACACAATGATGATACTGCCATTTATAGTAGTGACACTATTACAGGCATTAATATGTGATGAGGTAATTCATGAAATAATGTATACACAATGTATATCTGATGCATCATCAAACCAATAGCGAATAGTGTCAGAGTGACAAGAGAAAGAGAGAGAGAGAGCATCATTTAAAGGGCATCATGTATACTATATCATTCATTACATAATGCATGATGAATGAGGAACTTGCACAACAGTCTCACCAATTGCAACAGTACCCAGCAACTCCAGCAGGTATCACAAGACTAAAAATTTCCTCATGACGTAATATCACATGCTTACAGGGCCACTATTTAGGGCGATACATTCCATACGTGATTATAGTCATTAAGTATAGCCAAAAATGGGAATATAATGAATGAATCCTTCCTCTATTGATAATCAATGAGAAACTGACACCATTGAGCACATTACGGTTGCTATGCTTTGTGTGGTGGAAATACAGATATTATGATGCATGTATTGTACTTATAAAAAGAATATACATGTACATGTATATAATATTTGCATGGTTCAAATTAGATTTTCTTTGTCAGAAAACACACATAAACAAACACAATAAACAGAAACACTGCTGTGAATGAGACGTGACGCATAGCTGGTTATAGTTTACAATCTCAGTCTTTTAGTTCTCATTCCGTGTGGAACAAACATTTGGAACAAAATAAAAAACGCACTCCTCAATGCTAAGTGTACAAAATGGTAGTTTAGAATAAAAATAAACAGAGCTAAAGTCAATTCCTACTTGCTACCGTATAGGAGGAACTTTGGAGGTAGTTAAATTTGGCAAATTCGGCGTTTTGATAGCCATTCGCCAAAATAAAAATCCGTCATTTTGAGCCATTATACGCATGCTCAATGGCACATGGCCACAAATTCGCCAAATTAAAATTCGCCAATCACAAAAGTTTAGCAATTCACCAAATTTAACTCCCTTCAAATTTCCTGCTATATGGTATTATGGTAAATGCATGTGAGTATGTTCATGGGCAGAGAGTTGTGGTACATATGTATGGCTATTCTTTAACCTGATGATGAACACCTTGTATTGTTACAAGGCACATGGTTGCTATAGTGCTCATGAATGAGAGGGTACAGACTAACAAGTCTTTAATCTCAGAGACCGACAAAGAGAGTGACCAGCTGTATCGCCAGGGCAACCGAAGCTCGTGAGAAAATGATTGAGTGGTTTGTCTAGGAACAAGGTACGTGTGTACGGAGGAATAGAGTGAGAACAATGTCAAATGACGTGCAACCAGTTGTGTCTCCGGGGCTTTATACCAAACCATACACTCCTCCTCATGTACATGTAACAATTCGGGCCTGTCTATGCAAGGGGGCCCTCTGGGGGGCTGGGGATAAATAACTACTATCATTGGCAATTGGGATGCTATATTATCAGAAGCGCATTATTAATAAAATTATTTTTTTAATTATTAAATCAATTCACCTTCAATTCAATCATTTTAAATATCATAATAGATCTAATTATTAATAATAGTTAAAGTATAAATATACAAAGTTTATAGACTCTAGGCTAGGCTTACCTACCTTGAGTTTGCTGCATAATTTAAAGAAAGTAAAAAGATTGGGAATGCATTCCTGCTCTTCTTGTTTTCTTTCTTCTGTTAAAGAGTCCGAGTCCTTTTCTGCCATTTTGATAATAATTTTAAATTATTCATGGTACTGTAATTAGATATACGCCGTCAAGTCTGACCAATCAGATTGCAGAACCGACGGTCCGCTGGAGCTATAAAAAGCGAATGCAACAATTTTCTCTTCGTGCCTTCCCCTCTCTGCTCCTATCTCTCTTTCTCTTTCAGCTCTTTCTTGCTCTTTGCGTTGCCTCTTGGCTCTTTTGTTTGTTATCCTGTATCTCCTAACTACCTTCCTTTCTACTTTTAACCTCTCCTTCCTTCTCGCCTGAGAAAATCTACAGACGGTTCCTGCCGCCGACCACTAAATTTTAAAAAGAAGAAACCAACTCTGTTGAACAGCAAATCAAGATGAAGCGTCATCTTAGTGACTCAGAGACTGAAATGAACGAAGAATATGAAGAGTGTTCCAGGTAAAACCATTTTCCAAGTCATGGCGACAGTTCGAAGTGTGAACGTCACCTCTGACTGCATATTGCCGCTACTTGTAGCAACTATGGATGTGCCTTTGAAAACAATCTTGTTACTTGTTCTCTTTCTCTGTCTTTCTTTAGCCCCGACACCGCTCAAGTTGTTTCAAGAAAGAAGAGGCGTGGGGTGAGCCAGAAGCACCTACATACTCATGCATACATCGACACGCAGTCATGCACACAGCTATGTGTTCTTGCAATAACTGTGACATTTGCTAGCCTTGACTTTATTTTCCTCCCTCTCCCTCCCTCATTAGATTATTGAGAAAAGACGTCGTGATCGCATCAACAACTGTCTTATGGAGCTACGCCGACTTGTGCCTGCTGCATTTGAAAAACAGGTGAGAAAATTAGTATTATAGATCACAAACCACATAGTTTATAGTATGCACGTATGGTACTTTGTATTGAAAGAGGGCACATCTTTGTATTGAGAGGGTATATGTATATACGTGTATTAAAAGGGAAAGTTGCATTTGCATCAAAGGGGAAAAAGACACAATGAAACCAGTTGTTTGCCAGATAGCACAATAGCTAAATGAATTATTGATACATGTCGTCCAATTACCAAAACCATGCCTTGTTGTCAGTATGTGTATGGATTGTGCATGCTCACACAGGAGCCCAGAGGCGCCCCTCAGTCTGGTCTGGTTGCATGTGCCCTTAAGTGGTTTCTGACTGTCACCCTTTGAGATTGTTTGATGTTAAGGACAGATGGGAAGTGGCTTGTGTACCGGCAGGGTTTCAGTGCAGTTGTCTGGGGAGGGATCGGCTCTTTGTTGCCATGACTACTAACAGCCTTTTACCCACAGTCCAGCCTCCAGTCTCACCCTATCTCTTCCACCTGTTGTCATTGTATGCCATAGGGAGGGGAGAGAGTATCGCCCACTCTCTCATATTCTCAAAACAAGAGGATAAGGGATCGGGTTACAGAGGGTGCCAGTATTATTTTGACGAGTGACTTTGTGCGGTAGTTTATTGAAAGAGTGTGTGTGTGTGGATACAACCTTTTGTGTGGTCTTAGATTCCTATCATATCATCAGTCCCTGGCATGTGCTGTAGTTGTTGCAATTATAAATACCAAAAAGAATCCCTTTTCTATCATCCAATCCTCTTGTCAGTGTGGCTCCAATGGGTCATTTTGTTTATTTGTGCATATGCCACTAGTCTGGGTCGATTCCCATGGGACATAATAAGGATAACGCACACAAACAGACCCACAAAAGTAAACTAAGCACATGCACTACCACTGCCACACATTATGCAGCACAAAGTGTATATACCAGTATAATTATTGACACACACACACACAGTCTTTAGTAAATAATGCCACCTGGCTAGCTTGTCGGTGTTTGTGTCTACTATAGTTGTTATGGCAGATGATGTTGTGTCGTATATCTCTTAAATTACTATTTTTAGTTCATGTCTACTTATCGCACGCATTCCCTATGCCTTGCTTGTGTTATCTCCTTGTCTCTTGATGGCTCTTGCTGCTTATGCAATTATTCATAATCCACACACACGCACACGCAGATGCTTTGCTTGTAGCTTGTTATGTCGCACGTAAGCGATGATTACCAAATATTTGACAATTAATGACTCGAAGTGCATGCAATTAGACGACTACACGTTGACACTTATTTGATGGTCCTATAGTAGACAGGTGTTCCCTCTCTCTTGCACTCTTGAAATGCGTAGTAATAACAATTGACATATTTTCATTTGTATCTTGCAGGGCTCAGCCAAACTTGAGAAGGCAGAGATACTCCAGATGACCGTTGATCATCTTCGCCACCTCCACCAGACCAGAGACCCAAGAGGTAAGATTAGCTCCTATTAAAGGATTGTGAACAGATGTTATGACATTAGGAATGTCACTGGATGTAATAGAGTGTGTGTGTGTGTGTGTGTGTATAGTAGTTATGTATATGCTTTGGTATTTAAGAGTTTAAATGATCTCATTGCATTTGTATGACTTGCCAGCCTCATAGTCAAAATGTATACATGCTTATATACATGTACATGGTGCTAATGGTGCTTAGTTTATTAGTGATAAAACTGTGGAATGTGTGGCATCCAAAGTGATTAATGGTTTCATAAATTTAAATCATCTCTCTCCTCTCCTAATAGGATTCACTGACCCTCTCTCTGCCTATTCCAACACTCGTGCTTTCCTTGACTACAGGGTAATGGGATTCCGTGAGTGCGTGGCCGAAGTTGCCCGCTACATGACAAACGTTGAAGGCCTTGACATGAAAGACCCCATGAGAGTCCGAGTTCTCAACCACCTTGAGAACTACCTCACTCAGCGTGAGCTTGCCATCAATGCCGCAGTAGCTGCCAGTGCTCAGATGGGTCTTCCCAAACTGTCTCCTTCAGCAACTCTCCCTCCAGCTGGTATTGCCATACAGCACGCTACTGGGCCCTTTATTCCAATCCCAGCCCATAGAGCCTCACCAGACGGCAACCTGTCCCCGCCAACAACGTTTGCTCTCCCGACCCCGGTCATCTCCTTTGCCCCCACCACTGCCACTGTCCCTGTTTTCGGTGCCGCACATATTGCAACTCCTAATGCCCACCCACCCATCATATCCCTTGCCAAGATTGAGGGGTCCGTCCAGGCCACCCCTATTTCGCCAACTAAGAAATTTTGCAGTTCAACCTCTTCCTCACCTTCAGCTACCAAGCAGCCATTCCGCCCCTGGGCAGACCCTACCGACGATGACTAAACCCCTTGACTTTTAAGACCCAACGACTAATAGACAGGACTTTATTTAGACACCGATCGAAACAATTGACTAGGTGTAAACAAAACTGCATGTATGTATACTTGTAACGTGGTACATGCTCTTTTTTTGACAGACTTTATGACTAGACAGGAGGACTCCGCTCCAAAATGTCACTCTACAGGCGTGGAACATACGACCATTACCATCCACCACCTCCTTCCCCCCTCATCTACTACACAGAGAACAACCTCTGGACTAATCCCGAACGATTATACACGCGTACATGTGTATCATTGCGACAAACCCGAGGGAAAGGCGGTGCCACGCCCACACACAAATATTGAAGACACACACAAAATTAAATTCAAGACACAAGCTCAGAGACTCAAATAATAATAAACCGACAACTTGCTTTCCTTCACTCTTCCTCTCTCTCTTTCTTACGGCACAAAACCTACAAACCAAAGACTTTAGACGACGCTCCCAACTTCTGGGACAAGACGTTACTCTTTTTGTTAATTAACGAATACTCTCCCCCAGACCTCCACAACTGGAAACTGGCCGGAGCTGTCTCTTTCCATGAGAGACGACTTTAAAAAAACTTTGCATATTTTTTGCAGTGACGATTTGACGTTTAATATTACGACAAGTTCATTCTAAAGACTATTACTCACCAGACCCCCCAAGTACAAGATCAGAGACTCATTGACGTATTTACATTCTGACTATATCTCATACATGAACTATTGCTAACATAGCATCGACCTATACAGGTGTTTGCCTATTTTATTTTGTTGTGACTCATTACTAAAAGCTGCTTATTATTTTGCCATTATTGTTAGTTAGTGTGTATCACGTAGAGTTAGTATTATTATTATTATTATTCAATATTTATTTTTGCTGATTTTTCCTTGACCATTAATCTAATTTTGTTTGAGTTATGTTGTGATGTTTTTTTTGAAAAAAAATTCAATTTCAACCATTACGTTAACAGTTGCTGCAGTACATCCACTGACACTTGATTAATGACTAACTTTGAGTTGAATACAGTGCCTCATTAATTAATAATAAGAGATTAAAGTTTAAATATAATTGTCTTTTGCTGCCTAAAGTTTATGTAATAACAGTTTCAGTATTACTCATCTGTTGATTCATTAGTTTCAGTACTCTCACTGTTTGATGTGTGGCCTTCTGACTCTTGTGCCACAGTGCTACTAGCATCAGTACTTTTACTACTAGCTAATGCATCTTGAGAAAGAGTGGACTCTAGTTCTGGCATATCGCCTTCCTTTGATGCATAAGTATTCGTATCACCTTCCTTATTATCTTCTTTATTATCTTTACAAGACTCTAAAGCGGTTTCATTATCAGGTTGTAGCGCTTGCATAGGCTTCTGTGTCACAGTCCTGTCCTGATTCTCATCACTCTTTAGAGCAACTGGTGTCGACTCCCTGCATTCTCCTTCCTGTCTTATGCTAGCATTATCATCAGTGTCGTGTGGGGAGTCCTGGAAACGTGTGACAATATCCAAATTATCACTTATTAGCTGGTTCTCTTTGCTCTTGATGACCACAATTGCTTTTCTCCTTCCTTTCATTGCTGTATGAGTGTGGAGCTCATCAATGGCCCATTGCCCCTCAGACTCTGGCACATCATCATTTGAGAAGGAGGGAGAGTGAGAGTAAATGGCAAAGACTGGATTGCTATCTATTGATTCTAAGTCTCCTTCATAAGCACATCGAACTGTTATTCTATAAAGCAGGAGAGAGAGGAGAGAGAGAGGGGGGAGAGAGAGAGGGGTGAGAGAGAGGGAGGGAGAGAGAGGGAGGGAGAGAGAGGGGGGGAGAGAGAGAGGGAGAGAGAGAGGGGAGAGAGAGAGGGAGAGAGGGGGGGAGAGAGAGGGGGAGAGAGAGAGGGAGAGAGAGGGGGAGAGAGAGGGAGGAGAATGGGGAGGGAGGAAAGAAAGGGATAACATACAGTCTGTGCTTATAAACAAGAAATCATCAAACAAAAGAGTATGGTCTAATAAAGTATAATTGGTACATGAATGTAAATAAAAGTACATATGTGTAAACTGTAGCCTCAAACAATGAAGCTGATTCACAGAATAAGTATGCGCAAGTGTGTGTGTTCATTACTTATAAGTCTCTGTTGTCTCTAATCCTTCAATATAGGCCTTGTTTTTACCAGAGCTGTCCTACAGCAAAAACAAACACACATAATCTTGAACTCCTTCCTTGAGTGTGTGTGTGTGTGTGTGTGTGTGTGTGTACACTAAATGAGAGGCGGAAGCCATGCCAAAGTTTCCTTCTTTGCTATCATTTCAGTTTCCTCTTCACAATGACATCACACACACACACACACACACACAAACACACTAGTCTCGCTACACAGACCATTCCCTAGCCTACAGTCATTGGGTCCAATGCCTATAGGCTGGGGAATGGTCTGGCTAGCAAGGCTACACACACACACACACATTGATAAGGATGAGTACACATTACATTGCAAGGAGTATATAACATTAGGAAGAGCATATGTACTCTTGATGGCTCTATCTCTCAATCATTCCTGAGTACTTTCAGGAATATGAAATAAAGCCTGCATATGTTGTACAGTGCACCCACATGTAATAATCATTGTTACATCTTTTCAAAGTTTTGGGGGATCAAAAAAGGTCTCCTAAACTCCCTCTAGGTCTGTAATGATTATAGAAAGGCTAATAGATAGAAGGAGGCAGTAATATACTGGCATTATACGATAAAAGGTGTTTATAAGAGTTAGAATAGAGGGCTCATTAAACTCTAGGACATATGAATGGACAAAAACAATACAATAATAATGAGGCAGGCAGATCTAATTAAGAGAAATGAATTTCAAGCTACAAAGGAGAGCATGACTGGAGCACACAGTATACAAACAAGGACTGGGTCACACTCCTTGAATGATAAAGAGAGAGAGAGTGTATCAGTTAACAGAGGCTGGTATTGAGTGACAGGAGGCAGCAATATCTGATAGACCAAAGAAAAGAATAAGAAGGACGTATGGTTTACACTACCCGAAAGATAATAAGAGATATCCATTACACAATGAATGTCAGAGGCTTATGGATCAAAGCTCCATTAGTATAGACTTACGAAGCCCAGAATGAATGAGAATTGATACACTATACATACAGAGACTCATTAATCATGTGTCTATGAATTAACATACATTATAATGATTCTTTAAATTATAAGAAAAAAATGAATGTGAACCACTCAATACTACAGCTTATAGACTGTAGCTGTATACTTTATGGACATATATGTTAGCAATATAGGTTTAGCTCAATGTACTATACTTATGGACACAATATGATAGGGTTTAGCTCAATGTACATACTCATGCATAAAACAAGCCACTAATTGTAGATTGCATATCCATAAGTGGTGGATTAGTAATCATCATAATCAGAATAGCACTATAATGCATCAAG

>est_CL2933213.5

GTGTGCCTTCCCCTCTCTGCTCCTATCTCTCTTTCTCTTTCAGCTCTTTCTTGCTCTTTGCGTTGCCTCTTGGCTCTTTTGTTTGTTATCCTGTATCTCCTAACTACCTTCCTTTCTACTTTTAACCTCTCCTTCCTTCTCGCCTGAGAAAATCTACAGACGGTTCCTGCCGCCGACCACTAAATTTTAAAAAGAAGAAACCAACTCTGTTGAACAGCAAATCAAGATGAAGCGTCATCTTAGTGACTCAGAGACTGAAATGAACGAAGAATATGAAGAGTGTTCCAGCCCCGACACCGCTCAAGTTGTTTCAAGAAAGAAGAGGCGTGGGATTATTGAGAAAAGACGTCGTGATCGCATCAACAACTGTCTTATGGAGCTACGCCGACTTGTGCCTGCTGCATTTGAAAAACAGGGCTCAGCCAAACTTGAGAAGGCAGAGATACTCCAGATGACCGTTGATCATCTTCGCCACCTCCACCAGACCAGAGACCCAAGAGGATTCACTGACCCTCTCTCTGCCTATTCCAACACTCGTGCTTTCCTTGACTACAGGGTAATGGGATTCCGTGAGTGCGTGGCCGAAGTTGCCCGCTACATGACAAACGTTGAAGGCCTTGACATGAAAGACCCCATGAGAGTCCGAGTTCTCAACCACCTTGAGAACTACCTCACTCAGCGTGAGCTTGCCATCAATGCCGCAGTAGCTGCCAGTGCTCAGATGGGTCTTCCCAAACTGTCTCCTCCAGCAACTCTCCCTCCAGCTGGTATTGCCATACAGCACGCTACTGGGCCCTTTATTCCAATCCCAGCCCATAGAGCCTCACCAGACGGCAACCTGTCCCCGCCAACAACGTTTGCTCTCCCGACCCCGGTCATCTCCTTTGCCCCCACCACTGCCACTGTCCCTGTTTTCGGTGCCGCACATATTGCAACTCCTAATGCCCACCCACCCATCATATCCCTTGCCAAGATTGAGGGGTCCGTCCAGGCCACCCCTATTTCGCCAACTAAGAAATTTTGCAGTTCAACCTCTTCCTCACCTTCAGCTACCAAGCAGCCATTCCGCCCCTGGGCAGACCCTACCGACGATGACTAAACCCCTTGACTTTTAAGACCCGACGACTAATAGACAGGACTTTATTTAGACACCGATCGAAACAGTTGACTAGGTGTAAACAAAACTGCATGTATGTATACTTGTAACGTGGTACATGCTCTTTTTTTGACAGACTTTATGACTAGACAGGAGGACTCCGCTCCAAACTGTCACTCTACAGGCGTGGAACGTACGACCATTACCATCCACTACCTCCTTCCCCCCTCATTTACTACACAGAGAACAACCTCTGGACTAATCCCGAACGATTATACACGCGTGCATGTGTATCATTGCGACAAACCCGAGGGAAAGGCGGTGCCACGCCCACACACAAATATTGAAGACACACACAAAATTAAATTCAAGACACAAGCTCAGAGACTCAAATAATAATAAACCGACAACTTGCTTTCCTTCTCTCTTCTTCTCTCTTTCTTACGGCACAAAACCTACAAACCAAAGACTCAGACGACGCTCCCAACTTCTGGGACAAGACGTTACTCTTTTTGTTAATTAACGAATACTCTCCCCCAGACCTCCACAACTGGAAACTGGCCGGAGCTGTCTCTTTCCATGGGAGACGACTTTAAAAAAACTTTGCATATTTTTTGCAGTGACGATTTGACGTTTAATATTACGACAAGTTCATTCTAAAGACTATTACTCACCAGACCCCCCAAGTACAAGATCAGAGACTCATTGACGTATTTACATTCTGACTATATCTCATACATGAACTATTGCTAACATAGCATCGACCTATACAGGTGTTTGCCTATTTTATTTTGTTGTGACTCATTACTAAAAGCTGCTTATTATTTTGCCATTATTGTTAGTTAGTGTGTATCACGTAGAGTTAGTATTATTATTATTATTATTCAATATTTATTTTTGCTGATTTTTCCTTGACCATTAATCTAATTTTGTTTGAGTTATGTTGTGATGTTTTTTTTGAAAAAAAATTCAATTTCGG

>EST traduction

MKRHLSDSETEMNEEYEECSSPDTAQVVSRKKRRGIIEKRRRDRINNCLM

ELRRLVPAAFEKQGSAKLEKAEILQMTVDHLRHLHQTRDPRGFTDPLSAY

SNTRAFLDYRVMGFRECVAEVARYMTNVEGLDMKDPMRVRVLNHLENYLT

QRELAINAAVAASAQMGLPKLSPPATLPPAGIAIQHATGPFIPIPAHRAS

PDGNLSPPTTFALPTPVISFAPTTATVPVFGAAHIATPNAHPPIISLAKI

EGSVQATPISPTKKFCSSTSSSPSATKQPFRPWADPTDDD

>genscan_predicted_peptide MKRHLSDSETEMNEEYEECSSPDTAQVVSRKKRRGIIEKRRRDRINNCLMELRRLVPAAF

EKQGSAKLEKAEILQMTVDHLRHLHQTRDPRGFTDPLSAYSNTRAFLDYRVMGFRECVAE

VARYMTNVEGLDMKDPMRVRVLNHLENYLTQRELAINAAVAASAQMGLPKLSPSATLPPA

GIAIQHATGPFIPIPAHRASPDGNLSPPTTFALPTPVISFAPTTATVPVFGAAHIATPNA

HPPIISLAKIEGSVQATPISPTKKFCSSTSSSPSATKQPFRPWADPTDDD

>geneid_v1.2_predicted_protein_2|291_AA

MKRHLSDSETEMNEEYEECSSPDTAQVVSRKKRRGIIEKRRRDRINNCLMELRRLVPAAF

EKQGSAKLEKAEILQMTVDHLRHLHQTRDPRGFTDPLSAYSNTRAFLDYRVMGFRECVAE

VARYMTNVEGLDMKDPMRVRVLNHLENYLTQRELAINAAVAASAQMGLPKLSPSATLPPA

GIAIQHATGPFIPIPAHRASPDGNLSPPTTFALPTPVISFAPTTATVPVFGAAHIATPNA

HPPIISLAKIEGSVQATPISPTKKFCSSTSSSPSATKQPFRPWADPTDDD

**>Amq11**

TTCATAGAAGAGAAGTGAGTTAAAACAACTATACTGGATCCCCAGAATTCCTTCAAACAGACGAATTCGGGCTTCTCATTACAAACATACTAATATCAATGGCACCACTGTAAATTTGTGGGATCAGGTGACAATGATAAAAGGAATACATTACCAATGTTCACCTCTGTCATTGATCCAAGGCTAAGAGCAGGCAAAATATTTTTATACTCTACATAATTATTTTAGTTCTTATTATAATTGTGGTTTATGTTTGTAGACCTGCACCTCCTCGTAAAGTGCCTCCACCTAAACCGAAGCGAAGTCGCTGTCATACTCAAATTATTTTTGATCCTCCGAATGATTTGGCACTATTGGTAATTCAATTGTAAATTATCTCCACTCTATTCTTCTTGTTTTTACTTTTTACTTGTTAAAGTATACTCCTTTTTCCTCTTCCCCCACAGGGTAAGTTGGGAGCTAAGCCTTCAGGAAAGACAACTCATCGTAAACTTCCAGAGATACCACTAGCAGTCACTTATAGCAAATCAACTGAAAACCTTTATGAGAGGGTTGATGGACAAGGAAGTACCAAGCGTCAATCTGGTAAAACCTCTCATTCACCGACATCTTCCAATTCAGTTTATGTCAAGTCCAGTCGGAGTCAAAGTATACCTGGGATATTAGTGACACAGGACTCATTCCTCCATCAAAGCAGCAGTGGCTCACCATCACTAGAGAAGCCTAATAAAGCTATTGACAGTGAGAAGCCAATGGTTAGTAATGGCTTGCCAAAAGATAAACCAAAATTGGACTGGGATGTGGAGAGATCCTTACCAGCTCAATTATCACCTTCTTCTAATGGCAGTCATTCTAGCAACAGGACGAGTCTTGAGCAATCCTTACCAGCACGCTATTTGAAGCTATATTCACCTTTAAGGGAATCAATGTATAAGCGCTCATCAGCAATGGAGCTGATAATGCCTAGCCATTTAATGTCTAGGTCACCTACTGAGGAAATACTTACGGCATCAGTGTCTGCAAGGCAAGCTTTTGATCCTGAGAAACCAATTACTGATTCAATGGTAAATGGTACACCCAATGAAAATCCAACATTGAGCTCAGCACGTTCTGTGCCCTCTCATTTGGACACCATCTCTTTGGAAGGCAATGAAGAGGATGAAGAGCAATCTCTCCCAGTCCACTATACTCTGCCACATAAAAAATCTAATTTATCCGATTCAAATCTTTTAGCATCAAAGCCATCACCTCCTAGAGTGGAAAGTCGTGCCTCTCCCAGTGTCACTGATCATTACTCATTGATTTCTGATTCTGATTCTGAGAATTCATCGATCAGTAGTATACATGATGAAAGATCGCGTCGTCTTTCCAGCCCAAGGCCTTTATCACATCATAGCAGATTATCCCCAACCAATGAAAGGAGCTCTTACCTTAAGGCGGTCACCTCATCGCAGCAAATTTCACCCAAATTGAAATCCAAAGAGGTTGAGAATGAGACTGAAACATTATCTCAGTCTGATAGAAAGAAAGTAAGGAAATCTGTATTCTAATATCTAATTCTATCCTTTGATGTTGTAGCTATTAGATGACATTATTGCCTTGTGGAAGAGAGGGAAGGCAATGCAAGCTGCTTCCAAATGCACGCTTGCCTGCTTCTTTACTGGTTCAAGTAATAATGTCCAGTTGTTCATGGATTGTTTGTCAAAATTTTGTGATGACTTGTAAGTACACGTGCTGGTATAGTAATTTGTTCTTATATATGGGTATGCTCATTAATAATGATTATGGTTTGTTTTTAGTAAACATTGTACGATTGGTGTGTGTATCATACTCTTACCATTTGTAAGACAAGTAGTAGTAACTGAGACTGATATCAAGTGAGTTTCTTTCAACCAGTCTGTGTTTGATATTTTACGTGTGTAGGCATATAAAGATAATTTTCAACACTCTGTCTCTTATTACTGAGAATTTTTCATCAAATATAACCAATAGTGAGTATATTATGATCCCATGCTTTTCTTTCTCTGTGATTATGCATAATTCCCATATTTAAACTTTTGTTTCTATTTTTTTCTTTCTAGTAAAGAAAAAGGCGTCTCATACAAGCGATGTAGAGCAAAAGCAGGCTTGCTATCACGAGCTCCATCTTGTATTCTTCTCATTATCAAAAATAGCGATAACTGATGTTGTTCTATCAACTCTTGTGGACAAAATCAAAAATTTATTAAAACCATTTGCCAATCCTCTCTCTTCATTGTGATCATGTTAATCTATTCATGCAATACATAATAATTTTTATTCATGATATCTGTCTGTATAGACTTTTATATTGCAACATTAACGTGATACTATAAAAAGGCTGAGAGAAAGCTAAGAAAGCACAGTCTACTCTGCTGTGGTGAAAATGAAGTCTTTCTTGTTCCTAGTAGCACTAATGATGTGTGCTATAGCCACTGCTATGGCTCCAATTGTAATGGAGAAGAAACCAAACCACTTCAGTTAGTAGCAGCATATAAGCTGGGTTATCCTTCATTTCTTCTCTCTAAACAGAGCAGTATAAAGTAATGTCTCATGGTGATCTAACTGATCAAAATAAGGACAGGAAGGCTTCCATTGGTTCAGGTTGGTGGACACTTGGAGAGAAAAAGCAGCCAAAAGACACGAAAGGAGAACCTGAAGACAATGAGCCTGGTGATGTACCAGATGTCTGGCGCCCAAAACCAATTGTAAAAAGGAAAATATACAGTAAGACTCTACCATTTTCATTATTGTATATGCATCAATGCTTATAGATGAATGTTATCATCCAGTCTCTAATCAACGTATACATCATGGATCATATTCTTATCACAAAGAATCCTGCCTCATGTGCATGTGTCCGTTTGGTCGTATGAAATGCGAGTATGCCTGTTGATGTCCAATTGAGTTGCTCCATGCAGTGAAACCTTCGCTATATTCAACACCACCATTATACTACATAGTATAGCTACTCCATAATAGTACATTTTATCAACTATTGTTCTTTTTATGTAATTCTTAATTGGCTTTGAGAGCCTATTGAGAGAAATGAATGCAACCAGATGCAGTACATTCTACAAAGATCTGAACACAGGTGTCTGTGGCTAGGCTAGCTCGCCCACGCATAATCATCACATGATGGGCGTGGCTAATAATGTGCTATGAATCTAATTACAGGTTGACTGACAAAAGTTTAGTAATGGACTGGGGATTTGTGAAGGGTTACGTTAGGAGCTGGCTTGATTGGGCGGTTGAATATGTAAGAGATGACCCATACGACTTTATCTCCAGATGTAAGTTCAGCTAGCCTGCAATATATAAGATAATATTTGTTTTAAAGCTTGAATTTATCATATTTTTCATAAAATCTTCCCTTGTTCCTCCCTTTCTTCCTCTTGTGTAGTATTTATGATGTTGCTCCCAATGTTTATAATCAGTGGTTTATTATCTTGTTATTTAATTCGTGAGATAGATAGGGAAAATAAAAGGAAAGAAAAACAAGGTATAATAAGTTTATTTAGCCAAGGGGCATAATTTGTATGGGGGGGGGGGGATACAGCTAAATCATGAATCCCACTTAGTAAGGTAGCATTGCTAATAGACTTTGGCCTGATTTTGGAGCAACTTTAAAAATTTGTAAAAAGACCAATAATTGAGGAATCAGCTCAAACTTTCTACGCAACATAAGAACATGTATGTGTATCAGATAGAACTATAAAATTGAGAATTCTTTCCTTGTCATTTTTGTAAAAATTTGGTCTGGGACCATGCATTATAGTACAGGGACTTTGGCCCGATTTTGGAGCAACTTTTAAAATTTATAAAAATCTTATTTTGGTTGGAATCAACTCAAAAATGGTAAATCGGAGGGAAAATGATTATGCTGATGCATGTTCAATGACTTGTGGCTACAAATTCACTAATATAAAAGCTACCAATGCATTGCAATCATAGAATCACTAATATTAAGCACCTGCCAAATATAATTTTCCCGCTATAAATTATAGTCTATGAATGTGGTTTATTTACTGCAGAAAACAAGAAGAAAGCTATCAAGAAGGCTACACAGGGAAAAGATAAACAACCAAAGGAAAAACCTAAGAAAAAGAAGAAACAATCATAAAATATAGTGGAAGAGTTTATATTTTCCGATATTTAAATTAAATTTATGATATGAAATTATTGCAAGAAAGATGGAAATTGCATACTAGACTAGATTTTTTACAGTAATCTATCTCAAATTATACAATTATTATACTATGATAGTAAAATTTATCCAGAATTTTTATAAAAATAGTGTTCACTCCGACACAATTTTGAACATTTTAATAACCATTAATAACTAGTGTTTCAGTTAATTAAGTAGTTATTAAGTCCACATGGTGTCACGTATCCACGTGATGCTTTGATGAATTTCAGATTGTTGAAGTCTACCAGGAAGAAGTGAGGGCTTTTACTTTAGCTGGACCCAGCGAGAGACCCAGATATTACAAAAGAGCCGAGAAAACCTTTTTAAAGAGCTGAATAAAGACATGAGCGATGACGGGGAAATTGACGTTGAGAGCGACGACGTAGGAGAAGGAGGTGACGAGTCCCGTGCTGCGGGCGGGAAG:GTAAGAACCCTTTACTAGGGACCATTGGCACTTAGTACCAACGTAGAATAAGAGTTTGTATGTGTATTACTGGTCGTTAGAGACTTATCAGTCAAATGAGATTTGAAATAGTGGGTGTAAAGTGCTGAGGTGGCAATTTTTGTCACAAGTGTGGGTGTGTTTCTCTCCTCTAACTTTTTCTTTTTTTAAATTTTTTCTACGTCAGAGTTTAGATAAAAGAGCACATCACAATGCTCTGGAGAGAAAGAGGCGTGATCACATCAAAGACTCTTTTACGAACCTTCGAGACTGCATTCCATCTCTGTCTGGAGAAAAGGTATCCTGAGGGGGAACAGTCATACAAGAGAATTGTCATATTTTAAAAATTTAATGTACGTTTAGGTTTCAAGGGCTCATGTCCTTAATAAGGCAACGGAATACATACGACAAATGCAGAGAAATAGCTCAGTACGGACAGTAGAGATAGACGAGCTTAGAAAAAAGAATGAGATTCTTGAAGAACAAGGTAATACAATTGAGGAAAGAATGAGAGAGAATGAATAACATGAGAGAGAGGGGGGAATGACAAGGAATAGAAGAGGAAATGAAGTTGTAAAGTGTAGAAGAATAACAAACTGGGAAAAAGAAACTGTATAATGTTAGAACTAATGTCTTTGGGTGAGTGGGTTTAAAGAAAGAGGGAGAGGTGGAAAGAGAGCTGGAGGGGGAATAAGGATGAAAAGGAGATACATGGGGGTGGGTGGGTATACATATGCAAAGTGTGTACAATAGTATTCATGGTGTTAGTTGATACAGGTACAAGTGCACATTCCTTATAGTTAATGGATTAGAACAAGCACAAGAAACTGGTGCCCTTTCTTCACCTGAAGATCTCATTGCTAGTATTGCTGAGAAAGTCCAGCAAAGACAGCTTCTTAGGCAGCAACAGCAGCAGCAGGCAAATTCTAACAAGGCCAATAAAGTACAGGTAAGTTATTAGGGGACACTCCTTTTTGATCATTTTTATTATAATTTTTTGCTTGTAGCTGGAACCTTCTGCTGACACTAATAATGTAGTAACTGTGGGCCCCAAGGATAAACCTAATGTGAAGTATTAACATTAGTAACTGATACAGTAGAATCTTTATTTATCACACAATTTTGTATCATATTTAAGTTAAATGTATGATTAAGCATTCTGTTTGATGCCTGTCTTCTGTATGTTAGCATGATGTTGAGTATGTTTGAATTTACTTTGAACTTGAATAGAGTCAAGTGGTAATGGTTTTGTGTGTGAAATTCAATCATGGGCGAGGCATCAATATTTCTCACCACATGAATTGTTATTCATTTTGTGTTTATTAACAATACACAAACTCATTTTCAATGTTTCCCCCTCTCTCACACACACACTCTCCCTTTCCTTTTACTTATTAATAGAAACCAATACTCCTGTTAGTAGTGCAGCTGCTAGTGTCACTCCATCTTCAGCTCCTGCTACTAATGATCAAGCAAACTCTTTTAATATACTTGCCAACAACATTAGGACGACTCAGAACCTTCTCTCGGCACTGGCAAAGAACCTTCCGAGTACTACCAACAATCAAATGAGAGCTATAGCTGCTCGTGTTCTAGCATCACCAGTCAGACCACAAGGTAATATTTATAAATATCAATTAATTCTGTAATTGAAGTACGATTTTGGCACATGATAATTGAACCTTTTTTTTAAATTAAGGAGGATCTGATTATGATTGCTATTATTATTGTTATCATCATTAGAATTTTGCAGGTTGTTTTTTGATTTTAATAAACAGCCACATATCTTGTAGAGGCTATGTTTGCTAACTTTCCTACAAGTTATGTGCACCGACCAACAAAGTATAGTCAGTCATGTTACAGCCTGCATTTCTCTACTTGCATGTGTCATTTTTGGCTATGCCATCTGTTTGCAGTCCATTTGTATGTACTTCATTTTTGGCTGGTAATACTAATATCCATTAACTGGAATTCCTCATATTTTATGAGTCGCATTTTAATGTCTTCATAACTGTGGTCTCCCTATGTTGACCAATGATGTGGTAAATGTGGGGAATTTCTGCTAGCACACATGTAAAGATCTAATTGCATCTTTTAAACCATAGGCATGCCTCCAGCTTCAATACCAAGAGGTGTTGTGACAGCAACCACTGTTCCACAATTGACTGAGCAGCTTCCCAAAGCAGTAGTGACTCCCCCTCCTCTCCCTCCACCTGTCTCCGCGTCGCAAGAGGCAGTTACTACCACTGTCTCGGCTGGAGGCAGTAAATAGTCAAACAAGTACAGCAACTACTGAAGCAAGTGAGTTTATTGTGTTTGATGAGGTCGTTTATTAACGAGTGTTTGTGGTAGGTGTCGTCTCGTTACAAGGGAAGGAGACACAGACTGATAGTGAAGATCAACCACCGACTAAGCGTTTCAAGGCATCTGCGACCGTGTCCGAGTAGTCTTGTCCTTATGTCTTATATAGTAGTGCTCTCTCTGCCTTTTTACTGTCCCTTATAGGACAGCTTGGGATTATGAACTACACTCACACACCATTTGCATACACATGCACAAAAACAGTTATTTTATTACTTGAATTTAACAAAAAAAAAAAACAACTAAAAAGGCATGTAAATTGGGGGGGGGGGGTGGTTTAAATGTTTTATGGGCGTGGCTCAGGTGGAATTAAGCAACGAGGGTTTACAATCTTTTCTTATTATCTTCTCTTACTCTCGATCTAATGTCCGTTCCTCCTCGAGTCCCCACACTCCAGTACACCCTCTCTCACGTCGCCCCTGACAAAGCAGCTGAGATACGCTCAGCAGTGACCGGGGCACTGGTATACTGTAGCCAGACTGGACTCTACTCCTTCGAGCACAGGGTAGAGACAACCTTCGTGCTACCATGGGACCTGAATCCACAAGTACAACCACAGAGACTGAGCAAGTAATTCAATGACTCTCTTTTTTACTTGAATTTTTACTCATGATTTGATTATTATTATTATTAATTTTATTTATTTATTTATGTATTTCTCTCCATTTTCCCCTCCTTCTTCGTCAGGGAGGTTCGTGAGGGCATAAGGTTGGAGTTATTGGATCCAGACACCCTCTTACACTTGGAGGGCTGTGGGGGCATTAATTGGAGTAACTTGTCATCGTGGCAGTCGAGACCGGCCCAAGGGGGTACCTGGGAGTACATTAATGATGTCATTTGTGGTGAGTTGTATGTGTATGATATACTGTTGCGTAATATGATTTATTGGCAAGAGGTATATTTAGGAATTATTTTGTCTGTGTATAAGTAATTGGTGAACTTCATCTCAAACTTTATATTGGTGTCTTCTGGTGATTTAGTAGCTACGTGTCGTGATTGATTCACAAAATTTTAATTCAAGTATATATATCACCATTGAAATCAGATTCCGCATGCCCTCAATCATGTTTGAATTTAACACTTATGTTAGCAGATATGTTTTCTGAAGGAAATTAGTTAGTAGCTTGGCATGGTTTACTTGAATATAAAGTACACATACATGTATAACAGTGACTGAAATGTAACAGTGAATGGCCAGTTGTTATTGTTATCACTGCTGTATTGATTGAGCAGATAAATTCATTCTTTCTACACAATCAATCATAATATCCCCATTCATTTTATCTTCATATAAATGCATGTATAGGTCACATTGAGGCTGGTTACATTAGACCCCACAATGAGTATTCCTTTGCGATGCATAACAATGTACATATTCAACAGTTCCAAGTCAACTATATCAATGGCTCATTGGCTAATGTCAATACGCAGGAGTTGTATCAAATCAGACGATTGGCCTTTGCTCCATTAATGCCGATGAAAGTGAGTCCTTGTTTGATGAAATTTTCCACATCATAGTACAATCGATAGCATTTTTGCTGTATTTTGTATTTCACAAATACATTTTAAATTTGATTTTCATGCAATTAGTTTTTGCGAATCTTACAATTTTTATCCTTGAAAAACAGACAGTATGATTATGTTGCCAATCTAATTGGATTTTTAAATGAGTAGCCAACACTTAGTGGTTGTGATGCCAAAGCATTACTGGTACTAATAATCCTAATAGCTCCTGTAGTATAGTCATTATTGCATTCTTCCTTTCCATAGACTGAGGCTGGTTCTAGTTCATTGTTTCATGCTGCCTCTTTAGGATTATGGGGTGTACATGACAGGAATCAGCTACTAAAGGAAGCTGTAACATGCACTCTTACTAACCAGCAGGCGCAGATTAATCTATACGATAGATGGAACACTGTCAGGTCTCGGTCTTTACCATCTGGCTCTCATAAATCAGTGGGTATTGTAAGTATTGCCTATCCATTGTCTTTACTCTTGGACCAATGTATACTGTCCAGTGTTTGTTACATGTAGTGCAGTTTATACAGGAGTGTCTAGCATGTTGCATGAAACAGAAATGTTTCTTATTTCATGCAGTTTGTTACATGTCTGTATAAACTGGTTGCATGCATCCAACCAGCTGCATGTTATCCTTTAAACATTATCATTATAAAATGTCCCAACTCTTGTGTTTTGCAGACAGACACTTTCAATAGACATTGGTCTTATTAGTGCTCTCTTTCTCGTGTTGCTTCACTCAATCTTTCCTTCTCTCTTTCATTTCTCTTTTCTTTTTTAGCAGTTTGCTCAGTGGGAGGCGGCTAAAGACAATATCGTCGGAGCTCCACAGCTTCACATATTCACTCTCTCAAATATCATCCAGAGACCAATAATAGTCTATTCACACCCTGAGAGTGAAGTGGGGTAAGCCTACAGGGACATAACGCACACGCAAGAAAACTTTGAGCAGTTGGA

>PCR product

TCAGATTGTTGAAGTCTACCAGGAAGAAGTGAGGGCTCTTACTTTAGCTGGACCCAGCGAGAGACCCAGATATTACAAAAGAGCCGAGAAAACCTTTTTAAAGAGCTGAATAAAGACATGAGCGATGACGGGGAAATTGACGTTGAGAGCGACGACGTAGGAGAAGGAGGTGACGAGTCCCGTGCTGCGGGCGGGAAGAGTCTAGATAAAAGAGCACATCACAATGCTCTGGAGAGAAAGAGGCGTGATCACATCAAAGACTCTTTTACGAACCTTCGAGACTGCATTCCATCTCTGTCTGGAGAAAAGGTTTCAAGGGCTCATGTCCTTAATAAGGCAACGGAATACATACGACAAATGCAGAGAAATAGCTCAGTACGGACAGTAGAGATAGACGAGCTTAGAAAAAAGAATGAGATACTTGAAGAACAAGTTAATGGATTAGAACAAGCACAAGAAACTGGTGCCCTTTCTTCACCTGAAGATCTCATTGCTAGTATTGCTGAGAAAGTCCAGCAAAGACAGCTTCTTAGGCAGCAACAGCAGCAGCAGGCAAATTCTAACAAGGCCAATAAAGTACAGCTGGAACCTTCTGCTGATACTAATAATGTAGTAACTGTTGGCCCCAAGGATAAGCCTAATGAAACCAATACTCCTGGTAGTAGTGCAGCTGCTTGTGTG

>predicted_cds_PCR

MSDDGEIDVESDDVGEGGDESRAAGGKSLDKRAHHNALERKRRDHIKDSFTNLRDCIPSLSGEKVSRAHVLNKATEYIRQMQRNSSVRTVEIDELRKKNEILEEQVNGLEQAQETGALSSPEDLIASIAEKVQQRQLLRQQ

QQQQANSNKANKVQLEPSADTNNVVTVGPKDKPNETNTPGSSAAAC

Note: Proteins predicted by Genscan and Geneid do not contain the bHLH domain

**>Amq12**

TTGTATGTAAATTTGTATGTATGGAAAACTTCATACATAAGTAAGTCTATCAGGGGTAGTTAACTTTTGTTTTTATTAAATGTGAGCAAACAAATAAAAAATTTATTATTAAATCTATTACACATGTACCTTTAACTTATTTCTATAGGCGATTTTAATCACTCACCTGTTCATCAAGCCCAATCCACAGCACCAGTGTCAGGACTTCACTCAATGTCATCAACTGAACATTATTTACAGCACCCAACACCTGGTGTCCCTGTTAAATCTGAAAGCCCAATCATGATGAGTACTGATCAGCCACTGTCACAAAATGTAAGCCGTTAAGCCTGTTATGTTGATTTTTTTAATTGCATGGTTTTTTGTACTCTAGGATCAAGCTCTTATTGATGACATTATGGGTATTGAGGATAAACTGGAGGGATTTACTCCACTTCCTTTTTTTGAGCAACAAATGATGATGCCACAAACTGTAAGCCGTTATAATTAAAATACATGAATACCCTACATGTAAATGTACATGTGTACATTATTGTTGCCTTTTGTTGTTTTTGAAGTTCTTACTATACACTGTATTTTTCCTAAAAATTCTATTATGATGAAGCAAATTACTTGTACATGTACATTTTAAAGTGAAATGTACACATGATGAGTACTTCTAGTACATATTGTACATGACTATATGCATGTACATGTTAATGCAATTGGTTACATGCACATGTACATGTACATTAAATTTCAATTAATCTACACCATAGCTATTGAGGCACTTCCATTTGCTTGTGTGTAAGTAAATGTGCACTGTTTTTGATATTTTAAGGTTGATTCTGTAGAAAAGAAACCATTATGCAGCATACACTGCATGTATGCCGACAAAATTGTTAATGGCTTTGAGTGTTGAGCCATTATCAAGTTCTTGTCTTGATGATTTCATTGAATTACCTCATACATGTACATGCAGAAACTGCAAACAATATACACACATTGTAGCTACTGCTTGATAATTTCAAATTTGCTAAGTTGATTCGGTTTTTAATTAAATATTGTGCTTGTACATGTACATGTACAATTGTCTGTAAATGCACTAGTACATGTGAGTGCTTTTTCATTTAATATTAAATAATTATTTATTTGATTTATATTGATTTAGCTTCCTGTAAGTAACAACTTACTTGACATGTTTGGAGGTGGTGCAGCTTCCTCTCATCAAATGTTTGTACCGCAAATTTCATCATCATGTCCTCCTGAAGTTAAGGAAGAGAAAATGAGAGAAGCCTGTAGTTTTGGTATAAATCCTAGTGAGTTGTCATTGTTACCAATAGCTTCTATTGTATTAATTTTTATTTGAAGATGCTAGTATTGATATAAAACAGTTTGAAAAGGAGAGAATAAAGAAAAACAATCACAACATGAGTAAGTTCACGCATTTGCTTTAAAACTTACTATACATGTATATTATGTGTAGTGCTGTTATGTGTACTCTAAACAGAGTGTTTTTATGTACTACTTGTATATACTTTATTCTGAAATTTAAATGTTTGCAAATTTATAATTTGAAATTTAGCTTTTATACATATTTATACACATGTATTTAATGGGTGAAAAATCTCGTGATGTGGCAGCATTTCGTGTTTGCTAATAAAAAATTTGCAATTTTTAATGTAATTATTGTACCTACATGTAATTTACATCTGTATACTATAATTAGCATGCTTTTTTGGATTTTAATTTACATGTACACCTGTAGTTAATCCAAAATGTGAAAACTGCAAAATTGAATCCCCACGAAATGTTCATGTATGGCGTATTGAGGATATATATACATGTAAAAAAATTTGCCACATGCTTGATCACATGATATGGTAAGTATGTTTAAATAAATACACACTCATGCAGCAGTCTTCTATAGGGCTGTTGGATACTCTCTTTAGGTTATATGTACTCTTGGTAATTGGTTGCACTAAATTTAGTGAATAGATGGAAACATGCACAATGCAGCTAAAATTTATTAGTTTACAGATTTTTAAAATTTTATTTAAGTAAAAAGATCCTTTTTATTTTGGCTGTTATGTTGTTGACTGGAAATCATGTAAATGAATCATAAACGAAGTACAGCCAGCATCCTGTGCTCACTGCCAAAAAGAAGTAATAGTAAAAACAACAGCAACATAGTGTACATGTACATTGTGTATATCGTATTGCCGAAACTTTGAGGGATTGAAGTCATTTAACTTTCGCAGATCTGCCCAAAAAACTTTTTTGTGGATTTAATTTTTGCAAATGAAAAGTATTCCACTACATGTATCTTGTAAAAGGTTATTGACCTGGAAAGTTTTCCAGGCATATGTCCATAATGAAGAGTAAATACAAGTACTTCATGCATGTATGTACATATTTGTGTTCTTCTAAAATATACTGTACTTTTTCTTTTTTTTGTGGTACATCACCGTACATGCGCAGATTTCTTCCCTTTTTCTTCTTCTGCTTCACTCACTTCTTTTCAATATTGTTTATAATGATGATGATGATAATAATAATAGTAATAATAATAACATTCAATCCATTGATAGAAACCCTGCTTACATGTGTATTATTTATTTATAGTTGAAAGAAGACGAAGATTTAATATAAATGACAGAATAAAGGAATTAGGAATGCTACTACCGTCGTCGGAAAGGTAGAGCATAATGCATTTTTTCGTGCATTTATAATTTTTGTTCCTCACCCTATCTGTGTTATTTTTATTCTTTTACATTAATTGTTTACAATATACATGTACATGTACATGTATGTAAGTGTCTTAATTTTATACTGTATGCTGTATGTACTGATACCGATAGCCTTTACATTGTAAGTGAGATGTTATTTTCTAAATTTCTCCTGTAGTGAAGCACGCCAGAATAAAGGTAGCATTCTTAAAGCTAGTGTTGACTATATTCGCAAACTTCAAAGAGATGTTGAAAGGCTTAAGATACAGGATGCTAAACAAAGGCAACTTGAAGAAACTAACAGACAAATGAGATTAAGAATTCAAGTAAGTGCATGTACATGCATGTATGCATTTACATGTAAGTCTGCACCTACATGTACATGTACATTGTAAGTTTTACAACTACATTTACATGTATGAAGTTAAGAAAATTTCCTATAGTGAACATTAAGGATTAAGGATATGTTTATGACTATGCATGCATTTATAAATTTAATTGCTTTTGCTACACATACAGTGTACATGTATGACTATTGGTGATTAATTTATTTTTTGTTTTGCTGGTTTTTGCTATATTAAGTTCTGTCATACATGCATGTACAGTACGTAATATACATGTATGTGTTATTGTAAATTGGGCTCACTACAATCCGTTTCATTTCTGCTGATTGATTTAAGTATCTAAATGCATGTACATTGAACCTGTATTAATCTTATTGCTATTGATTTATAAATGTACATGTACATATACATGTACATGTACATTTATGTACTTTAGACCACTACTTAAAAGTTCAACTGGTATATATCAAGTATGAGTTGTTGTTCCGTGCATCCTGCCTTTTTACTTGCCAGAAGAGGTTCTGTGGAATTTGAATGCAATTCATTATTTGTTACTACATAGACTGTATGCCTATCCTCCTTGACAAAAATTTTTGCCTCTTCCTCTGCTTATTGTTCCTATTCCTGTATGGTCAATTATTGATAAAATTTTGGCTCCAAAATCTAATGAATGATGACTATATGTACATTGTTTGGTTGCGAATGTTACATGTATGTACATGTACATGTATATTACTAAATTCAGGAGCGTACATGCAAATGTAACATATGTTGACTTGCATGTCCATGTACTAATGGAGCCATCCTTGTTACAAAGTCACAGCCTGTATCTTTATTGTAACATTTTCTTGTTCAACGTTCATACATTGTACTATGCATTTGTATGCTTTTTAGTAATAAAACTAGGTTCAAAGGCACACAATTCATGTACTCAATGGAGCAGTAGCAAACCCTTTTAGTGTCTTACATGTACATGTACATGTACCTGAGGTTGTTTCCCTTTGAGCTGCTTATCTCTGGTGTATGAGCTAATGTTTTTATAATTGAATAAGCTGTTTGGCATCATGATGATGTCACTTCTATATTCTACATGTACATTTAGATGTATGCATTACATACATGTACATGTAAATGTATTCACTTTTGCAGTAAATGTACAGTAAACTAGGGTAAGGCTGATTCTGAATGAGTCAGTAATAATTATTCTAATATTCTAATGGCTGGTATTTTTTAACTTTTTACTCTACAATTGTTTATGTGCATATACAAGTCTACAGCACACCTGTATACATGTGTATATACATGCATGTGTGTGTATTTACAGGTAATCTGGTATGCATTTAAAATGTACATGTGCATGTAATGTACATGTATTTGTCTAGTATGCTGTATTTTATGTAAATTAACCACTCTCCAAATATTTGATAAGAGTAGCACTCCTACAGTCCTATTAGCAACTACTAGGGAATGCTAGAATGTTTTATGTGTATGCATACAGACTGTACAGGTAAGGCTTACAAGGTGTGTCACTCTAATGGAATTTGTTATCCATGGCCACTGCATTGATTAGAATGTGTCATCACAGATGTTAAATTTATTTGGAACAACTTTTATCACATCATGCCTTTTACTAGCACACAAGATAAAAATGTGCATGCCATGTATGTGTACATATGTGTGTATAATGAATAAGCATGTACAATATGTGTATGTCTGTTATGAAGTACATGTAATAATTATTAATTTTATTTATTTTTAGGAATTAGAATTAACTGCGAGAGCTCATGGCATTCGAACTCCTTCTCTGACTCCTGAGACAAAACAATTAGCTGATGCAGCAGATAAGGTATTAGGTCCTACTACACCACGAGCTCGTTCTCCTGTTAGTTCAACAACTACAACACCTCTGTCTCCTCATGTATTTAGTGGAGTTGATGATTACATGCTACGTGCTAGTCCTGGTATTGATATATCAAAACTTAATATCAAAACAGAACCACTTACTGAGGAAGTTAGTAAATTAAATTACACACTGTAATGATATTAATGTTTATCAATGAGATCCCTGTTTGTGTGTGTACTGTAATAATTCAAAGCTATTACATTAATGTGCATGTTTCATTTAGGATGATGATCCTACTTTATCTTCTCACCCTTTGTCCTCTCCTCCTGTCGTTACTCAAGATCCTCAAATGTCACTTGAAACTACTGCCATTTTCTCTACTAGTTCGTCATTTCTTACTTCTTCCTCTATTGCTATGGCAAACTCTACTGTTTTTGGAGAATCTTAGAGATCTTATAGGATGACAATCAAGAGTGCTGCTGTTGACTCTTTCCTCTTTATGATGTACTTTTGCTCTCCCATGTTTATTTTTTCAAAATTATTATTTTCTATTTGATTTCAGGTATTATTAAAGTTGTTGTAATGCCTCCTACATTTTTGTAATTTTAATAAAATATAAAAATTAATACACACGCAATGACAAACAATGTATATATGGTATTATACAAATGGGAAAATTAATAAAATAAGAAAAGCTAATAGTAACAATAGAGTGTGTGAGTGACTACATGAAGTCGCAGCACTGGGATATGTTATTATTATTGTTGGCAAGGTGGTATATCTAAGTGAAGACTTATCATTAAATATACATGAAAAAACACTTACTTTGTTGTTGTGACACTATAGCTAGTAGAAGTAGGTGAGGGAGTTGTGGGTGGTGGTGCAGTGGGTAAATTTATAGTAGGAATTGATATATTACTAGGAAGTGTTTTGATAAAGTCTTCATCTGTAACAAATAGTAAAATCGGAGCTTGTAATAATGCTGCCTATTGATTGTATATAATTTATGATATTAATGTCACAATACTGTCATACTTGTTGATAGAAATTGTAAATATTATAATTGTATGCAGAATTGTTAGCCTGTTCCTTGATGTCATATGACGAGAATAGTATCGAGTCACTGTTCACAGTATGCGAACCATTCCAACAATTATTAGCAGAAGTTTGTAGCTGACAAAAAGTTGGAATACTCACTAATATAAAATGCAATCATTGAGAAAATAATAATTACTATACAAACCATAATTAAAGACTCCTCGAACAGATCCCATACTTTCAACTGCAGATTTAAGTGAATTTGGGATAGGGATTGCATCAACTGATCTCTTCCTCCTCATGCCATTAAGGAGTTCTAACAAATTTATGCCACATCTTTGTTGTGACTGACTACTCTACAAATATAATTATATCAATTGTCATTGAATTAGCAATATTACACTCACTAAATTAAAGAGTGTATTTGGAGAAAGGATCTCAACAGTATGACACAAAACAGTGTTGTTCATTAGTGATTGAGTAAGAAACACTAACTGACGGCTGATATTCCACAAAGCTTCAAATTGTGGCAATAGACTCTCGTGCAGTGGTGACTGACAAGCACTATATACCTGTACGCAGGTACTTTCACATAAAGGTGGAATAAGCCGTACACATTGAGCACAATACTGTGTTATAATTGATTCAAGACACTGCTCTGATGGATAGTAAGCTGTGGTATTTAAAACATCAAATCTATTAAGCAACATTTCTATTTTTCTCAACGACTGAAATATTAATTGAAGGATTCTGAAGCCCTCACCAGTTTTCTGAATAGAGTCAGTGTCAACGTGATTATGGATAAGATCTTGAAGGCATTGTTTTTGTGAAGCTTCATATGGAATACTTTGAGTAAAAATTTCAATCAGTGTTGCAGTCATGTTCGTAATATAACTCTCAAAATCAAACATAAGATCTATGGCATAATTGGGATGTAGCTGCTGGAACAATTTTACTTTTGCAGTATTGTTTAGATAGAATGATGCCACTGGTGACAAATGATGTGGAAATGGAAATATGTCAATAAAAATAGACAGAAATTCATCAATTGTAGCTATAAAAAGAATAATTAAAAAAATAATATGAATCACACCAAAATGTAATTCTTTACTTAAGACCCCAATGGGTATACATAGAGCTTAATTCTCGCATACATAAGTTTTTTACTAATTACAATGCTAAAATGTTACTACTATTGTATATTTAGTTTAGTCGTAAGTAAACAATGAAAAATAAATCAATACGCAAAAATAAACAACACAAAGACATAATTAAAGTATCACCTTATTCACCTGCAAATAACAATAGCAGGTTTGCTTCAGTACATTGATAGTAATATTAAGTTTGGGAAGACACGGAGGAGCAAATAGAATTAGGTATTATACACAATAACAGAATTAATACATTAATTAAGCAAGCAATGTTGTACTAATCAACACAGAGTGACAGCTACTCACAAAAACACTCAGTAATTGACTAGTGTTGTTATCATACAAGCCACTCCTGACTAGACAACACTACTAACATTTTCTTATTACAGTGTACTGTAAAGTAATGAAGTAAAGCACCTTTACTGAAAGAATAAGCATCCATCCAAAGACTAGTTGTTGCATGAAGATCTTTTTGCATTCCAGTAATTAGATCCTCAGTGATATTAGCCTTCATTCTCAATAAGTGACTTTCAGTGCAACATTGGACCGTGTCTTCAAGTGAACAATATGGTAAGTCTGATCCTAAAGTCAAACAAAAGAGGAAACTGACAATACGGCCTATAGTATACCAACCTGGAATTGGTAAAATAGGATCCGAGAAGTCTAACTTGTGACAGTCAATCACACTTTCAACGGAAATGGCCAAGAACAACAATAAAAGTGTAATAGGTAAAATCCTGGCCATCTTCACCTCTTCTCTGCCGCCACACACGCTCAGGCTCAGCTCACTCATCCTTTTGTTGCGTGGGCGTACCGGATATCAACTGCATGGACATGAACCCATAAAACTGATTGATAGCCTAAAAATGAAATAAGGCATTGACCTAGAACAGAATGGCAGATTCAAACTGTTCCATCAACATAAACATCATAAATACTCCTGGTATCACAGTGCCAAATGTCATCATTTAAACTACTATCAATCCATGGATACTTATTGTATCTTTACTTGTCCAAAAGATGTGTTTTTAGTTTTTGATACTTTACTGTGCCCAAAAATGACACATGTACGTATAAAAGAAAATGTGCATAATGTCTACGGAACACATGACTGATAAGGTCAAAGCTGATATTTTAGCAACCAAGTTACAACAACCTTTAATTCATTATACTGAAAACCATATCTTTTAACACACAAGCATACTCACGGAATAAACCTAGATCTACCAGTACCAGAAAACAACCACGATAGTCGATAACCCACTTATACTTAAATTTCCAGTTTCTCTGTGAAGTTTGTTAATTAAATATAAGTAAGACATATGTAATAGCAAGGAACATCAACAGAACAGAGGAGCTCATAATTCCAGCTCCACTGGGAGTTGGAGTATCTTCAGGATTACCCAAGCTTTGTGGAGCAGTACTTCCAAAATCATCACCAGAAATATAATCAGTCACAGGATTACCTAAAAAAAAGTTGCTCATAGCATAAATAAAATCCCAGTGCATACCAGGGGCAGACACTTGACTTCGAACTTCATCAGTGGTTACACCCTTCAGGTAGTGGATTACTAACTTCCAGAACATCATCAATATCTTCTTCAAGTTTAAAAATTTTTTACCTGGATCAGATATCTCTGTTATATTAATAGCAGGAGGTGTATATGTTGTTACATTAGCAGTTCCATTCCAGCAGTTAGCATTGCTGTAATTACGGCAATAAAGAGGTCCATTATGTCCTACAAGTTAGTAATAATAGTGATGCTAGAAAGATAAAGAACAGCAATTACCATAGGAAACAAATCCTCTCCCAAGAAGAGAATTTGCAGCTTGAATAAACATTCCCGGAACTTCAAGTCTATTGCTTTCATCATCATCATCGTCATCATCATCGTCATCATCGCTGCGGCGTCTACGATTTGAAGCTGATGGGTTTGTAATAGTAATTCCACAATCAACAGCATGCTAAGTATAAAAAGCAGTATTTTCGTAATATTAAACCATTGAGCTTACCCGAAATATTTCAGCATTATCTAAAGAATTACTACGATAACGAGGAGCAATTTTGGTATATCTGCCATTGATGACCTTGATATATCCTCAGGTGGG

>genscan_predicted_peptide

XDFNHSPVHQAQSTAPVSGLHSMSSTEHYLQHPTPGVPVKSESPIMMSTDQPLSQNDQAL

IDDIMGIEDKLEGFTPLPFFEQQMMMPQTLPVSNNLLDMFGGGAASSHQMFVPQISSSCP

PEVKEEKMREACSFGINPIERRRRFNINDRIKELGMLLPSSESEARQNKGSILKASVDYI

RKLQRDVERLKIQDAKQRQLEETNRQMRLRIQELELTARAHGIRTPSLTPETKQLADAAD

KVLGPTTPRARSPVSSTTTTPLSPHVFSGVDDYMLRASPGIDISKLNIKTEPLTEEVSKL

NYTL

>geneid_v1.2_predicted_protein_1|249_AA

DQALIDDIMGIEDKLEGFTPLPFFEQQMMMPQTLPVSNNLLDMFGGGAASSHQMFVPQIS

SSCPPEVKEEKMREACSFGINPIERRRRFNINDRIKELGMLLPSSESEARQNKGSILKAS

VDYIRKLQRDVERLKIQDAKQRQLEETNRQMRLRIQELELTARAHGIRTPSLTPETKQLA

DAADKVLGPTTPRARSPVSSTTTTPLSPHVFSGVDDYMLRASPGIDISKLNIKTEPLTEE

VSKLNYTL*

**>Amq13** GGCCCGAGGAGAGTATTTTCCCCAGGAGGAGTTAGAGGATTGAGTGATGGCCCAAAAGATAGATTCAAACAAAGATCAGTCAGTTGAGAGAGTTTTGTAAGATTGTGTCGAGTACACCCCCGAGGCCCTTCACCTCCCCGAATAGGGTTCTTTTAAACAGTTCATTGAAGGGTCAGTCTGGTTGTTGTTGTTGGTTGTTGTTTATTGTTTGTGTTTAGTTTGGGTGGTACTATTCCTCCATTACGTACTAAGGAGCCAGAACAAGATGAGCAGACTCCAATGGAAACTGATCCACTCCCTAAGCCTAAACCAGCCCCACAGCCTCCTCCTGATAAGGTACTCCCCACTCACTCACTGGTAGAGTCTAACCTTTATTGAACAGGAACCATCTCCGGAGCCTGAGCCTCAGAGTGAAGAAAGTGATTTAGGTATGAACAGCAACAATAACTGATAATTTATTATTATTATCAGATATTGATGACAGTGATACTATTGAACCAGAAACTGATCCTCCACAAGAAATGGGTGATCCAAACAAAGAAGTAACTGCATGTGTATACCACTGCATGTATTGGATCAATTGTACATGAGTGTGTGTGTGTTTATTGTCATTATATTGTTAGGTAACTGAAGAGATGATGGACACATCGCAAGAAGAAAGGGGATTAGCAATGACTGCAATATCTGATGGTACTTTAATTTTAATTTTATTTATTTGTTTGTTTGTTTCTAGGAGATCTAGAGAAAGCAGTAGTCCATTTTACAAATGCAATATTAAACAATCCAAACTCGGCACTTCTTTATGCCAAAAGAGCAAGGTTCAGTGTACATGATACTAAATTGGTCTATTCATTTTGTTTAGTATCTATGTTAAACTCAAGAAACCGAATGCTGCCATTAGAGACTGTACTGAAGCGATTAGGATGAACCCTGACTCCGCCCAGGGCTACAAATGGAGGGGAAAGGCTCACACGTAAGCTAGACCTGGAGCTGGTAATGGATCCAATGGTGAGTGTCTCGTTGATATATTGCAGGCTATTGGGTCACTGGGAAGATGCAGCCAGGGACCTTCAAACTGCCTGTAAACTGGACTATGATGATGAAGCCAATGAAATGCTTAAAGCTGTCAAACCTAAAGTTAGTATACTGACCAGTATATTATTGGATATTATTGGATATTATTAGGCTGAAAGGATCAGAGAACACAAGTTGAAGTATGAAAGAAAAAAAAGAGAAAAAGACATTGAAGAGAGAAAGAGACGCATGTAAGCAACTGTGTTATGTTTATTTATTTGTTTGTTTGTTTTTTTGTTTGTTTTATAGTCAAGAAGCTCGTGACCAAGAAAGAAAACAAAAGGTAGTGAATTGTTTTTCATTAGTAATTCTATTAATTTAATTTTAGGAAGAACAAGAGAAACAAGAAAGAGAGAGATTTGCTAGAATGGGAGGAATGCCAGGAATGGGTGGAATGCCAGGAATGGGTGGAATGCCAGGAATGGGTGGAATGCCTTTCGGTGGAATGGGAGGAATGGGTGGGATGCCAGGAATGGGAGGTGGTATGTTAACAATATTATAATTATGACAATAGTTTATTATTATTACTAGGTGGTGGTGGTCCTGACTTGGGTAGTCTGTTTCAAGATCCTGAACTATTAGAAATGATGCAAGTGAGACCCTCATACCAACAGTAGCAGCTGCTCATTGAACAACTTGTCTTTATAACAGGATCCTGAAGTCCAGACTGCATTCATGGACATACAGAGGAACCCAGCCAACATCACCAAGTATCAATCCAATCCAAAAATACAAAAAGTAATGGAGAAGATATCAAATAAAGTTGGAGGAACGGGAGGTGCCCCTCCAGGGGCAGGGGGAGGTTCCCCCTTTGGAAGCACTCCTCCCGGAGGAATGGGAGGCTCCTCTCCTGCTGCCGGGGGTGACAGTATGGGACTAGATTAAACTGATAGTTGCCATGGCAATGACAGCGTGTAACATTATTGTGTTGATTAAAACAAATTTAATTATCCTTTATTAATTAATGTTAATTAATGATTATTTTTGTTCTTTGTCTTGGTAAAGTGAATGTAACACACTAAAAATGAATTAATAGCAATCAATGATAACAATCAGTGATTATAATCACTGATAATGGGACAGTATCTAACTGCTCTGATGTGTGTGTTCCTTAATATGGTAATTACCATTTGGTCTAGTATGTCCATCAGTTTTTCTCTTGATATGTTAATTTGTTTATTTTTTAATTTACTGTGAATAAATGACAGCTCCAATGGCTATAAATATACTAGAATATACTAGAATATCTGGTAATTCAGATACCTTATATTTCAGTATATCTTGATACATCTCAGTGTCACTAACAATAGCATCTCTAACACACTGTTGATCATTCATCAATCATTCAATCATTATTATTATTAATATATTACTTTACGATCAATTTCACTTTTATTTCCTGTACTTTTGGACTCATCAACAATATATCCTTCTTCATCTGCAACAACAACATACCATATAAGGTATTATATATACCACCTTATATACCATATAAGGTACAATATTATGCTTACCGACCAGTGGGTGGTTATAGGTGTATATTTGCTCCAGAAGTTGAATCATTTTCTTCTTTGGCAGAGCTCGTACACCAAACTTGGAGCAAGCACTCTACAGTAGGGACACAACATAACACTGGTAGAGCAGCTGCATGTTGAATTTTCTGTAAAATACTAAAATTGTTGTTATTTATTGTTAAACTGCAGCAAATTTACCATTGAGCATAATTTGCATAATGTTAAATTATTTTTCTACATTTTAAAAGTTTTTCCAAAATTGTGCCAAAGTCCCTGTACTATAGTACAGGGACTTTGGCCAAATTTCATCAAAAATGAAAGGTAGAAAAATTTCAATTTTTATTACATTTCCTGATACATGTACTTATGTTGTGTAGGAAGTTTGAACTGATTTAATCTGATTCCAATCAAAATTGGATTTTTTAACAAATTTTTAAAGACCTGATGTACTATTAATTATATACTAATTAATTACTTTCAAATGAGGTGTGGCCATTGCTCTATAGTCCGGCATTGGAGTTATGTTATCGTCAGATTGAAGGACACTCCTCCTTTCACCAGCTGGGGTTATCAGCATTGCCCCATAACCACACCCACTAGGAGTGTCAATAACTGATTGGGCTTTAATTGGAATATCATGTGACATACACGTGCACTCTTCATCAGAGGACGTGGGTACTGAAGAGGAACATCCCATTTCCCCTTCTAGTGGGGAGGGCATTGCTGGAATGTGTTCAGGCGATATAGTGGGAGTGGTCAATGATGAAGAAGTGGGTGTGGTTATTAATATCTTCAATAAATCAGGTACCTCCCACCTATTAATAATAAGACTAATGACAAGGTCACTCTCACTCACTCACAGTTCTGATAGAGTGATCAGTTCCTGTTTGATTTCAGCTGATACTTCACCTTCAGGTACAGCAGCTGCATACATGAACCTCATGAAGATATTAACAGATTCAAAACTGAAAGAAGAACAATCAATGGACAGATTATCAGGAGATAGACTAGATAATACCTAACATAATAATAATAATAATAATAATGATCAATGATGATAATAATAATAATAATAATGATCAATGATGATAATAATAATAATAACTCACTCTGGCCATTGCAGGTGATCGTACAGACAGTATTGATGAGTGTCCATTGATTCTCCTACCATTGGATACCTTTATAACAACATCAGAATACACTGAACTTGGTTAAAAGATTATTTAAACCGGTTTTAAATCTTTTGGTTGGACTAAGATCATCATCAATCCTCAATAAAAGGTACATCAACCTCGGTTTTCATTCCAATGGAATTGGGTACCGGTGAGTTTGGTATTGGTGAGTTTGGTACCAGTGAATTTGGTACCGGTGAGATTGGTACAGGTGAGTTTGGTACTGGTGAGTTTGGTACCAGTGAGTTTGGTACTGGTGAGTTTAGTGCCAATGTAGCAAGGCATGGATCAGAATGACATATGCAGCATTTGGACTCATTAACCTTTAAATTTAAAATTATTTAAAACTCAATAATTTATTCTGACTAACCTCACTTCCATCAATAAAGTCCTTTAGTCCTGGCACATTAAACACTGAAGGAGGTTCCTCAGGACCAGATGCTGAAAGAACCCAAAGTGTTTTATAACCTGAGAACTCACTGGACCCGTGGGACTGACCATCAGTGGGACACTGGAGGGACAGCTGGTCCGCTAGAGGTACATCATCCTTATCCTTTGATCCAACAGCAGCTGCTGGTGACTGACAAGTAAACAGCTCCTCCAGTCGTGTAGCTATTACTTCATTTTGAATACTACTCGTTAATAAGGTGGGCGGAGATCTCAGTTCACCTTTCTTATGACGTGATTTTGATTTAATATTGTAGGATTGGCCAATAGGTGGAGCTACATATAAAGAAAAAACAAAACAAAACAATTGTTACAGTTAAACAGTTCAAACTTTCTACACAACATAAGTACATGTATATGCATCAGAAAATGTAATGAAACCGAGAAATTTCATACTTGTCAATTTTGGTGAAATTTGGCCAAAATCCCTGTACTATAGTACAGGGACTTTTGTCCGATTTTGGAGCAACTTTAAAAAATCATAAAAAATCCAAACTTGATCGGAATCAGTTCAAACTTTCTATGCAACATAAGTACATGTATATGCATCAGAAAATGTAATAAAATTGACGAATTTTCTACTTGTCAATTTTTATGAAATTTGACAAAAATCCCTATACTATAGTACAGGGACTTTTGTCCGATTTTGGAGCAACTTTAAAAAATCATAAAAAATTTAAACTTGATTGGAATCCCCTCAAACTTTCTACACAACATAAGTACATGTATATGCATCAGAAAATGTAATAAAATTGAGGAATTTTCTACTTGTCAATTTTGGTCAAATTTCACAAAAATCCCTGTACTATAGTACAGGGACTTTTGTCCGATTTTGGAGCAACTTTAAAAATCATAAAAAATCCAAACTTGATCGGAATCAGTTCAAACTTTCTATGCAACATAAGTACATGTGTGTGTGTATGTTGACATAAACTAAGTACCACTCACATATCTCAGAGGAAGACAAAGCCATTGCTAATTGAAGCTGTTCACTCTCAATCACTTTAAGAGCCTCATCACTATAATAATAATAATTATTATTATTATTAATATTATACTAAATTTCTTACTTTTCTTTTCCTTTGGTCTTCTTCAGTTTCTTTATGTTAATTGTTGGGATTTTAAAATCATCGCCATCATCATCAACAATGTGTTCCATCTTTATCTTCTTAATGTTCACTGTATCTCCTTCACTGTCCCACTGTCCTTTAGACCAAACTTCATAATCACTATCAATCTTTGGTTGTGCTACTGTCTCCTGGGGCTCCTGCTCCTTCTTAAGCTTCGAAGGATTTCTTCTTTTAAGTGAGAGTTTGTGCTTTTTGGGCCCATGACGACTGTCCATGATTTAATATTTATATATAATTAATTAGAACATGGAAAGTACAGGTGTTAATTAATGACAGTTGCTATTGTTTGTCTTTTTCTCTCTTAATCTCAGCTGCTACTCTTGTTTTAAACTCTGAAGCTGCTAGTCTGCTTGCTTCAGCTTGTCTCTCTCTCTCCTCCAGGACTTCAGGAGTCAAAATGACCCTCTTCCTCAAGCTGTAGGCCCCAATGAGAATAACTAGGACTGAACCAAAGGACACTGGATAGAGGAAACGCCTCCAGTTAGACATTAATACCAAACAAGAAGGCTTCTAATTTAAATTCTAATAATTGTATTCCTGCAAAATAAAAATGGCTCCCAATGGCAAATGGGCGGATTTCTTATGGGTGGGTCTTGTTGTCATGGCAACAGTATCATAGCAACCCTAATGAGATGCATGCAGTGGTTGTGGTCCGAGGCGGTCCTGTGTTTTGATTGGTCAGTTGTGTATGGAGCGATGGCCTTATGTCGACTGCTCGTCCAGTGAATAGATAAGAAGAGAGAGACCATTGATAGGGAGACATGACTGACCATCACGCCACATTAGGAGTCAAGAGAGAGAATACCTTCACTAGCAATAGCTGCAGCCTCCAATCCTTTGGCTGTCCCTCTCCTACTGATAAAAGGTCACCTTTCCTTCTCTTAGACCCTCCAGTTAGTACCCCAGCCACTGGGAGGGAAGGATTTGATATCGACATGGACCTGGCAAAGTTCCTTGGGGACCCCGACATGACAACACCAAGTGAGAACCTTTTGAGAGGATATCATGTCTACAATGATAATAATAATGATAACATGATACGATACATGTACTGGTTTGATTCAAGTGTTTTAAGTTACTCAAAAATTAGATAAAAGACCTTGTACTACAGTACATGGTCTTTTATCAAAATGACTAGGAAAGGATTCTATGATTTAAAGTGTTCTTTATCCCTCCAGCTACCACATGCAGTTCCAATGGGATGGACGGATCGGAATGGAACACCCTCTTCACTGAGACTGACCTGGAGGGACTGGATGTGTTGGATCCGAGCACTGGTGCCCCGGCCGGAGGAGGAGGGTCTAATGGACTAACGGATCATCCTAGCTTTGTACCTGGACTGACTCCTGTCACCAGCTCTGGGTTTAATGGCGGCCATGTTGAACTACAACAAAACAATAATAATGTGACTGACTCCAACTGGCTTATTGAACAAAGTCAAAAACTTGAAAGACAACAGCAACTTCAACAACAGAAACTACTGGAACTGGAACAGGTAACATTTTATTTATTCATTCATTTATTTATTTATTATAATTATTTCAGACATTATTAAGGACAGAGATACTAAAGAATAGGCTTGAGATTGTTAGTAACTTGAAGCATGCCTCCCCAGAGCTCCAGTCCCAGCTCATTGGTGTACTCTCCACCCGTTCCTCCTCCATTGGGGGCGTGTCTACCACACACACTCAGCAGTCCTTGACCACGCCCACCAGTGTAGCACCAAGACTAGTGTCTCGTCCACAGACCAGTGTACCAGCGCCAGTAGTCCCTCGTTCTACTTCCCAAGTCGTTGCTCCAACTTCATTGCCATCACCTCCTGACTCTGAGCTTGATGACTTCACTGAGTATGGTCCTGGGGGTGTGGTCAAGAAGAAGAGCCACAACCTCATTGAGAAGAAGTATCGTACCAGCATCAACGACAGGATAGGGACATTGAGGGATATTGTCTCCAAACACTTCAGAGATGATAAGAAAGTAATTCATTATTATTATTATTATTATTATTATTATTATTATTGTTATTATCTTGTGTGTGTAGGTTCAAAAGTCGGCAGTTCTACAAAAGACAATTGATTACATTCGTTATCTTGAGAACTCCAACAGGAAACTGCTTGAAGAGAACAGAGCATTAAAAACACAGTTAATGAAACAAGGTGAGAATTAATAATAATAATATAAATCTTTATAATAATTAACAGGAAGCCAGTCAGTACTAACGAATGGTCTAAGCTCCAATCATTCTCCTTTGTCATGGAGTGATCCTTCTGATGATGGACACCCTTCATCACCTGATGTCTTCCTTAGTGTGAGTATTTTATTTATCTATTAATTAATTTGTTTATTTATTTATTTATTTTATTATGTAGGACACTAGTCCAACAGCCAGCCCGCCCCTCCCCTTGTCCCCTCGTTATAGTTCAGTCCTCAAGTTGGGTGGACGTATCTTCATGTGTTTCATGTTGGTCATGTGCTTTATGGTAGACCCTCTCTCTCTCATCGGAGGAGGAGGGGGTGTGGTCAGTAATGGAGGCGTGTCCCACGGCTCCCAGAGGACCCTTAGCTCCATTGACAGTGAGGGAGAGGAGTTTAACTTTAGCTGGTGTCTGGTTTGGGGCCTCAGACTGTTCTTTGCTGGTCTGTGCTTTGGGTGGGTGTGGCTTCACTCTCTGCCAGCTGTTAATCCAGAGTGGGAGGGAGTGAGGTACTGGAGACTGAGGAAACAAGCAGAGAAAGATATTAAAAATGTAAAGCATTTAATGTTACTAAAATAGATACTGTTAATGGGTTGATGTTACTAAATATAGAACTGATGGTTATTGGTTATTAATGTAGGGTGATTTCTCGTCTGCTCAAGTTCATCTAGTGTCAGCTCTCACTGAAGTTGGCCTGTCCCTCCCCACCAGCTACACTGGGCTCACCCTATCACTATTATGGAATGGACTCTCTCACGTGCTCTTTCAAGTGTGTTACTATTTTTAGAACTAGTTCTTCCTCTTTTCAGTGTTACTATATTAAGTAACATGTTCTCATTCCTTTAGCTTTATGTTGGTCAGCTTTTGGTTGGTTTTGTATCTTTTGTGTTCTCTAAATTTAATAAAGTATCTTCAAGTCAACAAAAATGTCTACACATTGTTAAGAATGCTGACGTGGCAAAAACCAAACACCTCCTTCATCAAATAATACTCTCAGGTATTGTATTGTATTGTATTGTATTGTACTGTACTGTACTGTACTGTATTGTATTGTACTGTACTGTATTTATTTATTTATTTATTTATTGTTTGCAGGTTATGATGAGCGCTCCATGCACGGGTTAGGGTTCTATCTGGGGCTCACGGCTCTTAACTATGGAGAGGTGGAGAGAGAGAGTTTCTCTCGTGAGCAGTGGGCGGAGCTACTGTTTGTAATGGGAGTGCAGTGTCAATACTCGTGTGCCTGTATAGCCTCACTATTAGCAGTAAGGAGAACCATTATGATATGATTTATTAGTACATAATACATTATTATTATTATTATTATTATTATTATTATTATTATTATTATTGTTATTATTATTATTGTTATTATTATTGTAGCATTATTATTTCTTATGTGCTGGTAACATGAGTGGTCTGTTGTCATTCATCAATACGACTGATGGTTGGAATTACCTCATGAATAAAGACTGGCTGGCTGACGTACAGTCCGCCAATAAACTCTACAAAGGAGTGGCTGGTATGTGGGTGTGGTCATTGTACATTAATTATTATTAATTATTAGTGTTTCCTAAATATTCAATCACGCGAAGTTACTGCAAGTATCTGTTAACTGATGCTTATCAGGAGCTGCTAACACCCAGCGAGGACCCAGCCTCCCTTGTAGAAACAGAGAAGGTTTTTAAAACAGTTCTGGCTGTGACGAGACGTGCCAATGGACATCTACTGGAGGATATGAAGGTACTAATGAGTGGGCGTGGTCACTGATTTTTATTACTGTACATCAATTAGTTATTAATTGAAGATATACCAACGTTGAGTGATACTGCCTTGTCTCGTGGGTGTGGTTTTGAGTCTTTAATGTGGTGGGCGTCGGTCGGACTAGTATCCTGTTCCTGGAGGAGTGGGTCTAAAGGAGATGATCATTATCCCATTGTTGAAGATATTTCTAACGGGAGGTACTCCATTAATTATCATTATTATTATTATTATTCAACCATATCTCTCCTTCACAGTGGCCCTATTCCATCAGTATTACATTTATCATTCAAGGCTCAGAGGTTATACAATCACTCATCAGTGGCTCCTCCTTTAGCTGTCATTAATCAGTTGTGTTCCACTGCTAGTCAAGCACTCAAGATTTGTAGACAAGATCTCTCATCAGAAGATACCTTGAACCTTGTAATGATAACTGTTACACACACAGTCTTATGCTATGCTTATTCTGTTCTTGTGTAGTTTGGTCTCTTCCTTGCCTGCGACTGGCTCCTCGAGCTAAGGTACAAGGTGTGGAAGGATAATGCTAATAAAGAGGAGACTGACCCACTCCTAATGAGCTCCATAGTTAAAGGATACTCTCAAGACATTGAAGTCCTCAATGAACTAACTGTACACCTTCCACTGGCCTTTACTCAAGTAAGAACAGACACAGTGTTCAATAAATGATAATATTGTCCCTAGTTGAGGAGGTATAAAGGATATCAAAGACTAATTACTGGGGGTAACCCCCTCCTGGCTGATACCTTCCTCACCCCAGGGACTGTTCACAAGAGACTAGTGTCTACGTAACAGCTGGACTCATTATTATTATTATTATTATTATTAATTATACGAAGCTTTTCATGAACTATAAATACTCTGTTTATTATTGTTGTTGTTCTGTAGTATCAGTTTACACATTTAATAATAAAAATAATTAAGATTATATTAATTGATAATGGTGTTATTAATTGTGTGTAAACTAGTTGTTTTGTCACTAAACAATCTAGACAAAGCGTCCTGGGTTCCAATTACTGCCCTAATTAACTGATATCAATTAATTAATAATGACATTAGAACTATTACCCTATCAATCCAATTAATATCAGTAATATATCAAAGAAAAATTGAAATCGAGCAATGTTCCTATAAATAGAAACACATACAGAGGTGTTACTAAGCAACACGTACTTTCCAAATAGTTTAACATGAAAGACAGCAGGAAGAATGAAGGCAAGGAGAGTACAACAAGATGAGCCAACCAGAGCCATCAAAATGGAGAAATTAGGGATAAGCAGCACAATGATCCCAGTTACTAGAACCAATAGAATCCTCAGGATACACTAACAGGGAGGAACAGATCAGGAAATGTAATAAAATTGAGGAATTTTCTATATGTCAATTTTGGTCAAATTTCACAAAAATCCCTGTACTATAGTACAGGGACTTTTGTCCAATTTTGGAGCAAATTAAAAAAATCATAAAAAATCCAAACTTGATCGGAATCAGCTCAAACTTTCTACGCAACATAAGTACATGTATATGCATCAGAAAATGTAATAAAATTGACGAATTTTCTACTTGTCAATTTTGGTCAAATTTCACAAAAATCCCTGTACTATAGTACAGGGACTTTTGTCCAATTTTGGAGCAAATTAAAAAAATCATAAAAAATCCAAACTTGATCGAAATCCGCTCAAACTTTCTACACAACATAAGTACATGTATATGCATCAGAAAATGTAATAAAATTGACGAATTTTCTACTTGTCAATTTTGGTCAAATTTCACAAAAATCCCTGTACTATAGTACAGGGACTTTTGTCCAATTTTGGAGCAAATTAAAAAAATCATAAAAAATCCAAACTTGATCGAAATCCGCTCAAACTTTCTACACAGCATAAGTACATGTATATGCATCAGAAAATGTAATAAAGAGGCTGTCATTATTTAGTGGGATTACCCCAGAGAGGTAGCCAGTGAAGTGTGAGGGAGAGAATGAGACACAACTGATCCTTTTCTCTAGTATCTCAGATACAGGAAACATCATCACTATGGCAACCAGTAAATGTGATCATTAATTAGTTAAGTTACTTGGATAAGTGAAGAATAGAGAGAGACATAGACAAGACTTCACAATCAGAGGAAATGGACCTAATTAATTAATTAATTAATTAAATTATAGAGTATGAATATTAATTAGGCATGTACCAGGTGGAAGGTTTAGTGTTATAATACTCTGAGTATAAGGACCAAATGACTATTGAATAGTAATTATTAATAATAATGAATAATGCAGTATTCTAAAAATAGAACACTTACCAGGTACCCCATTACACCAAATACTATGTACAATAGTGTTACTAAAAATAGAACGAGCTTGAATATGCTACAATAAATTATTAATATATTCAGATTATAAAGTATTTTAAGAACCTTCTGAAAGTATTTCTATAATCTTTAATAACTGACTGCTCCAATGACAGGACCATACCCGCCCCCTACAGTAACGATTGACCTCATTAATGTTCATTGACTAATTAATATTCATTACCTCATAGCAATAAATAGCCACGCCCAAAAAAAATGGCAGACCATCAAGTGAAATTGACCTTACATGTGAACTAAAAATGGAATATTAATAATTAATAAATAATTAATTAATTATTAATCAATTACTCACTGGACAAGATGCAAATGCCTCAAGTCAAAGTAGAAGACTATGCTGTAAGCAAAGACGTTAGCAAAATCAGCAAACAAGCTAATGAAACATCAAATAATAATAAGACACTAAACCATTAATGACTGAACTAGCTCATTTCATTCACTCATTAGCACAAATGCTAACAACTGACAGCCTCTCCATTTCATAATGGAGTAGAAACAGTACGAGTTACCAAAGCAACAGTACGAGTTACTAAGGCAACAGTACGTGTTACCAAGGCAACAGTACGTGTTACCAAGGCAACAGTACGTGTTACCAAGGCAACAGTATTGTACCTGAAAGGTGCTAGAGCTGCCAAGTGTCTGAGATTACACAAGAAACACAACGGAACTAATAATAAAAATAAATAAATTAACCTAACAAATGAATAAATTAATATTGACTATATGAGTGATGAATACTTTGGTATACGAGGGAAGATGCTGTAGAAATTCTCTGAAATAAATATGAGGTAAGCACAATTGAATCCTGAGGACACAAAGAGTTACCAATGCAATCAATTAATTAATTAATTAATTATTGTATGTACCAATTTGTGATACAATAATTGAAAAGTCAACGACAACTTTACCAGCTGAACCGTAAGCAGCAAAACCTAATAATAATAATAATAATAATAATAATAATAATAATAATAATAATGATGATGATGATACCTAGTTCTCCATAGTCAATGTGAGTTATGCCAACTTTAGCTGGTGGGGGGAGTTCATCCTGTTCCTGTCGGCCATTTTTAATTATTAACACTCGCGATTCTTTTAGTATCTTATCTTTACAGTCGATCAAGAGGAGCATTGCCTTCACACTGCAACAGAACAATCACATGATCATCACATGACCTGCACTGTCGTTACCTTATCACACCAATCAATGACATCACTATAACACCTTCGATTAGACCAGCCTAACATCAATAGATAAACAATAAATAAAATATAAAATAAATAAAATTACTTCCATAAAAGCATAGGGTAGTCCCAGTATACCCGCTCCAATGAATGATATGAATATGTTTCCAAATATCTTAATACCACTGGATGTTCCTTCGGTATCTCCATGCATCATGGACCAACAGAACACAAACTGAAAGAAAGACAAGTTTACAAAATGGATGGGGCTTGAGTACTGGGTGGGGCTTGAGTAGTGAGTGGGTGGGCTTGTACTTCTTGCAACCCACTCAAGTAATTAGTAGTAGGTGGAGCTTATAAACTCTCACACAACTGGAGAAGAAAAGAAGAAGTCATGTCTCTTCCTCGTAGCTCCAGTAAAACTGGAGTTGAGTATGACTTTTTATATCGTCGTCCTTCAGATATACGAACCTGCCAGTTTAGAGTGAGATCCTACAGTATTATTCTACAATGATACACATTCTCTTCTCTTTATTGTCTGCCACAGGAGTCCATTCTTGATATTGCTGCTTGCAAGAATGAGCAGCAGCTTAGACTAGCAGTTTGTAAAGGAGTGGAGGCTTGCTTTAAGAACCTGGTAAGCTCCCTATCCTTCTAATCATTATTATTATTCATTTTTTGCAGTATAAAATGGAAGCATTGATGTTCTTCAGTGATGAGATGCAGTACAAGACCAATGGAGATGAAGGCCCCTTAAAAAATCTTGCTACAACAGGAATACTGTGGTGATGATAACAAAAATTAGACTATTAATGATTATGTAATCTTAGATTGTGCATGTAGTCCAATATAAATGTTGCAATAATGAGACTAACAGCTTACCATGCCAGCAGCTAATGCTGTGTAGTATTGGATGCATCCATCACGTACACTTGCAGAGTTCTCATTATAATATCAGCTTAACTGAGCCTGTCACTATCAAAACACACTCTGAACACGTGAACGCACGTGACAGGAACATTGCAATGCAGTGTGTAGGTGGGTAAGAGTATAAATAGACCTGGTAGCAGGAGGTTATCCTTCACTAGCAGCTGCAACAGGAGGTTATCCTTCACTAGCAGCTGGTCAGTTTAGAGCCTGTGACTTGTATTGTGTTGTGGGTAGTACATTGCCAGGAATTCACAATATTATAGGTCCAGATATACTAAGATACACTGTATATTGACCTGGTGTTGTATTTTATAATAAATATATTGTGTTCTTATTTTATAGGACGACAGTCAGGGAGAGATCTATTAAAGCATATGATACAATATCTCCTGAAGATTCATTTTATTCTGTCATGGACGTGACTCCAGAGAGCATTGAGGATTCTATTGGTGAATTCAATGTTCCTTCATACCATGCACTCACATGCAGTGTTCATGTACCTGTGCAGCCAAAAAGAGGTTGCTGCAGTCATTACTGTTGCTTATTGCACATCTACTGGCAATGGGGGAAATTTTAAATAGGAAATACAAGAAAAATTATGATTTGCCCTACTAAAATTTGTGTCTATCTGGTATGTTTATTTTTTTTCCTGTTTTGGAATGCGGTGTTAAATTTGTGTTAAATGGTGCAATTGCTTCTTGCTAACGAGTGAATGATGTAAGTTTTCAGAATATCCTGTTGTGCTAGCTGTTTTTCATACCAGTTTACAGTTGATACATTTTACCTTTTCTGTCTTTCCGTTTATTAATTATTACCTTATGCTCACTGTCATTAATTAACAACACGTCATTCCAATAATCACTGTTTGCTCTCCCTGTAGTAATAGTTGATGCCATCAGTAATGTTGGTCAGATGAGAACTGTTGCCCTCATAATAGTAAGATTAATGGATTATGAATTACTGTATCTATTTGATATTCAGGCAATCTGCAGACAACCCATATCTGATGAAGAGACTGATAATTACCATTGGCTTTCCAAGAATGTCAGCATATTAATATTGGGATTATATTAATATTGGGATTATATTAATATTGGGATTATATTAATATTAGGGTTATATTAATATTAGGATTATATTAATATTAGGATTATATTAATATTGGGATTATATTAATATTGGGATTATATTAATATTTGGATGATTGTTATTTGTCATTGAATAGGTGCAAGTTGCAGCTGGTCGTATTCTGGAGTCCATCAAAGCTGAGAGGGAAAAGAAAAGAATGGAAGAAATGCTGAAACTCTCAGGTACATGTGATTGATTATTTATTTATTTATTTGTTTATCTATTTATTTATTTGTTTATTTATTTGTTTGTTTGTTTATTTATCTTTAGGGAGCCTTATTGATGTTGATGTCCCTATGTTGCTTCTTAAACTTCACTCTCATGTATGTACATGTACACTTGTCATTATATGCTATAGCCCCACCCATTAATAATAGATCAAGGAGATTATTGGTTGCAAACGCTGTGTTGTGTTTATTCTGGATGAAGAGACTGATGAGCTTGTTTGTGAAGTAATTAATAATAATGATATTTATTAATATTTATTAATTAATAGGTATTTGATGATGTTGCACTTGACTCTGAGATCAGGATACCAGTTAGTTATTAATAGTTATCAATAGTTATTAGTTATCAATAGTTATCAGTATTTAGTAGTTATTAATAGTTATTGATAAGTTATTAATTATAGATTCCAGATTGCATTTATGGTCAGTCCATCATGTCTGATAAGATCATTCAAATTGATGATGTTTCCAAGGTAACACAGTATTATTATTATTATTATTATTATTATTATTATTATTATTATTATTATTATTATTATTATTATTATTATTATTATTATTATTATTATTATTATTAACTATAGGACCCTCATATCAAACCCAGTAAAGACTTTGTTAATGGTAACGTATACTATTATTATCTGATATTAGCTCCAGCCACAGGATATGAGCCTTCCAATTCGTAGTGCATACCCCTCAAGAGCAAGAGGACCAGTGCTGGTACTATAGCAGTAGTTGGAGCAGCTGCTGACTATGATAAACCAGACGGGTACTGCGCTTCACTTATAACTGTGCTTGATGTAGCTCCTGGAACTGTAGCTCAGACATTTTATGTGAACAGCTTATCAGTATC

>genscan_predicted_peptide

MDLAKFLGDPDMTTPTTTCSSNGMDGSEWNTLFTETDLEGLDVLDPSTGAPAGGGGSNGL

TDHPSFVPGLTPVTSSGFNGGHVELQQNNNNVTDSNWLIEQSQKLERQQQLQQQKLLELE

QTSVPAPVVPRSTSQVVAPTSLPSPPDSELDDFTEYGPGGVVKKKSHNLIEKKYRTSIND

RIGTLRDIVSKHFRDDKKVQKSAVLQKTIDYIRYLENSNRKLLEENRALKTQLMKQGSQS

VLTNGLSSNHSPLSWSDPSDDGHPSSPDVFLSDTSPTASPPLPLSPRYSSVLKLGGRIFM

CFMLVMCFMVDPLSLIGGGGGVVSNGGVSHGSQRTLSSIDSYDERSMHGLGFYLGLTALN

YGEHYYFLCAGNMSGLLSFINTTDGWNYLMNKDWLADVQSANKLYKGVAGMWFGLFLACD

WLLELRYKVWKDNANKEETDPLLMSSIVKGYSQDIEVLNELTVHLPLAFTQESILDIAAC

KNEQQLRLAVCKGVEACFKNLYKMEALMFFSDEMQYKTNGDEGPLKNLATTGILW

>Amq_2|geneid_v1.2_predicted_protein_2|394_AA

MDLAKFLGDPDMTTPTTTCSSNGMDGSEWNTLFTETDLEGLDVLDPSTGAPAGGGGSNGL

TDHPSFVPGLTPVTSSGFNGGHVELQQNNNNVTDSNWLIEQSQKLERQQQLQQQKLLELE

QTSVPAPVVPRSTSQVVAPTSLPSPPDSELDDFTEYGPGGVVKKKSHNLIEKKYRTSIND

RIGTLRDIVSKHFRDDKKVQKSAVLQKTIDYIRYLENSNRKLLEENRALKTQLMKQGYDE

RSMHGLGFYLGLTALNYGEFGLFLACDWLLELRYKVWKDNANKEETDPLLMSSIVKGYSQ

DIEVLNELTVHLPLAFTQESILDIAACKNEQQLRLAVCKGVEACFKNLVQVAAGRILESI

KAEREKKRMEEMLKLSGSLIDVDVPMLLLKLHSH

**>Amq14**

GATGACATCAACTGGTGGTGGCTTCACAAGTGGCTTTAGAACGATAAAGAGCGGCTATCTTAATAAGAGATGCAGAGTAAGATCCAGCTCGATTAGTAGAATGCATAGTCTATTATCTGGTGATCTTTGCAACACTGGCTTATGCCTGAGCCTAGTTTAGAATTTAGGTCTAGCTTGATCAGTTTATTGGGCTCAATATTGTGTAGACTCAGAATGCACGACTATTGCTATACTGCTTGCATAGCAAAAGCCCCAAGGGCCCCCCATAATAATGATGTAGTGATATTATAATCAAATTATTGTGTACACTTTGATTTTTATAGTGGTCCTGGAGGCCTAGATATGTGGAGCTGATATCAAATCCTAAAGACATGACTGGTATATATTTGCAATAAATATGAATGATAAGAAACATGGCAATTTCTTGCTGCTAGCTAGCTGTATGAGCTCATAGCTATTGCATTATTGAAAAAAAAACACAACAAAGCTATGTAATCAGGAGATTACTCCTACACTTGACTCAATGAAGCAATAGGAGTTATAGCTTACACAACTTATTGGGTTCACCGATTTTGCGATTTACCTCCATATATAGCTCTGAACCTACCTCCTGTGCTTTTGTTTACTGTGAATGGCTTATCACAATAATATTCACACATTTGCTATCAGTGCCAATCATTCAATGCATACTGTAGTAGAAAATTTGTGTGTCCTGATTGGCTAGAGTATAGTTTAACTAGACTATCTCACAAAAAATGTGAGTAGAATTATTGTTGTGCCTCATACCTGGTTTGTAGTGTAATCATTGTGCGAATGCATCAAAAGTGTTGACGAATTGGGCCAACTAATGCTAACAATAAGTAGTGGGAGAGGTAGCCTAATGTAAATATTCCAATGTCCACTGCTAGAATCAGCTTTCAGTTTGGAAGTAGCAGTCATCACAATCACAGATATAGCTTTATGGGAGTTCCAGGAAATGTTGTGATTAGTATAGTAATCAAGAGGGGAATTTTGTAGCATTTGTATTATTGAGAGAACTTGGTCTATTTGTATAATACAATTATAGTAGTACTATAATATCACTGCTTGTGTGTGTTTGTCTGCTTTGTGTACTCTCAATTCAATATCAATAGTTAAAGTGTTACCATAACAATCTTTAGGCCCACATTTTCGCTGTCTCTTACTCATATATGATCATTCCTCTCGATACGTGAAGAACAAGATTCGTGTTGACGACATAGTAACCGTGCGATCTGGAAAAACCTTCTCCTTTCAAATTTACCTTAACAATGGGATGTTTCTTTTCTTCAGAACGGGATCACTCGATAATCAATCAGCCTGGATGGCTGATCTTAAAATGGCCCTTGGAAAAGGTTTATTATTAAAATATTTGTGTGTGTGCATGTGTGGTCATGAGAAGATAAAATGGTATAAATAAACAACGGAGGGCTTGGGTTTAGGGTTTATAGGCAATGAGGCTGAAAGAATAGCATATATTAGGAGGGAAAGAACTGCTTATAAGCATAACAAAGTTAAAATAACACTGTAAAATGTTTCGTTGTATACTCTCTCTTTCACTTTATATCATGCCATGTCTTTATCATTTTAGTAACATCCCAGTTCCATGTCAGAGTACATGTAGCTGCACCATCATCCTGCAAGGCACTAGTACTAGACCATGCTCTCCTCTACTGCATTGGAGTCAACTTACCGACCCACCACTCTGGCCAGCTGGATAGAGTCCTTGTACTAGCCTCACCAGTCACCGATGTCAACTTACACGCTTGGAACTTGAGCCGAGTCAAACGCTTAATTGTAATGGAAATTGGAATACAAATGGAACTGTGTTCAAATTGTAGCGGGTGTCACGAGAAGAAGGTTGTCATGTTTCTGTTGCCGTCTGATCTCATAGAGGACTGTATGAACTTCTTACTCCATGAGATGCAGGCGACAGATGTGCAGCCTCAGCGATCGATACCCAATGACCCAATTATCTATGTCCTCCCTCACCAGTGTGGAGGGGCTCGCTCCGGCTCATTCTCTTTCCCCCAGCACCAACAACAGCAGCAGAGGCAGCAGGAGTCGTATGACTCTGCAATACTGCATGCTCTCAATAGCCACCCAGCTCCCAATTCACCTTTGCCAGCTGAATCACCTAAAAAAGACCCTCCGGCAGTGACACCCAACCCTGCCCTACCTCCTCGTAACCAGCTCCGTCCAAACTACCAGGAGTACATTGATAGAGGGGTTGAACTCAAGCCTCGTCATGTCAGTAGTAGTAGGCCCCATGTACCTGCTCGTTATAGACATGATCATTTTGAAGTCCCATACCACATCAGCCAGCGTATATACATCAATGATACCGGGACCATTAGACACGAGTCTCAAGTGCTTGGGAGTGGTAGTGGAGAGTTCAGGGCCAACAATGGCTCACCCTCTCAACCCCCTCGGGTCATAGTAACGGAGAGCAGTAAAGAGACAGGCTACAATCCAGTGTACATATCAAGTGATGATATTCACCCAGGGCCCACCCCAGCAGGACGTCCAGGGACTACTGGACCAAACAGACCAATCAGACCTGAATTTATCGGAAGAAGACGACCAATGCTTCCTCCCAGATCGTCTGATATTTCGGAAGATATTGATCCTCCTCCTCCTCCTCGCTCTTCTCCTCTTTCTAGTCGAACTCCTCCTCCTCCTACTTCAACTTCACCTCGGTCTTCCCCTTCTCCACCTCTCACTCCTCCTCTTCCTGCCCGACTGCCTCCTTCCCCTCCTCCTTCATATTCTCAAGATGATCCTAGTGACACAGGAACAGGATACGTCAATGTAGAGGATACCACTCGCTGCACTTCAGCTCTCTCGCTGTGCACTGATGATATAACTAATGCAGATTTTGCTCTAATGACAAAAGTGACATTTAAAAACATCATCCCTTCCCTTCCCCCGAGATCCAACAAGCAGCCTGTCCCTACACCAAGGAAGCAACAGAATTCGCATAAAACTCAAAATACTCCGTCAAACTCGGTTGTAAGTACCCAGCAGTCGTCATCGTCTGAGTACTCGTACGCCACTGCACCTTTTTATGGGCTTGGCATTGGAGGTTATTCCAGTGTTGTCCACCATACTGATCTGAGGCTTGAGAGACCAAGGTCTCAGAGCTCTGGTAGCTTGTGTTTGAACCAATTGTACGATAGGACTATGCATCACGGACAAGTCAGTAATGAGTGTTAACGGAACTATATCATTGTGTTTGAAATATATATGTGCTGTGTTTGTACTATTCGTCGTGTGAAAAACTTCACAATAATAATAATAATAATAATAATGATAATAATAATAATAATAATAATAATAATAATAATGATAATAATAGTAGTAATAGTAATAATAATAAATGTAATGAGACCATATTATTGTCTGTTTGTTTTTATTGTGTTATTGTCCTTTTTGATAACATTACTTTATTAAAAATGAACAACAAATTCAACAAAAAATAACTTCAAAGTTGTTTTGTACCTAGCTAGTATTGACCCCACTTTCTACTTGGTATTGTGTTGTGTAGACTACACAAACAAATTCATTAAACATATGGTGCATTTAATTGACAGTTTAAATCTGTTTTACTTAGCAATAACTAAATTTAGGTAAGTGTATCAATATTGACCATGAGCATGCTAATTAACAATAATAATTATAATGTCCCAATTTTTGTATAAATGTCCTTATTCAACTGTGAACATGCATCATGGTTTTGTTATAATGGTTCTGGAATCAACCCCAATTTATATTGCATTTTCTGATGTATATTGATGTCTCTATGCTATCTAGAAGTTTGAGCTGATTCCAATAAAAGTTGAAATTTTTTACAAATTTTTTAAGTTGCTCAAAAATTGGGCCATTTACTTTGTACTGTAGTAGAGGGTTGTTGGCAAAATTTCACCAAAATTAAAAAGAAGAGAATTTTTCATTTTCATAATTTTTGAGTTGATTCCAATCAATACTGGATTTTTTTATGAATATCTAAAATTGTGCCAAAGTCCCTGTGATATATAGTACACACCTCTGGCTAAATTTTGCCAAAAATGACTTGGAGAAAATTCTCTATTTTTACAATATTCCCAACACATACATGTTCTTATGTTGCGTAGAAAGTTTGAGCTGATTTTGTTCCAGTTTCAATTTTCTATGATTTTTAAAAATTGCTTTAAAATTGGGTACAGTGTCATGGACCACACAATTTAGCCAAAATTGAAAAGGAGAAAAATTGTCATTATTTTATTATGACGTACTTATGTTGCATACATATTGATGTCCTTATACCTGATTCTGACTAAAATTAAGCTACGGGGATTTTTGAAGGAAGCAAATTTTTAGAAAAAAAAACAATAACTATATTCAAATGTTTTTTCCAAAAATGGCTCCAAAATGCAAAATTACTCCCAAATGTTACTACATTTCATAATCATGTCTTTATTTGTATAGATACAAGACATTGCATAAGACATTGTTTATGGTTGTTATTCTCCTTGAGATGTTTAAGAGAGGGCGAGCAAGTTAATTTTTTGCTCTTGGTACTTCACCTGAAGGCGCTAGACAGTGTGTAAATACTACTAACAATAGTAATGATAGTAGAGAATTATGATTAATGATTTCACTCTTTTGTGATTTCCACATGAAAGGGTATCCTGCTCTTCCTATACAAACTTCCTGTTAAGTGGTCAGGCTAGAAAGAATAAAAGCTCTCTACCGCATTACATTAGAACACATAATTCATAGTATACCCAAGCTCAGAAACAGCAGCAGGATTTAGATTTGATATAGAAAGAGTGGTCATGGCCGTGTCAAGAAGACAGAAGGAGAACGATACCTTTAATGACATCGCGGGGACCCTACCGATAGAGGAGAGCGCCAAGGAACTGGACAAAGCCTCTGTTCTACGGATAGCGATACACTACCTCAAGCTAAGAGATGTTCTAGCGGACTGTGAAGAAGAAACGAGTCCCATCGATCTCTCATCAGAAAGAACTACTGGTAAGAACTTTTATAACGCAGGTAGACAGTAGCGTGTAGCTCATACTAAAATAGTTTGAGGCAAAATCTGCCAACTATTTTTGTACACACACAAAAACAAGCAAGCAAGCTTTAAAAGTTTTAACACTTAATTTACACCTTACTTTATTTACACCACAAGATGTATGCCTTAAAACTAGCACTTTATTTACATCTCTGAAGGTGTCAATAAAATGCCCAAACTTATTATTATGAATATTTTCAAATGGTATTAATTTAATAATTACACAATGTTATGTAATTGCTTTCCTGTCTTTAATAGAGCATGATGACCTGCACAACTTGGTGCCGAATTTCTCAAAGGATGTTCTACAGGTAAGAGATAAGAATTTCCTTTATATGGTATAGATAGTTGTATGTTACAATTACGTAGCTGGAAATGGGAAATTTATTATAGGAAATTATTATGCATTTAATAACAACCGATAAGAAATCAACTAATGATGTTATTTGTTATTATTCTGTAGGCTTTGGATGGATTTCTAATGATGGTTTCTAAAGATGGTCGTGTCCTTTATGCAACTGACTCTATCTCTCAGTACTTGGGCCTCAGACAGGTAATACTATAATTTAACTATGAACTATGTGAATGCGTGCACATACAAAACAAAACTATAGGCTATTTATTGTGTTTGATCGTGGTTTGACCATTGTTTCAGTATGCACTGTTCTGTTGGCAATATGAGACACAGACAATGTGTGGATTTAAAAATTAGATTTTCAGTCAAAACAGAATCGCTAGTAAACCTTATTACATACATACAGACAGACCTTGCAATACATCAGTAGATTCTTGATGTCAATGCATCAGTAGATTCTTGATAACAATCAACATAAAACTTGGCAGCTAGTAATTGGACAGGAATATCTAAAGGCTAAATCGTAGCTAGCCAGTTCCTACAAAGCATTAGACACTAAAGAATAAAGCCATTATAGCCAAAGGAAGCTAAAGTCTAGACAGTACACTGGCTAATGGTGCCCTCTTTATGTTCACATTACAATTCATACACAGACAAAACTGCTTCATAACACGTTTTGCATAGGGTGAATCTTGTTTCGTAACACAGCCAATGTATCATAATGCTGATAACAATGACTTGGACCATTCAAAATATCTACTTGTTTTTCCCTGCCTTTACATGTAGCAGTATAAATGACTAGTCTTAACACATAATCATATTATAAACATATAAATCTAGCTAAATAAGGAATAATTGAATAGACACATTTAGTGTGATATTATTATTGCTACATTAAAACATCTGCAGGTCGATGTGATTGGTGTACATGTTCAAGACATAATTCATCCTCAAGATTATTCAGAAGTTGCTACAATCTTCCAGAGCCAAGGCTGTGACATTGGGCCAGAGATGGAAGCACTCTGCAAGGACCCGCTGAGAAGGAACTTTGTTGTTCGTATGCGCTGTGCCTTCACACCCAGCGTACGCTCAATCACAAGATGCTCGAACTTTAAGGTACAAATAATATTAGCACATGTGCAACATTTATGCATCATATGATTGGATTGGAGGTCATGCATGTAATATCTCATGTAAGGTCTATTGAAAGATCAGTGAACCTTTTAATTGGGTTAGCTTGACCACAAGCAGAGATCCTATTCCATGTACATACACAGTCATACCTTAATTTTGCAATTATGTGGATTTTTGGCTACAAGGAATGCACTTTTATAGTTCATGACATGATTTTTGATCCTTATTTTAACTCAATTAATCCTAATTATGGTCCTGTTTGTTTTTAGCCCATATATTGCTCTGGATTTCTCAAGTTCTCGAACAAGAAGGTCAAAGAAGGAGAATTTGTTGGTATGGTGATGCTTTGCAAGCTGGCCAGTGTCATGAAGGTTAAGGAGCTCTGTGTTGGAGCTTTCTTTAAAACCAAACACTCACTGGATCTGAGCTACACTG

>genscan_predicted_peptide

XTSQFHVRVHVAAPSSCKALVLDHALLYCIGVNLPTHHSGQLDRVLVLASPVTDVNLHAW

NLSRVKRLIVMEIGIQMELCSNCSGCHEKKVVMFLLPSDLIEDCMNFLLHEMQATDVQPQ

RSIPNDPIIYVLPHQCGGARSGSFSFPQHQQQQQRQQESYDSAILHALNSHPAPNSPLPA

ESPKKDPPAVTPNPALPPRNQLRPNYQEYIDRGVELKPRHVSSSRPHVPARYRHDHFEVP

YHISQRIYINDTGTIRHESQVLGSGSGEFRANNGSPSQPPRVIVTESSKETGYNPVYISS

DDIHPGPTPAGRPGTTGPNRPIRPEFIGRRRPMLPPRSSDISEDIDPPPPPRSSPLSSRT

PPPPTSTSPRSSPSPPLTPPLPARLPPSPPPSYSQDDPSDTGTGYVNVEDTTRCTSALSL

CTDDITNADFALMTKVTFKNIIPSLPPRSNKQPVPTPRKQQNSHKTQNTPSNSVVSTQQS

SSSEYSYATAPFYGLGIGGYSSVVHHTDLRLERPRSQSSVYPSSETAAGFRFDIERVVMA

VSRRQKENDTFNDIAGTLPIEESAKELDKASVLRIAIHYLKLRDVLADCEEETSPIDLSS

ERTTEHDDLHNLVPNFSKDVLQALDGFLMMVSKDGRVLYATDSISQYLGLRQVDVIGVHV

QDIIHPQDYSEVATIFQSQGCDIGPEMEALCKDPLRRNFVVRMRCAFTPSVRSITRCSNF

K

>geneid_v1.2_predicted_protein_1|504_AA

HQQQQQRQQESYDSAILHALNSHPAPNSPLPAESPKKDPPAVTPNPALPPRNQLRPNYQE

YIDRGVELKPRHVSSSRPHVPARYRHDHFEVPYHISQRIYINDTGTIRHESQVLGSGSGE

FRANNGSPSQPPRVIVTESSKETGYNPVYISSDDIHPGPTPAGRPGTTGPNRPIRPEFIG

RRRPMLPPRSSDISEDIDPPPPPRSSPLSSRTPPPPTSTSPRSSPSPPLTPPLPARLPPS

PPPSYSQDDPSDTGTGYVNVEDTTRCTSALSLCTDDITNADFALMTKVTFKNIIPSLPPR

SNKQPVPTPRKQQNSHKTQNTPSNSVVSTQQSSSSEYSYATAPFYGLGIGGYSSVVHHTD

LRLERPRSQSSVYPSSETAAGFRFDIERVVMAVSRRQKENDTFNDIAGTLPIEESAKELD

KASVLRIAIHYLKLRDVLADCEEETSPIDLSSERTTEHDDLHNLVPNFSKDVLQALDGFL

MMVSKDGRVLYATDSISQYLGLRQ

**>Amq15**

TCACCAATTTTTGTACATGTAAATAAATTTTAACAAATATTATTTATTTACTACACGATTAGCTTTTATACTGTAAATGCACATACACTTTCTTCTTTTGCTTTAATTTTAAAAAATACATGTATACACATTTTCAAAATAATCGTAAGGTTTCTTCTCAATCTTTTCCGAGCAGGGTCTCATGGTCTTACCTTGGGGCGGGTCAGGCTCCATGGGTTCTGTCCTGTACTCCAAGTCATTGTACGAGTTAGGGGGGAGGGGGATAGGAGGACGTGGGGACCTGGTCGAGGTTGCAGGAGACGTGGACGGGACCTTTTTCTTTGTGTGTCTTCGCTCAGAGATGCCACTTCCACTGGAGGAAGCACTACTGGACTTGTTACCCATTTATAACTGATGGAGAAAAAAAGAGAGAGAAAGTATGAGATTGGAGGACCATTTGTAACCTAATAGTGTGATTTTATTGCACTTTTTAGTGTCATGATAATCGCACAAAATAATAATAATTTGTACATCATTAAAATACGATAAATAGCGATAGCACGTCCAGATTGTCTCATAGCAAGGCTACAAGGGTTGACGACTCACCTAGACTAGCATAATACCGGTACAAGAGTGAGAACAAGGAGATAGGAACAACAAGACACGAGCAAAACAGCAAGGGAACTTCTATGATTATGCTTCAAGCCGACCGGATTCTCTATGCACGAACTTTATGCAGAAAAAGCAACTGATCACGTGACCACAAGACCCAACCCACCACAAGTGGCCATTGTTGTCACATGATTGTACACCCACGCACCAGCCCCGCCTTTCTTTCACACTAAAATCCATTTTCTCTAATTTTTCTTGCTTTGCATAAACTCCTGCCTGCTTTCCAGGCTGCCAAGTTAAGCTACCTTGAAAATTTATAAAAATCCTTAGTTTTATATCGACTAAGAGAGCCTGAGAAAGGGCAGACATTTTACACTGGCAATCCCTGAATGCCAATACTACTATAGCATAGCGCTGCATTGTTTGCATTTTTATAATTAGCCACACCTTGGATATTTTTATTGTGTAACAACACGGGACTGTGATCATGTACAAATAGTGGCAAAAAGTTCCCAGCCAGTCGTCCCACGACTTTACAGCCTATAAAAAGATCCCGGAGCTTTCCCTGCGTTAAAAAAACTAGGACCTTGTGCTATCCCTCCATTGTTCGAAGAGTTCTTTAATATTATAACCGTAAGTGAGTGCAGCGAAGCAAGGGATGAGATCATGCATGGTGATAGAGAGAGGCGCTCACACTCCCACGTTCCTTTATTATGTGAAATATGCCTTCTGTATCTTTGCCTGTCACCTTCTCACTTCGCATTTATGTTTGTACAACACGCAAGCGTAGAACTTCTTGTAGAGATAAAGCCATTTTGAGATAATTATGAATTGTTATGTAACAAAGTTAGAGAATAAAGATAAAATGCACTTGTTAGACATCTTTTCTTACACAATAGGCAAGAAAGTTTCACAATAAAGTGCAGCTGCAGTGATGATTGGAATTTCACTATAAATACTTTTCCGCTCTAAAATACTTTCATATTGCCTTGTCGAGTACTCCTTTGCTTCATGTTCCAGCTGCCATAGTAGATTTCTTTTGTATACACGCCTATATCTGTTATATTTTCATGGCAGCTGGCATGAATACAGCATGTTTGTAAATAGGACAGTCTTTTTGCAGTGATTCCATCCTTCCTTATTATAGATGTTTCAATCAATGTGTTTAGCAGCTGCATAGCTCTGCATCAAACAGCAGCACTTGAGAGTTTTCGTTTCTTTCTCCTCTTTCCCTCTCTCTTTTTCTCTCCCTCTCTCTCTCTCTCTCTCCCTCTCTCTCTCTCCCTCTCTCCCTCTCTCTCTCTCTCTCTCTCTCTCCCTCTCTCTCTCTCTCTCTCCCTCTCTCTCTTTCTCTCTCTCCCTCTCTCTCTCTCTTTCTCTCTCTCTCCAGAGGTAGATCATGGCTTCCAGCCCAGTGAGTGTTGGCGAAGATGACGAACCATTGATTGGAGGAGCGAGAAAAAGAGGCAAAGTTCGGAAGGAGAGTGAGCACAAACGTCGCCTTATGATGAACCAGTATTTTGACGAACTGGTCATTCTCCTCTCCATGGTAACCGAGACCGTTTCCTCTCGTAAAATGGACAAAGTCACGACACTACACGAAGCTGTTAGTTTATTTAAACTATACTATGACTTGGACCAAAGCCAGGGCCCAACTTCAAAAGCTAACGAACAGCTCCATCAAACGTATCGACCAGAGTACCTCCAATTTGGCGATGCTGCCTCGTTTATTCTCGACTCACTTGGCGCCTTTTTAATGGTTTTATCAGAAACAGGGCGGATCCTATACAGTACTGACCTCATCACTTCTCTGACTGGTTACCTACCATGTAGAGTTGTGGGTCAAACCATTTACGATTGTCTCCATACAGACGATCATTTCATCATTAAGGATCTCTATCGGGTATCGGGTGAGGAGGGAGTGGCCTGGAATAGAAAGGACTCGCCCATTATATGTTACCCTCCAAAGAGTCTCCGGTGTCGATTTAAGATATTCTCAAACGACAACTCAATGTCGGGATCTAGCAGATCATTCTCTTGCTTGTCATATTTGAGACAATGGAAGGAAGTTCCAGCTGATAATACCAGGAGCCCAATGGGTAAGTAAGAAAAGTATGAGCATACCTTTTCTTTCCTCTTCTCTCTCTCTCTCCCCCTCCCCCCCCCTCTCTCTCTCTCTCTCTCCTCTCTCCCTCTCTCTCTCCCCCTCTCTCTCTCTCCCTCTCTCTCTCCCCCCCTCTCTCTCTTTCCTCTCTCCCTCTCCCTCTCTCTCCCCCTCTCTTTCTCACTCTCGCTAAATTGTATAAACTGGTCATGCATGAAGTATTACCATTTGAAGAGTTAATGTGTGACAGTGCTAATAGCATCCATGTACATATACATGTATGACGCGTCTACATGTAAATTAATGCTGACTAAGATGACATTCATTCTTGTTGAATGTTCAGCTCATGTTACAATTAAAGTACAATTTAAAGCTAGATGTTTGGTGATACTCCCCCCCCCTCACTCTCTCTCTCTTTTTCATTTATTGCAACACTTTAGGTGATTCCTGTAGCTCAAGGGACGACACTGGACTAATGACACCTGACACTCCGGTCTGCTGCCTGCTCCTCATAGCTAAACTGGACGAGAACCTCTCGATAGTTGATGAGCCATTCATTAGTAATGGATCCATAGAGTTCTCTTTTGACATACGAGTCAGTAGAGAAGGGAAGATACTGGACATGAGCAAGCAGGCTAGTCTAATTCTTGGATACACTTCAAATGAGCTTGTTGGGTCTCTCTTCTTTGACTACGTCGATCCATTTCATCTCGAAAAAGTCTCGGAGTCGATACTCGAGTTTTTAAAGAAAGGACTCGGAGTCTCTCAACCTTATAGACTCATATCTAAAAGCCTCAGGTCGGTGTGGGTGGTATCTAAAGGATTCCTCTCGTATAATCCTTGGAATCACAAACCAGATCATATTCTACTACAGTGTAAGGTCCTTGGATGTGACGAGATACTCCCAGAGAGTCGGTTTAGTCATGATAGTCGATACTTGCCTGATCTTAAAGGAAACGAGTTTTACAGACCCGGCCCCGTGACTCCTGCTCAACCAGTTCCTGAACCAAGACCAGTTAGAAGACAGCAGCAGCAGCAGCCACCTGTTGCTCTACCACCTAAACCACCTCCTTCTCTCCCTCCCCTTCCTCCTGTATCCCTTGCTCCTGTCCAACCTTTGACCAGCCCAGCCAATCAGCAGAGACACGAGGAGAGAAGAGGAGGAGGAGAGAGAGAGAGGTCTCTTTTGGATGAGGTGAAGAGAGAGTTGGAGAGGAAGAACCAAGAGTTGTTCGAAATGCAGCAAAAGGTGCTAGCCCAGCAGCAGCTTATAGAGCAAGAGAGACATCAGTTCTATCAAGTCACAAACCAAGTCATGAATTTCATCGGATCTCAGCAACAGAACATTGGGACTAACACAGACCCATTGAGCGGGATGGATATACATCCGAGTATGGCCATGATGATCCGTAACTCGTCAGTACAAACCCCACCCCCTCCTCTCGGCCAGGGGAAAACCCCCAACTTTCAGTTATCGTTGCAACAGCAGCAGCAACAATCGTTTAACTCGTCTCTATCAATGCCTACACCCACTACTCCTAATTCTGCTGCCAGTGAGGGTGGGATGATGTCATCGGGCCAACTGACCAATCAAATGATGGGTGGTATTCCTCCTCCGGGTCACATGAGCACTAGCACAAGTCACATGACTCAAATGACCAATCAGATGAGTCCGATGATGCCACCCCCACTCCCTCCATCAACTCACATGGCTAGCGGAGGATATCCAAATAAGGATATGTCATACAATAATCACATGACTTCTGGTCAGCCGCCTGCCCAAATGGACATCCCGTTCCCTGGTATGCTAAATCAAACATCGTCTGCTAGTTCACTATATGCGTCACGTGAAACGTCAGCTGCATATCCACCAAATCAAGAGAGCATCCCTCCAAACGATAACTACTTACCGTCTTACCTTCCCTCAAGTTTATGTGAAATGACTGATCCTATTTCTGCTCCTCCTCCCTCGTTCTCTTTTGCTTCTACTACTCCTCCCTCTTCCTCTCTCTCTAATCCAGCCGATCAGCAGCAAAGGCTCCTGCTTGATCATCTACAGCAGTTGTATCGCTCTTACAATGGGACTAGTTGAATTAAATTAATTCCTGTGTTTATGTACATGTACGTGTATTTATTATATTGTCGTGTTTCCCACTTAAAATTTAGTATTTAATGTAATATAATAGTACTACAGACAGATTAATGTGTCGAAATGATTTAATTATAAAAATAACAAAAGTCTAGCTTTGATGACCATTAGCCCTGCCCACTTAGTAAACATGGCTATCCTTTTATCTGGAGGAGATAGTTGTTTTCTATTCACTATAATCCTCCTCTTTCTACCTCTTCCTTCTCAAGGAGCTCCAAAAGTAGGAGGGAGTCCCCAATGTGCCCTGTATGAGGGATTCACCGACAGCAGGTGCTTGTCTAGCTTCAATATTGACTCCTCTCTAACTGAGAGTAAGACTGCTTGGGTGGTCGAGTTCTATTCAAGCTGGTGCGGGCATTGTCACCACTTTTCACCTACCTGGAAGAAAGTGGCGAGCAGGATAAAAGGTAAAAGGAATGTCGATTGATTCACATTGTTTGTTGTTGTTTATGTTATTAGAGCCATTATTTAGAACAAACCGTATCAGAAGATACAATAATTAGTGTATTATAATTATTTTGCTGTCTCCATTTTATTATGATTAGGATGGTCTAGCGTTGTCAGGATGGGTCAAATTGATTGCACTCATTCAGACAATAGAGCGATCTGTGGTCTGTATCACATCCAGGCCTTTCCAACGATAAAAGTGAGAGAGAGAGAGGGGGGAAGGAGGAGAGAGAGGGGGAGGGTAATAACTAATGAGAATGGAATAGTAACACATTGTCACTGCAGACTTTTTCATGTAATTATTTACTATCAATTGTTGTGACTGATTTGTATGTCCATGATCGTAATCAATTGTGTCTAGCTATTCAGTTCAATGACCAAGAACCCACACTCGGAGGGTATCAAGACATTTCTAGGTAAGAGAAAGAGAGGGGGGGTTTAGCACATTGATTTTGTAAATGAAAGAGTTAGAGTGACATTATGTTCATGCCAAAATAGGAAGAAGATCAGAAGAGTCACTACTGGAGGCTATCATCAGACTTGTTGAGTCTGAATCACTTGTTAAATGTCTGAAGCCATTAGAAACAAAGTAAGAGAGAGAGAAAGAGAGAGAGGGGAGAGAGAGAGAGAGAGAGAGGGGAGGGAGGGAGAGGGGAGAGAGAGAGAGGGAGAGAGAGGGGGGGAGAGAGAGGAAGAGGGAGGGAAGAATAGCAATGAAAGATGCAAGGACATTTACACAATGTTAACAGACTACCAAGCACTGATCCCATCAATGTTCTTGTCTTTGAAGAAAAGAAATCACTCCTTGGTTCAGAGGTACAGCAAGACATAAACATAATACTGTGCTGCAGTAATTCAATGAATTTTCAACCCATTAGATTATCATGGACTTTTTATTGGAGAATGTTAAAATACGTCGTGTACTACCAGATAATGTAAGGATTATATACCATAATAGTATTATTTATTTTATTATTAATTCTATGTACAGACAGCTCTGTCTAGTCAGTACTCAATACTTCAATTGCCATCACTTGTCATACTACCATATTTACCTAACCAGCAACACCATACCTTTATACCAAAGTAAGTTACTATAAAACCTGTCAAACCGGTTTATCCTGTTTGACTGGTATAGGAGAGAGGGAGACGAAGGCCAAGATGAAGCCGTGGCTTCCGTGAGAGAGTTCTTTGAGGAGAAAATTACGGAGGAAGTCTTGAATAGAAGGAGAGGGAGTTTAAAAGAGAGGAATATTCCTATTGATAAAAATGAAAAGCCAATTGAAGCAAGTCTAACAGTTAATAGGTAGGCAGCTGCTAATTAAATATGCGTTCATGCGTGTGTCCATTTAGGGTAACAATGACCGACATTTTAAGCACGATATCTTATTGCTTGAGATATGAGGTTACTTTGAAGAAGATCATAAGTGGAGAAGACTTTTATACACTGCATCAGTTTATATATCACCTGGAAAAGGTTAATATGACTACTGCAAGTACATGTACCATCAAATGTTTTGAAGAAAGGGCTACAAAATAGTAATTTTTATTGCTTTTTCTGATGCATATACATGTACTTATTTTGCATAGAAAGTTTGAGCTGATTTCGATCAAAGTTGGATTTTTTACAAATTCTCCGAAGTTGAGCCAAAGTCCCTGTACTATAGTACATGGTTGTTGGCCGAATTTTGCCAAAAATGGAAAGTTGAAAATCCTCCATTTTTATAAATTTTTCTGATACATACACATGTACTTATGTTGCGTAGAAAGTTTGAGCTAATTCCAATCAAAATTGGATTTTTTAGGAATTTTTAAAGTTGCTCCAAAATCCAAATGGCCAATAAGCTAATAACTCCTTTACCAGCTGGAGGCCATAGATATTTT

>genscan_predicted_peptide

MASSPVSVGEDDEPLIGGARKRGKVRKESEHKRRLMMNQYFDELVILLSMVTETVSSRKM

DKVTTLHEAVSLFKLYYDLDQSQGPTSKANEQLHQTYRPEYLQFGDAASFILDSLGAFLM

VLSETGRILYSTDLITSLTGYLPCRVVGQTIYDCLHTDDHFIIKDLYRVSGEEGVAWNRK

DSPIICYPPKSLRCRFKIFSNDNSMSGSSRSFSCLSYLRQWKEVPADNTRSPMGDSCSSR

DDTGLMTPDTPVCCLLLIAKLDENLSIVDEPFISNGSIEFSFDIRVSREGKILDMSKQAS

LILGYTSNELVGSLFFDYVDPFHLEKVSESILEFLKKGLGVSQPYRLISKSLRSVWVVSK

GFLSYNPWNHKPDHILLQCKVLGCDEILPESRFSHDSRYLPDLKGNEFYRPGPVTPAQPV

PEPRPVRRQQQQQPPVALPPKPPPSLPPLPPVSLAPVQPLTSPANQQRHEERRGGGERER

SLLDEVKRELERKNQELFEMQQKVLAQQQLIEQERHQFYQVTNQVMNFIGSQQQNIGTNT

DPLSGMDIHPSMAMMIRNSSVQTPPPPLGQGKTPNFQLSLQQQQQQSFNSSLSMPTPTTP

NSAASEGGMMSSGQLTNQMMGGIPPPGHMSTSTSHMTQMTNQMSPMMPPPLPPSTHMASG

GYPNKDMSYNNHMTSGQPPAQMDIPFPGMLNQTSSASSLYASRETSAAYPPNQESIPPND

NYLPSYLPSSLCEMTDPISAPPPSFSFASTTPPSSSLSNPADQQQRLLLDHLQQLYRSYN

GTS

>geneid_v1.2_predicted_protein_1|784_AA

MASSPVSVGEDDEPLIGGARKRGKVRKESEHKRRLMMNQYFDELVILLSMVTETVSSRKM

DKVTTLHEAVSLFKLYYDLDQSQGPTSKANEQLHQTYRPEYLQFGDAASFILDSLGAFLM

VLSETGRILYSTDLITSLTGYLPCRVVGQTIYDCLHTDDHFIIKDLYRVSGEEGVAWNRK

DSPIICYPPKSLRCRFKIFSNDNSMSGSSRSFSCLSYLRQWKEVPADNTRSPMGDSCSSR

DDTGLMTPDTPVCCLLLIAKLDENLSIVDEPFISNGSIEFSFDIRVSREGKILDMSKQAS

LILGYTSNELVGSLFFDYVDPFHLEKVSESILEFLKKGLGVSQPYRLISKSLRSVWVVSK

GFLSYNPWNHKPDHILLQCKVLGCDEILPESRFSHDSRYLPDLKGNEFYRPGPVTPAQPV

PEPRPVRRQQQQQPPVALPPKPPPSLPPLPPVSLAPVQPLTSPANQQRHEERRGGGERER

SLLDEVKRELERKNQELFEMQQKVLAQQQLIEQERHQFYQVTNQVMNFIGSQQQNIGTNT

DPLSGMDIHPSMAMMIRNSSVQTPPPPLGQGKTPNFQLSLQQQQQQSFNSSLSMPTPTTP

NSAASEGGMMSSGQLTNQMMGGIPPPGHMSTSTSHMTQMTNQMSPMMPPPLPPSTHMASG

GYPNKDMSYNNHMTSGQPPAQMDIPFPGMLNQTSSASSLYASRETSAAYPPNQESIPPND

NYLPSYLPSSLCEMTDPISAPPPSFSFASTTPPSSSLSNPADQQQRLLLDHLQQLYRSYN

GTS

**>Amq16**

CCTTCAAAAAGTGGCTTTGAAATTGCATGTTAACTTAGTCCAGTGTTCCTTTAAACAATCAGTTATACCATTTACACATAAAGCTGATTAGACTTCAAGTGTTGCATGGGATATATAGAAACCAACATTATTAATTCATTTATGATGGTTGAAATAAACCTAGGCATCATATTCCTTGTCTATTCATGATTCCCTATTATTATTATTATTATTATTATTATTATTATTATTATTATTATTATTATTATTGTTATTTTTATTATTATTATTATTATTATTATTATTATTATTATATTATTATTATTATTATAGACTTCAGATGCAATGTCATACGTGGCTGGGTATACAGTAGCTCATGATGTCAGTGCCAGAGACTGGCAGATGAAGAAGAATGGGAAGCAATGGCTCCTTGGGAAGACCTTTGACACTTTCTGTCCTTTAGGACCAGCTCTTGTAACAACACCATCCATCTCAGGTAAGGATGAGGGAGAGAGGATAAATACCAGCATATAATACAGAAACTTAATTTTTGATAATATAATATAGAGGCAGCCAAAATGCATTAGTTAAATAACAAAGTCTGCCAAATACTAGCCACACCAAAAATCCAATGTTAATTATGCCAACGAACATAGGATCATTTAATTATTTATTCTTGTATTTTCAGATCCTCATAATTTGGGCATTAGGTGTCGTCTAAACGGTGAGACGGTACAAGATTCAAATACCAACCAGCTGGTGCATAAAACTGAAGGACTTGTCTCATACATATCACAGTATGTCACTATACGTTACACCGTTCAGTATACCCTTGTATACTTCAGTATACTCTAGTATATTTAATTTGTATATTTTATTGCAGATTTGTTACACTTAAGCCAGGTGATGTTGTTTTAACAGGCACGCCCCCAGGTGTGGGCTGTTTTCGTAAGCCACCCCTTTGGCTTAAGGTACATGTATAAGCATCAGCAATGATGTCATTGAGTTGTAATGTTGTGTTCTATTTTTAGAAAGGAGATGTGGTAGAGTGTGAGATTGATGAGATAGGAAAGATCAGAAATGTCATACAATGATATTAGTGATATTAAAGCTATGCACCAGCTACTGCTGCCTCACACACACAATCTCATTTAAACATTTATTCGTTTTATAAGCTACTTTTATATTTTGTTTAATAACAATATTTGATTATTAAATATAATTACTGATTTTCTGCTGTAAATTTAATCTATGCTACAGTTGGATATTTGCTGCCAATTGTTTTATGACAGATTTTCAATTAGAACTACTATATGCTAGTATTTAATTATTTATCAAAAGCTTGTGAATGAACTATTCAAATAGACAGTTCATACAAACTTTAAGGATTTGTGACACAAACAAATTTTAACCAAGTCCCTTGTTTTAAACAATGCCTGTTATAACTGAATTTTATTGCATAATAATTTAAAGGTAGAAAAATTATCAAAGAATAAAACAAAATGGGATTCTCTAATCCATTTTCAAAGGTTTATTATTCCATTTATAAAGCAATGTTAGTATTAGTACAGAAAATATGATAAATAAGGTCCAATATAAATTATACTAGCTCTAGGAAGCTGAGGAGGAGGATGATAATAGGAACTGGTTGGCGAGTGCTGCAATATCCTTGTGACTGTACAGCTGCAAATGATGACCCCCGGGAGCTGTTACAACCTTGATCTAAGGAGAAAATGTAGTTAATAATAAAATATTTAATAATAAACTAATGCATGGCAATTAATAACTTGCTTTGTCATTTCCAGCATTTTCTACTTTTTTATTCAATCCTTCCTTGAAAATACTCTCCTCAGCTCTAGAGAAGGTACATAGATACGGTTCCAATACAAGTAAAAGATGAAAAAGAGAGAGTCATGCATGTAGGAACAGAAAGAGAGTGAGGAAGAAATAGATGGAGTTTAGCTCACATTATTAAAAGTGTTGGACATTGAATTGCTTTTATAAATTCTGCGTCTGTTTCATGAGGCAAGTACAACAGAGATGGCTTTGAGAAAAATAAAATAAAATAAAATAAAATAAAATATAAATATAATAATAGTTAAAATTGCAAATTCGTAATTACTTCTTTTAGTCTGTTGTCATGCGAAAATTTGTAGCCTTCGTCAATGCAGATAGAGCCTCTCCTTGCAATCACGGCTACGTCATCCCTTGATAAAAGACTGTCCAGTTTCTTATTAGCGTCCCACACTCTGGTGAAGGGGAGAGAGAAATAAAATTAAAGAAAAGGACAAGTAGAAAGGTCAGGCATAGTTGTTGAGCCATTCATGTCTCTTTTCAATAATGAATGAGCTAGTTTTAAAAAACATCTCTGAAGTACAGTATAAAATAACAAAAGGCTTTCATTATTAAAAGAGGATGAATTGGAGCCAATTGCAAAAATAAAAAGCAATCCCCACAATACTAAACAATTCACACTTTCTACAAAACAACACCAACATGCATGATGTAGCTGAGAATCTTAAAATTCTTTCCTTTTCCATTTTGGCAAAATTTGTCCCAAGACCATGTACTATAGTACAGGGACATTAGCCTGATTTTTGAGTAACTTTAAAAATTCATAAAAAATCCAAATTTGATCAGAATTAGCTCAAACTTGCCACACAACATAAGACCATGTATATGTATCAGGAAAATATATAATAATGGAGAATTCTCTCCTAGTCATTTTTGGCAAAATTTGGCTAAATCCCTGTACTATAGTACAGGGACTTTGGCCCGATTTTTGAGTAACTTTAAAAATTCATAAAAAATCCAAATTTGATCGGAATTAGCTCAAACTTGCCACACAACATAAGACCATGTATATGTATAAGAAAAATATATAATAATGGAGAATTCTCTCTTAGTCATTTTTGGCAAAATTTGGCTAAAACCCCTGTACTATAGTACAGGGACTTTGGCCCGATTTTTAAGTAACTTTAAAAATTCATAAAAAATCCAAATTTGATCGGAATTAGCTCAAACTTGCTACACAACATAAGACCATGTATATGTATAAGAAAAATATATAATAATGGAGAATTCTCTCTTAGTCATTTTTGGCAAAATTTGGCTAAAACCCCTGTACTATAGTACAGGGACTTTGGCCCGATTTTTAAGTAACTTTAAAAATTCATAAAAAATCCAAATTTGATCGGAATTAGCTCAAACTTGCTACACAACATAAGACAATGTATACATATTAGAAAAATATATAAAAATGGAGAATTATCTCCTAGTCATTTTTGGCAAAATTTGGCTAAAATCCCTGTACTATAGTACAGGAATTATAGTACATCTATAGTACATCAATATAGTATTAATACCTAGTGACTATCTCATCCCATGTCTTGTGCACTTTATTCTCAGTTGGAACTGGTGGAGAATTAAGAGATCTGCCTAATAATCGAGGGAGATCTTCCTAAATTATGCACAAAAAAACAATAATTTTTATTCTTGCGGCACTTGAGTACTTTTGGTCTGGACATGATTCCAATGTTTTCTATGAGTACCATCTTCTGTACAAGTTCAGGTTGAGCTGCAGCAAACTAAAGAATAATAAAAAAGAGGGAATAGATGGAAGAGAGAGAGAGAGAGAGAGAGAGAGAGGGAGGGGGAGAGNNNNNNNNNNAGAGAGAGAGAGAGAGAGAGAGAGAGAGAGAGAGAGAGAGAGAGAGAGAGAGAGAGAGAGGGGGTATCGTTGTGTAGACCTACATATGATGCAACTCCAGCTCCTGAAAGACAATATCTCATTAATAAAAATAAATAATAAATGATAATGGTTTACCCATGGAATGACAGAAGAAAGAAAACTTGTCCCAACCCAATCCTATGGCAAAAAAACATAAAAAATGGTAACTGTACACTACAATTAAATCAAAAAAAATTCTACACATATATATATAATGGATGTATATTTTTAAGAGTGGAGATATATTTAAGAAACAATAGCAACAAAACAATCACATAATGAATGATGTGGACCTTTTATGACATCACGAACATCAACAAGGTAATCAACGAAATGAAGTCTACAAGACTGGGACCTGTGGTATGAGTGCCCGTGACCAGGCAAATCAATAGACACAAAGTAAATATCTATAGAAAAAACATTATACTGCTATTAGGGATACCGTATAGCTCCAAATTTTCGTCAAATTTATCAGGTACTTTTGCTTGATGAAATTTGTAACCAACAAAATTTTTACTTCACGTGGGTAGTATATCAAAAATGACAGAATTTTTATTAATGAAAATTAATTTTAACAAAAAATTAAGATTTTGACAAAATTTTCGACTGAGGAAAATTTGGAGCTATACGGTACTACATCACAATATACTGTAATTTTATGCTGTTAATATCTGTTGATACAGGTCATTAGATTCTGGACTTAAAACAGCAAAAGATAAACCATCTTATATCATAAGTATTTTGCTCTTCCATGACCATGCATTATCATTAATGCTGGCCATTACGTTATATCATAATAATTATTATCATACCTTTGTCCAACAACGGAGCAATGTTATCAAATGTAGCTGCATTATTTAACCAACCTACAAGAGAGAGAGAGAGAGAGAGAGAGGGAAAGGGTAAAGTAATTTAAAATATTTGTTACCATGGAGTCCAAGGACTGGTTGGCCTTTTGGATTGCCCCATGCTTTCCCGGCAATATTCCCGCCACTTGAAACAGGGATGCTGACTGAAAATAATAACAATAATAATAAGAGGTATATATTACTGTAAAACATTTTTACAAACACTAAAATTTCAACAATCAAATCCACTACAACATGCTAGCAAAAGAACTCCACCAAAAAATGAGCTTGGAGGTTGCATTTCATTTTTGGTCGAAAAACTTTTTTGCTAAACAGTAAAACAGAGATGTACTATGTAAAAAGAACAAGGAACTCCTAAAAGAATGACACATGCTTAGTTCTTTGACAGGTGGCAGTTGATGATGATGTCAGGTTTGTTTAGACTCTGCCATGCAACAGTAGCGTCAGTCTCTTTATATATAAAGGATGGGAGGCTCGTGATAGGATATATGATACGACAGAGAACACTGAACTATTATTCAATAGCTCAATAGGATATTAGATACAGCACCTGGCGTGAACTCTTCTTGTAAAGTTCCTCCCGGCTCTGCCATCTGTTAAAACCATGGAGATGTTATTATAAGGAAGGGAAGTACTCACAAATATTGTTTTATATCTCAGGATTTGCTTAATCCTTTGGCTTTGGCTTGCAGATCTCGTCAGACTTCTGCTGAGAAGAAAAGCAGAGAACATTAAGCCACGCCTTAAGCCTTGTGCTGACAAGAAAATAATTTTAACTCTTTAATGAACTTGTAGCCTACTTTGCAGTTTGCAGCAAAAACTCTAACAATTAACAACTGATCAGTGGATGTAGACAAAAGTAGCAAACAAGTTTTTAGGAGAAAGCCTTTTGTCACTTTCTTGCAATAAAAACCCTGCAAAATTTACGTATTAATTATTTAAGAATGAAAAGTTTAAACACAACAGCCTAGGCTGGAGCAAAAACAGCTGTAAAGCTTCATATGCAAGCTACAAATGCAAAGCTTAGAGTTATAAATGCATCAAATCATATAGCTGCTAGATTTAGCTGTTAGATTTAGCTGCTTACTCAGATCTAATGAATATTAATTAGTTGGTTGCAATAATTTGAAGCCCTATGCTAGCAGGGGCGTGGACACCCAGCTAAGGCTAGGCGGCTGAGAGTATAAGGCCTAGGATTGACCCTCTCTCCTCTATTCCTCACTGTACTGTAGAGTCTGCAAACAGTTCTCTCTAGAAACTGTAGAGCAGGAGCAACTTGAACTGGAGAGCAACCGGTGCAGAGCTTCGTTGAGCAATCAGAGGATTAAGTAGGTAAGATCGCACCCCAATCATGTAGATCACCTGCGATTTGAACCTGCCTAGCTAGCTCCCACTTCACGGGGCTCCTAGTGTACCTGCAGGTATGTTCAAGCAAGCTCCGGCTCGGGCCAAACAAGTTGGAATGGGTCGCCTTTGCACGCGCAATTAAGCAGCCATTCCTGAGCCTGCAGCTTGTAGAATCAGTCACCTGTGTAAAAGTTGTTTTTTGAGTCCCCATAAGTTTGTATATGAGTTCTCTCCTTTACATAATAATGACTAGCATGTGCATGTGCATCCCTAGATGTGCTGGTGGACAGATCTAGCTGGCTAATAATGAAAGGTAGGCTATTTGTTGACAAAATTCAAGTGAAAACTATAAAAACCCAGAGTCGATCACAGGCCTGTACTAGTACAAACCTACCGGTTGAGGGTTTGGCGTTTTGGGAATACGGATGCTCACTTTTTGTCTTATCATGGGGATTGCTGGTCAAAGTCGCCAAACGTAAAGTTATTGCTTTCGCTTTGGTACCACCACTGGGTATTTATAGCAAGGAGCATTACATGCATTATGCATGTAAGGCCACGCATGCAACATGCAGACTCGAGTTACTTAGCTGGAATTACAAGTGAGTGCTGTTCTTGGCAATAACTGTTGCAGAGTGGTACAGGCAGGTTGTCATAGTGAAGGCAATCAATTGCGAAAACACATCTACTATAGACCTGCATAGACTGTTATTGTAAATACTGCATTGCCTTTTAGATGTCAGCATCATTTTCGCAACAAACCCTTTTGTTCAAGCATTCAGTCATTATCATATTCACTCTTTCTTTTCTATAGAACAATATATTCCTGTCACCTTGACACAGTTTTTCTCACACGTGTGACTTGCATTGGAATAATTCTAAAAAGAAATTGAAGCAGCACAATGGCACATGAGTCTTCTCTTAGCCCTCCAGTCCCATCAGGGGAGCACATACCTCCTCCACTCATCAACCATACTCTACCTCAAAGGTAAGATGTATGTGTGTGTTAGTGTGTGCTTGCATGTAAAGTATTATATAATAATATGCCATTTAGCAAGAAACAACCTTCCACAAAACAAATTATGATAGTGGTATTATAGCCAGCCTTTACCTTACCCACTGACCAGAAGCCAGCCTATGTGTTTGTGTGCAATTTATAATTAAGCAAATTAGATTATGGTCTAGTGCAAAGACATATGCAAAAGAAACCATGTAAAATGGCAGAATGCGTTCCATAATTAGGTTTACAGTCCGTGAAATCACAAAATAAAGGAAAATGTAAACTTCTAATTATTATAGGAATTTCACAACAGTCGAATGTTTATAGTGCATGTTTTTGTGATAGCTTCTCATAACTTTAATCAATCATTGACTACTGTATGCAAGCATACAGATATAGCCTAAGATTATACACCTATTAACAATATTCATAAATTATCTTAGACTTTCCTGTCAAGCAGTAAAGCCTATACTTGGGGTAGTTGACATTCTTTCCTCGACCACTGGAATATATAACACAGTCAAGTTGTGTCTATTAATGATTAAGTCTATTCATGGATTTAGATAGCCTGGCTTACATAAAAACTATCATACTATCTCACACGTTGACTGTCAAATTGTTACTATACTCACGCATTCAAGGCAACAATCAATCTGAATTGCAAGCTTATCACGTATATAACTGCCCTGCTGGCAATATAGTGTTTACTGGCAACAGTGAAAATGATTAGAGCTCATTGTTGGTCAGCTAAAAAGATCTCCCTACAGATAGCATAATCAGTCCATACAACATACATTCAATGTATGCACTTTTATTATCAGACCAGTTTCAGTAAAAAGTAGATAGCAGGTCTGTACATTAGACTACCTGTCTAGAGGCTTGGTGGTGGCTGAGGTCAATAATTAGCATTGCAGGTCTCTGAAAGCCATCCCAATGAGCTACTAGACTCTCAATTACTTCCCTTTCCCTCTCTCTTTCTCTCACGCTCATCACAGGTCTACACCGTATTACATTGCAGCTGCTCAGTTTGAGAAGCAGCCCCCAGACAACATGAGGAAGAGCAACTTTTTTCATTTCATCATCTCTCTCTTTGATGGGCAGCAGCATCGCATTCAAGTCCAAAAGGCTGTCTTTAAGGACTTTTATGATACTGTTGGAACCGTAAGTGGGGAAAAATCTGCATGCATGGGTGCATGCATGCAGGGAACATATGATAATCCATGACAGTCTGTGGTTAGCTGTTTTTCTTGTTAAAGTAAAACAGAAAGTGTAAAATAGGGAGAGAGCCAGACAGAGAATGGCATGCACTCCATCCAGTGACAAAAGTAGCTATGACAACTCTTCTTTCCCTTTACCTTTGCACTATAGGATGAGCAAGAGTACAGGAATGGATTGATATACAAGCTCCTTGTGGTTTACAGTGATGGTCAGTCCACAGAGCTATATCATTACACACATGATGCAACACACATCATCATAGATTTACATTATATGTGTACTGTGTCCATGCAGACACGGTCTGGCCTATATGAAACCCATGTATCCTATTGACGAAGCACTAGACTATATATTGAAAGCCTCATTAAGAGCAGTATGGCAGTGGTGACTCTGTTTATGTTGTGACAGTGCACCTGGGAAGCCTAGTGGAGCCCATGAAGGGCGTGTACGAGGCTTTTATTATTTGGTTTGCAGTACTGGGGCTTATGCCTTCCTCATTCAGACTAGACATAACAGTGTCTTTATTTTGATAGACAGCCCCCACTTTTCCAAAAGACAATCAGCAATAGGTTTTTAATTGCTTTATTATTCTTAGGTACCCAGAAAGAGGAAGAGTTGTGCGTCCGTTTGATTGACTCAAACACAAAACAGGTGGGTAGATTCATTAGTTCTGATATATTATTATTATTATTATTATTATTATTATTATTATTACTTATCAATGCTCTCTTTTTTTCTTTCAGCTGGTCCCATACGAAGGTACTAACAAGAATCCAGACTTTCGTAGGGTTCTCTTGACTCATGAACTGATATGCAGTCGCTGCCTCGAGAGAAGATCCTGTGGGAATAAGAACGACACGCCATCGGATCCGATCATACTCGACAAGTGAGTATATTTATAATAACAATTAGAATAATGGCTTAATTGTGTACCTGTGTTACAGTTCAAAAAATAAAATGTATTAAGAATCGGTGTGTAGTAATTGTATTCGCCTACCTTTCTTTGAAGAAGAAAGACACAAAGCGTTTAAGCATCTGCTCCTGTGGATAATTATCACAGTTGAGCAGAGAGCTAGAGTTTCCTGCTAATGCAATAATTTTTGCTCCAAGAGGAAACATGCGCCTGTAATTTTATAAACCACATGAGAAGAGACTCTTCCTCTATCTCCAGTCCCTCCCTTCCTCACACTTAAAAAAAATTGTGTTCAGTATGTAATACAGCCATAGGGCAATACACCCCTTTACTGGTACAATGTACAGTGGGATGGTCTACTATGTATATTGGTGTTGGGTTATTCGATACCTCTATTTAATGGCTGTCAGTGTCTTATTTTGTATGTAATATGAGGAGTATTTGTGGGTGTGTATCTCACTGTGTCTGTGTAAATACAATATAAGTGGGTGGGAGAGAGAGGGAGGGAGAGGAGGGGAGGGAAAGACACTGAGTGACCTTTACTTGTACATTATGATCTAGTCCATGCACATATTAATTATATGTATGTTCTTGCCTTTTAGTTTTCGGTTAAAGATCTTTGCAAAATGTAACCAGAACTGCTTGAAAAATGCCGGCAACCCGAAAGACTCAAGAAGACGATTTCAAGTAAGACACAAATCATTAAGGACCTTGCTTGAGATATAATGATCTTCTTCTCTTTTAAAGGTTGCTCTGTATGAAATTGATAAAGTCTCTGGCCAGCCCATTGCTTGCTCTGACTCAATGTTTGTTCACAATAACTCAAAACACGGGAGGAGACCTATTTATAGAGACAATGTTGTTGGAGATGGTAAGAGGGGGAGAGAGAGAGAGAGAAAGAGAGAGAGAGAAATTAATCCAATTATACATAACTATTCACTTTCTCTTTCTCTCCCTCTGTCTTTAGGGAAGCCTTGTATAGTAAGCATATACCCAAATGAAGGGTGGACAACTGGAGGCTCAAGGATAACTGTCATTGGAGTTAACTTCTTTGAAGGATTGGACATCGTATTTGGCACAGTCCCTGTTCAGAGTGAGGTAAGAAAAACAAGAGAGAGAGAGAAGCAGTCAATAAGAGAGTACACATAATGTCTGCCCTACTGTGCTACTGTGCATGGCATTAGTTGCTAAGCATTGACATGGGTTCTTGTGGCACAAGACAGTGACAGATGAGACAAGCCACATATTAATGAACTAATAATGGTAACCACTGTGGTGGGCAGTGGTAGCCATGGCAGCAATTAATTGTGGGCATCAATACAAGTACAATTTAATGTAATGATGTAACCCTCTCTCTCTCTCTCTCCCTCCCTCTCTCCCTCTCCCTCTCCCTCTCTCCCTCCCTCTCTCTCTCCCTCCATCTCTCTCTCTCCCTCCCTCTCTCTCTCCCTCCATCTCTCTCTCTCCCTCTCTCTCTCCCTAATAGGTTCTAAGTCCCAATGCTATTGCTGTGAGGACTCCCCCAGCCACTATGACTGGAGAAGTTGATGTTACGTTAATATTCCGTGCCTCTGGGGCTCAGTTTTGTATCAGCAATCCTGGGAAATTCTTATACACAGGTAATATGATAAAGAAAGAATACAATACTAATAGCAATAATGATACATTTAATAATAATGATGATATATAATGAATAATTGTATTTACTTTTTCTATTTTCTTTCAGCTCCTGATGATCAAATATTTGAGAACAGTTTCTGTAGAATCGAGAGAGTGATACGCCAACAAGACGACCCGGACCAACTACCAAAAGTACATACATACATACTTGTACATGCATCATCGCATTCTCTCTTCTCTTTCCTAACAGGATCTCGTCCTACAGAGGGCGGCAGAGCTCCTGGAGAGTTGCTTCCTAAGCTCAAGAGGAGCACAATCATCCACCGTCCCTTTTGGTCAGGCCCCGTTCGTGTACCCTCCCTCCCCGGCCCTCATTGGGAGCAACGGAGTGTTCCTCTTCACACCACAGCCCCTGACCCCCAGACAAGGCTCCAATGGTGGAATGCTCCCAATGAGTGAAGGACTAGGTGGTGGGTTTGGATACGGGGGCGGGATGCCGAACACTAACGGAGACGTCTATCCTCGACACAGTTACATGCCTCAGACTGCCTTCCCATTTCCCGAAATCACCCAACATGGTGACCCAGGTGGTTCAATGTCGACCGAAGTCAAATCAGAGTCTCAGATGTCTTCGCCGAGTCAACAAGGACTCCATAAATCAACGCTAGCGAATGGTATGGTAATGCCTTTTAACCTTCCTTACGAGACGGCTCCTCCGTATGGCAGTAACTCGCTACCACTTACTCACGGAGCAGGTCAAGTCAACACTAACAATAATAATAGTACCAGTGTCACCGACTCCGCCCACATTGGGCAAATGGCCGGCTCAAATTCGCTGGCGTACATGTCAAGGTTGTTTCCGGGTGGAGTCCCTCCATCTCCAGGTTTCTTAATGACGCATACTTGTAAGTTTTTTAACAATTTTCGTCGTTTTATTTTTTCCCGTTTTTATAGTTTCTCCGATACCAACCACACCCACTAGTATGAGTGGTGGAGGAGGGTTGAACAATCAGTTTTTTGCTTTTAATTCTCCTCTCCTCTCTCCCACCAAGGGAAGGATGGCCAAAACGGCTCGACCTAATCCTCTCTCCCTTTCAATGTCCAACGAGATGCCTAACNNNNNNNNNNNNNNNNNNNNNNNNNNNNNNNNNNNNNNNNNNNNNNNNNNNNNNNNNNNNNNNNNNNNNNNNNNNNNNATAGAAACCCACTACCAATATAACATTATGTTGTGTATCTATTGTACATGGATACTTCCATTGATAGAGGCATCTAATTGTACATGTATAGTACTTTCTATAACATACTGATCATATATACATGTATGTGTGTGCAATTTCATTCTCTTCCTCTCTCTCCATCAGGTATATCTTCACCTAATTTGGGATCAACCTTTACATTCCCTCCAATCACTCCATCAGCTCTCATTGCACCCATCAACCTACCAGGTACACATAGAGATTCTGTATACATATGCAATGTATGCATGTATGTGTGATACAAGAAAAGTACAATAGACACCATTATTATTATTTTTTATTAGTATTAATTTTACTTTTTTGTCTCTAGATCTGACAGGAGGTGGTGGCGGCTCTGAAGTACAGGATCCTCTCTCTGTTAATTCTGGAGCTACCAGTGGTAGCAGCACGCTCCACTCGACCAGTCTACCTCAAACCCCTCCCCCTCCTTCCTCTCGTAAAAGAAAGCATGATGGTAGTCCTACAACTGAGCTTCGTTAGGATCCAATGCTCAACACTTGACAGTCACTCTTATTATTATTATTATTATTATTATTACATTTCACTTCATTATCTTACAATTAAGCTAAATAAAACCATTTCTACTTATTATTGGTATCTGCTGTTATTATTATTATTATTGTTATTATTTATAGTCCAGAACTGTTTTAAATCCTCCTCATACTTTATTTATGTCACACATGCAACTCATCTCAGATACAGAAAGATAAAGCTTTATAATCTTCAGTTTAAAACTCAGTCTCTCTCTCTCCTCCTTCTCCTTCCGTAAAATTATTATTATTACCATTATTACTATTATTATTATCATTACGTGTATTATGTATTATGACTGTCTACTAATACTATGTCACTTCAACTTTGAAAAAGAACCCATTGTCAAAACCAAAACCAACTCTTAACAGTACATCCGAGGCACACACATTCATAAGTACACCAATCACAATAGCACTACAGTGCATGGCATAAAGGTTCACACTAAGAGCTTAATGTAATGAAATATCTCACACATTATTAAGTACGATATCAGACAATGTACAGCACCAAATTTTGGTCTTACAATAACAAAATTAATAAATTCTGCATTCATCTACAAAGCATAACTGTTGGTGATTGCTCTCCAATAAATAAATAATGTTTGCAATGAGTGTACTAAATCTTACGTCAAGTTGTCAACTAGATTCACGAGATAATGTGCAGCCTAGTGTGTGGCAGTTGGGCTTTAGTGAAAATTACAATCGCAGTCTTGTTGTAAAAATTATGATTCATTTTGATTATTGTTATTAGGGTAAACAATAAAGTTGATTAAAGTAATTATAATAACAAATAATAACAAACAACAGTAAGCATCTCTAACATGTTTTGATGCTTATCAATGTCTTTATGTTATTTCAAAGCTCAAGCCCATTTCGATAACAAACTTTTGGAGCTGTAGATTTTTGACATGAACTATCGCAATAGTCAATGGTCCTTAAAAATGGCTTTTAAAAAAGTGATAAGGACAATATGAGATTTCTGGTATAATTCATGCTCTAATGCAATGTAGAAGTTTGAGTCAATTCCTATATTTTAGATTCTTGTTACTTAAAGATGAGTCTGGCATTATACTATGCTGTACTAAAGATAATTACTACATTCCAGGCACTCCTGGGACTAGGCTAGTGCATTGAAGTGGGTTATGATGATTGGACACTATAATTGTAAGAAAAGAAGTGGTCACGACTCATTAGCCGTTGGACTAGCCAATAAATGCTCAGAAAAGAACCAACATACTATTCAACTAATGTATCAATGATGATGATCAATACTAAAAGAATTAATTTCATTTTCTATATAAACACACACTTCATAGCACAAGATATTCTCCACTAATTGTAGTCCAAACATACACACACACACAGAGAGAACAGGATGGGGTATCATAATGTACACAAAGACTCATTCTGTTTACCATTGTTCCATTGGGTCATCATTTATATACAAAGCTATTCCACTTAATCTATTCATTGAAGGTTTACAATGAGAACAAAACCAAACTATAAAATACATGAACAACTATACTTGGATATGTGCAGTATCAAGGATGAATAGCGTGGAGTGTACATTATTATTATACATAGATACATACATGTACATATACCTGTACATATATATTGTATTCATTTCATGGATGAGTGATACCCAAAGCATTTTTGTCCCGTTGATATCAAAACAGTACAAATACGCCAAACCATACAAACGATTGATATTACACTTTAATATTTGAATTGGGTCAATTATAGACACCAATAGTAGCCATACAGCCAATACTAGGGACCAGGGAGAGTATTAATTATACGCAGGTACACCAGTACTGATCCATCCAAAGGAGAAACCTTAATCCTGGACCCAATTATTCTACCACAACAGGCTGGCCAGCAGCTGTAAAGAAGTGACCCGATTTATAGTACTACTAGAACATATTGTGAACATTTGTTGTGGCTCATAAGTGTATGATATTGTCCCCTCTCTAGAGACTGTGGATGTACTATGCGAGAGAGAGAGGGAGAGGGAAGGAGGGGGAGAGACATAGTGTCAGTTTGAATGACAATTCCCCAATGAACTGGTTTAAGAGTTCAGCGGATGAGAGGTTTAGCGTGTTTACTACGCTCATCCAAATCTCTCTTTGTCTATCCCTCTCTCTCTCTCTCTCTCCCATATTTGTTCCATCTCCCCATTTACACAAAGAATAAATAATTATAGTATTAAATTAATATAGAGAATCAAAAAATTATTATCACACAGTGAAGGATACAAACAAATAACCAGAGGGAAGATATGACACACAGACGTTCATTATAATCAGTCATTGTTATCATTGCTTCCATCTGGTAAAGGGTTATCTAGTGATTGCTGAAGATTCTCAGTGGGAGATATCAACTGGGAGACATTAACATTTACTGTTTGAGACATGTGAAGTGGGGCAGTGATATCGGCAAAATTAACACCAGCTAAGTTAGCACCTCTGAGATTAGCGTGCTGCAAATCACAGCCACGCAAATCACAGTTCTGAGAGGAGAGAGAGAGGGGGGGAAGAGAAAAGAAAAATGACACAAAGATATAAAAGAGGAAGATTCGATTGAATGACATAAACTTGTACAAATATTCACTGATGTACATGTATACTAACAAGAAGACTCTTCACACACACACACACACACACACACACACACACACACACACACACACACACACACACACACACACACACACACACACACACACACACACACACACACACACACACACACACACACACCTTCACTCTCCATACCATTCAATTGATCAAGAAAGGCAGATTCTATATAACTACATCTTTAAGGTTGGTGCTTTCAAAATTTGAGCCTCTTAAATTGGCATGCTGCAAATCACTAGTGCTTAAATCACAATTCTACAGAAGAGGAAAACAAGAACAGAGGAGAAAGAGAGAGAGAGAGAGACGGAATTATTAAGCGGAACTGGGAATTCAATTTCCCTCTCCCACATCTAAAGGCAATATAAAGTACACATTTACAAAGAAGGAATGTCTGCTTCATAGTACTGCCAGTCTATGCGGATCCACTAGTAATGGCCATGATGTCACTATTAATGTTGGTTGGGGATTTGAAGACTATGGTAACCATTACACCTCAAGGGAAAGCTAAACGAATTGTAAAGAGTCTTCATGTATTGGATGCAGGTCTACACACGCTACCCACACAAGACCACAGAGTTCAATAACAGCAGCAGGAGTAAACAACTGACTATCACACACACACACACACACTCTTCTCTCTCTCTCTCTCTCTCTCTCTCTCTCTCTCTCTCTCTCTCTCTCTCTCTCTCTCTCCTCTCTTTTGCCCTATTCATACATATGCATGTCTATTTGTATCAAGAGCTGAAATAAAATCCAAATCTCTTGTCACATATCAATACCACAGTTGGATCCATTATAAGTAGTAGGCTGTTCAATATTCATATTATAATATTATGCAGNNNNNNNNNNGAGAAGAACTTAATTAGAAATCTTCTCTCTCTCTCTCTCTCTCATACCCAATACATACTGTATATGATTAGCACTACATTAGTTCATTCAATACATGCAGGTACAACTAATACCCGTGTAGCCACACCCACTAACCTCCATATCAGTTCCTGCCATTATTGCGTAGCGGAGGTTACAGGACCTGAGACACGCCCCCTTGAGACTCGCAAGACGGAGATTGACGCAACTCATCTGACTGTTATCAAAAGTTGCTGCCTTCAAGTTAGCCCCTTGGGAGATGCAAAGAGAAAGATAACTTACCACAGAGAGAGAGTGAGTTGACAAACAGAAAGCTAGTGTTGCTACTAGCAACTCTCCCAATAGTATACAGTAAATGGATCATCACTTATCACACACACCACACATGTACATGTACAGTGTAGCCTCTTTATTAAGGACACCATTGGGCCCACACTACCTGTCCTTAATACATGTACAGTGTAGCCTCTTTATTAAGGACACCATTGGGCCCACACTACCTGTCCTTAATACATGTACAGTGTAGCCTCTTAAATGTACTGCTAAAGCACTAATGCACATGTATATTGACAATCAAGACTATAACATTATACTGTGTTTGAGTCACTATTTCTCTCATTCCTTCTCTACCTTCAAGGTTAGTCGATACTCCTAGGCTGGCATCCATTATGCACTTCTTTAAACAAGCTCCTTCCATGACTACTCTGGGCATGTGAACACACTGAAGAACAGCATTCTAAAATTAAGAAATACACACATGTACATAAATGAATGAATGACAAATACATGTAGATGTAGTTAATGAGCATACATTGAGGGTAGCGTATGAGAGATCGGCTCTCTCAAGGACACAGTTGGTGAGATCACTGTGAGAGAAATCACAATATCTTAAATTAGCACACTTGAAGTTAATGTTCCTTAAATCGAGGTTAGAGAGGTCCACACCTTCCAAATTAATACCCTGAGAGAGAGAGAGAGAGAGAGAGGGAGAGAGAGAGAGAGGGAGAGAGAGGGGGAGGGAGAGGGAGAGAGAGAGAGAGGGAGGGAGAGAGAGAGAGGGATAGAAAACTAGAGCAATATGGTTACTTGACATCTAAGGACGGAAGAAGAGGAGGAACCAATGATCATCTGAAGGAACTCCTTTCTTGTTAAATGACCAGCTAATGAGAACTCTTCATTCTTTATGAGAAGAGGGAGAGAGAGGGGGGGGGAGAAAGGAAGAAAGAGGGGGGGAGGTAGAGGAGGAAAGGAGAGGAGGATGTGAATAATCAAATTTTGAAATAGAGTCTCAGACCTAATTGGACTACCAGACAGTTATTATTACCTTAACAAGTGTTTCCAGTGGTTGGATAGCTTTAGTAACATTGAAGAACTTGGCTTCCTCTAGCACTCCTATACACAAACACAACCATGGAGTCATTACCACAGGGACCCACACCCACAGCTTTAGCACGTACCCTGTGGGTTCACTCCTTCGTTAATAATGAGCTTCCCATGTCTCATGAAGTTGAGAAGTGGCTCAAAATATTCAGGACTCCTGTCAATGAGGTATGCCCCGGAGTCATCCGTAGCACTGAACCAATCTAAGGACAGAGTATAATGTATCAAACTGTATATATAATGAATGGAAATAATCGAAATGACTACAGTATATAATTATTATGAAATTATGTCTATACGTGATTTAATTATGGCATGAATATGTATTTTCAAAATGATTTAAAGCATTCACGTGTAGTAGAAATGAATTTCACAGTAAAATCTGCTAATAAACCTACTGTAGTTATCATTTTAATACTTAAATAATTCAATATAATAATAATAATTATTATTATGATTTATTTATTTATTTTACTACCTGACTCAAACATTCTAGCCAACATTGAGGATGGATCAGAAGTGATGGTTGCCCTGCAGTTACATTAATAGTTATTAATAGTTATTAAATGACACGCCCACCTGCTAGTGGCAAATACTTTCCCGCCTACATTCAACCTCACCCAGTCATGACGTGAAGGAGCATCTAGAAACGGACAAAAAGTGATTAAAATACCCAAAGAAGATTACCAGGGGAAAGGAGCCTTGAGCACTTTTTAAGGAGGAAATGACTTTGATCAGTTTGAGGGCCGAGTTCGGGTAGCTGGTCCTCAGGGAGGTCGCTTAGTATAATGGGAGAGGAGGGTGTGGCTCTTGTTGACTCTAAACATGAAATGAAATGTACCTAAGAGGTAGATGCAACACAAACCTGTGCTTGAGGTAACAGCAATTAGAATGTCATCCTCTCTAGAAAGAGACGGAGAGAGAGAGAGAGGGAGAGAGAGAGAGAGAGAGAGAAAATGAATTTTTGTACGAACATGACTAAATGAAAGAGAAAGGGATTGAGGGAAAAGAGAAAAAAGCTAAAAAATAATAATAATAAATAATAGTGTGGCAGTGATTCTTAGAATCATGACTGCATGGTGCCAACTGAAGGAGCTTCTGAAAGACCTCAAAATGAAAGCAGACATAATAAGAATTAAGACTATCATAATAAGAATTAAGACTATCATAATAAAACTGAATTTCAATCTTTAGTCACCTCTAAATTGATGCAGACCTGATAAAAATAATTATCAAGTGATAAAGCTTCATTAGAGGTTACCTTAATTGCTACTTTTATAACAAACATTAGATAGATGTAATGGTTTTGGACTACGTTACGCAATAAAAAGGGAAAGAGAGAAAGAGACATATATGTACGTGTAATATAGCAAAAGGGAAGCCATTACTTATTATTATTATACATGTATACATGTATACACGTGTAGATGTCAACAATTAAATAGATGCTGTAGAAATTAATACAGTATATGTATATACAAGGAGACACTGTATAAACACTCCCTCAAACCAGGGATCATCTGGTTAAGCCACAAAGAGTGAGGCTATGTATACAGAGCCTCCCAGACCAGCTGGTCAGTGAGACTCCCTCTTGGGAGCTCTGATGAGTTTGTAAAACTACTAGCAATCAATAAGCCCAGCCAGCTGTATAGACAAGATCTACTCCTCTAACAGTTTTAAAAGTGGCCCCAAATGACTAATTGGGGTACTATCAAATGAGCCTGTGGTTTCAAGCCTACTACACTCCTTTGATTCAAGAGACCAGCATCAGCTGGTATCACTACACGTGGCGACTCAAAAATTGAATTATTTGAAAAGGGTCTTGAAGCCAGAGAGGAGTGCGTCAACAAGGAGTGGTGGGACTGTTACTCGATACACTTGAAATTCGAAAGAGACTACGTGTAAGGATTCATTAAAATAACATTAGGGCAATAGAGACCAGCTGGGTTACACACGCACACACACACATGTACTATGAATATAGAACAAAGACTGTATAATAATGATGTACATCTATTAGCAGGGGACTCTTATTTAGTAGAGGAACTATGTGGAGGTTGGTGCCAAATTAAGATCAAGAATGCAATCCTAGAGCAGTTAACATGCAATGCATTTCATGTAGATTGATTTCAATCTTGAAGTTGCTAAGGAATTTGGCCAAAAACCATAGGCTATAACAGGGTATTTTGTCAAAAATGAAAAGGAGAGAAATTTCCATTTTTATAATTATTTCTGATACATATGCATGTTCTTATGTTATGTTTCAAGTTTGAGCCAATTCCTATTAAAATTGAATTTTTTACAGATCGTTAAAGTTGCTCCAAAATTGGGTCAAAGTCCCTGTACTATATACAGGGTCTTTGGCCAAATTTCATCAAAAATGACTAGGAGAGAATTCTTCATTTTGTAGTATTTTTTTATACATATACATGTTCTTATGTTGTGTAGCAAGTTTGAGCTGATTCCGGCCAAAATTGAATTTTTTACAAATTTTTAAAGTTGTTTCAAAATTGGGTCAAAGTCCCTGTACTATAGTACAGGGTCTTTGGTCAAATTTCGCCAAAAATGAAAAGGAGAGAAATCTCCATTTTTATAATTTTTTCTGATACGTATACATGTACTTATGCTATGTTTCAAGTTTGAGCTGGTTTCAAT

>genscan_predicted_peptide

MAHESSLSPPVPSGEHIPPPLINHTLPQRSTPYYIAAAQFEKQPPDNMRKSNFFHFIISL

FDGQQHRIQVQKAVFKDFYDTVGTLVPYEGTNKNPDFRRVLLTHELICSRCLERRSCGNK

NDTPSDPIILDNFRLKIFAKCNQNCLKNAGNPKDSRRRFQVALYEIDKVSGQPIACSDSM

FVHNNSKHGRRPIYRDNVVGDGKPCIVSIYPNEGWTTGGSRITVIGVNFFEGLDIVFGTV

PVQSEVLSPNAIAVRTPPATMTGEVDVTLIFRASGAQFCISNPGKFLYTAPDDQIFENSF

CRIERVIRQQDDPDQLPKDLVLQRAAELLESCFLSSRGAQSSTVPFGQAPFVYPPSPALI

GSNGVFLFTPQPLTPRQGSNGGMLPMSEGLGGGFGYGGGMPNTNGDVYPRHSYMPQTAFP

FPEITQHGDPGGSMSTEVKSESQMSSPSQQGLHKSTLANGMVMPFNLPYETAPPYGSNSL

PLTHGAGQVNTNNNNSTSVTDSAHIGQMAGSNSLAYMSRLFPGGVPPSPGFLMTHTCISS

PNLGSTFTFPPITPSALIAPINLPDLTGGGGGSEVQDPLSVNSGATSGSSTLHSTSLPQT

PPPPSSRKRKHDGSPTTELR

>geneid_v1.2_predicted_protein_1|688_AA

TSDAMSYVAGYTVAHDVSARDWQMKKNGKQWLLGKTFDTFCPLGPALVTTPSISDVLVDR

SSWLIMKGRLFVDKIQVKTIKTQSRSQACTSTNLPVEGLAFWEYGCSLFVLSWGLLVKVA

KRKVIAFALVPPLGIYSKEHYMHYACKATHATCRLELLSWNYKSTPYYIAAAQFEKQPPD

NMRKSNFFHFIISLFDGQQHRIQVQKAVFKDFYDTVGTDEQEYRNGLIYKLLVVYSDGTQ

KEEELCVRLIDSNTKQLVPYEGTNKNPDFRRVLLTHELICSRCLERRSCGNKNDTPSDPI

ILDNFRLKIFAKCNQNCLKNAGNPKDSRRRFQVALYEIDKVSGQPIACSDSMFVHNNSKH

GRRPIYRDNVVGDGKPCIVSIYPNEGWTTGGSRITVIGVNFFEGLDIVFGTVPVQSEVLS

PNAIAVRTPPATMTGEVDVTLIFRASGAQFCISNPGKFLYTAPDDQIFENSFCRIERVIR

QQDDPDQLPKDLVLQRAAELLESCFLSSRGAQSSTVPFGQAPFVYPPSPALIGSNGVFLF

TPQPLTPRQGSNGGMLPMSEGLGGGFGYGGGMPNTNGDVYPRHSYMPQTAFPFPEITQHG

DPGGSMSTEVKSESQMSSPSQQGLHKSTLANDLTGGGGGSEVQDPLSVNSGATSGSSTLH

STSLPQTPPPPSSRKRKHDGSPTTELR
